# A Novel Ferroptosis-Associated Gene Signature to Predict Prognosis in Patients with Uveal Melanoma

**DOI:** 10.3390/diagnostics11020219

**Published:** 2021-02-02

**Authors:** Huan Luo, Chao Ma

**Affiliations:** 1 Charité—Universitätsmedizin Berlin, Corporate Member of Freie Universität Berlin, Humboldt-Universität zu Berlin, and the Berlin Institute of Health, 13353 Berlin, Germany; huan.luo@charite.de; 2 Klinik für Augenheilkunde, Charité—Universitätsmedizin Berlin, 13353 Berlin, Germany; 3 Berlin Institute of Health Center for Regenerative Therapies and Berlin-Brandenburg Center for Regenerative Therapies (BCRT), Charité—Universitätsmedizin Berlin, 13353 Berlin, Germany

**Keywords:** uveal melanoma, ferroptosis, gene signature, prognostic value, tumor immunity, target therapy

## Abstract

Background: Uveal melanoma (UM) is the most common intraocular tumor in adults. Ferroptosis is a newly recognized process of cell death, which is different from other forms of cell death in terms of morphology, biochemistry and genetics, and has played a vital role in cancer biology. The present research aimed to construct a gene signature from ferroptosis-related genes that have the prognostic capacity of UM. Methods: UM patients from The Cancer Genome Atlas (TCGA) were taken as the training cohort, and GSE22138 from Gene Expression Omnibus (GEO) was treated as the validation cohort. A total of 103 ferroptosis-related genes were retrieved from the GeneCards. We performed Kaplan–Meier and univariate Cox analysis for preliminary screening of ferroptosis-related genes with potential prognostic capacity in the training cohort. These genes were then applied into an overall survival-based LASSO Cox regression model, constructing a gene signature. The discovered gene signature was then evaluated via Kaplan–Meier (KM), Cox, and ROC analyses in both cohorts. The Pearson correlation coefficient examined the correlations between risk score and UM common mutations and autophagy. The analyses of GSEA and immune infiltrating were performed to better study the functional annotation of the gene signature and the character of each kind of immune cell in the tumor microenvironment. Results: A seven-gene signature was found from the training cohort and validated in all cohorts by Kaplan–Meier and Cox regression analyses, revealing its independent prognosis value in UM. Moreover, ROC analysis was conducted, confirming the strong predictive ability that this signature had for UM prognosis. A total of 52.24% (256/490) autophagy-related genes were significantly correlated with risk scores. Analyses of GSEA and immune infiltrating detailed exhibited specific pathways associated with the seven-gene signature, also confirming the crucial role that Mast cells resting played in the prognosis of the seven-gene signature. Conclusions: In this study, a novel ferroptosis-related seven-gene signature (ALOX12, CD44, MAP1LC3C, STEAP3, HMOX1, ITGA6, and AIFM2/FSP1) was built. It could accurately predict UM prognosis and was related to Mast cells resting, which provides the potential for personalized outcome prediction and the development of new therapies in the UM population.

## 1. Introduction

Uveal melanoma (UM) is the most common ocular malignant tumor in adults, with an overall mortality rate of 50% [1]. Although UM is rare, it accounts for 85–95% of all ocular melanoma cases [2]. About 85% of the tumor cases arise from the choroid, while the remaining cases arise from the iris (3–5%) and ciliary body (5–8%) [2,3]. Approximately 40% of UMs have metastatic disease, of which the liver is the most affected site, causing a high mortality rate [4,5]. The primary disease treatment is the surgical removal of the tumor, but conservative approaches, such as radiotherapy, are also often adopted in clinical practice [6]. With the deepening of research, although considerable progress has been made in the diagnosis and treatment of primary UM, the survival rate has not improved significantly in the last three decades [1].

Ferroptosis is a newly introduced type of programmed cell death discovered in recent years. The process of ferroptosis is usually accompanied by a large amount of iron accumulation and lipid peroxidation [7]. This is closely related to the maintenance of homeostasis and the development of diseases, especially cancer [8]. The induction of ferroptosis leads to mitochondrial dysfunction and toxic lipid peroxidation in cells, which play a key role in inhibiting the growth and development of cancer [8]. In the past few years, ferroptosis has been found to be a promising trigger option for cancer cell death, especially for malignant tumors that are resistant to traditional therapies [9,10,11]. Ferroptosis is a double-edged sword in tumor development because ferroptotic cancer cells release a variety of signaling molecules, either to inhibit tumor growth or to promote tumor proliferation [12]. The role of the signals released from ferroptotic cancer cells in tumor microenvironment needs further investigation [12]. A recent report suggests that ferroptosis suppress metastasis in blood, but ferroptosis is suppressed in the lymph [13]. Therefore, it is essential to address how ferroptosis genes affect the prognosis by analyzing the correlation between each ferroptosis gene and tumor patients’ prognosis.

Currently, several studies are mining the prognostic gene signature related to ferroptosis in tumors from public databases [14,15]. Liu confirmed that the ferroptosis-related nineteen-gene signature could predict glioma patient survival [14]. Liang et al. discovered a novel ferroptosis-related prognostic gene signature for hepatocellular carcinoma [15]. However, there is still no study declaring whether a ferroptosis-related prognostic gene signature can predict UM prognosis. In order to fill this gap and widen the options in the diagnosis and therapy of UM, the present study performed comprehensive analyses utilizing The Cancer Genome Atlas (TCGA) and Gene Expression Omnibus (GEO), along with ferroptosis-related genes identified in previous studies, to determine and validate the optimized prognostic genes of UM. Besides, the gene signature characteristics in the tumor microenvironment were studied through GSEA and immune infiltration analysis.

## 2. Materials and Methods

### 2.1. Cohorts and Ferroptosis-Related Genes

The dataset with project ID: TCGA-UVM (80 UM patients) was chosen as a training cohort and downloaded from the GDC Xena Hub (https://gdc.xenahubs.net). For validation, an independent cohort, GSE22138, which contained 63 UM cases, was selected from the Gene Expression Omnibus database (GEO, https://www.ncbi.nlm.nih.gov/geo/). A comprehensive list containing a total of 103 ferroptosis-related genes was retrieved from GeneCards [16] (https://www.genecards.org/) and are provided in Table S1.

### 2.2. Identification and Validation of the Prognostic Ferroptosis-Related Gene Signature

Kaplan–Meier and univariate Cox regression were conducted in the training cohort to screen potential prognostic genes. Only genes that showed significant (*p*-values < 0.05) in both Kaplan–Meier and Cox analysis were considered as potential prognostic genes. The genes in the overlapped part of potential prognostic genes and ferroptosis-related genes were identified as potential prognostic ferroptosis-related genes, which ewre then entered into an overall survival-based LASSO Cox regression model in the training cohort. The LASSO analysis with ten cross-validations were conducted by applying the “glmnet” R package study, the best penalty parameter lambda [17,18,19,20]. According to the optimal lambda value, a prognostic gene list with coefficients was generated from the LASSO model. As shown in the following formula, each patient’s risk score can be obtained from the gene expression level and corresponding coefficients. In the formula, n, Expi, and βi represented the number of hub genes, gene expression level, and regression coefficient value, respectively.

(1) $$Risk\;score=\overset{n}{\underset{i}{\sum }}Expi*\beta i$$

In the training cohort, patients were divided into low- and high-risk groups using the median risk score as a cut-off point. The survival difference between the two groups was measured by Kaplan–Meier analysis. Cox and ROC analyses were also conducted for further assessment of the gene signature prognostic ability. Moreover, in the validation cohorts, the same formula and statistical methods were adopted to validate the prognostic capacity of the gene signature.

### 2.3. Correlation between Gene Signature and UM Common Mutations

In UM, chromosomal aberrations and gene mutations are closely related to treatment options and prognosis. Most of the samples in the validation cohort own the information on chromosome 3 status. Besides this, Robertson and collogues detailed studied the status of chromosome 3, 8q, and 6p of every patient in the TCGA-UVM project in their latest public research [21] (Table S2). The Pearson test was conducted to examine the correlation between copy number aberrations and gene signature risk score in the TCGA-UVM cohort. 

### 2.4. Relationships between Gene Signature and Autophagy in UM 

Autophagy is a conserved intracellular degradation system that plays a dual role in cell death; thus, therapies targeting autophagy in cancer are somewhat controversial [22]. Accumulating studies have revealed crosstalk between autophagy and ferroptosis at the molecular level [22]. To explore the relationship between autophagy and our gene signature, we first identified 232 autophagy-associated genes from the Human Autophagy Database (HADb; http://www.autophagylu/index.html), which contains an exhaustive, up-to-date list of human autophagy-related genes [23]. Another 363 autophagy-related genes were retrieved from the Molecular Signatures Database (version 7.1, https://www.gsea-msigdb.org/gsea/msigdb/index.jsp). Through merging them, a list of 490 autophagy-related genes was obtained (Table S3). Pearson test was performed to examine the correlation between autophagy and gene signature risk score. 

### 2.5. Gene Set Enrichment Analysis

GSEA, using the JAVA program (http://software.broadinstitute.org/gsea/index.jsp), was employed for assessing the possible mechanisms between high- and low-risk groups based on the Hallmark (v7.1, https://www.gsea-msigdb.org/gsea/downloads.jsp) gene set collections. The number of random sample permutations was set at 1000, and | NES | > 1, NOM *p*-value < 0.05, and FDR q-value < 0.25 were set as the significance threshold.

### 2.6. Relationship of Gene Signature and the 22 Tumor-Infiltrating Immune Cells (TICs)

The relative proportion of 22 TICs in the training group was calculated using the CIBERSORT algorithm [24,25]. After the quality filtering (*p*-value < 0.05), 43 UM cases were qualified for subsequent analysis. The Pearson coefficient examined the correlations between 22 kinds of TICs. To identify the relationship between 22 TICs proportion and risk score, an integrated analysis of the Spearman coefficient and Wilcoxon rank-sum was applied. Besides, univariate Cox and Kaplan–Meier analyses were deployed to screen 22 TICs with prognostic meaning using TICs proportion and survival data.

### 2.7. Statistical Analysis

Kaplan–Meier analysis was conducted using “survival” and “survminer” R packages. Cox proportional hazard regression analyses were performed using the “survival” R package. ROC analysis was conducted using the “survivalROC” R package. *p*-value < 0.05 indicates statistical significance.

## 3. Results

### 3.1. Characteristics of UMs

**Figure 1:**
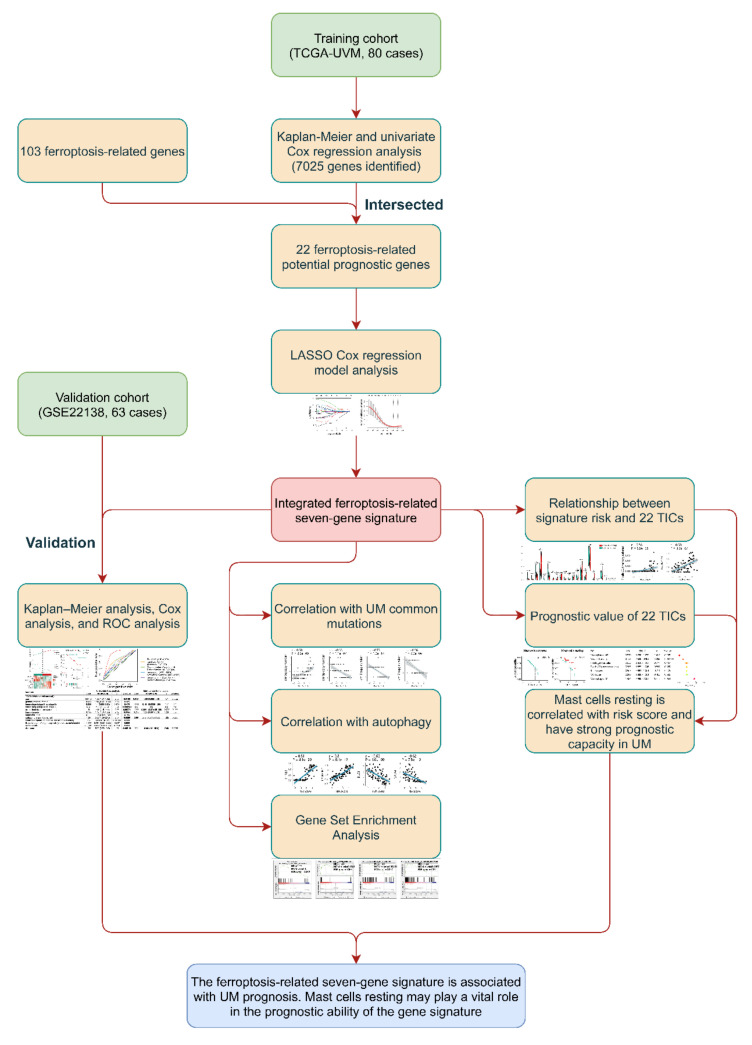
Flow chart of the study. LASSO: the least absolute shrinkage and selection operator Cox regression model; UM: uveal melanoma; ROC: receiver operating characteristic; TICs: tumor-infiltrating immune cells.

The present study’s flow diagram is displayed in Figure 1. A total of 80 UM patients in TCGA-UVM dataset were treated as the training cohort. GSE22138, a dataset from the GEO database, contained 63 UMs, was selected as the validation cohort. In Table 1, the clinical characteristics of each patient included in this study were summarized.

### 3.2. Identification of Prognostic Ferroptosis-Related Gene Signature

**Figure 2:**
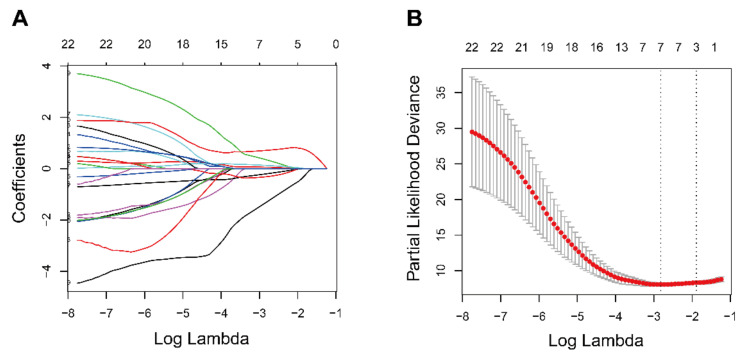
Prognostic gene signature construction using he least absolute shrinkage and selection operator Cox regression model (LASSO). (**A**) Distribution of LASSO coefficients of the 22 ferroptosis-related potential prognostic genes in training cohort. (**B**) The generated coefficient distribution plots for the logarithmic (lambda) sequence for the selection of the best parameter (lambda).

The analyses of Kaplan–Meier and univariate Cox were conducted over the training cohort, and 7025 potential prognostic genes were identified (Table S4). The potential prognostic genes and 103 ferroptosis-related genes were intersected to obtain a list containing 22 ferroptosis-related potential prognostic genes (Table 2). The 22 ferroptosis-related potential prognostic genes were then subjected to an overall survival-based LASSO Cox regression model (Figure 2A). When seven genes were gathered, the regression model reached the optimal ability (Figure 2B). The regression coefficient of each gene was calculated and shown in Table 3.

Drivers are genes that promote ferroptosis. Suppressors are genes that prevent ferroptosis. Markers are genes that indicate the occurrence of ferroptosis.

### 3.3. The Prognostic Capacity of the Seven-Gene Signature

**Figure 3:**
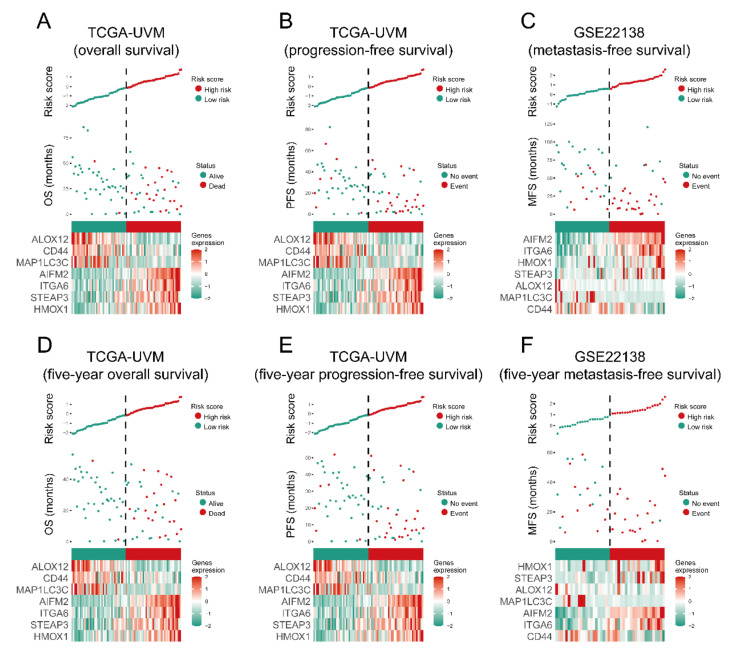
The overall performance of the seven-gene signature in all cohorts. The upper and middle parts of each plot (**A**–**F**) indicate the distributions of risk score and patients’ survival time, respectively. Bottom parts show heatmaps of seven gene expression profiles.

Each UM case’s risk score was a linear combination of each seven-gene signature expression level and its risk coefficient. Patients were sorted into high- and low-risk groups based on their median. The distribution of risk scores, outcome status, and gene profiles of the seven-gene signature in training and validation cohorts are shown in Figure 3. As demonstrated in the figure (Figure 3A–C), more events happened in the high-risk groups than that in their corresponding low-risk groups. Additionally, the patients in high-risk groups had less survival time than those in the respective low-risk groups. Besides this, we checked the capacity of the seven-gene signature from five-year survival (Figure 3D–F) and found more events and less survival time in the high-risk groups, which was consistent with the results shown in Figure 3A–C. The heat maps show that AIFM2/FSP1, ITGA6, HMOX1, and STEAP3 were overexpressed, while MAP1LC3C, CD44, and ALOX12 were downregulated in high-risk cases.

**Figure 4:**
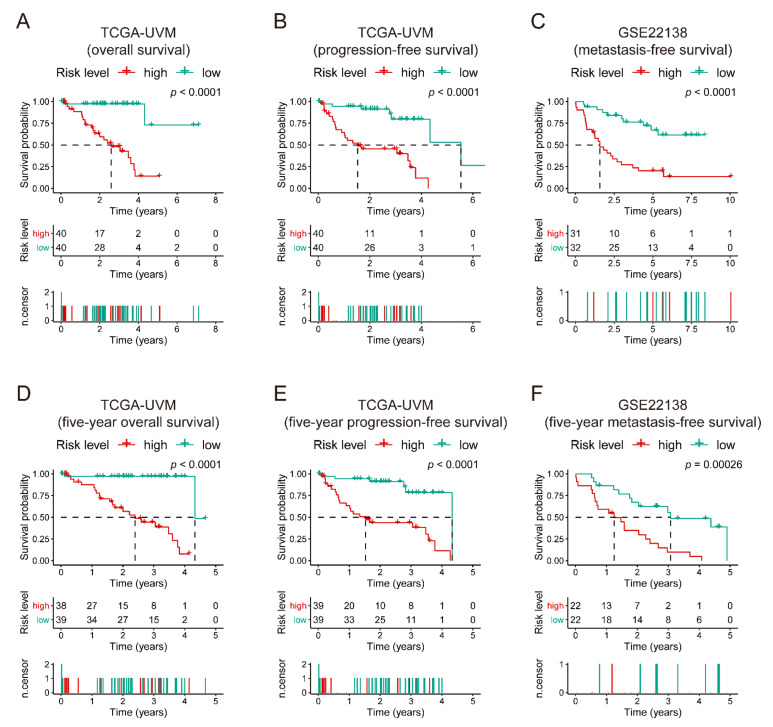
Kaplan–Meier curves of the seven-gene signature risk score in all cohorts. The middle part of each graph (**A**–**F**) indicates the number of patients at risk. The differences between the high- and low-risk groups were measured by log-rank (*p*-value < 0.05).

Kaplan–Meier curves displayed that the high-risk patients have poor overall survival and progression-free survival in TCGA-UVM (*p*-value < 0.0001, Figure 4. Kaplan–Meier curves of the seven-gene signature risk score in all cohorts). The middle part of each graph indicates the number of patients at risk. The differences between the high- and low-risk groups were measured by log-rank (*p*-value < 0.05).

Figure 4A,B showed poor metastasis-free survival in GSE22138 (*p*-value < 0.0001, Figure 4C) compared to specific low-risk patients. The Kaplan–Meier curves of five-year survival showed the same pattern as high-risk score groups, which had significantly unfavorable outcomes compared to those in their corresponding low-risk groups (*p*-value ≤ 0.00026, Figure 4D–F).

Univariate and multivariate Cox models were built in both cohorts using the overall, progression-free, or metastasis-free survival data and other available co-variables, including risk score, gender, age, ethnicity, tumor stage, mitotic count, radiation therapy, chromosome 3/6p/8q status, etc., to validate the prognostic capacity and the independence of the seven-gene signature among other clinic-pathologic characteristics (Table 4). In the overall survival-based Cox regression model of the training cohort, both univariate and multivariate results suggested that the seven-gene signature was a powerful player (HR = 5.22, 95% CI = 2.59–10.5, *p*-value = 3.99 × 10^−6^, and HR = 68.6 95% CI = 3.36–1400, *p*-value = 0.00598, respectively). Consistent with those in the training cohort, either in univariate or multivariate analysis, the seven-gene signature showed excellent ability, in an independent validation cohort, to predict metastasis-free survival (*p*-value <= 0.0388). We also utilized progression-free survival data in the training cohort to perform the Cox analysis, and found that the seven-gene signature had the ability to predict the outcomes not only in univariate, but also in multivariate models (*p*-value <= 0.0334). These pieces of evidence indicate that the seven-gene signature was an independent and prognostic variable.

**Figure 5:**
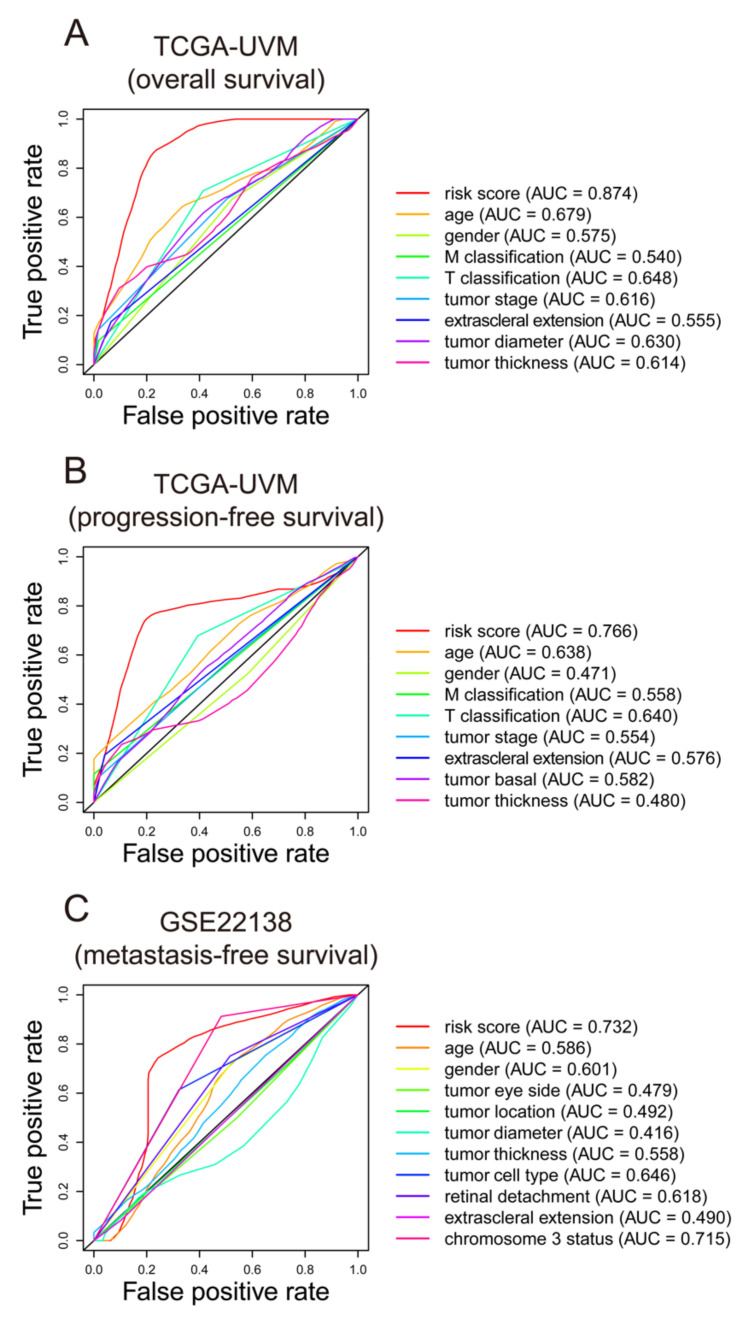
ROC curves of the seven-gene signature. The ROC curves were constructed by risk score, age, gender, T classification, M classification, tumor stage, etc. to show the prognostic ability of each variable. The ROC analyses were conducted in TCGA-UVM cohort based on overall survival and progression-free survival (**A**,**B**), and in GSE22138 cohort based on metastasis-free survival (**C**). ROC: Receiver operating characteristic; AUC: area under the ROC curve. TCGA: The Cancer Genome Atlas; TCGA-UVM: A project ID in The Cancer Genome Atlas database.

We conducted ROC analysis to evaluate the seven-gene signature performance in predicting UM outcomes. As displayed in Figure 5A, the risk score AUC in the TCGA-UVM cohort ranked the highest among other clinical characteristics, which was 0.874. The AUC of the seven-gene signature in the TCGA-UVM cohort predicting progression-free survival reached 0.766, which was the leading variable compared to other factors (Figure 5B). In the GSE22138 cohort, the AUC of the gene signature was 0.732, which was the best and superior to the chromosome 3 status (AUC = 0.715) (Figure 5C).

### 3.4. Identification of the Correlation between Seven-Gene Signature and UM Common Mutations 

**Figure 6:**
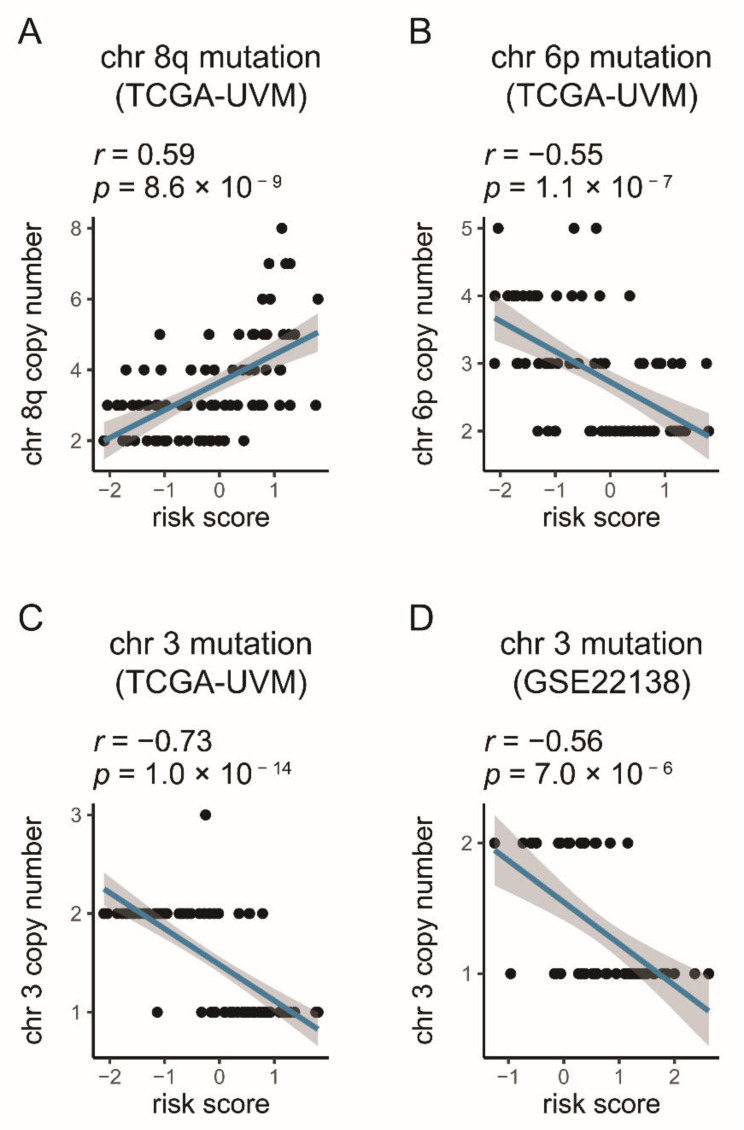
Correlations between the gene signature and the chromosome aberrations in UM (**A**–**D**). The blue line in each graph fits a linear model that indicates the proportional trend of copy number and the risk score. The grey shading around the blue line indicates the 95% confidence interval. The correlation examination was conducted by the Pearson coefficient.

The chromosome copy number aberrations of each UM patient in the TCGA-UVM cohort were downloaded from Robertson’s public research (Table S2) [21]. The Pearson test was conducted to evaluate the correlation between the gene signature and the chromosome copy number aberrations. The results exhibited that, in the TCGA-UVM, the gene signature was negatively correlated with chromosome 3 copy number (R = −0.73, *p*-value = 1.0 × 10^−14^) and 6p (R = −0.55, *p*-value = 1.1 × 10^−7^), and positively correlated with chromosome 8q copy number (R = 0.59, *p*-value = 8.6 × 10^−9^) (Figure 6A–C). As in TCGA-UVM, the chromosome 3 copy number in GSE22138 had a negative correlation with risk score (R = −0.56, *p*-value = 7.0 × 10^−6^) (Figure 6D).

### 3.5. Identification of the Autophagy Correlation with the Seven-Gene Signature

**Figure 7:**
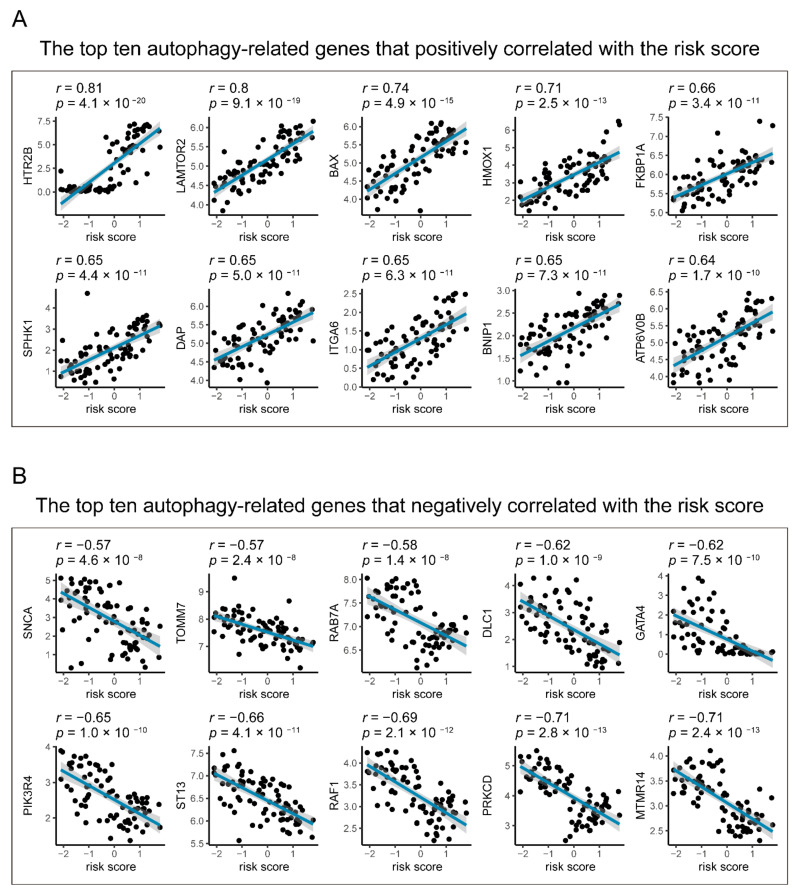
Correlations between the gene signature and the autophagy-related genes in UM. The blue line in each graph (**A**,**B**) fits a linear model that indicates the proportional trend of the expression level of each gene and the risk score. The grey shading around the blue line indicates a 95% confidence interval. Pearson coefficients examine the correlation test. Only top ten positive and negative correlations are plotted. UM: uveal melanoma.

Moreover, the Pearson correlation coefficient was used to evaluate the relationship between autophagy-related genes and the seven-gene signature risk score. Of the 490 autophagy-related genes, 256 (52.24%) were significantly correlated with risk scores, of which 197 were positively correlated and 59 were negatively correlated (Table S5). As shown in Figure 7, HTR2B, LAMTOR2, BAX, HMOX1, FKBP1A, SPHK1, DAP, ITGA6, BNIP1, and ATP6V0B are the top ten autophagy-related genes that positively corrected with the risk score, while MTMR14, PRKCD, RAF1, ST13, PIK3R4, GATA4, DLC1, RAB7A, TOMM7, and SNCA are the leading ten that have negative relationships with the seven-gene signature risk score.

### 3.6. Gene Set Enrichment Analysis

**Figure 8:**
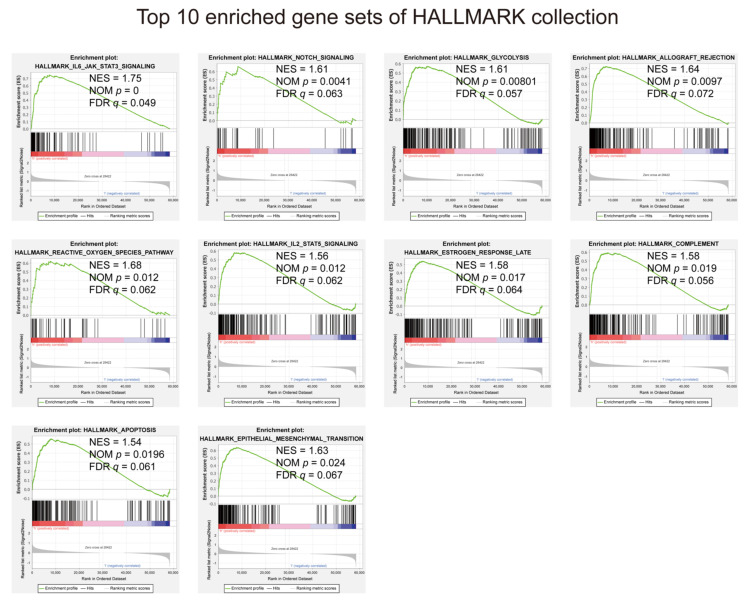
Gene set enrichment analysis performed using HALLMARK collection. |NES| > 1, NOM *p* < 0.05, and FDR *q* < 0.25 are set as the significance threshold.

In view of the negative correlation between risk score and UM prognosis, we conducted GSEA between high- and low-risk groups. The results displayed that all enriched gene sets were in the high-risk group, mainly involved in mechanisms related to IL-6/JAK/STAT3 signaling, notch signaling, glycolysis, transplant rejection, reactive oxygen species, IL2, estrogen, complement system, apoptosis, and epithelial–mesenchymal transition (Figure 8 and Table S6).

### 3.7. Identification of the Relationship between the Seven-Gene Signature and 22 TICs

**Figure 9:**
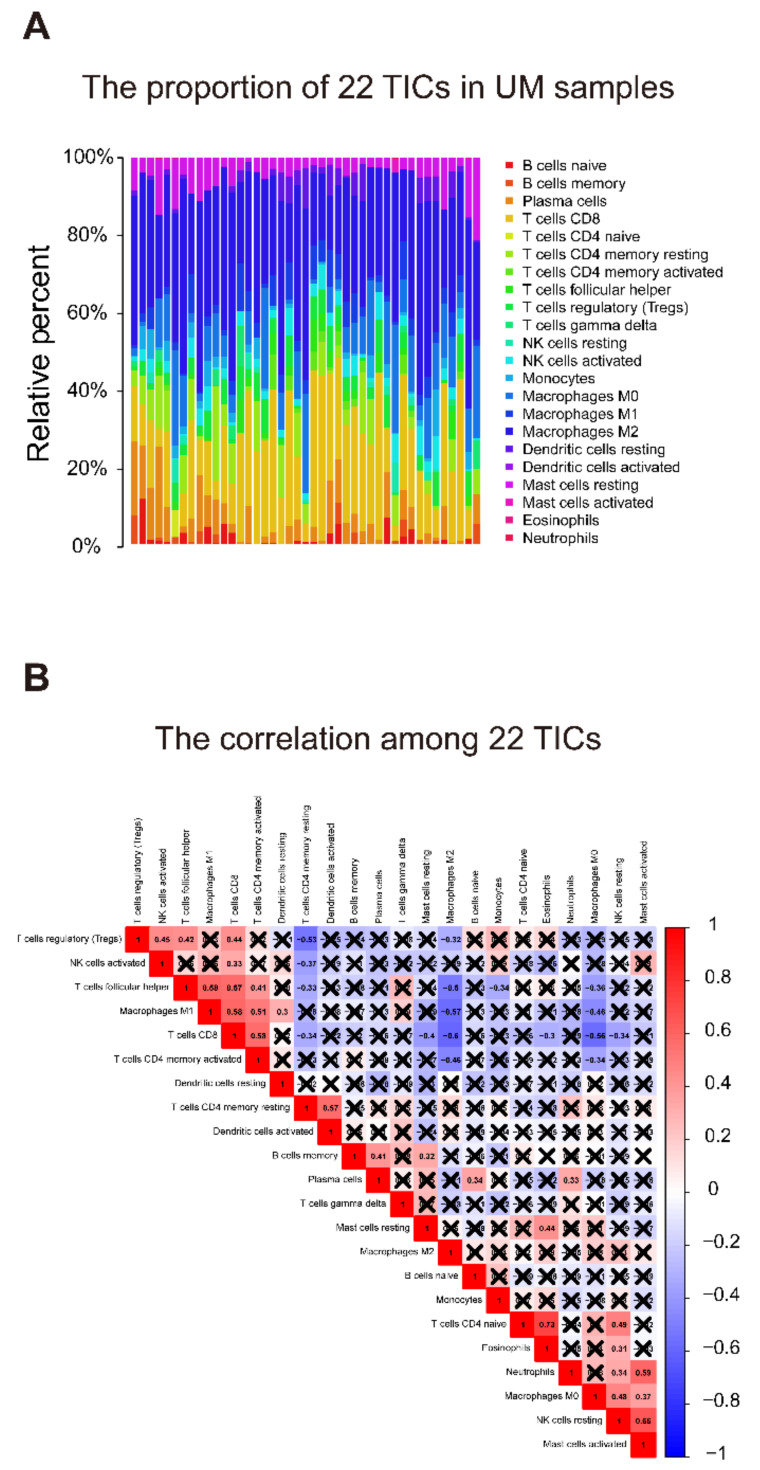
TIC distribution map and correlation analysis of UMs in the training cohort. (**A**) The bar graph shows the relative content distribution of 22 TICs of UMs in the training cohort. Columns represent UM cases. (**B**) The heatmap shows the correlation between 22 TICs. The color and number in each box indicate the coefficient between the two TICs. The coefficient of X-shaped coverage is not significant. Correlation test is conducted by the Pearson coefficient. *p*-value < 0.05 is the significance threshold. UM: uveal melanoma; TIC: tumor-infiltrating immune cell.

To better study how the seven-gene signature and the immune microenvironment interact, the CIBERSORT algorithm was applied, and comprehensive comparisons with the risk score were made. The relative content distribution of 22 TICs in the TCGA-UVM cohort, and the correlation between 22 TICs are shown in Figure 9.

**Figure 10:**
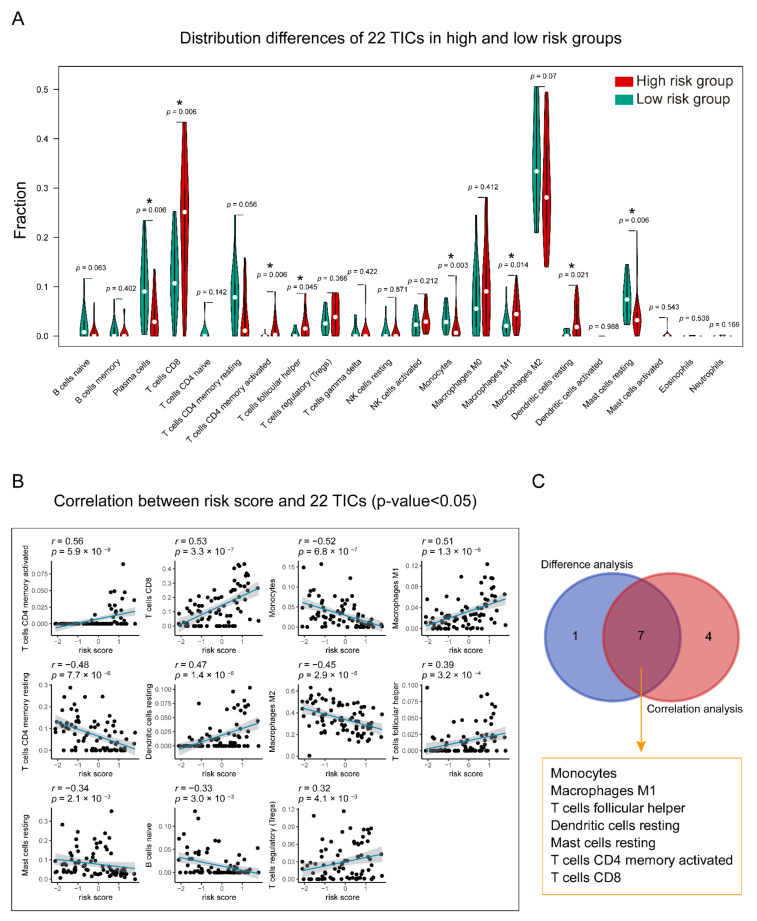
Relationship between TICs and seven-gene signature risk score. (**A**) The Violin plot shows the ratio differentiation of each of 22 TICs between high- and low-risk groups. Wilcoxon rank-sum was applied for the significance test. (**B**) The correlations between the TICs and seven-gene signature risk score (only correlations with significate were plotted). The blue line in each graph fits a linear model that indicates the proportional trend of the TICs and the risk score. The grey shading around the blue line indicates the 95% confidence interval. Correlation test is conducted by the Spearman coefficient. (**C**) The Venn diagram exhibits that the seven TICs have a strong correlation with the risk score. This strong correlation is co-determined by the results of the violin and scatters plots. *p*-value < 0.05 is the significance threshold. UM: uveal melanoma; TIC: tumor-infiltrating immune cell; * *p*-value < 0.05.

Comprehensive analysis of the results of difference (Figure 10A) and correlation analyses (Figure 10B and Table S7), seven TICs in the overlapping part were shown to have a strong association with the gene signature (Figure 10C). Specifically, T cells CD4 memory-activated, T cells CD8, Macrophages M1, Dendritic cells resting, and T cells follicular helper were found to have positive correlations with the gene signature, and Monocytes and Mast cells resting showed negative correlations with it. 

**Figure 11:**
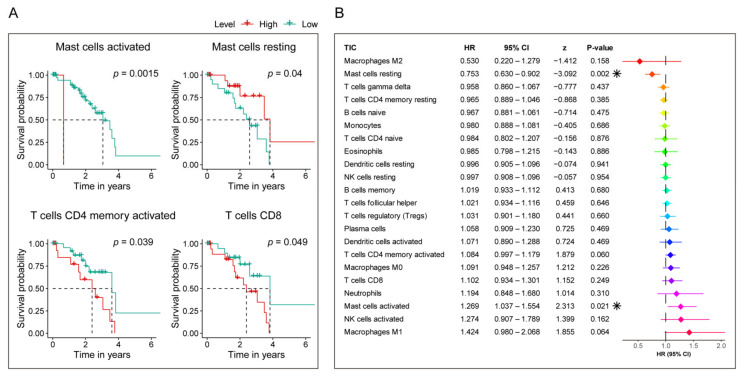
Evaluation of the prognostic ability of 22 TICs. (**A**) Kaplan–Meier survival curves of TICs that own prognosis value in UM. *p*-value < 0.05 in the log-rank test are set as the significant threshold. (**B**) Univariate Cox regression model built for 22 TICs based on overall survival. Asterix shown in the B plot indicate *p*-value is statistically significant.

Furthermore, to examine each TIC’s prognostic capacity, Kaplan–Meier and univariate Cox analyses were conducted. As shown in Figure 11, Kaplan–Meier analysis (Figure 11A and Table S8) indicated that Mast cells activated, T cells CD4 memory activated, Mast cells resting, and T cells CD8 can predict the survival of UM, while univariate Cox regression model (Figure 11B) highlighted that Mast cells resting and Mast cells activated impacted the prognosis. Based on the survival analysis listed above, we can see that Mast cells resting and Mast cells activated had a potential prognosis capacity in UM cases.

Taking together, the above findings revealed that Mast cells resting not only have a significant correlation with the risk score, but also have prognostic value in UM. Therefore, the significant infiltration with Mast cells resting may play a vital role in contributing to the prognostic value of the seven-gene signature in UM.

## 4. Discussion

In this study, we built a ferroptosis-related seven-gene signature for UM prognosis by comprehensively mining TCGA and GEO databases. After discovering the potential ferroptosis-related prognosis genes using Kaplan–Meier and univariate Cox analyses in the TCGA-UVM cohort, the LASSO Cox regression model was applied, and a seven-gene signature was generated which was related to the outcome of UM. By applying the seven-gene signature in training and validation cohorts, pronounced statistical differences were seen in Cox regression models, ROC curves, and Kaplan–Meier analysis between patients in terms of high- and low-risk score, demonstrating the effectiveness and broadness of the gene signature in predicting UM prognosis. The seven-gene signature was found in the following correlation analysis, correlated with the common mutations of UM and most autophagy-related genes. The GSEA and analysis of immune infiltration exhibited critical pathways that relate to the signature, as well as the vital role that Mast cells resting may play in backing the seven-gene signature influencing UM outcome. Compared with previous studies on UM’s prognostic gene signature, we are the first group to utilize ferroptosis-related genes for training and validated in an independent cohort. This work aimed to present future UM research with more information.

Ferroptosis is a new form of regulatory cell death, which is caused by the excessive accumulation of iron-dependent reactive oxygen species and lipid peroxides. It is characterized by increased mitochondrial membrane density and cell volume contraction, which is different from other morphological, biochemical, and genetically regulated cell death [7,44]. Recent research has shown that ferroptosis is closely associated with the pathophysiological process of many diseases, such as tumors, neurological disorders, ischemia-reperfusion injury, kidney injury, and blood diseases [7]. The fast-growing studies of ferroptosis in cancer have boosted the perspective of its usage in cancer therapeutics [45]. Ferroptosis is a newly introduced phenomenon in melanoma, and research is increasing to discover its role in melanoma [46]. Zhang and colleagues showed that the role of ferroptosis regulation by miR-9 in melanoma, and the knocking-down of miR-9, induce ferroptosis in melanoma cells [47]. miR-137 negatively affects necroptosis in melanoma cells and the inactivation of miR-137 enhances the antitumor activity of erastin by elevating ferroptosis [48]. The mitochondrial complex I inhibitor is a critical target in the induction of ferroptosis in melanoma cells [49]. A recent study performed by Ubellacker et al. exhibited that Oleic acid kept melanoma cells away from ferroptosis in an Acsl3-dependent manner and increased their ability to form metastatic tumors [13]. They also found that increased exposure to the lymphatic environment protects melanoma cells from ferroptosis and improves their capacity to survive during subsequent metastasis through the blood [13]. These pieces of evidence have highlighted the potential importance of ferroptosis in UM therapeutics, but the roles of ferroptosis in tumorigenesis and development remain unclear. Many studies have recently begun to mine the prognostic gene signature related to ferroptosis in tumors from public databases. For example, Liu confirmed that the ferroptosis-related nineteen-gene signature they discovered could predict glioma cell death and glioma patient progression [14]. Liang et al. found a novel ferroptosis-related gene signature, which can predict the prognosis of hepatocellular carcinoma [15]. However, there is still no explanation of whether a prognostic gene signature exists in UM. In order to fill this blank, we conducted in-depth research and discovered a ferroptosis-related seven-gene signature that is strongly linked to the prognosis of UM.

The ferroptosis-related seven-gene signature that we discovered showed strong prognostic prediction capabilities in the training cohort and the independent validation cohort after being examined by various statistical methods. Our signature was composed of seven genes, which were ALOX12, CD44, MAP1LC3C, STEAP3, HMOX1, ITGA6 and AIFM2/FSP1, respectively. In the signature model, STEAP3, HMOX1, ITGA6, and AIFM2/FSP1 were unfavorable genes for UM prognosis, while other genes showed a protective effect on the outcome. Lipid peroxidation plays a crucial role in ferroptosis execution. ALOX serves as one of the major enzymes for the oxygenation of arachidonic acid, an essential PUFA, finally triggering lipid peroxidation [38,50]. ACSL4 is a key protein in ferroptosis. ALOX12 was shown to be related to ferroptosis independently of ACSL4. Inactivation of ALOX12 can reduce p53-mediated ferroptosis, caused by active oxygen stress, and eliminate the dependence of p53 on tumor growth [38,50]. ALOX12 plays an important role in inflammation and oxidation, while abnormal DNA methylation and genetic variants of ALOX12 are associated with various human diseases and pathological phenotypes, including cancer [50]. MAP1LC3C is involved in the KEGG pathway of ferroptosis and was found to be downregulated in colorectal cancer [51]. AIFM2, also known as FSP1 or PRG3, has recently been demonstrated as an endogenous ferroptosis suppressor [42,43,52]. AIFM2/FSP1 blocks erastin-, sorafenib-, and RSL3-induced ferroptotic cancer cell death through a mechanism independent of ubiquinol, the reduced and active antioxidant form of coenzyme Q10 [42,43,52]. ITGA6 is a potential clinical prognostic marker for gallbladder carcinoma [53]. Public, database-based research revealed that ITGA6 expression is an independent prognostic factor of survival in breast cancer patients [54]. Besides this, Brooks et al. reported that ITGA6 is a hypoxia-inducible factor-dependent target gene, and high ITGA6 expression enhances invasion and tumor-initiating cell activities in models of metastatic breast cancer [54]. ALOX12 and MAP1LC3C, which promote ferroptosis [27,38,39,40,41], are downregulated in the high-risk group based on our findings; however, AIFM2/FSP1 and ITGA6, which inhibit ferroptosis, are upregulated in the high-risk population, suggesting that ferroptosis might be suppressed in patients with low prognosis. Cancer cells with stem cell and EMT properties are resistant to multiple therapies, leading to poor prognosis. Interestingly, these cancer cells are highly sensitive to ferroptosis [55,56,57]. CD44 expression suppressed ferroptosis in cancer cells in an OTUB1-dependent manner [37]. Notably, overexpression of the cancer-stem-cell-marker CD44 enhanced the stability of SLC7A11 by promoting the interaction between SLC7A11 and OTUB1; the depletion of CD44 partially abrogated this interaction [37]. A previous study reported that soluble CD44 inhibits the growth of melanoma tumors by blocking cell surface CD44 binding to hyaluronic acid [58]. CD44 is a well-known stem cell marker and is known to be activated in malignant tumors [59]. Our study found that CD44 has a protective effect on the prognosis of UM, which may not be directly administered by CD44 but is determined by genes that are upstream or downstream of CD44; there is still no research revealing the impact of CD44 on UM prognosis, which makes our findings interesting and worth further research. STEAP3 was reported to play a role in ferroptosis by mediating iron metabolism [7,45]. STEAP3 is a positive regulator of Myt1 and, together, STEAP3 and Myt1 cause a pronounced effect on the cell cycle, delaying the G2–M progression [60]. However, it belongs to an unfavorable factor in our study for UM prognosis. Since ferroptosis might not be activated in tumors, STEAP3 might affect poor prognosis independently of ferroptosis [45]. The role of HMOX1 is largely controversial. Previously, it was well known as an antioxidant enzyme, but several studies suggest that HMOX1 promotes ferroptosis [29,30,61]. HMOX1 is anti-cancer, anti-inflammatory, anti-apoptotic, anti-proliferative, and antioxidant [30]. The expression of HMOX1 is upregulated in different types of cancer, but its role in cancer or UM has not been elucidated [30]. 

Several studies have confirmed that chromosome aberrations and gene mutations in UM patients are very closely linked to prognosis [21,62,63,64,65,66,67,68,69]. The chromosome 3 loss (Monosomy 3) is associated with an increased metastasis possibility and bad outcomes [62]. Besides chromosome 3, the increased chromosome 8q and lack of 6p gain are found to be associated with poor prognosis [21,62,63,64,65,66,67,68,69]. Given the backgrounds listed above, the Pearson method was performed to examine the relationships between chromosome 3, 8q, and 6p and the signature. The results showed that the signature was negatively correlated with chromosome 3 and 6p, and positively correlated with 8q (Figure 6). These pieces of evidence further identified the importance of the signature in predicting UM outcomes.

Autophagy is the natural, regulated mechanism of the cell, which removes unnecessary or dysfunctional components. It allows the orderly degradation and recycling of cellular components [70]. The original study shows that ferroptosis is morphologically, biochemically, and genetically distinct from autophagy and other types of cell death [71]. However, recent studies demonstrate that the activation of ferroptosis is indeed dependent on the induction of autophagy [71]. Additionally, accumulating studies have revealed crosstalk between autophagy and ferroptosis at the molecular level [22]. Autophagy is a vital cellular process that maintains cellular homeostasis through the recycling of intracellular constituents. Previous studies on the role of autophagy in cancer have caused a debate as to whether autophagy is cancer-promoting or anti-cancer [72]. Autophagy is commonly upregulated in UMs and may be associated with hypoxia and intense pigmentation [73]. In the advanced stage of malignant tumors, melanoma cells hijack the autophagy mechanism to alleviate drug-induced and metabolic stress in the tumor microenvironment, thereby enhancing resistance to multiple therapies and tumor cell survival and progression [72]. In this research, we found that the risk score correlated with more than half of the autophagy-related genes (52.24%, 256/490), which elaborated the relation between the ferroptosis-related seven-gene signature and UM, and, moreover, introduced further capacity and more information for autophagy-targeted strategies.

The GSEA in HALLMARK collection found that gene sets regarding IL-6/JAK/STAT3 signaling, notch signaling, and glycolysis were most enriched. The IL-6/JAK/STAT3 pathway is aberrantly hyperactivated in many types of cancer, and such hyperactivation is generally associated with a poor clinical prognosis. In the tumor microenvironment, IL-6/JAK/STAT3 signaling acts to drive the proliferation, survival, invasiveness, and metastasis of tumor cells, while strongly suppressing the antitumor immune response [74]. IL-6 sends signals directly to melanoma cells through the JAK/STAT3 pathway, which leads to increased tumor production (immunosuppressive cytokines) [75]. Notch signaling denotes the Notch intracellular domain’s cleavage, its translocation to the nucleus, and subsequent activation of target gene transcription. The involvement of Notch signaling in several cancers is well known [76]. Notch signaling is a complex pathway that can regulate multiple aspects of the biology of melanoma and many other cancers. Not only can Notch signaling in melanoma cells interact with additional pathways involved in tumorigenesis, it can influence the fate of melanoma tumors through interaction with supporting stromal cells [77,78]. Changes in energy metabolism are the biochemical fingerprints of cancer cells and represent one of the “hallmarks of cancer”. This metabolic phenotype is characterized by preferentially relying on glycolysis to produce energy in an oxygen-independent manner [79,80]. These GSEA results gave a detailed description of the ways and methods by which the seven-gene signature participates in UM’s progress, which may benefit future precision medicine research.

Moreover, the CIBERSORT algorithm-based TICs analysis discovered that Mast cell resting has a strong prognostic capacity in UM, and a significant correlation with the ferroptosis-related seven-gene signature risk score, revealing that the activities of Mast cells resting may act as a key player affecting the seven-gene signature prognostic ability. Mast cells can be used as an essential innate immune sentinel. They can enhance the immune response mediated by T cells, but, in other cases, also show the ability to suppress the immune response [81]. Consistent with their functional plasticity, the number of mast cells in TME is associated with cancer progression and improved patient survival [81]. Mast cells are prototype innate cells that respond to various stimuli, including signals and components from the human microbiota, so they can be used as modulators of suppressive immune responses to initiate tumor immune control [82]. Mast cells are an essential source of CXCL10, and CXCL10 plays a vital role in melanoma’s immune defense. Thus, the mast cells are a promising potential choice for future melanoma treatment strategy development [82]. Based on our research, Mast cells resting can target the signature for the UM treatment means, and effort should be made to investigate these immune cells further.

## 5. Conclusions

Our study found a novel, robust ferroptosis-related seven-gene signature for UM. The signature is strongly associated with the prognosis of UM and can precisely detect UM risk level. Remarkably, we validated the reliability and applicability of this signature by applying it to an independent cohort and identified the vital role that Mast cells resting might interplay in the new gene signature’s prognostic capacity, which could advance the discovery of new treatments for UM.

## Figures and Tables

**Table 1:** Clinical characteristics of patients involved in the study.

Characteristics		Training Cohort (TCGA-UVM, 80 Cases)	Validation Cohort (GSE22138, 63 Cases)
age			
	<65	45(56.25%)	36(57.14%)
	≥65	35(43.75%)	27(42.86%)
gender			
	female	35(43.75%)	24(38.1%)
	male	45(56.25%)	39(61.9%)
T classification			
	T1	0	NA
	T2	4(5%)	NA
	T3	36(45%)	NA
	T4	38(47.5%)	NA
	unknown	2(2.5%)	NA
M classification			
	M0	73(91.25%)	28(44.44%)
	M1	3(3.75%)	35(55.56%)
	unknown	4(5%)	0
tumor stage			
	stage I	0	NA
	stage II	36(45%)	NA
	stage III	40(50%)	NA
	stage IV	4(5%)	NA
extrascleral extension			
	yes	7(8.75%)	5(7.94%)
	no	68(85%)	48(76.19%)
	unknown	5(6.25%)	10(15.87%)
tumor diameter, mm			
	<20	60(75%)	44(69.84%)
	≥20	19(23.75%)	9(14.29%)
	unknown	1(1.25%)	10(15.87%)
tumor thickness, mm			
	<10	29(36.25%)	10(15.87%)
	≥10	51(63.75%)	53(84.13%)
tumor eye side			
	left	NA	33(52.38%)
	right	NA	30(47.62%)
tumor location			
	all over the eye	NA	1(1.59%)
	anterior to equator	NA	3(4.76%)
	on equator	NA	42(66.67%)
	posterior and on equator	NA	3(4.76%)
	posterior to equator	NA	9(14.29%)
	unknown	NA	5(7.94%)
tumor cell type			
	epithelioid	NA	21(33.33%)
	mixed	NA	23(36.51%)
	unknown	NA	19(30.16%)
eye color			
	blue	9(11.25%)	NA
	brown	15(18.75%)	NA
	green	6(7.5%)	NA
	unknown	50(62.5%)	NA
person neoplasm cancer status			
	with tumor	9(11.25%)	NA
	tumor free	56(70%)	NA
	unknown	15(18.75%)	NA
radiation therapy			
	yes	3(3.75%)	NA
	no	63(78.75%)	NA
	unknown	14(17.5%)	NA
ethnicity			
	hispanic or latino	1(1.25%)	NA
	not hispanic or latino	52(65%)	NA
	unknown	27(33.75%)	NA
tissue or organ of origin diagnosis			
	choroid	67(83.75%)	NA
	ciliary body	5(6.25%)	NA
	overlapping lesion of eye and adnexa	8(10%)	NA
retinal detachment			
	yes	NA	36(57.14%)
	no	NA	22(34.92%)
	unknown	NA	5(7.94%)
mitotic count			
	<20	42(52.5%)	NA
	≥20	11(13.75%)	NA
	unknown	27(33.75%)	NA
chromosome 3 status			
	disomy	NA	18(28.57%)
	monosomy	NA	37(58.73%)
	unknown	NA	8(12.7%)

TCGA: The Cancer Genome Atlas; TCGA-UVM: A project ID in The Cancer Genome Atlas database; NA: data not available.

**Table 2:** 22 ferroptosis-related potential prognostic genes generated from the training cohort.

Gene Symbol	Description	Category	Genomic Location	Kaplan–Meier Analysis (*p*-Value)	Univariate Cox Regression Analysis			
					HR	HR_95L	HR_95H	*p*-Value
VDAC1	Voltage Dependent Anion Channel 1	Protein Coding	chr5	7.12 × 10^−6^	5.291343594	1.781142996	15.71929771	2.71 × 10^−3^
STEAP3	STEAP3 Metalloreductase	Protein Coding	chr2	3.19 × 10^−3^	4.206162616	2.092921898	8.453160136	5.49 × 10^−5^
SLC39A8	Solute Carrier Family 39 Member 8	Protein Coding	chr4	3.77 × 10^−2^	3.869852511	1.326333156	11.29109861	1.33 × 10^−2^
SLC11A2	Solute Carrier Family 11 Member 2	Protein Coding	chr12	4.76 × 10^−3^	3.094601274	1.570769273	6.096730571	1.09 × 10^−3^
PEBP1	Phosphatidylethanolamine Binding Protein 1	Protein Coding	chr12	4.86 × 10^−2^	0.234529606	0.075635069	0.727230595	1.20 × 10^−2^
MAPK1	Mitogen−Activated Protein Kinase 1	Protein Coding	chr22	9.03 × 10^−3^	2.895607401	1.163501743	7.206299663	2.23 × 10^−2^
MAP1LC3C	Microtubule Associated Protein 1 Light Chain 3 Gamma	Protein Coding	chr1	1.31 × 10^−2^	0.459748503	0.271733125	0.777853955	3.78 × 10^−3^
LINC00472	Long Intergenic Non-Protein Coding RNA 472	RNA Gene	chr6	6.36 × 10^−3^	0.043258919	0.003322917	0.563160037	1.65 × 10^−2^
ITGA6	Integrin Subunit Alpha 6	Protein Coding	chr2	1.13 × 10^−3^	4.613594536	2.148024539	9.909223176	8.85 × 10^−5^
HSPA5	Heat Shock Protein Family A (Hsp70) Member 5	Protein Coding	chr9	8.91 × 10^−3^	2.25326069	1.228917946	4.131426149	8.63 × 10^−3^
HMOX1	Heme Oxygenase 1	Protein Coding	chr22	2.19 × 10^−3^	2.334473768	1.59857086	3.409149952	1.14 × 10^−5^
GSS	Glutathione Synthetase	Protein Coding	chr20	3.31 × 10^−3^	3.851728269	1.85357871	8.003874117	3.02 × 10^−4^
FTH1	Ferritin Heavy Chain 1	Protein Coding	chr11	4.64 × 10^− 3^	4.040699109	1.10619952	14.75976891	3.46 × 10^−2^
CD44	CD44 Molecule (Indian Blood Group)	Protein Coding	chr11	6.66 × 10^− 3^	0.304760194	0.142947413	0.649740862	2.10 × 10^−3^
CASP8	Caspase 8	Protein Coding	chr2	3.91 × 10^−2^	2.604965341	1.209052632	5.612530215	1.45 × 10^−2^
BAP1	BRCA1 Associated Protein 1	Protein Coding	chr3	1.40 × 10^−6^	0.561778701	0.412394493	0.765275275	2.56 × 10^−4^
AURKA	Aurora Kinase A	Protein Coding	chr20	2.54 × 10^−2^	3.390492663	1.565174215	7.344511806	1.96 × 10^−3^
ANO6	Anoctamin 6	Protein Coding	chr12	2.56 × 10^−2^	2.263254914	1.292723147	3.962428319	4.26 × 10^−3^
ALOX12	Arachidonate 12-Lipoxygenase, 12S Type	Protein Coding	chr17	1.48 × 10^−3^	0.022909689	0.002539428	0.20668188	7.66 × 10^−4^
AIFM2/FSP1	Apoptosis Inducing Factor Mitochondria Associated 2	Protein Coding	chr10	6.03 × 10^−6^	6.104896507	2.780109603	13.40586045	6.55 × 10^−6^
ACSL6	Acyl-CoA Synthetase Long Chain Family Member 6	Protein Coding	chr5	4.56 × 10^−4^	2.283441779	1.099533069	4.742109633	2.68 × 10^−2^
ACSL1	Acyl-CoA Synthetase Long Chain Family Member 1	Protein Coding	chr4	6.64 × 10^−3^	1.873686292	1.236619189	2.838950221	3.06 × 10^−3^

**Table 3:** 7 ferroptosis-related prognostic genes obtained from LASSO Cox regression model.

Gene Symbol	Description	Role	Risk Coefficient
STEAP3	STEAP3 Metalloreductase	Marker [26]	0.055060532
MAP1LC3C	Microtubule Associated Protein 1 Light Chain 3 Gamma	Driver [27]	−0.202884346
ITGA6	Integrin Subunit Alpha 6	Suppressor [28]	0.34461317
HMOX1	Heme Oxygenase 1	Driver [29,30,31,32], Suppressor [33,34], Marker [35,36]	0.125266141
CD44	CD44 Molecule (Indian Blood Group)	Suppressor [37]	−0.316011897
ALOX12	Arachidonate 12-Lipoxygenase, 12S Type	Driver [38,39,40], Marker [41]	−1.311120914
AIFM2/FSP1	Apoptosis Inducing Factor Mitochondria Associated 2	Suppressor [42,43]	0.710789029

**Table 4:** Univariate analysis and multivariate analysis of the correlation of gene-signature risk score with outcomes among uveal melanoma patients in two cohorts.

Variable	Univariate Cox Analysis				Multivariate Cox Analysis			
	Coef	HR (95% CI)	z	*p*-Value	Coef	HR (95% CI)	z	*p*-Value
TCGA-UVM (overall survival) *								
age	0.0447	1.05 (1.01–1.09)	2.35	**0.0186**	0.101	1.11 (0.976–1.25)	1.57	0.115
gender (male vs. female)	0.433	1.54 (0.651–3.65)	0.984	0.325				
tumor stage (stage III vs. stage II)	0.336	1.4 (0.556–3.52)	0.713	0.476	−3.06	0.047 (0.00199–1.11)	−1.9	0.0579
tumor stage (stage IV vs. stage II)	4.37	79.3 (7.55–834)	3.64	**0.000269**	NA	NA	NA	NA
extrascleral extension (yes vs. no)	1.54	4.64 (1.5–14.4)	2.66	**0.00774**	−4.25	0.0142 (3.98× 10^−13^–5.1× 10 ^8^)	−0.343	0.732
tumor diameter	0.155	1.17 (1.01–1.35)	2.12	**0.0344**	0.723	2.06 (1.11–3.83)	2.29	**0.0221**
tumor thickness	0.111	1.12 (0.949–1.32)	1.33	0.183				
radiation therapy (yes vs. no)	1.68	5.35 (1.09–26.3)	2.07	**0.0389**	7.79	2410 (4.34 × 10^−8^–1.33 × 10 ^14^)	0.617	0.537
ethnicity (hispanic or latino vs. not hispanic or latino)	−16	1.09 × 10^−7^ (0-Inf)	−0.00205	0.998				
tissue or organ of origin diagnosis (choroid vs. not choroid)	−0.286	0.751 (0.254–2.22)	−0.517	0.605				
mitotic count	−0.0119	0.988 (0.931–1.05)	−0.394	0.693				
chromosome 3 copy number	−1.86	0.156 (0.0574–0.422)	−3.65	**0.00026**	2.76	15.9 (0.894–281)	1.88	0.0597
chromosome 6p copy number	−1.06	0.348 (0.176–0.687)	−3.04	**0.00237**	−0.874	0.417 (0.0559–3.11)	−0.852	0.394
chromosome 8q copy number	0.516	1.67 (1.27–2.2)	3.68	**0.000235**	−0.88	0.415 (0.126–1.37)	−1.44	0.149
risk score	1.65	5.22 (2.59–10.5)	4.61	**3.99 × 10^−6^**	4.23	68.6 (3.36–1400)	2.75	**0.00598**
**TCGA-UVM (progression-free survival) ^#^**								
age	0.0271	1.03 (0.996–1.06)	1.7	0.0886				
gender (male vs. female)	−0.139	0.87 (0.422–1.8)	−0.376	0.707				
tumor stage (stage III vs. stage II)	0.381	1.46 (0.665–3.22)	0.946	0.344	−0.959	0.383 (0.094–1.56)	−1.34	0.181
tumor stage (stage IV vs. stage II)	3.31	27.4 (5.06–149)	3.84	**0.000124**	2.87	17.6 (0.677–455)	1.73	0.0845
extrascleral extension (yes vs. no)	1.45	4.26 (1.57–11.5)	2.85	**0.0044**	−0.682	0.506 (0.00917–27.9)	−0.333	0.739
tumor diameter	0.113	1.12 (0.999–1.25)	1.94	0.0527				
tumor thickness	0.00859	1.01 (0.875–1.16)	0.119	0.905				
radiation therapy (yes vs. no)	0.0846	1.09 (0.142–8.33)	0.0814	0.935				
ethnicity (hispanic or latino vs. not hispanic or latino)	−17	3.98 × 10^−8^ (0-Inf)	−0.00217	0.998				
tissue or organ of origin diagnosis (choroid vs. not choroid)	0.0872	1.09 (0.377–3.16)	0.161	0.872				
mitotic count	0.0545	1.06 (1.01–1.1)	2.64	**0.00829**	0.0575	1.06 (0.976–1.15)	1.37	0.17
chromosome 3 copy number	−1.86	0.156 (0.0631–0.386)	−4.02	**5.88 × 10^−5^**	−1.3	0.272 (0.0293–2.52)	−1.15	0.252
chromosome 6p copy number	−0.628	0.534 (0.321–0.888)	−2.42	**0.0155**	0.709	2.03 (0.611–6.75)	1.16	0.247
chromosome 8q copy number	0.521	1.68 (1.31–2.16)	4.08	**4.56 × 10^−5^**	0.146	1.16 (0.546–2.45)	0.381	0.703
risk score	0.933	2.54 (1.65–3.91)	4.25	**2.13 × 10^−5^**	0.703	2.02 (0.486–8.39)	0.967	**0.0334**
**GSE22138 (metastasis-free survival) ^&^**								
age	0.0213	1.02 (0.995–1.05)	1.59	0.113				
gender (male vs. female)	0.353	1.42 (0.714–2.84)	1	0.316				
tumor eye side (left vs. right)	−0.193	0.824 (0.424–1.6)	−0.57	0.569				
tumor location (on equator vs. others)	−0.325	0.723 (0.357–1.46)	−0.903	0.366				
tumor diameter	−0.0165	0.984 (0.893–1.08)	−0.336	0.737				
tumor thickness	0.116	1.12 (0.951–1.33)	1.37	0.171				
tumor cell type (epithelioid vs. mixed)	0.753	2.12 (0.954–4.72)	1.85	0.0649				
retinal detachment (yes vs. no)	1.06	2.87 (1.24–6.68)	2.45	**0.0142**	0.857	2.36 (0.981–5.66)	1.92	0.0553
extrascleral extension (yes vs. no)	0.563	1.76 (0.668–4.62)	1.14	0.253				
chromosome 3 status (monosomy vs. disomy)	1.67	5.29 (1.82–15.3)	3.07	**0.00217**	1.24	3.45 (1.04–11.5)	2.02	**0.0435**
risk score	0.646	1.91 (1.31–2.78)	3.37	**0.000745**	0.523	1.69 (1.03–2.77)	2.07	**0.0388**

* Multivariate Cox analysis: Concordance = 0.936 (se = 0.049), Likelihood ratio test = 38.13 on 9 df, *p* = 2 × 10^−5^, Wald test = 11.72 on 9 df, *p* = 0.2, Score (logrank) test = 29.7 on 9 df, *p* = 5 × 10^−4^; ^#^ Multivariate Cox analysis: Concordance = 0.815 (se = 0.067), Likelihood ratio test = 24.64 on 8 df, *p* = 0.002, Wald test = 19.68 on 8 df, *p* = 0.01, Score (logrank) test = 47.01 on 8 df, *p* = 2 × 10^−7^; ^&^ Multivariate Cox analysis: Concordance = 0.764 (se = 0.05), Likelihood ratio test = 20.65 on 3 df, *p* = 1 × 10^−4^, Wald test = 17.24 on 3 df, *p* = 6 × 10^−4^, Score (logrank) test = 19.56 on 3 df, *p* = 2 × 10^−4^; The bold *p*-value indicates statistical significance.

## Data Availability

The following publicly available datasets were used in this study: TCGA: https://portal.gdc.cancer.gov/projects/TCGA-UVM; GEO: https://www.ncbi.nlm.nih.gov/geo/query/acc.cgi?acc=GSE22138.

## Supplementary Materials

The following are available online at https://www.mdpi.com/2075-4418/11/2/219/s1, Table S1: Table S1. 103 ferroptosis-related genes retrieved from the GeneCards; Table S2. The status of chromosome copy number aberrations of TCGA-UVM cases; Table S3. Autophagy-related genes; Table S4. 7025 genes were significantly predicting the prognosis of UM patients by both Kaplan-Meier and univariate Cox regression analyses (*p*-value < 0.05); Table S5. The correlations of 490 autophagy-related genes with the seven-gene signature risk score examined by the Pearson correlation coefficient; Table S6. Enriched gene sets in HALLMARK collection (|NES|> 1, NOM *p*-val < 0.05, and FDR *q*-val < 0.25); Table S7. Correlations of risk score with 22 kinds of TICs tested by Spearman coefficient; Table S8. Prognostic capacity of 22 TICs examined by Kaplan–Meier analysis.
