# Supplementary material for: A Novel Ferroptosis-Associated Gene Signature to Predict Prognosis in Patients with Uveal Melanoma
Source: Diagnostics (Basel). 2021 Feb 2;11(2):219. doi: 10.3390/diagnostics11020219 (PMC7913108; doi:10.3390/diagnostics11020219)
Supplement: Supplementary file 1 [file diagnostics-11-00219-s001.pdf]

Table S1. 103 ferroptosis-related genes retrieved from the GeneCards.

Gene Symbol	Description	Category
GPX4	Glutathione Peroxidase 4	Protein Coding
AIFM2	Apoptosis Inducing Factor Mitochondria Associated 2	Protein Coding
TP53	Tumor Protein P53	Protein Coding
ACSL4	Acyl-CoA Synthetase Long Chain Family Member 4	Protein Coding
SLC7A11	Solute Carrier Family 7 Member 11	Protein Coding
VDAC2	Voltage Dependent Anion Channel 2	Protein Coding
VDAC3	Voltage Dependent Anion Channel 3	Protein Coding
ATG5	Autophagy Related 5	Protein Coding
ATG7	Autophagy Related 7	Protein Coding
NCOA4	Nuclear Receptor Coactivator 4	Protein Coding
HMOX1	Heme Oxygenase 1	Protein Coding
SLC3A2	Solute Carrier Family 3 Member 2	Protein Coding
ALOX15	Arachidonate 15-Lipoxygenase	Protein Coding
BECN1	Beclin 1	Protein Coding
PRKAA1	Protein Kinase AMP-Activated Catalytic Subunit Alpha 1	Protein Coding
SAT1	Spermidine/Spermine N1-Acetyltransferase 1	Protein Coding
NF2	Neurofibromin 2	Protein Coding
YAP1	Yes1 Associated Transcriptional Regulator	Protein Coding
FTH1	Ferritin Heavy Chain 1	Protein Coding
TF	Transferrin	Protein Coding
TFRC	Transferrin Receptor	Protein Coding
FTL	Ferritin Light Chain	Protein Coding
CYBB	Cytochrome B-245 Beta Chain	Protein Coding
GSS	Glutathione Synthetase	Protein Coding
CP	Ceruloplasmin	Protein Coding
PRNP	Prion Protein	Protein Coding
SLC11A2	Solute Carrier Family 11 Member 2	Protein Coding
SLC40A1	Solute Carrier Family 40 Member 1	Protein Coding
STEAP3	STEAP3 Metalloreductase	Protein Coding
ACSL1	Acyl-CoA Synthetase Long Chain Family Member 1	Protein Coding
GCLC	Glutamate-Cysteine Ligase Catalytic Subunit	Protein Coding
MAP1LC3A	Microtubule Associated Protein 1 Light Chain 3 Alpha	Protein Coding
MAP1LC3B	Microtubule Associated Protein 1 Light Chain 3 Beta	Protein Coding
SLC39A14	Solute Carrier Family 39 Member 14	Protein Coding
SLC39A8	Solute Carrier Family 39 Member 8	Protein Coding
ACSL5	Acyl-CoA Synthetase Long Chain Family Member 5	Protein Coding
GCLM	Glutamate-Cysteine Ligase Modifier Subunit	Protein Coding
PCBP1	Poly(RC) Binding Protein 1	Protein Coding
PCBP2	Poly(RC) Binding Protein 2	Protein Coding
ACSL3	Acyl-CoA Synthetase Long Chain Family Member 3	Protein Coding
ACSL6	Acyl-CoA Synthetase Long Chain Family Member 6	Protein Coding
SAT2	Spermidine/Spermine N1-Acetyltransferase Family Member 2	Protein Coding
FTMT	Ferritin Mitochondrial	Protein Coding
LPCAT3	Lysophosphatidylcholine Acyltransferase 3	Protein Coding
MAP1LC3C	Microtubule Associated Protein 1 Light Chain 3 Gamma	Protein Coding
MAP1LC3B2	Microtubule Associated Protein 1 Light Chain 3 Beta 2	Protein Coding
BAP1	BRCA1 Associated Protein 1	Protein Coding

PRDX6	Peroxiredoxin 6	Protein Coding
SESN2	Sestrin 2	Protein Coding
ARNTL	Aryl Hydrocarbon Receptor Nuclear Translocator Like	Protein Coding
CISD1	CDGSH Iron Sulfur Domain 1	Protein Coding
PROM2	Prominin 2	Protein Coding
NEDD4	NEDD4 E3 Ubiquitin Protein Ligase	Protein Coding
CA9	Carbonic Anhydrase 9	Protein Coding
ELAVL1	ELAV Like RNA Binding Protein 1	Protein Coding
NFE2L2	Nuclear Factor, Erythroid 2 Like 2	Protein Coding
ITGA6	Integrin Subunit Alpha 6	Protein Coding
PRKAA2	Protein Kinase AMP-Activated Catalytic Subunit Alpha 2	Protein Coding
FANCD2	FA Complementation Group D2	Protein Coding
LAMP2	Lysosomal Associated Membrane Protein 2	Protein Coding
ALOX12	Arachidonate 12-Lipoxygenase, 12S Type	Protein Coding
CD44	CD44 Molecule (Indian Blood Group)	Protein Coding
MAPK1	Mitogen-Activated Protein Kinase 1	Protein Coding
MYC	MYC Proto-Oncogene, BHLH Transcription Factor	Protein Coding
EGLN1	Egl-9 Family Hypoxia Inducible Factor 1	Protein Coding
GOT1	Glutamic-Oxaloacetic Transaminase 1	Protein Coding
MAP3K5	Mitogen-Activated Protein Kinase Kinase Kinase 5	Protein Coding
ATF4	Activating Transcription Factor 4	Protein Coding
FH	Fumarate Hydratase	Protein Coding
HELLS	Helicase, Lymphoid Specific	Protein Coding
SOCS1	Suppressor Of Cytokine Signaling 1	Protein Coding
OTUB1	OTU Deubiquitinase, Ubiquitin Aldehyde Binding 1	Protein Coding
CARS1	Cysteinyl-TRNA Synthetase 1	Protein Coding
MIR9-1	MicroRNA 9-1	RNA Gene
MIR137	MicroRNA 137	RNA Gene
HMGB1	High Mobility Group Box 1	Protein Coding
LINC00336	Long Intergenic Non-Protein Coding RNA 336	RNA Gene
NFS1	NFS1 Cysteine Desulfurase	Protein Coding
PEBP1	Phosphatidylethanolamine Binding Protein 1	Protein Coding
RB1	RB Transcriptional Corepressor 1	Protein Coding
G3BP1	G3BP Stress Granule Assembly Factor 1	Protein Coding
LINC00472	Long Intergenic Non-Protein Coding RNA 472	RNA Gene
EPAS1	Endothelial PAS Domain Protein 1	Protein Coding
HILPDA	Hypoxia Inducible Lipid Droplet Associated	Protein Coding
PRC1	Protein Regulator Of Cytokinesis 1	Protein Coding
NGB	Neuroglobin	Protein Coding
MDM2	MDM2 Proto-Oncogene	Protein Coding
TIGAR	TP53 Induced Glycolysis Regulatory Phosphatase	Protein Coding
VDAC1	Voltage Dependent Anion Channel 1	Protein Coding
HSPB1	Heat Shock Protein Family B (Small) Member 1	Protein Coding
HSPA5	Heat Shock Protein Family A (Hsp70) Member 5	Protein Coding
CDKN2A	Cyclin Dependent Kinase Inhibitor 2A	Protein Coding
CASP8	Caspase 8	Protein Coding
CFTR	CF Transmembrane Conductance Regulator	Protein Coding
AURKA	Aurora Kinase A	Protein Coding
MIF	Macrophage Migration Inhibitory Factor	Protein Coding

RIPK1	Receptor Interacting Serine/Threonine Kinase 1	Protein Coding
MUC1	Mucin 1, Cell Surface Associated	Protein Coding
ALOX15B	Arachidonate 15-Lipoxygenase Type B	Protein Coding
ANO6	Anoctamin 6	Protein Coding
GUCY1A1	Guanylate Cyclase 1 Soluble Subunit Alpha 1	Protein Coding
MT1G	Metallothionein 1G	Protein Coding
MIR7-1	MicroRNA 7-1	RNA Gene

GeneCards: https://www.genecards.org/

Table S2. The status of chromosome copy number aberrations of TCGA-UVM cases.

Patient ID	3 CN (ABSOLUTE)	8q CN (ABSOLUTE)	6p CN (ABSOLUTE)
TCGA-V3-A9ZY	2	2	3
TCGA-V4-A9EC	2	2	2
TCGA-V4-A9EH	2	2	2
TCGA-V4-A9EY	2	2	3 (less than half)
TCGA-V4-A9F7	2	2	3
TCGA-VD-A8KE	2	2	3
TCGA-VD-A8KO	2	2	3
TCGA-VD-AA8M	2	2	2
TCGA-VD-AA8Q	2	2	2
TCGA-VD-AA8R	2	2	2
TCGA-WC-A87T	2	2	3 (less than half)
TCGA-WC-A87U	2	2	3
TCGA-WC-A880	2	2	3
TCGA-WC-A884	2	2	3
TCGA-YZ-A983	2	2	3
TCGA-V4-A9E5	2	3	4
TCGA-V4-A9E9	2	3	3
TCGA-V4-A9EA	2	3	3
TCGA-V4-A9EJ	2	3	4
TCGA-V4-A9EK	2	3	4
TCGA-V4-A9EM	2	4	3
TCGA-V4-A9ET	2	4	4
TCGA-V4-A9EW	2	2	4
TCGA-V4-A9EZ	2	3	3
TCGA-V4-A9F2	2	3	3
TCGA-V4-A9F4	2	3	4
TCGA-VD-A8K7	2	2	4
TCGA-VD-A8K9	2	3	5
TCGA-VD-A8KA	2	3	4
TCGA-VD-A8KB	2	4	4
TCGA-VD-A8KG	2	4	4
TCGA-VD-A8KJ	2	4	3 and 4 (roughly half)
TCGA-VD-AA8S	2	3	4
TCGA-WC-A87W	2	2	3
TCGA-WC-A881	2	5	3
TCGA-WC-A885	2	3	4
TCGA-WC-AA9E	2	3	5
TCGA-YZ-A982	3	4	5
TCGA-V4-A9E8	1	3	3
TCGA-V4-A9ED	1	2	2
TCGA-V4-A9EF	1	4	2
TCGA-V4-A9EO	2, LOH	5	4
TCGA-V4-A9EQ	1	3	2
TCGA-V4-A9ES	1	2	2
TCGA-V4-A9F0	1	3	2
TCGA-V4-A9F8	1	4	2
TCGA-VD-A8KD	1	3	2

TCGA-VD-A8KF	1	3	2
TCGA-VD-A8KH	1	3	2
TCGA-VD-A8KK	1	3	2
TCGA-VD-A8KL	2, LOH	5	4
TCGA-VD-AA8O	2, LOH	4	3
TCGA-VD-AA8P	1	3	2
TCGA-VD-AA8T	1	3	2
TCGA-WC-A882	1	3	2
TCGA-WC-A888	2, Subclonal LOH	3	2
TCGA-WC-AA9A	1	3	2
TCGA-YZ-A980	1	3	2
TCGA-YZ-A984	2, LOH	4	3
TCGA-YZ-A985	1	2	2
TCGA-RZ-AB0B	1	7	2
TCGA-V3-A9ZX	1	7	3
TCGA-V4-A9E7	1	5	2
TCGA-V4-A9EE	1	5	2
TCGA-V4-A9EI	1	8	3
TCGA-V4-A9EL	1	7	2
TCGA-V4-A9EU	1	6	2
TCGA-V4-A9EV	1	5	2
TCGA-V4-A9EX	1	6	3
TCGA-V4-A9F1	1	5	2
TCGA-V4-A9F3	1	5	3
TCGA-V4-A9F5	1	4	2
TCGA-VD-A8K8	1	5	2
TCGA-VD-A8KI	1	5	3
TCGA-VD-A8KM	1	3	3
TCGA-VD-A8KN	1	4	2
TCGA-VD-AA8N	1	4	2
TCGA-WC-A87Y	1	4	2
TCGA-WC-A883	1	6	2
TCGA-WC-A88A	1	4	3

Table S3. Autophagy-related genes.

No.	Gene Symbol	No.	Gene Symbol	No.	Gene Symbol	No.	Gene Symbol	No.	Gene Symbol
1	ABL1	101	CSNK2A2	201	HTT	301	OSBPL7	401	SOGA3
2	ABL2	102	CTSA	202	IFI16	302	P4HB	402	SPHK1
3	ACER2	103	CTSB	203	IFNG	303	PAFAH1B2	403	SPNS1
4	ADRA1A	104	CTSD	204	IFT20	304	PARK2	404	SPTLC1
5	ADRB2	105	CTTN	205	IFT88	305	PARK7	405	SPTLC2
6	AKT1	106	CX3CL1	206	IKBKB	306	PARP1	406	SQSTM1
7	AMBRA1	107	CXCR4	207	IKBKE	307	PEA15	407	SREBF1
8	APOL1	108	DAP	208	IKBKG	308	PELP1	408	SREBF2
9	ARNT	109	DAPK1	209	IL10	309	PEX14	409	ST13
10	ARSA	110	DAPK2	210	IL10RA	310	PEX3	410	STAT3
11	ARSB	111	DAPK3	211	IL24	311	PHF23	411	STK11
12	ATF4	112	DAPL1	212	IL4	312	PIK3C3	412	STUB1
13	ATF6	113	DCN	213	IRGM	313	PIK3CA	413	STX12
14	ATG10	114	DDIT3	214	ITGA3	314	PIK3CB	414	SUPT5H
15	ATG101	115	DHRSX	215	ITGA6	315	PIK3R2	415	SVIP
16	ATG12	116	DIRAS3	216	ITGB1	316	PIK3R4	416	SYNPO2
17	ATG13	117	DLC1	217	ITGB4	317	PIKFYVE	417	TAB2
18	ATG14	118	DNAJB1	218	ITPR1	318	PIM2	418	TAB3
19	ATG16L1	119	DNAJB9	219	KAT5	319	PINK1	419	TBC1D12
20	ATG16L2	120	DNM1L	220	KAT8	320	PIP4K2A	420	TBC1D14
21	ATG2A	121	DRAM1	221	KDM4A	321	PIP4K2B	421	TBC1D25
22	ATG2B	122	DRAM2	222	KDR	322	PIP4K2C	422	TBK1
23	ATG3	123	EDEM1	223	KEAP1	323	PLEKHF1	423	TEX264
24	ATG4A	124	EEF1A1	224	KIAA0226	324	PLK2	424	TFEB
25	ATG4B	125	EEF1A2	225	KIAA1324	325	PLK3	425	TICAM1
26	ATG4C	126	EEF2	226	KIF25	326	POLDIP2	426	TLK2
27	ATG4D	127	EEF2K	227	KIF5B	327	PPP1R15A	427	TM9SF1
28	ATG5	128	EGFR	228	KLHL22	328	PRKAA1	428	TMEM150A
29	ATG7	129	EIF2AK2	229	KLHL24	329	PRKAA2	429	TMEM150B
30	ATG9A	130	EIF2AK3	230	KLHL3	330	PRKAB1	430	TMEM150C
31	ATG9B	131	EIF2AK4	231	LACRT	331	PRKAB2	431	TMEM59

32	ATIC	132	EIF2S1	232	LAMP1	332	PRKACA	432	TMEM74
33	ATM	133	EIF4EBP1	233	LAMP2	333	PRKAG1	433	TNFSF10
34	ATP13A2	134	EIF4G1	234	LAMP3	334	PRKAG2	434	TOMM7
35	ATP6V0A1	135	EIF4G2	235	LAMTOR1	335	PRKAG3	435	TP53
36	ATP6V0A2	136	EP300	236	LAMTOR2	336	PRKAR1A	436	TP53INP1
37	ATP6V0B	137	EPM2A	237	LAMTOR3	337	PRKCD	437	TP53INP2
38	ATP6V0C	138	ERBB2	238	LAMTOR4	338	PRKCQ	438	TP63
39	ATP6V0D1	139	ERCC4	239	LAMTOR5	339	PRKD1	439	TP73
40	ATP6V0D2	140	ERN1	240	LARP1	340	PSAP	440	TPCN1
41	ATP6V0E1	141	ERO1L	241	LEP	341	PTEN	441	TPCN2
42	ATP6V0E2	142	EXOC1	242	LEPR	342	PTK2	442	TRAPPC8
43	ATP6V1A	143	EXOC4	243	LGALS8	343	PTK6	443	TREM2
44	ATP6V1B1	144	EXOC7	244	LRRK2	344	PTPN22	444	TRIB3
45	ATP6V1B2	145	EXOC8	245	LRSAM1	345	PYCARD	445	TRIM13
46	ATP6V1C1	146	FADD	246	LZTS1	346	QSOX1	446	TRIM21
47	ATP6V1C2	147	FAS	247	MAP1LC3A	347	RAB11A	447	TRIM22
48	ATP6V1D	148	FBXL2	248	MAP1LC3B	348	RAB12	448	TRIM65
49	ATP6V1E1	149	FBXO7	249	MAP1LC3C	349	RAB1A	449	TRIM8
50	ATP6V1E2	150	FBXW7	250	MAP2K7	350	RAB1B	450	TSC1
51	ATP6V1G1	151	FEZ1	251	MAP3K7	351	RAB24	451	TSC2
52	ATP6V1G2	152	FEZ2	252	MAPK1	352	RAB33B	452	TSPO
53	ATP6V1H	153	FKBP1A	253	MAPK15	353	RAB39B	453	TUSC1
54	BAD	154	FKBP1B	254	MAPK3	354	RAB3GAP1	454	UBQLN1
55	BAG1	155	FLCN	255	MAPK8	355	RAB3GAP2	455	UBQLN2
56	BAG3	156	FOS	256	MAPK8IP1	356	RAB5A	456	UBQLN4
57	BAK1	157	FOXK1	257	MAPK9	357	RAB7A	457	UCHL1
58	BAX	158	FOXK2	258	MAPT	358	RAB8A	458	ULK1
59	BCL2	159	FOXO1	259	MBTPS2	359	RAC1	459	ULK2
60	BCL2L1	160	FOXO3	260	MCL1	360	RAF1	460	ULK3
61	BCL2L11	161	FTH1	261	MEFV	361	RALB	461	USP10
62	BECN1	162	FTL	262	MET	362	RASIP1	462	USP13
63	BID	163	FYCO1	263	MFN2	363	RB1	463	USP30
64	BIRC5	164	FZD5	264	MFSD8	364	RB1CC1	464	USP33

65	BIRC6	165	GAA	265	MID2	365	RELA	465	UVRAG
66	BMF	166	GABARAP	266	MIR199A1	366	RGS19	466	VAMP3
67	BNIP1	167	GABARAPL1	267	MIR199A2	367	RHEB	467	VAMP7
68	BNIP3	168	GABARAPL2	268	MLST8	368	RIPK2	468	VDAC1
69	BNIP3L	169	GAPDH	269	MT3	369	RNF152	469	VEGFA
70	BOK	170	GATA4	270	MTCL1	370	RNF41	470	VMP1
71	C9orf72	171	GBA	271	MTDH	371	RNF5	471	VPS13C
72	CALCOCO2	172	GFAP	272	MTM1	372	ROCK1	472	VPS13D
73	CAMKK2	173	GNAI3	273	MTMR14	373	RPS6KB1	473	VPS26A
74	CANX	174	GNB2L1	274	MTMR3	374	RPTOR	474	VPS26B
75	CAPN1	175	GOLGA2	275	MTMR8	375	RRAGA	475	VPS35
76	CAPN10	176	GOPC	276	MTMR9	376	RRAGB	476	WAC
77	CAPN2	177	GPSM1	277	MTOR	377	RRAGC	477	WDFY3
78	CAPNS1	178	GRID1	278	MUL1	378	RRAGD	478	WDR24
79	CASP1	179	GRID2	279	MYC	379	RUFY4	479	WDR41
80	CASP3	180	GSK3A	280	NAF1	380	SAR1A	480	WDR45
81	CASP4	181	GSK3B	281	NAMPT	381	SCFD1	481	WDR45B
82	CASP8	182	HAX1	282	NBR1	382	SCOC	482	WDR6
83	CCL2	183	HDAC1	283	NCKAP1	383	SEC22B	483	WDR81
84	CCR2	184	HDAC6	284	NCOA4	384	SERPINA1	484	WIPI1
85	CD46	185	HERC1	285	NEDD4	385	SESN1	485	WIPI2
86	CDC37	186	HGF	286	NFE2L2	386	SESN2	486	XPA
87	CDK5	187	HGS	287	NFKB1	387	SESN3	487	ZC3H12A
88	CDK5R1	188	HIF1A	288	NKX2-3	388	SH3BP4	488	ZFYVE1
89	CDKN1A	189	HK2	289	NLRC4	389	SH3GLB1	489	ZKSCAN3
90	CDKN1B	190	HMGB1	290	NLRP6	390	SIRT1	490	ZMPSTE24
91	CDKN2A	191	HMGB4	291	NOD1	391	SIRT2		
92	CFLAR	192	HMOX1	292	NPC1	392	SLC38A9		
93	CHMP2B	193	HSP90AA1	293	NPRL2	393	SMCR8		
94	CHMP4A	194	HSP90AB1	294	NPRL3	394	SMURF1		
95	CHMP4B	195	HSPA5	295	NRBP2	395	SNCA		
96	CISD2	196	HSPA8	296	NRG1	396	SNRNP70		
97	CLEC16A	197	HSPB1	297	NRG2	397	SNX32		

98	CLN3	198	HSPB8	298	NRG3	398	SNX5
99	CLU	199	HTR2B	299	NUPR1	399	SNX6
100	CPTP	200	HTRA2	300	OPTN	400	SOGA1

Table S4. 7025 genes were significantly predicting the prognosis of UM patients by both Kaplan-Meier and univariate Cox regression analyses (p-value < 0.05).

Gene Symbol	Kaplan-Meier analysis (p-value)	Univariate Cox regression analysis			
		HR	HR_95L	HR_95H	P-value
ZYG11AP1	1.45E-03	0.021984597	0.001378508	0.350612811	6.90E-03
ZXDC	1.96E-03	0.130055004	0.041290422	0.409642317	4.93E-04
ZSWIM8	3.29E-03	0.029947556	0.005349125	0.167664076	6.55E-05
ZSWIM1	1.94E-03	5.458849038	1.609848841	18.51045394	6.45E-03
ZSCAN9	2.64E-02	0.290478068	0.117992681	0.715107982	7.16E-03
ZSCAN5B	4.08E-03	3.33E-12	8.80E-22	0.012575931	1.88E-02
ZSCAN5A	3.44E-02	0.004786713	0.000138199	0.165794058	3.14E-03
ZSCAN23	1.40E-02	0.004956597	0.000101224	0.24270816	7.51E-03
ZSCAN18	7.03E-07	0.223822875	0.124305388	0.403012938	6.08E-07
ZSCAN12P1	6.86E-03	0.119551843	0.02579037	0.554185258	6.64E-03
ZSCAN12	1.23E-02	0.250552336	0.101109634	0.620875286	2.80E-03
ZSCAN1	2.45E-05	0.004530395	0.000205743	0.099757959	6.24E-04
ZPBP2	2.76E-06	7.84E+133	2.79E+43	2.20E+224	3.71E-03
ZP1	1.86E-03	34.58024722	1.705469122	701.1522418	2.10E-02
ZNHIT1	2.25E-02	7.763494756	2.477091173	24.33170466	4.38E-04
ZNF99	8.85E-04	5.24E-21	1.10E-38	0.002492958	2.45E-02
ZNF98	7.68E-03	2.50E-15	4.12E-26	0.000151407	7.95E-03
ZNF90	2.54E-03	0.000912941	9.74E-06	0.085550721	2.52E-03
ZNF883	7.68E-06	0.032143171	0.004473971	0.230932051	6.34E-04
ZNF860	2.35E-02	0.004610975	9.36E-05	0.227221309	6.83E-03
ZNF853	6.69E-03	0.416882096	0.230071386	0.755377208	3.91E-03
ZNF844	8.33E-03	0.339335255	0.157511538	0.731047495	5.78E-03
ZNF843	4.91E-03	0.067758928	0.011577088	0.396582657	2.83E-03
ZNF840P	1.52E-04	3.84E-12	9.99E-21	0.001472862	9.15E-03
ZNF836	1.19E-03	0.079168917	0.020546507	0.305050258	2.29E-04
ZNF835	3.81E-09	0.048548561	0.01016019	0.231980179	1.50E-04
ZNF833P	1.75E-02	9.80E-10	3.26E-17	0.029433815	1.82E-02
ZNF831	2.20E-02	24.92475872	2.537075826	244.8659954	5.81E-03
ZNF826P	2.08E-04	0.000250796	8.32E-07	0.075614643	4.42E-03

ZNF8	1.15E-02	0.239187067	0.060468455	0.94612063	4.15E-02
ZNF793-AS1	2.05E-04	0.208851084	0.089181783	0.489099611	3.09E-04
ZNF790-AS1	1.58E-02	0.337822645	0.123940585	0.920797161	3.39E-02
ZNF781	1.67E-03	0.031071334	0.001938988	0.497902936	1.42E-02
ZNF778	3.21E-03	0.170417146	0.040347219	0.719801869	1.61E-02
ZNF773	1.47E-03	0.146811172	0.037069359	0.581437629	6.29E-03
ZNF746	1.54E-02	3.59352244	1.103109098	11.70637024	3.38E-02
ZNF740	2.13E-02	0.158051465	0.054458928	0.458699182	6.90E-04
ZNF74	2.21E-03	0.098871175	0.019991091	0.488993288	4.55E-03
ZNF732	6.31E-04	6.64E-07	1.07E-10	0.004100931	1.40E-03
ZNF727	1.11E-03	0.000421396	2.82E-06	0.062881291	2.34E-03
ZNF725P	9.60E-07	7.34E+87	2.16E+39	2.49E+136	3.87E-04
ZNF717	1.10E-03	0.085800583	0.016447585	0.4475879	3.57E-03
ZNF710	3.70E-02	5.72283219	1.858611974	17.6211112	2.36E-03
ZNF707	1.61E-02	2.794721242	1.031192559	7.574207893	4.33E-02
ZNF704	3.85E-04	2.438170098	1.465305436	4.056951732	6.02E-04
ZNF702P	6.65E-04	0.044304142	0.004110279	0.477548316	1.02E-02
ZNF69	3.75E-02	0.228205307	0.081895646	0.6359027	4.72E-03
ZNF688	2.40E-03	5.021640226	1.82773153	13.79681323	1.75E-03
ZNF687	6.94E-03	0.156571794	0.052833032	0.464003788	8.22E-04
ZNF683	4.68E-02	2.455671586	1.164721808	5.177479204	1.82E-02
ZNF677	1.86E-02	0.144046984	0.040900991	0.507311272	2.56E-03
ZNF676	1.37E-03	0.006981478	0.000176566	0.276050545	8.14E-03
ZNF671	2.05E-03	0.351425751	0.194958685	0.633467846	5.04E-04
ZNF667-AS1	1.45E-07	0.312539384	0.183767438	0.531546109	1.77E-05
ZNF667	6.83E-05	0.037475473	0.006033151	0.232782338	4.25E-04
ZNF665	1.81E-03	0.008504301	0.000561918	0.128707562	5.84E-04
ZNF663P	2.86E-03	0.002898171	2.14E-05	0.391985366	1.96E-02
ZNF649	3.63E-02	0.339236907	0.144770451	0.794925194	1.28E-02
ZNF646	1.60E-02	0.108217668	0.020695843	0.565865503	8.42E-03
ZNF619	9.91E-03	0.018644997	0.001991449	0.174564296	4.84E-04
ZNF610	3.50E-03	0.188885881	0.070327532	0.507310224	9.46E-04
ZNF599	1.36E-02	0.048362684	0.007569113	0.309012311	1.37E-03

ZNF582-AS1	1.57E-03	0.192932369	0.078520778	0.47405158	3.34E-04
ZNF581	3.91E-03	0.395170056	0.216481644	0.721351567	2.50E-03
ZNF580	2.17E-04	0.095107751	0.032524226	0.278115284	1.73E-05
ZNF576	7.97E-07	29.22236718	5.699107895	149.8386694	5.20E-05
ZNF571-AS1	2.47E-05	0.117570239	0.036813757	0.375478138	3.02E-04
ZNF561-AS1	6.88E-07	0.059305384	0.011563596	0.304155262	7.07E-04
ZNF560	1.06E-04	0.060103518	0.004522972	0.79868569	3.31E-02
ZNF549	2.35E-03	0.247103102	0.09728088	0.62766643	3.29E-03
ZNF544	1.99E-02	0.340556209	0.165051135	0.702682422	3.56E-03
ZNF542P	1.65E-03	0.311945186	0.152537128	0.63794173	1.42E-03
ZNF541	6.76E-05	0.009309299	0.00073177	0.118429421	3.13E-04
ZNF532	4.49E-02	0.616001537	0.382616342	0.991745129	4.61E-02
ZNF529-AS1	2.75E-03	0.226433394	0.075442284	0.679619954	8.08E-03
ZNF513	3.60E-02	5.869113955	2.020159851	17.05137274	1.15E-03
ZNF511	6.98E-03	5.901520295	1.501380601	23.19727708	1.10E-02
ZNF500	9.05E-05	0.081020271	0.016696811	0.393145989	1.82E-03
ZNF497	1.40E-06	0.002927293	0.000262492	0.032644945	2.13E-06
ZNF496	4.05E-02	0.180435371	0.045492351	0.715657082	1.49E-02
ZNF492	7.23E-04	4.76E-07	4.74E-12	0.047695047	1.32E-02
ZNF48	7.71E-06	0.029540414	0.005284192	0.165140867	6.05E-05
ZNF469	1.79E-02	2.529557404	1.348431698	4.745261232	3.84E-03
ZNF467	6.33E-07	1.811471412	1.192150383	2.752529145	5.38E-03
ZNF454	1.21E-05	0.020560986	0.00201859	0.209430439	1.04E-03
ZNF428	1.50E-02	3.488471533	1.209726749	10.05965491	2.08E-02
ZNF416	2.75E-04	0.053313874	0.010732259	0.264843521	3.38E-04
ZNF415	1.53E-05	0.204314998	0.085249634	0.489675046	3.69E-04
ZNF395	4.98E-04	0.224315626	0.102463111	0.491079176	1.85E-04
ZNF391	1.18E-05	0.091257044	0.024104496	0.345489414	4.24E-04
ZNF385C	1.68E-04	0.000250857	1.15E-06	0.054521451	2.53E-03
ZNF382	2.07E-02	0.023886952	0.001975808	0.288786449	3.32E-03
ZNF350-AS1	3.27E-03	0.08551432	0.011843435	0.617447473	1.48E-02
ZNF329	7.82E-03	0.265274151	0.113354609	0.620798536	2.22E-03
ZNF324	7.06E-04	0.006296823	0.000475493	0.083387139	1.21E-04

ZNF311	1.11E-02	0.259266439	0.091178404	0.737225955	1.14E-02
ZNF30-AS1	3.16E-06	0.000427143	4.28E-06	0.042631083	9.55E-04
ZNF285	2.99E-02	0.135183478	0.034694758	0.526724322	3.93E-03
ZNF229	1.67E-04	0.215319181	0.087906955	0.527402521	7.80E-04
ZNF213-AS1	3.37E-02	0.216281453	0.060834906	0.768928067	1.80E-02
ZNF208	5.70E-04	0.005105897	6.27E-05	0.416045426	1.87E-02
ZNF205-AS1	4.69E-03	0.013803929	0.001011009	0.188473597	1.32E-03
ZNF204P	7.24E-07	0.056500609	0.007328985	0.435574451	5.82E-03
ZNF197-AS1	3.41E-02	0.01157156	0.000428992	0.312129083	7.99E-03
ZNF19	1.86E-02	0.059766794	0.004015018	0.889677123	4.09E-02
ZNF185	7.12E-04	0.321181722	0.155242363	0.664494517	2.20E-03
ZNF18	5.97E-05	11.68982743	2.966628765	46.0630824	4.41E-04
ZNF174	1.74E-03	0.060964605	0.010661928	0.348593908	1.66E-03
ZNF154	2.72E-03	0.245330837	0.097909621	0.614722221	2.72E-03
ZNF135	4.25E-04	0.114040198	0.038117451	0.341186685	1.03E-04
ZMYND8	7.52E-03	2.950477596	1.491833493	5.83531479	1.87E-03
ZMYM6NB	1.50E-02	3.465556441	1.522117544	7.890377122	3.07E-03
ZMYM2	2.97E-02	2.094324226	1.084024619	4.046212499	2.78E-02
ZKSCAN8	5.77E-03	0.492829915	0.263907798	0.920326443	2.64E-02
ZKSCAN7	1.74E-02	0.150203319	0.041042894	0.549694105	4.18E-03
ZIM2-AS1	1.53E-07	1.04E-07	1.26E-11	0.000850384	4.70E-04
ZIK1P1	1.99E-03	1.86E+75	9.79335E+12	3.53E+137	1.78E-02
ZIK1	7.93E-05	0.162089108	0.055994901	0.469201273	7.93E-04
ZHX3	1.27E-07	4.112650312	1.93367553	8.747016924	2.40E-04
ZHX1-C8orf76	1.19E-03	7.66239776	3.037406459	19.32976051	1.61E-05
ZHX1	1.03E-02	0.433248038	0.228818214	0.82031871	1.02E-02
ZFP82	1.83E-03	0.040835978	0.00644383	0.258786633	6.87E-04
ZFP36L2	1.00E-02	2.652206377	1.340560781	5.247206067	5.08E-03
ZFP2	1.09E-02	0.133275992	0.031053997	0.571987235	6.70E-03
ZFAT-AS1	4.71E-02	1.17512E+18	495.8222188	2.79E+33	2.12E-02
ZER1	1.22E-03	0.035680186	0.008779734	0.14500163	3.17E-06
ZEB1	1.00E-02	3.049466474	1.337453251	6.952950147	8.02E-03
ZDHHC8P1	7.31E-03	0.196164595	0.047877599	0.803727614	2.36E-02

ZDHHC5	6.71E-04	8.348268466	2.025958248	34.40030734	3.31E-03
ZDHHC3	1.71E-04	0.06099231	0.013114448	0.283661335	3.62E-04
ZDHHC20P4	4.11E-03	6.06115E+17	547602031.3	6.71E+26	1.16E-04
ZDHHC19	5.49E-04	0.013413836	0.000392473	0.458454902	1.67E-02
ZDHHC15	2.32E-02	0.069693249	0.007790545	0.623467161	1.72E-02
ZDHHC12	2.57E-03	4.142919117	1.869009416	9.183355988	4.65E-04
ZDHHC11B	1.89E-03	2.938427313	1.605825956	5.376893457	4.72E-04
ZC3HC1	7.08E-04	38.79184036	7.533079124	199.7598663	1.22E-05
ZC3H7B	1.47E-02	0.224401737	0.073344561	0.686569514	8.82E-03
ZC3H12D	5.76E-03	35.69567375	3.428453193	371.6489777	2.78E-03
ZC3H12A	4.30E-02	3.540828117	1.134974493	11.04647183	2.94E-02
ZBTB8OS	3.89E-02	2.581483865	1.075310487	6.197334656	3.38E-02
ZBTB7B	4.50E-05	4.173856035	1.68228599	10.35559608	2.06E-03
ZBTB47	2.28E-04	0.307489165	0.15538697	0.608478218	7.08E-04
ZBTB20	1.47E-02	4.42E-07	8.07E-12	0.02418447	8.57E-03
ZBTB12	2.38E-05	0.224801897	0.102958018	0.490839799	1.80E-04
ZBTB11-AS1	7.40E-05	0.143264084	0.042435513	0.483665602	1.75E-03
ZBTB10	9.82E-03	2.611958007	1.361740262	5.010004346	3.86E-03
ZBP1	1.01E-02	15.64810396	3.220050926	76.04325623	6.50E-04
ZBED9	1.43E-02	0.01434329	0.000361296	0.569421779	2.38E-02
ZBED3	3.66E-02	0.31675275	0.129319127	0.775850465	1.19E-02
ZBED2	7.86E-03	4.265999367	1.584883346	11.48270669	4.09E-03
ZBED1	4.59E-06	0.149938492	0.067897318	0.331111036	2.67E-06
ZAP70	1.75E-03	2.31998181	1.367703137	3.935295208	1.80E-03
ZACN	1.62E-02	225333.8317	16.41777434	3092705177	1.12E-02
Z99756.1	5.33E-03	1.92E+34	7.73728E+11	4.77E+56	2.70E-03
Z95704.3	3.83E-03	1.91E-06	8.80E-11	0.041273464	9.72E-03
Z84466.1	1.88E-02	3117.487883	1.046692411	9285183.111	4.87E-02
Z69720.3	8.86E-04	7.577126055	2.968751262	19.33905342	2.27E-05
YY1AP1	2.43E-04	0.046256415	0.010275671	0.20822543	6.22E-05
YWHAZP3	2.34E-03	8.477329545	1.876716405	38.29300794	5.47E-03
YWHAZP2	4.41E-03	12.1934831	2.640737466	56.30284419	1.36E-03
YWHAZ	6.40E-04	2.7930328	1.562587913	4.99237973	5.28E-04

YTHDF3-AS1	3.29E-05	3.632560494	1.898252082	6.951392738	9.80E-05
YTHDF3	2.08E-02	1.939887939	1.289021556	2.919396653	1.49E-03
YTHDF1	1.47E-02	3.893219109	1.438225378	10.53878986	7.47E-03
YPEL3	2.60E-02	0.365258207	0.152807512	0.873082455	2.35E-02
YIPF2	3.41E-03	9.440296053	2.446696207	36.4242971	1.12E-03
YIF1A	1.17E-02	3.996122272	1.837335011	8.691388947	4.75E-04
YDJC	3.93E-04	6.444728197	2.652295568	15.65983898	3.90E-05
YBX2P1	1.64E-07	9.72E+44	4.92E+20	1.92E+69	2.84E-04
YAE1D1	3.01E-02	2.662237995	1.124666102	6.301880291	2.59E-02
XYLT2	1.07E-03	5.308110657	1.238404546	22.75188576	2.46E-02
XYLB	4.10E-03	0.124565315	0.029959127	0.517922899	4.17E-03
XXYLT1	2.00E-02	0.08769555	0.021698764	0.354421551	6.36E-04
XXyac-YX65C7_A.2	1.98E-02	3.017767103	1.161824463	7.838463186	2.33E-02
XXyac-YM21GA2.4	4.94E-03	0.000299628	1.73E-07	0.517993949	3.29E-02
XXyac-YM21GA2.3	5.11E-03	9.74E-07	1.25E-12	0.76023772	4.55E-02
XXyac-YM21GA2.1	2.82E-04	1.42E-22	9.66E-42	0.002076179	2.55E-02
XXbac-BPG55C20.7	2.24E-02	0.58235889	0.348864706	0.972130084	3.86E-02
XXbac-BPG27H4.8	6.62E-03	1.25E+27	62979.22334	2.49E+49	1.72E-02
XXbac-BPG248L24.12	7.62E-03	1.982824448	1.277341369	3.07794994	2.28E-03
XXbac-BPG181M17.5	1.22E-02	957886.2902	77.58623744	11826145656	4.17E-03
XXbac-B135H6.15	3.00E-04	7.262631335	3.321899466	15.87820897	6.76E-07
XX-C2158C6.1	2.91E-02	1253117528	8.30896732	1.88989E+17	2.92E-02
XRCC6P3	1.91E-03	6.8186E+18	674415.7993	6.89E+31	4.53E-03
XPO6	5.29E-03	3.931333258	1.422817378	10.86251927	8.29E-03
XKR5	1.72E-02	9.49215769	2.055463486	43.83491034	3.94E-03
XIRP1	1.03E-04	40337898.64	1018.983762	1.59683E+12	1.19E-03
XCR1	9.12E-04	12.09304835	2.241717856	65.23649614	3.75E-03
XCL2	9.95E-03	2.057651235	1.19151111	3.553410933	9.64E-03
XCL1	6.31E-03	3.435277747	1.333795187	8.847785113	1.06E-02
XAB2	2.17E-02	0.246103988	0.08102753	0.747488827	1.34E-02
WWTR1	4.79E-03	1.919702674	1.260432637	2.923804293	2.38E-03
WWP2	1.85E-04	0.334607084	0.182878671	0.612219565	3.83E-04
WWP1P1	5.86E-03	1.84467E+16	398.1841527	8.55E+29	1.97E-02

WWC3	2.26E-03	2.199848537	1.079851336	4.481481315	2.99E-02
WWC2-AS2	7.38E-03	0.055278637	0.007405031	0.41265565	4.76E-03
WSCD1	4.97E-03	2.221567664	1.250308454	3.947316255	6.50E-03
WRNIP1	1.92E-03	0.410049594	0.209342518	0.803184518	9.35E-03
WNT10B	1.24E-06	17.22938406	3.586543546	82.76817809	3.78E-04
WNK4	4.48E-07	0.405643132	0.249048667	0.660699584	2.89E-04
WNK3	2.31E-03	0.116730383	0.024730691	0.55097459	6.67E-03
WIZ	2.25E-03	0.050729497	0.009339321	0.275553439	5.55E-04
WISP2	3.06E-02	0.076435125	0.008571633	0.681588716	2.13E-02
WIPI1	1.02E-06	2.328872303	1.531424135	3.541570282	7.73E-05
WIPF2	3.93E-03	7.848071876	1.779773266	34.60678579	6.50E-03
WIPF1	4.69E-03	2.185485678	1.249247408	3.823380073	6.15E-03
WI2-1959D15.1	5.28E-04	2.87E+54	2.00054E+12	4.12E+96	1.13E-02
WI2-1896O14.1	7.19E-03	1.540721821	1.057618827	2.244498367	2.43E-02
WHSC1L1	4.99E-02	2.617237895	1.191252508	5.750194984	1.66E-02
WEE2-AS1	1.29E-03	0.01640897	0.001357787	0.198303825	1.23E-03
WDR87	1.30E-03	1.06E-22	1.48E-41	0.000762798	2.24E-02
WDR82	1.85E-04	0.14917054	0.051852989	0.429133408	4.17E-04
WDR72	4.90E-05	114.8918481	14.88783451	886.6391385	5.36E-06
WDR6	4.91E-05	0.317674762	0.181652665	0.55555064	5.79E-05
WDR55	5.27E-05	11.20176443	2.661152612	47.15232256	9.86E-04
WDR49	1.33E-02	3.66E+36	83575719702	1.60E+62	5.19E-03
WDR48	1.29E-02	0.367923223	0.167012295	0.810524144	1.31E-02
WDR46	4.76E-02	0.419842458	0.180974869	0.973989869	4.32E-02
WDR45	2.18E-03	4.438614427	1.17352292	16.78816638	2.81E-02
WDR4	1.23E-03	6.799388299	2.531480099	18.26270776	1.43E-04
WDR36	4.78E-02	2.402804459	1.228719632	4.698768635	1.04E-02
WDR34	6.85E-03	2.427278901	1.28135727	4.598001668	6.52E-03
WDR31	1.52E-03	0.079462763	0.016648967	0.379262623	1.49E-03
WDR25	7.33E-04	5.381432024	1.796593568	16.11928882	2.64E-03
WDFY3-AS2	2.42E-04	0.003996663	0.000208324	0.076675367	2.48E-04
WBSCR27	5.64E-03	0.311052813	0.137384562	0.704255638	5.10E-03
WBSCR22	9.72E-05	204.9884817	31.31769104	1341.742518	2.81E-08

WBSCR16	3.09E-02	20.22794453	2.118395398	193.1507877	9.00E-03
WBP1LP2	8.36E-04	381.7285691	14.57791642	9995.715182	3.59E-04
WBP1L	5.84E-04	4.115430375	1.656342289	10.22540286	2.31E-03
WASF4P	8.27E-04	3.82E-08	1.53E-13	0.009553991	7.07E-03
WASF3-AS1	2.32E-03	1.33E+49	728841.192	2.44E+92	2.60E-02
WASF2	1.98E-02	0.468906294	0.231216281	0.950941309	3.58E-02
WAS	1.49E-03	1.958413074	1.293815306	2.964396659	1.48E-03
WARSP1	2.92E-02	493126258.1	4.113866228	5.91107E+16	3.49E-02
WARS	5.58E-06	1.649120723	1.315183172	2.067848204	1.47E-05
VWC2	8.29E-03	18.62307045	3.091757875	112.175263	1.41E-03
VWA8-AS1	2.30E-02	5.98E-10	6.19E-17	0.005778189	9.65E-03
VWA5A	7.13E-04	0.304011234	0.160086156	0.577331812	2.74E-04
VTN	3.99E-03	1.240072	1.009108045	1.523898827	4.07E-02
VSTM2L	1.01E-02	1.88332021	1.161865414	3.052758926	1.02E-02
VSIG10L	7.49E-04	0.112997585	0.031590516	0.404186308	7.99E-04
VSIG10	3.58E-02	6.989233086	1.580308332	30.91129632	1.04E-02
VPS53	1.25E-04	3.313586911	1.658256312	6.621327556	6.94E-04
VPS37D	2.75E-02	0.343865656	0.127101208	0.930310509	3.55E-02
VPS33B	7.21E-03	0.305696036	0.103262764	0.904973511	3.23E-02
VPS28	1.29E-02	4.157393272	1.789638309	9.657772039	9.22E-04
VPREB1	1.06E-02	2.21E+22	25.70985338	1.90E+43	3.64E-02
VPRBP	1.88E-02	0.170230672	0.05955579	0.486577068	9.52E-04
VOPP1	1.05E-06	1.995939587	1.264227436	3.151153597	3.01E-03
VN2R19P	9.75E-03	2.15E-10	1.68E-18	0.027619814	1.95E-02
VN1R92P	1.88E-02	3.46E+35	1.590745787	7.53E+70	4.87E-02
VN1R85P	6.77E-03	0.010238984	0.000287579	0.364549345	1.20E-02
VN1R20P	4.97E-02	1.10E-07	1.44E-12	0.008453589	5.24E-03
VMP1	1.30E-03	2.553676602	1.395307937	4.67370966	2.36E-03
VMAC	2.90E-02	0.161833727	0.048412721	0.540976731	3.10E-03
VLDLR	2.21E-02	1.936425926	1.20128267	3.121451312	6.67E-03
VKORC1L1	4.08E-02	3.730960159	1.411552413	9.861528045	7.93E-03
VIMP	1.84E-02	2.963390016	1.506135902	5.830602917	1.66E-03
VIM	7.16E-05	0.285432336	0.161152501	0.505556028	1.72E-05

VILL	3.50E-05	0.231613644	0.086730831	0.618521461	3.52E-03
VGLL2	7.62E-03	2.67777E+11	641.7316891	1.12E+20	9.37E-03
VGF	1.26E-04	1.575664014	1.293896288	1.918791412	6.09E-06
VENTXP6	5.21E-03	360256.3235	39.05396575	3323212281	6.02E-03
VENTXP3	5.97E-03	14478130.44	369.2684788	5.67653E+11	2.25E-03
VDR	3.53E-02	2.259489822	1.217788327	4.192267361	9.74E-03
VDAC1P2	7.35E-03	7314.967906	82.42448356	649185.2075	1.01E-04
VDAC1P11	1.70E-03	33236990231	5166.693974	2.13811E+17	2.45E-03
VDAC1P1	3.55E-03	43.0827826	5.711992718	324.9524725	2.62E-04
VDAC1	7.12E-06	5.291343594	1.781142996	15.71929771	2.71E-03
VCP	1.21E-02	3.25394311	1.0022593	10.56427788	4.96E-02
VCAN-AS1	5.28E-04	6.23E+38	579480078.3	6.70E+68	1.13E-02
VCAN	5.77E-03	11.45895012	3.19113195	41.14763661	1.85E-04
Vax2os1_3	3.89E-02	0.323445428	0.114235521	0.91580048	3.35E-02
VAX1	2.68E-05	25.57594915	3.896818479	167.8623673	7.33E-04
VAV2	4.44E-05	2.317447108	1.353251286	3.968635505	2.20E-03
VAV1	4.78E-02	2.118910703	1.177874413	3.811766786	1.22E-02
VASP	2.94E-02	4.914441688	1.434810766	16.83269855	1.13E-02
VAPB	7.09E-03	4.639957442	1.579106004	13.63379343	5.26E-03
VAC14-AS1	2.08E-02	55517968813	1.303145989	2.37E+21	4.76E-02
UTS2R	2.61E-03	44.69855095	2.58743838	772.1770196	8.95E-03
UTS2B	4.25E-02	0.00175807	1.07E-05	0.28776582	1.47E-02
USP5	2.32E-03	4.707548346	1.448890704	15.29515744	9.97E-03
USP41	1.47E-02	0.00366228	1.53E-05	0.874690528	4.47E-02
USP4	2.21E-04	0.107911673	0.035133041	0.331452353	1.01E-04
USP35	1.59E-02	4.518279664	1.680030705	12.15147501	2.81E-03
USP31	4.61E-02	6.893371616	1.594407718	29.80327534	9.75E-03
USP3-AS1	3.78E-02	4.773791354	1.421657911	16.029935	1.14E-02
USP20	2.03E-02	0.082393414	0.021737894	0.312296793	2.41E-04
USP19	2.85E-07	0.074296061	0.02501413	0.220671463	2.86E-06
USP11	1.22E-02	0.259843724	0.108025753	0.625024673	2.62E-03
USMG5	9.13E-03	5.764248273	2.067604054	16.07007787	8.12E-04
UROS	4.61E-04	11.06346301	3.468067853	35.29348873	4.88E-05

URB1	3.01E-02	2.342275477	1.076019741	5.098655908	3.20E-02
UQCRQ	1.35E-05	14.45218593	4.768882093	43.79761841	2.34E-06
UQCRBP1	2.42E-03	16.22416044	3.497886412	75.25212397	3.72E-04
UQCRB	1.44E-03	4.30851423	1.806914442	10.2734775	9.86E-04
UQCR11	6.02E-04	17.8043412	4.417125945	71.76489184	5.15E-05
UQCR10	6.05E-04	8.279743913	3.205872291	21.38393331	1.26E-05
UPP1	2.25E-03	4.852547277	2.105429374	11.18404415	2.09E-04
UPK2	9.22E-03	0.2161266	0.069988338	0.667407003	7.75E-03
UNC93B3	6.56E-03	1.59E-05	1.15E-08	0.021964324	2.75E-03
UNC5D	2.19E-03	3.923297286	1.632700559	9.427485962	2.24E-03
UNC5CL	4.75E-03	0.042811733	0.005102184	0.359227416	3.69E-03
UNC5B-AS1	1.56E-02	19.63482179	3.458087359	111.4853925	7.79E-04
UNC45B	4.69E-02	9.71E+23	118601.1326	7.95E+42	1.29E-02
UMPS	1.43E-02	0.171504639	0.045873207	0.641198702	8.78E-03
ULK3	8.85E-05	4.883951506	2.020336537	11.8064401	4.29E-04
ULBP1	6.33E-06	17.85860864	5.997861093	53.17393947	2.24E-07
UGT8	3.99E-06	5.199112891	2.090075278	12.93291928	3.92E-04
UFSP1	5.65E-03	4.038073455	1.847578704	8.825625231	4.67E-04
UFD1L	1.97E-03	6.763664505	2.147099079	21.30649581	1.09E-03
UFC1	3.99E-06	158.8919274	17.82209907	1416.592092	5.61E-06
UCP2	2.11E-02	1.687498396	1.177635018	2.418109849	4.36E-03
UCK2	2.79E-02	5.340869666	1.640789705	17.38485358	5.40E-03
UCHL3	4.47E-03	10.20099993	2.658471862	39.14293813	7.12E-04
UCHL1	2.24E-04	1.378696081	1.100087085	1.727865829	5.30E-03
UBXN7-AS1	9.26E-04	0.000159929	4.99E-07	0.051221067	2.98E-03
UBXN7	2.19E-02	0.31973412	0.135083043	0.756793042	9.49E-03
UBXN2B	1.63E-02	2.248981844	1.328194303	3.808117024	2.56E-03
UBXN11	3.96E-05	0.191356534	0.080763951	0.453386969	1.72E-04
UBXN10-AS1	2.36E-02	0.219213168	0.078988175	0.608374774	3.57E-03
UBTD1	5.10E-03	2.932490933	1.240408419	6.932799667	1.43E-02
UBQLN4	1.69E-02	0.176461371	0.070966489	0.438779143	1.90E-04
UBQLN1P1	3.43E-03	3.25E+19	45.75892393	2.30E+37	3.22E-02
UBL5	4.71E-03	6.986387933	1.906545739	25.60107284	3.35E-03

UBE2Z	7.91E-03	5.041691364	1.381713287	18.39647346	1.43E-02
UBE2W	4.26E-02	2.313181857	1.38372851	3.866950968	1.38E-03
UBE2V2	1.18E-02	2.411613605	1.488466667	3.90729622	3.50E-04
UBE2SP2	2.05E-02	250.6334851	11.68208699	5377.219317	4.13E-04
UBE2S	8.18E-03	5.579874545	2.100708238	14.82119191	5.62E-04
UBE2R2	1.68E-02	0.22741916	0.072947067	0.709000055	1.07E-02
UBE2L5P	4.96E-02	59.05524569	5.686800199	613.2661464	6.36E-04
UBE2L2	1.77E-03	749750100.7	6186.429279	9.08642E+13	6.22E-04
UBE2G1	1.93E-02	3.178076948	1.006834936	10.03160769	4.87E-02
UBE2F	9.91E-03	8.668382845	2.303790073	32.61619278	1.40E-03
UBE2E1	1.20E-02	0.438755682	0.219287598	0.877872487	1.99E-02
UBE2DNL	1.71E-02	5.47E+20	538.932453	5.55E+38	2.40E-02
UBE2D2	6.29E-04	10.58179289	2.107138497	53.14047502	4.17E-03
UBE2CP4	2.95E-04	8.45185E+18	433276.8729	1.65E+32	5.25E-03
UBE2C	1.04E-02	1.980776853	1.160143753	3.381888607	1.23E-02
UBD	2.27E-03	1.563621279	1.183094922	2.066538752	1.68E-03
UBASH3A	1.83E-03	3.044964416	1.475056588	6.285730575	2.60E-03
UBAC2	4.96E-05	4.820236569	1.824133336	12.7373806	1.51E-03
UBA2	3.88E-02	1.857942438	1.011472139	3.412798011	4.59E-02
UAP1	3.64E-03	2.787590671	1.029170762	7.550410523	4.37E-02
U91319.1	1.60E-02	1.3315E+15	114.6925863	1.55E+28	2.33E-02
U82695.9	1.71E-02	0.200401919	0.059564062	0.674247655	9.41E-03
U47924.32	4.08E-03	8.563631835	1.500639799	48.86968228	1.57E-02
U47924.31	1.05E-03	3.93607347	1.430179677	10.83267691	7.99E-03
U47924.29	5.40E-03	1569943523	1922.518221	1.28203E+15	2.30E-03
TYSND1	3.22E-03	9.566605182	2.699055599	33.90813243	4.69E-04
TYROBP	1.99E-03	1.654351704	1.204500683	2.272210885	1.88E-03
TYMP	2.28E-04	1.885484753	1.391370684	2.555072343	4.31E-05
TXNRD2	1.02E-03	4.581795386	1.86395219	11.2625469	9.10E-04
TXNL4A	1.47E-02	8.518449302	1.906270581	38.06593841	5.04E-03
TXNDC2	9.52E-04	1.00688E+15	49920.46809	2.03E+25	4.32E-03
TXNDC17	8.28E-05	8.27155074	3.315856008	20.63375233	5.89E-06
TWIST1	4.19E-02	1.650671571	1.187934125	2.293659704	2.83E-03

TWF2	6.73E-03	9.931387068	2.490558151	39.60254816	1.14E-03
TVP23C-CDRT4	1.49E-03	1.93E-14	2.48E-22	1.50E-06	6.57E-04
TUSC2	1.33E-05	0.209215893	0.083793677	0.522369844	8.05E-04
TUSC1	7.89E-08	0.161606326	0.069795793	0.374185944	2.09E-05
TULP3P1	3.32E-06	3.19E+64	5.79E+26	1.76E+102	8.09E-04
TUBB8P8	3.52E-02	994100923.9	16.04965136	6.15737E+16	2.36E-02
TUBB6	3.30E-03	2.943888153	1.320871029	6.561183696	8.28E-03
TUBB4A	8.20E-03	0.372168635	0.233087701	0.594237672	3.47E-05
TUBB3	4.64E-03	1.779522261	1.117368542	2.834068939	1.52E-02
TUBA8	2.04E-02	7.57435526	2.768298419	20.72423161	8.06E-05
TTYH3	1.03E-03	2.49022528	1.602586927	3.869507382	4.97E-05
TTL	5.13E-03	4.434521032	2.032310053	9.676169615	1.83E-04
TTI1	9.32E-03	3.878372395	1.690785258	8.896323388	1.38E-03
TTC38	3.04E-04	2.64536747	1.199723124	5.832986718	1.59E-02
TTC28	2.11E-02	6.625551391	1.443812277	30.40418199	1.50E-02
TTC24	1.92E-02	24.59527702	1.856258044	325.8855381	1.51E-02
TTC21A	2.45E-05	0.034356985	0.004371884	0.269998545	1.35E-03
TTC1	7.10E-03	4.358058622	1.375679616	13.80603066	1.23E-02
TSTA3	4.37E-02	3.560135119	1.684342596	7.524931151	8.83E-04
TSPYL5	2.15E-04	2.081342429	1.36840886	3.165710506	6.13E-04
TSPYL1	2.31E-02	0.560209259	0.318905011	0.984099979	4.38E-02
TSPY26P	4.59E-03	0.158365616	0.04440803	0.564755251	4.50E-03
TSPAN8	1.46E-02	0.188932679	0.039819508	0.89643391	3.59E-02
TSPAN6	5.17E-04	0.354127244	0.181110644	0.692428133	2.41E-03
TSPAN5	1.67E-02	1.749388422	1.093147772	2.799584769	1.97E-02
TSPAN33	8.65E-04	3.253675009	1.680725367	6.298709636	4.64E-04
TSPAN3	2.01E-03	2.457973309	1.177214022	5.132144773	1.67E-02
TSPAN17	7.82E-05	4.133654285	1.648949163	10.36241634	2.47E-03
TSPAN14	1.18E-03	0.361758669	0.192912961	0.678385392	1.53E-03
TSPAN10	6.85E-03	2.521825417	1.536110072	4.140070137	2.55E-04
TSNAXIP1	3.34E-02	0.090154745	0.010507892	0.773502258	2.82E-02
TSKU	4.86E-02	0.510995619	0.294325931	0.887167916	1.71E-02
TSGA10IP	3.85E-05	0.208168382	0.055701406	0.777970944	1.96E-02

TSFM	3.99E-03	9.046046869	2.255263906	36.28442939	1.89E-03
TSEN2	1.37E-03	0.129626624	0.035286324	0.476191895	2.09E-03
TSC22D3	1.26E-04	3.054013577	1.713303265	5.443869232	1.53E-04
TSC22D1-AS1	6.64E-04	13.42209932	2.995249133	60.14616555	6.90E-04
TSC22D1	2.21E-05	2.481758821	1.545787844	3.984458068	1.68E-04
TSC2	3.07E-03	0.279169669	0.098520691	0.791059251	1.63E-02
TSACC	2.36E-02	10.44582922	2.724911589	40.04362878	6.21E-04
TRUB2	1.78E-02	3.912697502	1.255692583	12.19183895	1.86E-02
TRPV4	4.24E-02	4.000876429	1.716642502	9.324604385	1.32E-03
TRPV2	1.27E-04	1.915109704	1.380293493	2.657148784	1.01E-04
TRPM8	3.93E-03	2.953489411	1.425459296	6.119501082	3.57E-03
TRPM4	2.21E-06	5.236188472	2.436304577	11.25379395	2.22E-05
TRPM2	9.44E-03	2.133419527	1.403685457	3.242520508	3.89E-04
TRPC5OS	8.43E-03	5.80E+32	280.0409155	1.20E+63	3.42E-02
TRMU	4.23E-02	6.619737028	1.900364346	23.05921936	2.99E-03
TRMT2A	2.47E-03	4.362316316	1.465794608	12.98258537	8.12E-03
TRMT112P2	5.16E-03	1831875.557	5.510971297	6.08925E+11	2.62E-02
TRMT112P1	1.88E-02	4.32229E+13	1.194951867	1.56E+27	4.87E-02
TRIOBP	3.62E-03	0.227308573	0.082389085	0.627136316	4.22E-03
TRIML2	3.07E-02	5529.274646	36.11221613	846607.6411	7.87E-04
TRIM69	1.49E-03	1.842922407	1.281024188	2.651287174	9.86E-04
TRIM65	3.77E-04	4.81635971	1.869716321	12.40686654	1.13E-03
TRIM64	5.16E-03	6.47E+120	1.9874E+14	2.11E+227	2.62E-02
TRIM60P17	3.84E-03	6.39E-06	1.36E-10	0.300429392	2.93E-02
TRIM6	2.51E-02	2.611772187	1.243923344	5.483741413	1.12E-02
TRIM53BP	5.28E-04	4.76E+75	1.2426E+17	1.82E+134	1.13E-02
TRIM51	1.44E-02	3.163949468	1.499596534	6.675513051	2.50E-03
TRIM47	1.87E-03	2.260627776	1.39144908	3.672745207	9.87E-04
TRIM39	2.39E-02	0.214428585	0.065701587	0.699825075	1.07E-02
TRIM13	3.08E-02	8.04255646	2.221497663	29.11671503	1.49E-03
TRIL	6.90E-03	2.576721488	1.346891508	4.929494014	4.24E-03
TRIB1	5.67E-05	2.840031986	1.764626528	4.570815159	1.71E-05
TRI-TAT2-3	1.24E-04	24801.01871	94.6877787	6495986.468	3.68E-04

TRI-TAT2-2	6.97E-03	0.151435267	0.035866635	0.639386453	1.02E-02
TRGV7	4.87E-02	2.812096538	1.240940778	6.372493417	1.32E-02
TRGV6	4.42E-03	3031.745062	30.7495026	298914.6928	6.20E-04
TRGV4	1.42E-02	9.711348974	1.064311513	88.61155569	4.39E-02
TRGV3	2.53E-02	3.060890759	1.079868957	8.676101091	3.53E-02
TRGV2	1.86E-05	3.900875966	1.168740652	13.01985456	2.69E-02
TRGV10	1.73E-04	2.384412563	1.175338891	4.837262949	1.61E-02
TRGC2	1.53E-04	1.986101227	1.147336135	3.438049204	1.43E-02
TRG-AS1	4.19E-02	4.390085029	1.321476375	14.58432926	1.57E-02
TREX2	4.00E-08	0.018697433	0.002611778	0.133852871	7.42E-05
TRBV7-7	2.84E-02	25564.31024	14.23152273	45921576.37	7.94E-03
TRBV7-6	2.94E-03	1.724670538	1.084326021	2.743168021	2.13E-02
TRBV7-4	2.73E-03	1030.786191	15.86877791	66956.64763	1.12E-03
TRBV7-3	2.52E-02	2.21543496	1.17569502	4.174681339	1.39E-02
TRBV6-8	2.15E-02	3930416.719	4.134804338	3.73613E+12	3.06E-02
TRBV6-6	1.49E-02	2.589015883	1.343631103	4.988722891	4.47E-03
TRBV6-5	5.55E-03	2.159757841	1.232095113	3.785871628	7.17E-03
TRBV6-4	5.23E-03	254.4417642	2.828831801	22885.98825	1.58E-02
TRBV5-6	2.38E-02	1.882275539	1.008069286	3.514600887	4.71E-02
TRBV5-5	2.32E-04	32.55244718	4.980888943	212.7455218	2.77E-04
TRBV5-4	1.99E-03	4.813897307	1.360929357	17.02778118	1.48E-02
TRBV5-1	1.52E-02	1.67389643	1.050848315	2.66634986	3.01E-02
TRBV4-1	1.73E-02	5.248506189	2.212579195	12.45009322	1.69E-04
TRBV3-1	1.07E-02	3.822785096	1.562487818	9.352831885	3.31E-03
TRBV29-1	3.56E-02	1.652985513	1.129673545	2.41871744	9.66E-03
TRBV28	3.97E-06	1.627206927	1.237957762	2.138847112	4.83E-04
TRBV25-1	1.96E-02	3.695189267	1.510573777	9.039229945	4.19E-03
TRBV23-1	7.80E-03	14.0048124	2.903692198	67.54668091	1.01E-03
TRBV20-1	3.16E-03	1.605480449	1.13802307	2.264951862	7.01E-03
TRBV19	5.56E-03	2.138712354	1.252913138	3.650764281	5.33E-03
TRBV15	2.36E-02	26.70336869	4.240130511	168.1716866	4.68E-04
TRBV14	1.50E-03	8.157826371	2.501783158	26.60107886	5.00E-04
TRBV13	3.58E-03	25.59240574	3.766883033	173.8761799	9.11E-04

TRBV12-4	4.36E-03	2.116596366	1.218045319	3.678007795	7.82E-03
TRBV12-3	7.94E-03	3.658394425	1.394417873	9.598162806	8.40E-03
TRBV11-2	1.84E-03	7.188598997	1.778509633	29.05576364	5.64E-03
TRBV11-1	2.03E-04	5.763824396	1.00020417	33.21489018	5.00E-02
TRBJ2-7	8.47E-06	1.820248187	1.21175814	2.734294373	3.91E-03
TRBJ2-3	4.73E-02	1.715910527	1.119179956	2.630809212	1.33E-02
TRBJ2-1	2.58E-02	1.887733619	1.105259897	3.224163136	2.00E-02
TRBC2	1.45E-05	1.513065445	1.202907485	1.903194609	4.03E-04
TRAV9-2	1.66E-02	6.409938697	1.354854298	30.32600196	1.91E-02
TRAV8-6	1.77E-03	3.431519468	1.246444092	9.447135203	1.70E-02
TRAV8-5	1.26E-02	10.12963801	1.123224581	91.35267152	3.91E-02
TRAV8-4	3.79E-03	13.79076512	2.954636455	64.36839372	8.43E-04
TRAV6	6.94E-03	13.90517514	1.33105354	145.2638004	2.79E-02
TRAV5	1.67E-03	2.869756672	1.157065383	7.117578205	2.29E-02
TRAV41	8.43E-04	12.79519907	2.087971372	78.40965706	5.85E-03
TRAV4	1.15E-04	2.685328922	1.394575213	5.170744002	3.13E-03
TRAV39	4.92E-04	4.328246861	1.527178942	12.26688005	5.84E-03
TRAV38-1	4.31E-03	237.2641357	8.8806055	6339.012594	1.10E-03
TRAV35	2.87E-04	8.993527224	1.75469281	46.09555102	8.43E-03
TRAV30	9.45E-03	3.612609054	1.550625253	8.416568832	2.92E-03
TRAV3	2.64E-02	3.437142723	1.582463161	7.465545099	1.81E-03
TRAV27	8.09E-05	20.08038135	2.781464804	144.967398	2.94E-03
TRAV26-2	2.15E-02	3.325380436	1.141470286	9.687641613	2.76E-02
TRAV26-1	2.25E-02	4.373438759	1.450944943	13.18242065	8.76E-03
TRAV23DV6	1.18E-02	12.11542091	2.503851108	58.62306402	1.93E-03
TRAV22	2.51E-06	18.32190026	2.412716619	139.1344621	4.93E-03
TRAV21	9.32E-04	3.536973532	1.651407568	7.57546593	1.15E-03
TRAV20	2.87E-02	61.78112746	2.647461939	1441.723356	1.03E-02
TRAV2	2.07E-03	3.964836146	1.529404473	10.27846193	4.59E-03
TRAV19	2.10E-03	1.62825589	1.115126803	2.377502931	1.16E-02
TRAV18	2.38E-02	35238.87433	23.51818135	52800777.65	5.01E-03
TRAV16	9.98E-03	4.305110774	1.331662468	13.91792531	1.48E-02
TRAV14DV4	8.33E-05	2.398316258	1.238623687	4.64380016	9.47E-03

TRAV12-3	4.00E-03	2.819322686	1.095719429	7.254211431	3.16E-02
TRAV10	2.95E-04	25.40377845	2.724806273	236.8432448	4.51E-03
TRAV1-2	4.71E-02	1.907726282	1.042298265	3.49172563	3.62E-02
TRAT1	3.47E-04	3.204734987	1.332149707	7.709588707	9.31E-03
TRANK1	2.60E-02	3.170178108	1.376026304	7.303660699	6.74E-03
TRAM1	2.03E-03	2.1894456	1.42598254	3.361662502	3.41E-04
TRAK2	2.02E-02	2.104413526	1.307183288	3.38786177	2.19E-03
TRAJ9	7.71E-03	14.33600785	1.864536213	110.2264036	1.05E-02
TRAJ61	5.16E-03	1078.33943	2.28524933	508835.4744	2.62E-02
TRAJ59	1.99E-03	1399.270022	3.491074946	560846.337	1.78E-02
TRAJ58	1.99E-03	4072.638318	4.197954073	3951063.443	1.78E-02
TRAJ57	1.99E-03	4072.638318	4.197954073	3951063.443	1.78E-02
TRAJ52	2.76E-06	33373.40895	29.36026399	37935095.72	3.71E-03
TRAJ21	5.16E-03	642.4016434	2.149366012	192000.743	2.62E-02
TRAJ18	2.55E-02	6.000485108	1.472500069	24.45216968	1.24E-02
TRAIP	1.82E-04	0.039730631	0.004723865	0.334159237	2.99E-03
TRAF6P1	2.59E-03	5.12E+40	7.52E+21	3.49E+59	2.27E-05
TRAF3IP3	5.16E-03	4.009394571	1.661073605	9.677623419	2.01E-03
TRAF3	5.87E-04	3.939372552	1.677257449	9.252399571	1.65E-03
TRABD2B	5.28E-06	16.45848455	3.708329399	73.04683175	2.30E-04
TRABD2A	4.50E-02	0.265730993	0.084624345	0.834428442	2.32E-02
TRABD	1.23E-02	11.68215851	2.023782089	67.43454652	5.99E-03
TPTE2P3	5.19E-05	0.129539296	0.032574806	0.515135196	3.71E-03
TPTE2P2	6.46E-04	2.55E-15	1.10E-26	0.000592954	1.19E-02
TPT1P8	4.55E-03	10531027473	3007.127892	3.68799E+16	2.69E-03
TPT1P4	2.21E-02	3.533630729	1.155024174	10.81063619	2.69E-02
TPT1P13	2.43E-02	96.6739485	1.300619196	7185.694589	3.76E-02
TPT1-AS1	1.99E-03	0.236468056	0.080214965	0.697091141	8.95E-03
TPST2	1.80E-03	4.859228662	2.056419256	11.48214457	3.14E-04
TPRXL	1.66E-03	4.80E-07	3.15E-12	0.073238308	1.69E-02
TPRG1L	8.45E-03	0.250481691	0.123192283	0.509293895	1.32E-04
TPR	7.85E-03	1.781929768	1.115692846	2.846010628	1.56E-02
TPPP	1.45E-05	2.230603573	1.353729732	3.675469472	1.64E-03

TPM4	3.88E-03	4.201047892	1.755445786	10.05374449	1.26E-03
TPM3P2	3.10E-02	2.64133E+14	2.462035564	2.83E+28	4.39E-02
TPM3	8.69E-04	8.400906261	1.865443292	37.83295172	5.57E-03
TPK1	3.41E-03	20.45242774	2.529207583	165.3884811	4.65E-03
TPI1P4	3.95E-02	1437815.392	14.65100087	1.41104E+11	1.56E-02
TPI1P3	2.26E-03	3386.456118	19.61384245	584693.4414	1.99E-03
TPI1P1	6.44E-03	3.190956049	1.593190195	6.391076554	1.06E-03
TPI1	7.49E-03	2.708684965	1.301596634	5.636903208	7.70E-03
TPBGL	3.93E-02	2.408721617	1.231509121	4.711243895	1.02E-02
TP53RK	1.96E-03	4.232806303	1.639663825	10.92702597	2.86E-03
TP53INP2	2.22E-02	2.549831387	1.309781511	4.963911953	5.89E-03
TOX2	1.98E-03	2.883826415	1.411237301	5.893023649	3.68E-03
TOR3A	3.99E-05	7.111933937	2.483273778	20.36811437	2.58E-04
TOR1AIP2	4.23E-03	5.875693375	1.843472856	18.72757308	2.75E-03
TOPAZ1	8.16E-03	1.52E+71	2.51E+19	9.19E+122	7.06E-03
TOMM70A	1.58E-02	0.451869712	0.248249864	0.822502917	9.34E-03
TOMM7	2.74E-02	0.27650353	0.118565705	0.644825602	2.92E-03
TOMM40	5.10E-03	7.717747908	2.393568224	24.88486945	6.24E-04
TOMM20L	3.22E-02	0.182195947	0.040392207	0.82182593	2.67E-02
TOM1L2	7.13E-04	0.117619141	0.034451861	0.401553412	6.35E-04
TOB2P1	2.04E-06	0.016713451	0.001736354	0.160877037	3.98E-04
TNXB	3.46E-03	0.01532767	0.000955833	0.245793546	3.17E-03
TNRC18	1.20E-04	7.271017588	2.092509346	25.26521416	1.80E-03
TNPO3	3.00E-02	7.772633871	2.01008052	30.05543145	2.96E-03
TNPO2	5.64E-03	0.116697578	0.030577202	0.445375112	1.67E-03
TNPO1	3.72E-02	2.195494411	1.148557932	4.196737122	1.74E-02
TNNT1	2.05E-02	11.24029682	2.372781133	53.24733533	2.30E-03
TNNI3	6.14E-03	0.028511913	0.001261611	0.644358017	2.53E-02
TNKS1BP1	6.56E-04	3.156432598	1.259909598	7.907763197	1.42E-02
TNK2	9.08E-04	0.08627063	0.025808757	0.288375827	6.90E-05
TNIP2	1.83E-02	8.176164245	2.341950613	28.54443701	9.88E-04
TNFSF9	2.51E-03	1.572274577	1.052062779	2.349714668	2.73E-02
TNFSF18	1.54E-02	562.5992329	5.451941898	58055.99231	7.43E-03

TNFSF15	2.17E-02	103.4801114	3.73889847	2863.980805	6.17E-03
TNFSF13B	7.85E-03	1.975000915	1.22624403	3.180956252	5.13E-03
TNFSF13	1.04E-03	0.171385623	0.063492321	0.462623373	4.99E-04
TNFSF10	1.86E-02	1.910540424	1.099634914	3.319433266	2.16E-02
TNFRSF9	7.31E-05	2.554090245	1.30348351	5.004571928	6.29E-03
TNFRSF8	1.11E-05	4.757249678	1.612017275	14.03919477	4.73E-03
TNFRSF4	4.08E-03	2.959973595	1.572433336	5.571901512	7.73E-04
TNFRSF21	2.15E-05	2.373024349	1.479703092	3.805658441	3.36E-04
TNFRSF1B	1.16E-04	1.911934216	1.351843763	2.704079085	2.48E-04
TNFRSF1A	7.14E-04	5.356181677	2.080627395	13.7884766	5.04E-04
TNFRSF19	1.97E-04	2.213458257	1.554510969	3.151729099	1.05E-05
TNFRSF18	1.16E-05	4.129554565	1.966057114	8.673817654	1.80E-04
TNFRSF13B	1.55E-02	11.77152946	2.090513803	66.28461656	5.17E-03
TNFRSF12A	5.71E-03	1.874648385	1.232258041	2.851924231	3.33E-03
TNFRSF11B	1.32E-02	5.886743549	2.505482106	13.83117027	4.75E-05
TNFRSF11A	2.58E-02	89.65226444	5.221097356	1539.4328	1.94E-03
TNFAIP8L3	2.00E-03	18.82422368	5.684385234	62.33768165	1.55E-06
TNFAIP8L2	3.76E-04	2.064639376	1.318620804	3.232722964	1.53E-03
TNFAIP8	3.62E-02	5.818048527	1.804751623	18.75587102	3.19E-03
TNFAIP6	6.79E-03	2.978585452	1.313768879	6.753068545	8.96E-03
TNFAIP2	3.44E-03	2.16222434	1.435509718	3.256832075	2.24E-04
TMX2	1.28E-02	3.742867886	1.363801126	10.27206955	1.04E-02
TMSB4XP8	2.51E-03	2.222840826	1.372545191	3.599897017	1.17E-03
TMSB4XP2	3.91E-02	13.23645082	1.418544461	123.5094387	2.34E-02
TMSB4X	7.98E-03	1.584972868	1.136029767	2.211332012	6.72E-03
TMSB15A	4.98E-02	0.091708496	0.019527048	0.43070762	2.47E-03
TMSB10P1	2.84E-02	3.64053664	1.30301116	10.17144553	1.37E-02
TMPRSS6	7.93E-03	2985019638	61.13553354	1.45747E+17	1.57E-02
TMPRSS5	4.42E-02	0.369465227	0.164753039	0.828540431	1.57E-02
TMPRSS11GP	2.28E-02	2.75551E+13	650.4071257	1.17E+24	1.32E-02
TMPRSS11D	6.25E-03	100373306.5	110.7049743	9.10059E+13	8.48E-03
TMPPE	8.58E-04	0.001555687	3.29E-05	0.073482935	1.01E-03
TMEM9B-AS1	8.54E-03	0.265315174	0.111381574	0.6319909	2.73E-03

TMEM8B	1.05E-05	0.140928096	0.053355952	0.372230794	7.68E-05
TMEM8A	4.87E-02	3.793589642	1.426004168	10.09206192	7.57E-03
TMEM87B	1.24E-03	3.439935142	1.767195487	6.696007241	2.77E-04
TMEM87A	1.20E-02	2.922700111	1.415135019	6.03629747	3.75E-03
TMEM81	1.36E-03	3.828758749	1.558323842	9.40715477	3.42E-03
TMEM70	1.36E-04	2.167692691	1.394460211	3.369684962	5.88E-04
TMEM68	1.77E-02	2.554730662	1.490401959	4.379119815	6.47E-04
TMEM67	4.00E-02	5.067380227	1.973024843	13.01470808	7.46E-04
TMEM64	1.00E-02	2.38044779	1.440537033	3.933624442	7.14E-04
TMEM62	7.93E-03	3.270634266	1.479770907	7.228854446	3.41E-03
TMEM53	1.09E-02	0.476169635	0.2361193	0.960266784	3.81E-02
TMEM44-AS1	2.91E-02	0.293877615	0.132278003	0.652898064	2.64E-03
TMEM43	7.62E-03	0.311941664	0.143492756	0.678135987	3.28E-03
TMEM42	6.02E-03	0.238391412	0.08327115	0.682474852	7.54E-03
TMEM41A	1.72E-04	0.066469868	0.013072604	0.337977303	1.09E-03
TMEM39B	3.33E-02	3.483484481	1.119686818	10.83755202	3.11E-02
TMEM259	1.22E-02	6.023100055	1.535549835	23.62524058	1.00E-02
TMEM255B	8.98E-05	15.22035475	3.182752755	72.78579789	6.50E-04
TMEM255A	1.36E-03	2.364237646	1.633323438	3.422236842	5.12E-06
TMEM254	1.50E-02	0.146925658	0.035068714	0.615567168	8.70E-03
TMEM251	4.48E-02	2.501728838	1.127590889	5.550459161	2.41E-02
TMEM25	2.25E-04	0.164984039	0.061567526	0.442111853	3.40E-04
TMEM230	5.50E-03	3.775349963	1.458833584	9.770317529	6.17E-03
TMEM229B	1.37E-02	2.329826568	1.209540536	4.487730402	1.14E-02
TMEM223	1.95E-02	5.211652871	1.471838497	18.45401225	1.05E-02
TMEM221	1.95E-05	0.076553758	0.011887007	0.493015413	6.85E-03
TMEM220	1.57E-04	0.285217607	0.141388423	0.575358868	4.59E-04
TMEM217	1.43E-02	0.000197652	1.15E-06	0.033917817	1.16E-03
TMEM214	5.08E-04	4.314907305	1.969814337	9.451867975	2.58E-04
TMEM209	4.64E-02	2.895480502	1.429112001	5.866445268	3.17E-03
TMEM208	7.59E-05	6.426606558	2.937160771	14.06163131	3.21E-06
TMEM207	3.61E-03	6.39E-26	2.78E-47	0.000146858	2.08E-02
TMEM206	9.17E-03	2.861594645	1.537024265	5.32764778	9.15E-04

TMEM205	4.85E-02	3.458172756	1.559156722	7.670145432	2.27E-03
TMEM203	3.07E-02	0.187472247	0.049686043	0.707358468	1.35E-02
TMEM200C	1.84E-03	2.893071707	1.307452619	6.401657527	8.75E-03
TMEM199	3.43E-03	9.826581618	1.862923476	51.83342608	7.08E-03
TMEM198B	7.79E-05	0.156910053	0.062043419	0.396831201	9.14E-05
TMEM191A	1.71E-03	23.64841319	5.426888641	103.051211	2.53E-05
TMEM189	4.39E-02	6.890894736	2.108470587	22.5207933	1.40E-03
TMEM187	4.87E-04	2.92363283	1.154443105	7.40411449	2.36E-02
TMEM185B	7.46E-03	7.786351828	2.147919463	28.22604657	1.79E-03
TMEM185A	8.72E-06	0.076166696	0.024131448	0.240406853	1.13E-05
TMEM184B	1.93E-03	3.253721499	1.358732101	7.791604822	8.10E-03
TMEM179B	4.57E-04	3.52390914	1.592113548	7.79965452	1.89E-03
TMEM175	2.69E-02	4.786159286	1.724029531	13.2870814	2.65E-03
TMEM173	5.50E-05	3.736418261	1.771624729	7.880236255	5.36E-04
TMEM171	2.49E-03	2.090238377	1.116008536	3.914931055	2.13E-02
TMEM161A	2.16E-04	4.150047775	2.030767524	8.480978905	9.51E-05
TMEM158	3.96E-04	4.024431425	1.766998322	9.165853807	9.15E-04
TMEM156	3.07E-02	22.97383934	3.969897984	132.9498381	4.67E-04
TMEM155	4.81E-02	7.346839629	1.840707698	29.32353279	4.74E-03
TMEM154	1.72E-03	3797.219145	32.21557898	447574.549	7.07E-04
TMEM150B	1.78E-03	3.236504998	1.518661206	6.897499295	2.35E-03
TMEM147	1.58E-02	4.891914364	1.524869547	15.6936875	7.60E-03
TMEM139	2.93E-02	0.020031041	0.000676334	0.59326081	2.37E-02
TMEM129	3.24E-02	2.813589617	1.088725675	7.271148938	3.27E-02
TMEM127	4.19E-02	3.628620147	1.093770539	12.03806805	3.52E-02
TMEM119	9.35E-03	1.666891412	1.251525779	2.220111662	4.75E-04
TMEM106A	1.04E-05	6.86148621	2.671558351	17.62267067	6.29E-05
TMEM104	3.77E-02	2.646546861	1.087092591	6.443066895	3.20E-02
TMEM101	9.84E-04	0.35544292	0.194208086	0.650537637	7.96E-04
TMED9	4.85E-03	5.77328914	1.801013765	18.50672557	3.18E-03
TMED7-TICAM2	5.87E-04	216439.9681	32.29361882	1450635187	6.28E-03
TMED3	9.49E-03	6.881004608	2.01260138	23.52588291	2.10E-03
TMED2	1.16E-02	3.036880498	1.269266645	7.266119531	1.26E-02

TMED1	2.28E-04	4.194712671	1.74868104	10.0622206	1.32E-03
TMCO2	1.62E-02	1.38884E+16	1487933.588	1.30E+26	1.51E-03
TMCO1	1.09E-02	3.840168748	1.823837938	8.085639465	3.97E-04
TMBIM6	4.70E-02	2.839378276	1.00448298	8.026088198	4.90E-02
TMBIM4	4.03E-02	3.982890644	1.890222827	8.392353355	2.79E-04
TM9SF2	6.22E-03	2.265675807	1.163871405	4.410527521	1.61E-02
TM9SF1	1.15E-03	10.89503787	2.789857069	42.54764571	5.90E-04
TM7SF2	3.82E-02	0.347520638	0.141237466	0.855088931	2.14E-02
TM6SF2	2.78E-03	3.672521391	1.55504541	8.673324443	3.01E-03
TM4SF20	1.04E-02	5.95927E+11	306.7424898	1.16E+21	1.30E-02
TM4SF19	1.25E-03	181.4547742	13.53760624	2432.175562	8.59E-05
TM4SF1	9.76E-03	3.286293079	1.587134562	6.804541	1.36E-03
TLR8	4.25E-03	3.893239842	1.132710968	13.38145113	3.09E-02
TLR7	3.40E-02	3.165700856	1.027670157	9.751827321	4.47E-02
TLR12P	6.69E-03	2.28162E+18	201233.5444	2.59E+31	5.85E-03
TLR1	2.73E-03	0.167231479	0.058984755	0.474128739	7.69E-04
TLL2	6.06E-04	1247362673	61520.92559	2.52908E+13	3.48E-05
TLCD2	3.43E-04	2.355278541	1.014184143	5.469753244	4.63E-02
TLCD1	3.38E-04	2.870128477	1.714513857	4.804649108	6.05E-05
TKT	2.55E-03	0.305845029	0.149592105	0.625308281	1.17E-03
TJP2	5.11E-05	0.174429789	0.08673537	0.350788279	9.65E-07
TJAP1	2.55E-02	0.192928678	0.067152792	0.554280373	2.24E-03
TISP43	4.17E-04	1.18E+133	7.81E+50	1.78E+215	1.50E-03
TIPIN	1.98E-02	5.262172584	1.539111314	17.99120054	8.11E-03
TIPARP-AS1	2.18E-03	0.022328977	0.002096884	0.237773367	1.63E-03
TIPARP	4.46E-04	0.128222154	0.040918061	0.401801075	4.24E-04
TINAGL1	4.58E-02	2.810430867	1.297598957	6.087028363	8.78E-03
TIMP3	3.65E-04	0.204955847	0.079809648	0.526338614	9.89E-04
TIMP1	1.93E-03	2.370896052	1.353801757	4.152120546	2.53E-03
TIMM8B	6.72E-04	3.939673508	1.422535506	10.91081894	8.34E-03
TIMM44	1.59E-02	5.758466039	1.464132979	22.64816898	1.22E-02
TIMM23	3.44E-05	10.96810373	3.617202073	33.25755572	2.32E-05
TIMM17A	9.87E-04	2.91894607	1.51343116	5.62975468	1.39E-03

TIGIT	2.44E-04	2.448438131	1.321521608	4.536323314	4.43E-03
TIFAB	2.07E-02	57.31363917	5.131499213	640.1351922	1.01E-03
TIE1	1.60E-02	3.884326193	1.49390826	10.09967639	5.38E-03
TICAM2	4.94E-02	1526609920	405.1922233	5.75168E+15	6.20E-03
TICAM1	1.93E-03	5.181474116	1.787830944	15.01689748	2.44E-03
THYN1	1.01E-03	5.56379896	1.766260225	17.52621637	3.37E-03
THY1	7.33E-03	1.573668983	1.067179383	2.32054152	2.21E-02
THPO	3.53E-03	257856.9301	304.2911002	218508514.9	2.92E-04
THOP1	1.48E-05	0.064487374	0.016695546	0.249085685	7.01E-05
THOC3	4.27E-02	5.002163723	1.469505085	17.0272578	1.00E-02
THG1L	1.04E-02	0.504073631	0.254201481	0.999562332	4.99E-02
THEMIS2	1.07E-05	2.900287815	1.759209627	4.781504877	2.99E-05
THEMIS	3.97E-03	6.137922582	1.55256524	24.26570727	9.68E-03
THEM6	1.87E-03	3.433724383	1.758206367	6.705960895	3.03E-04
THBS2	4.06E-04	2.052042076	1.473009492	2.858689441	2.14E-05
THAP8	4.45E-02	4.503072924	1.48568847	13.64866603	7.82E-03
THAP3	2.24E-02	0.294438639	0.088260579	0.982251788	4.67E-02
THADA	1.01E-03	7.260044007	1.941513481	27.14801597	3.22E-03
TGS1	3.49E-02	1.888727333	1.235029722	2.888425173	3.35E-03
TGIF2	1.31E-02	0.176673552	0.054721971	0.570402407	3.75E-03
TGFBI	1.30E-04	2.254786348	1.487102585	3.418769846	1.29E-04
TFPT	1.83E-02	6.57565728	2.181445586	19.82138309	8.21E-04
TFPI2	3.31E-02	1.390905583	1.149591016	1.682875313	6.89E-04
TFG	1.92E-04	0.241244999	0.09581652	0.607402043	2.54E-03
TFF3	3.86E-03	0.553457241	0.351847898	0.870589023	1.05E-02
TFEC	1.75E-02	11.15594541	1.861434912	66.85977424	8.29E-03
TFDP2	4.51E-04	0.18436179	0.068720892	0.494598782	7.85E-04
TFDP1	3.76E-03	4.672051794	1.416980998	15.40462998	1.13E-02
TFCP2L1	2.83E-02	3.228798098	1.247045453	8.35986943	1.57E-02
TFAP4	3.64E-03	0.155769923	0.038946294	0.623018685	8.56E-03
TFAP2C	1.26E-04	2.480837683	1.320146903	4.662023292	4.76E-03
TFAP2A-AS1	5.88E-05	0.405241617	0.238836304	0.687587128	8.12E-04
TFAP2A	3.87E-05	0.275182898	0.151206856	0.50080816	2.41E-05

TEX261	8.67E-04	5.886512463	1.952954673	17.74287414	1.64E-03
TESPA1	5.24E-03	3.997025886	1.339610341	11.92601717	1.30E-02
TESK2	1.23E-02	0.192389675	0.071568647	0.517178797	1.09E-03
TESK1	5.79E-03	0.105428822	0.03439281	0.323184891	8.28E-05
TES	1.57E-02	0.458598425	0.254793196	0.825424379	9.33E-03
TERF1	1.50E-02	2.290424545	1.297257693	4.043949499	4.27E-03
TEP1	1.82E-02	3.755287829	1.690060798	8.344188975	1.16E-03
TEKT3	9.90E-04	0.054655755	0.007719575	0.386971003	3.61E-03
TEKT2	4.63E-03	0.005029273	5.45E-05	0.464056798	2.19E-02
TECRL	1.65E-03	1.23E+53	7.93969E+11	1.89E+94	1.15E-02
TEAD4	1.59E-03	3.206202789	1.530838944	6.715099824	2.01E-03
TEAD3	1.23E-02	0.118562975	0.037034566	0.379569163	3.29E-04
TDRD10	1.08E-03	0.171720499	0.054271004	0.543345945	2.72E-03
TDH	5.14E-03	5.927059568	1.783928919	19.69250834	3.67E-03
TCTN1	3.26E-10	0.074348774	0.025460246	0.217112605	2.00E-06
TCTE1	3.73E-06	2.13E-09	1.03E-14	0.000442575	1.39E-03
TCP11X2	5.28E-04	2.89E+124	1.30E+28	6.42E+220	1.13E-02
TCP10L	1.01E-03	2.39E-05	5.78E-08	0.00985199	5.34E-04
TCN1	3.55E-04	1.932689642	1.332340798	2.80355391	5.17E-04
TCL1B	5.53E-04	8.04E+39	94034183.9	6.88E+71	1.43E-02
TCL1A	3.42E-02	1182.699621	2.830257576	494223.0013	2.16E-02
TCIRG1	3.27E-05	3.883282871	2.117138813	7.122766709	1.17E-05
TCHH	3.51E-04	14.96909163	2.152723635	104.0884675	6.24E-03
TCF7L2	3.50E-02	0.193926017	0.070004016	0.537216328	1.60E-03
TCF19	2.15E-02	0.330671706	0.134988954	0.810020179	1.55E-02
TCF12	1.61E-02	1.82777524	1.106330464	3.019678511	1.86E-02
TCEB3B	2.12E-03	24094605.43	2497.330183	2.32468E+11	2.82E-04
TCEB2P1	3.25E-02	17147.706	52.14888571	5638544.663	9.77E-04
TCEB2	2.81E-02	4.41276532	1.660191175	11.72906956	2.92E-03
TCEB1P28	2.02E-02	13.77195091	2.061456829	92.00611392	6.80E-03
TCEB1P2	2.47E-03	80.18753322	4.813808111	1335.749231	2.25E-03
TCEB1	1.76E-05	2.925142296	1.810166177	4.726890582	1.17E-05
TCEAL3	1.07E-04	4.324976219	1.746359965	10.71109031	1.55E-03

TCEA1	1.55E-02	2.54226674	1.474271237	4.383942394	7.90E-04
TBXAS1	4.55E-03	3.03965116	1.431302608	6.455294024	3.81E-03
TBX3	1.05E-03	2.638267659	1.241020854	5.60865373	1.17E-02
TBX21	2.14E-03	3.776540366	1.552896647	9.184292569	3.38E-03
TBX20	7.66E-05	8735.972056	23.25490563	3281768.112	2.70E-03
TBX2-AS1	2.06E-02	0.393286728	0.206033715	0.750723976	4.67E-03
TBX2	1.93E-02	0.355140285	0.189081994	0.667036662	1.29E-03
TBX18	1.85E-02	8.452778654	2.172738654	32.88451966	2.07E-03
TBX1	1.64E-03	13.88459391	1.370745488	140.6402208	2.60E-02
TBL2	1.67E-04	7.958951863	2.146833129	29.50621261	1.92E-03
TBL1X	8.85E-03	0.311094123	0.132510705	0.730352716	7.33E-03
TBCB	2.55E-03	5.466343118	1.719151579	17.38119399	4.00E-03
TBC1D7	4.27E-02	2.586190084	1.26899558	5.270608704	8.90E-03
TBC1D5	1.64E-02	0.258634289	0.098499107	0.679109661	6.04E-03
TBC1D22B	1.83E-03	0.210947298	0.069217218	0.642885747	6.20E-03
TBC1D10C	1.51E-02	2.291802477	1.344183668	3.907470921	2.32E-03
TBC1D10A	1.18E-04	3.437551558	1.418341994	8.331390288	6.26E-03
TBC1D1	9.22E-04	3.674290518	1.80148561	7.494043103	3.45E-04
TATDN3	2.14E-02	2.029391012	1.031262702	3.993577847	4.05E-02
TATDN2	1.32E-06	0.034882965	0.006728497	0.180845914	6.42E-05
TAS1R1	7.29E-05	0.000948838	3.10E-06	0.290223196	1.71E-02
TARBP2	2.42E-02	3.922249975	1.162203403	13.23696423	2.77E-02
TAPBPL	2.39E-07	2.238507054	1.496366707	3.348720477	8.81E-05
TAP1	6.86E-03	1.48576181	1.182426065	1.866914324	6.78E-04
TAMM41	2.78E-04	0.049991791	0.00915976	0.27284331	5.40E-04
TAGLN2P1	2.14E-04	35.01344036	5.066056499	241.9911831	3.12E-04
TAGLN2	1.49E-03	4.596733472	2.161950932	9.773560673	7.39E-05
TAGAP	1.35E-02	3.145857392	1.302187063	7.599844148	1.09E-02
TAF5	1.27E-02	5.470670298	1.610131748	18.58744388	6.46E-03
TAF15	7.05E-03	0.153839284	0.04523484	0.523192421	2.72E-03
TADA3	1.20E-02	0.140592374	0.040089382	0.493053633	2.18E-03
TACR2	3.31E-02	0.320472207	0.117369388	0.875035961	2.64E-02
TACC2	4.67E-03	3.200298845	1.547321062	6.619125756	1.71E-03

SYVN1	1.03E-02	6.627099851	1.461201167	30.05640389	1.42E-02
SYPL2	4.64E-02	0.080353674	0.01250474	0.516341236	7.90E-03
SYNPR	5.83E-05	0.564414157	0.402384091	0.791689702	9.23E-04
SYNPO2L	6.44E-03	6.412739381	1.958009343	21.00256902	2.14E-03
SYNJ2	4.00E-02	2.393929371	1.493823145	3.836396464	2.86E-04
SYN3	2.34E-03	0.000576398	4.02E-06	0.08260751	3.24E-03
SYN2	1.08E-02	0.085210852	0.022259893	0.32618707	3.24E-04
SYMPK	1.87E-04	0.080647774	0.016617163	0.391406378	1.79E-03
SYBU	1.27E-02	0.479817698	0.270511241	0.851073776	1.20E-02
SVIL	2.05E-02	0.360436166	0.162576327	0.799096842	1.20E-02
SUSD2	1.12E-04	2.045957411	1.386276142	3.019558369	3.13E-04
SURF4	4.23E-03	3.423705941	1.457510724	8.042316381	4.73E-03
SUPT5H	8.71E-04	0.125583335	0.028324958	0.556794252	6.32E-03
SUPT3H	1.12E-02	0.368223684	0.161158039	0.841339861	1.78E-02
SUPT20HL2	3.06E-05	1.50E+85	6.30E+31	3.58E+138	1.76E-03
SUMF2	4.60E-04	3.222220701	1.612802361	6.437680462	9.21E-04
SUMF1	4.49E-05	0.113063549	0.036153994	0.353581023	1.79E-04
SULT2B1	2.05E-02	7.305403826	2.090487676	25.52941386	1.84E-03
SULT1C4	1.92E-02	0.506374327	0.29700133	0.863346166	1.24E-02
SULT1A1	4.39E-04	1.789968543	1.164722917	2.750858026	7.92E-03
SULF2	9.54E-08	2.277670487	1.57098752	3.302243194	1.40E-05
SUGT1P	1.74E-02	0.082854455	0.017456769	0.393249229	1.72E-03
SUGCT	1.07E-05	0.075358795	0.018504203	0.30690043	3.08E-04
SUCNR1	1.49E-04	10.94080191	1.613514876	74.18657759	1.43E-02
SUCLG2P4	2.76E-06	1.63E+60	3.4468E+19	7.67E+100	3.71E-03
STXBP5L	4.16E-04	2.45E-05	1.86E-09	0.323049525	2.83E-02
STX4	2.82E-03	8.152496514	2.601087351	25.55208282	3.18E-04
STX3	7.71E-03	2.19817712	1.147898996	4.209414475	1.75E-02
STX19	3.39E-02	6753.438748	6.241863853	7306941.644	1.34E-02
STX18-AS1	3.60E-02	0.006329519	0.000163729	0.244689599	6.63E-03
STX17-AS1	2.16E-03	0.221126835	0.086011795	0.568492695	1.73E-03
STT3A	2.48E-02	3.894035945	1.314892381	11.53213462	1.41E-02
STRADB	1.62E-04	2.864523781	1.530890519	5.359949905	9.94E-04

STRA13	1.52E-06	20.95270113	5.421954172	80.97001018	1.03E-05
STPG1	1.80E-05	0.057294967	0.015203949	0.215911885	2.39E-05
STOM	6.81E-03	1.934150005	1.345863244	2.779581252	3.63E-04
STK4	3.10E-02	3.530388355	1.349984243	9.232435123	1.01E-02
STK39	3.41E-02	1.629874708	1.010766762	2.62819442	4.51E-02
STK32B	6.25E-03	5.055419915	1.992191601	12.82872114	6.48E-04
STK19	1.27E-02	0.15823816	0.057282597	0.437119069	3.76E-04
STK17B	7.32E-03	2.836162968	1.531647171	5.251745004	9.12E-04
STK16	2.53E-03	7.477112431	1.446731365	38.64380884	1.64E-02
STK10	2.50E-02	2.300652841	1.151993203	4.594648196	1.82E-02
STIP1	4.23E-03	2.800425568	1.174765209	6.675702774	2.02E-02
STEAP3	3.19E-03	4.206162616	2.092921898	8.453160136	5.49E-05
STEAP1B	1.63E-02	0.096739788	0.016293087	0.574390038	1.02E-02
STEAP1	5.17E-03	0.229484001	0.084333236	0.624462059	3.95E-03
STAT6	2.50E-03	3.777635941	1.397291701	10.2129951	8.81E-03
STARD3	4.96E-04	4.947884867	1.371533471	17.84977558	1.46E-02
STAR	4.73E-04	0.003121679	9.88E-05	0.098666245	1.06E-03
STAP1	3.84E-05	49.92994842	4.48438175	555.9294208	1.47E-03
ST8SIA4	1.94E-02	4.662094315	1.696567126	12.81123692	2.84E-03
ST8SIA2	1.66E-06	6.590299552	2.49955727	17.37589641	1.38E-04
ST8SIA1	3.43E-04	5.840912817	1.403707268	24.30439972	1.53E-02
ST6GALNAC4	3.57E-03	2.912534412	1.543020893	5.497564382	9.73E-04
ST3GAL6-AS1	4.16E-03	0.50807316	0.318150848	0.811370885	4.58E-03
ST3GAL4-AS1	5.60E-04	3.65669789	1.704726888	7.843742921	8.69E-04
ST3GAL4	1.08E-04	3.504904934	1.628918838	7.541418462	1.34E-03
ST3GAL2	1.19E-02	3.798515329	1.017402318	14.18192041	4.71E-02
ST3GAL1	1.47E-03	2.660876178	1.509699655	4.689848085	7.13E-04
ST20-MTHFS	2.38E-03	48.6432698	4.535571646	521.6911741	1.33E-03
ST20	1.28E-03	4.53874951	1.87596715	10.98113425	7.92E-04
ST13P10	4.22E-02	2.06E-06	1.70E-11	0.249083164	2.83E-02
ST13	5.75E-03	0.178618939	0.063692565	0.500917576	1.06E-03
SSXP9	3.43E-03	180896.3068	21.1081687	1550275360	8.79E-03
SSXP4	1.06E-02	29772591.75	2.962441079	2.99215E+14	3.64E-02

SSXP3	6.68E-05	4.34E+22	1849528430	1.02E+36	9.05E-04
SSX5	2.85E-07	1.419373825	1.150079922	1.751723524	1.10E-03
SSX3	7.36E-03	11755.76909	3.871898492	35692595.58	2.20E-02
SSX1	5.26E-03	1.360295556	1.094333731	1.690895516	5.57E-03
SSUH2	1.02E-05	0.08116379	0.019342361	0.340576867	5.99E-04
SSTR2	4.66E-04	4.416905282	1.061984614	18.37037186	4.11E-02
SSR4	4.75E-02	2.874873428	1.099933177	7.513999396	3.12E-02
SSBP3-AS1	4.02E-03	16.44790279	4.718853293	57.33034902	1.11E-05
SSBP2	1.27E-04	0.097895706	0.031506095	0.304181441	5.88E-05
SSBP1	3.69E-03	17.56591905	4.410498664	69.96068602	4.81E-05
SRY	5.16E-03	1.52E+38	33038.98459	7.03E+71	2.62E-02
SRXN1	3.48E-03	11.45305404	2.669057145	49.14561203	1.03E-03
SRSF9	2.64E-04	3.222158214	1.165541823	8.907705711	2.41E-02
SRRT	7.87E-06	27.36440721	4.00467165	186.9843142	7.38E-04
SRPR	1.21E-02	5.464623784	1.461404238	20.43384871	1.16E-02
SRP54-AS1	7.44E-03	16.87277176	1.812239981	157.0931167	1.31E-02
SRP14	3.33E-03	3.907889414	1.433077088	10.65650955	7.75E-03
SRGAP3	1.08E-04	0.010307102	0.000717535	0.148057417	7.66E-04
SRGAP2D	7.03E-03	11.61025778	2.917341976	46.20578832	5.03E-04
SRGAP2B	1.04E-02	25.62808437	2.782874513	236.0144898	4.19E-03
SRGAP2	4.65E-05	3.919033692	1.803899172	8.514237001	5.60E-04
SRGAP1	1.00E-02	7.279907922	1.770176118	29.93886247	5.93E-03
SRF	4.36E-02	0.369083316	0.14006571	0.972561333	4.38E-02
SRD5A3-AS1	3.02E-02	37.8616893	2.128409033	673.5112914	1.34E-02
SRD5A3	3.52E-05	2.620827217	1.58163711	4.342801049	1.85E-04
SRCIN1	4.22E-05	3.772303654	1.575871916	9.030096111	2.87E-03
SRA1	5.22E-04	8.702616678	3.091445604	24.49842136	4.18E-05
SQRDL	1.08E-02	2.279432956	1.42344291	3.650174211	6.04E-04
SQLE	2.02E-03	2.000467246	1.425076543	2.808178425	6.15E-05
SPTLC1P1	3.16E-02	355011.6817	39.43135253	3196271141	5.94E-03
SPTBN1	1.25E-02	1.744993505	1.059536309	2.873900883	2.87E-02
SPSB3	1.14E-03	0.014846451	0.001631646	0.135088821	1.86E-04
SPRYD4	2.88E-04	14.38911194	3.537351293	58.53151846	1.96E-04

SPRY4	5.75E-03	2.584048637	1.353965279	4.931668091	3.99E-03
SPRY3	4.36E-02	168.737237	6.426881962	4430.181749	2.10E-03
SPRY1	1.51E-02	0.62692356	0.421624114	0.9321885	2.11E-02
SPRR4	4.58E-06	1.70906E+15	460811.3826	6.34E+24	1.81E-03
SPRN	4.82E-03	8.804965829	2.175952676	35.6291863	2.29E-03
SPRED3	3.07E-03	7.390656602	1.028332355	53.11687876	4.68E-02
SPPL2A	4.33E-02	2.401684166	1.243529319	4.638480767	9.08E-03
SPP1	3.04E-02	0.798682391	0.680996086	0.93670665	5.71E-03
SPON2	5.18E-06	2.442152246	1.61955744	3.682553916	2.04E-05
SPO11	5.28E-04	1.85E+41	2096198468	1.63E+73	1.13E-02
SPN	2.89E-05	2.333372915	1.355131158	4.017787597	2.24E-03
SPINT1	6.81E-03	0.637020438	0.439007129	0.924347263	1.76E-02
SPIN2A	1.21E-02	630.3894931	3.290432227	120771.6451	1.62E-02
SPIDR	2.80E-03	2.696186135	1.530069816	4.751037892	6.00E-04
SPIB	1.90E-03	21.47661571	3.417688751	134.9581708	1.07E-03
SPHK1	3.35E-03	2.03787598	1.353082782	3.069242003	6.56E-04
SPG20-AS1	1.32E-02	0.306664078	0.12267502	0.766601521	1.15E-02
SPEF1	2.31E-06	0.003999153	0.000138075	0.11582969	1.30E-03
SPECC1	3.53E-04	2.690768265	1.107816737	6.535588071	2.88E-02
SPDYE6	1.62E-02	0.000205742	3.08E-07	0.137600948	1.05E-02
SPCS2P4	1.18E-04	6.885217969	2.331196868	20.33557402	4.80E-04
SPCS2	2.02E-04	7.538682415	2.539182759	22.3818992	2.74E-04
SPATA9	3.86E-02	3.302829043	1.196531686	9.116916684	2.11E-02
SPATA45	1.24E-02	56.02597613	1.856841488	1690.456628	2.06E-02
SPATA32	1.49E-03	691997.2562	138.5315715	3456686425	1.97E-03
SPATA31A7	7.79E-04	9.02E-86	1.70E-165	4.79E-06	3.66E-02
SPATA31A1	4.44E-03	1.79E-13	3.90E-25	0.082450288	3.22E-02
SPATA24	1.44E-04	17.38822828	4.612830344	65.54554584	2.46E-05
SPATA20	1.25E-02	2.508487242	1.164028993	5.405800269	1.89E-02
SPATA13	4.24E-02	3.550767551	1.539008807	8.192253445	2.97E-03
SPARCL1	1.84E-03	1.320691676	1.072681895	1.626042642	8.76E-03
SPARC	1.87E-04	2.917683711	1.638219048	5.196422449	2.77E-04
SPANXN2	1.99E-03	1.29E+71	1.87688E+12	8.93E+129	1.78E-02

SPANXD	7.23E-05	2.61E+29	147966559.4	4.59E+50	6.65E-03
SPANXA2-OT1	4.94E-03	1.2306E+11	2354.853874	6.43091E+18	4.86E-03
SPAG8	1.23E-05	0.001331769	5.49E-05	0.032279739	4.69E-05
SPAG7	1.24E-04	8.786919539	2.425405323	31.83383588	9.36E-04
SPAG5-AS1	2.14E-02	15.20173551	1.027027893	225.0111842	4.78E-02
SPAG16	3.64E-05	0.056915449	0.013309284	0.243391631	1.11E-04
SPAG1	7.32E-05	22.09524844	5.781394173	84.4433002	6.04E-06
SP7	1.57E-04	1.97E-14	4.32E-25	0.000894773	1.17E-02
SP140	3.52E-03	5.242977833	1.866719846	14.7257322	1.66E-03
SP110	1.35E-02	4.011939581	1.424741256	11.29725074	8.54E-03
SOX17	4.40E-04	24.78283555	3.258442134	188.4915897	1.93E-03
SOX15	9.46E-05	0.271206112	0.127395328	0.577358341	7.12E-04
SOX13	1.95E-02	3.524458118	1.500316318	8.27945739	3.84E-03
SOX1	1.13E-04	2.674295226	1.565399178	4.568710048	3.18E-04
SOWAHD	1.01E-02	3.419658219	1.327950308	8.806099342	1.08E-02
SOS1-IT1	2.85E-03	2.455161044	1.441520948	4.181566533	9.46E-04
SOS1	3.78E-02	1.89125468	1.123213462	3.184474176	1.65E-02
SORL1	1.14E-03	2.160797635	1.382156866	3.378087202	7.26E-04
SORD	4.03E-02	2.253270659	1.110824706	4.570683957	2.44E-02
SORBS2	2.15E-03	0.147603093	0.051655276	0.421770532	3.55E-04
SOD1	4.63E-02	5.734864866	2.066892445	15.91213665	7.95E-04
SOCS3	1.52E-02	1.535873129	1.046631929	2.253806904	2.83E-02
SOCS2-AS1	5.18E-03	4.834458205	1.767283326	13.22480996	2.15E-03
SOCS2	4.24E-06	2.720370806	1.61805504	4.573649931	1.60E-04
SOBP	1.63E-09	0.192932128	0.094894415	0.392254971	5.50E-06
SOAT1	4.64E-03	2.241922824	1.388734655	3.619278838	9.54E-04
SNX33P1	5.16E-03	1.69E+79	2381745179	1.20E+149	2.62E-02
SNX32	3.22E-02	2.153524076	1.112665298	4.168069188	2.28E-02
SNX29	1.10E-03	0.072974042	0.018340078	0.290359224	2.03E-04
SNX25P1	4.59E-02	5.100001613	1.427911987	18.21541992	1.21E-02
SNX25	2.13E-02	1.834408203	1.247724912	2.696951405	2.03E-03
SNX22	3.70E-04	2.16572275	1.226238129	3.824995259	7.75E-03
SNX21	3.15E-03	3.263360383	1.428682805	7.454083545	5.01E-03

SNX18P8	5.16E-03	3.03E+39	47060.69653	1.95E+74	2.62E-02
SNX1	2.03E-02	2.52254783	1.118751081	5.687813547	2.57E-02
SNW1	2.78E-02	3.033625771	1.106164195	8.31963768	3.11E-02
SNURF	3.22E-02	10.44309589	1.264621415	86.23786561	2.94E-02
SNUPN	1.18E-03	8.639517687	2.742920185	27.21233606	2.30E-04
SNTB2	1.47E-02	2.059670336	1.058216005	4.008861966	3.35E-02
SNRPGP2	1.61E-03	6.676653923	3.013153374	14.79437057	2.91E-06
SNRPGP10	1.45E-02	9.590782646	2.55587904	35.98883606	8.06E-04
SNRPG	4.51E-06	9.740676395	3.744316517	25.33994554	3.06E-06
SNRPD3	1.93E-02	9.20605852	2.251929491	37.63506532	2.00E-03
SNRPD1	4.50E-02	3.375331533	1.084885183	10.5014458	3.57E-02
SNRPB	6.50E-04	5.074760369	2.021682026	12.73849818	5.42E-04
SNRNP35	2.64E-02	9.480758002	2.345980113	38.31437948	1.60E-03
SNRNP25	1.58E-03	6.676112594	1.890303586	23.57847686	3.19E-03
snoZ196	2.51E-02	3.399717851	1.442414641	8.013008981	5.15E-03
snoU109	1.09E-02	4.829943885	2.210426955	10.55377916	7.85E-05
SNORD88A	2.61E-02	3.896863291	1.054186957	14.40498141	4.14E-02
SNORD53	3.02E-02	44.4388186	1.060646145	1861.892025	4.65E-02
SNORD45	5.16E-03	6259.853835	2.814051935	13925034.41	2.62E-02
SNORD116-6	1.01E-02	5.079095158	1.049273283	24.58578528	4.34E-02
SNORD11	2.56E-02	13.29207366	2.045030484	86.39441975	6.75E-03
SNORA71D	3.96E-02	75.20242551	3.065929831	1844.596946	8.14E-03
SNORA70F	9.85E-03	2.77E-05	1.60E-09	0.481747646	3.52E-02
SNORA36	2.28E-02	16.61360031	1.092448851	252.6541311	4.30E-02
SNORA14A	1.49E-03	6.965948175	1.421360946	34.13941696	1.67E-02
SNHG8	1.96E-04	0.406241236	0.254738297	0.647848963	1.55E-04
SNHG7	2.48E-04	0.437941717	0.285476489	0.671834474	1.56E-04
SNHG6	7.20E-03	2.388745156	1.158001581	4.927543723	1.84E-02
SNHG19	9.16E-03	0.481584304	0.284307875	0.815747514	6.58E-03
SNHG18	1.25E-05	0.531940759	0.357026395	0.792549164	1.92E-03
SNHG17	3.29E-03	0.407926614	0.186455473	0.89246038	2.48E-02
SNHG16	4.18E-04	0.145446254	0.055745466	0.3794858	8.14E-05
SNHG15	2.22E-02	3.880751138	1.381556388	10.90091546	1.01E-02

SNHG11	2.67E-03	3.355116582	1.199895873	9.381486786	2.10E-02
SNHG1	2.39E-02	0.445000667	0.247790606	0.799165058	6.72E-03
SNF8	1.84E-04	10.81498014	3.221138956	36.31131625	1.17E-04
SNCAIP	4.97E-06	27933604360	94482.37333	8.25854E+15	1.82E-04
SNCA	6.56E-03	0.730231962	0.537741573	0.991626361	4.40E-02
SNAPIN	2.58E-03	17.39828255	3.929343354	77.0358323	1.68E-04
SNAPC5	2.40E-02	4.540363477	1.765470588	11.67671704	1.69E-03
SNAPC4	5.40E-05	0.031695969	0.005519966	0.182000124	1.09E-04
SNAPC3	4.27E-02	3.007650113	1.273216441	7.104808666	1.20E-02
SMYD3	2.42E-04	18.72666197	4.230316867	82.89872357	1.13E-04
SMOC2	4.96E-03	1.72970114	1.131059664	2.645188515	1.15E-02
SMN1	2.77E-02	12.81259952	2.551201362	64.3472165	1.95E-03
SMIM9	3.05E-02	4.45557E+15	41.34084859	4.80E+29	2.88E-02
SMIM7	4.72E-04	31.19750026	4.978819601	195.4848941	2.38E-04
SMIM13	8.71E-03	0.409075549	0.193358618	0.865453046	1.94E-02
SMCO4	9.00E-03	2.60498187	1.189722047	5.7037949	1.66E-02
SMCO3	1.32E-02	0.156800573	0.032814597	0.749252529	2.02E-02
SMCO2	7.38E-04	2.25E+49	5.00798E+17	1.01E+81	2.24E-03
SMARCE1P6	2.36E-02	2161835094	65.39215769	7.14693E+16	1.50E-02
SMARCD3	4.82E-06	12.07044383	4.115886952	35.39835179	5.70E-06
SMARCC2	4.24E-02	0.328591484	0.140979358	0.765873561	9.94E-03
SMARCC1	4.81E-03	0.275252159	0.12126276	0.624789927	2.04E-03
SMAD9	2.12E-03	3.394832334	1.234096273	9.338725691	1.79E-02
SLMO2P1	2.36E-02	128246404.2	1.958662117	8.39713E+15	4.20E-02
SLIT1	1.76E-04	1.734777866	1.127234135	2.669768551	1.23E-02
SLIRP	3.21E-05	13.22146902	4.029077511	43.38641853	2.06E-05
SLFN11	3.67E-03	2.437146582	1.406414648	4.223280433	1.49E-03
SLCO5A1	5.65E-04	2.820189263	1.82611713	4.355398321	2.93E-06
SLCO4A1-AS1	5.20E-03	0.577665023	0.388575119	0.858770575	6.68E-03
SLCO3A1	6.76E-07	7.098923357	2.701656193	18.65326646	7.00E-05
SLCO1B1	5.16E-03	2.01E+25	987.6546551	4.08E+47	2.62E-02
SLC9A9-AS2	2.60E-02	11243963.14	2.313809062	5.46401E+13	3.88E-02
SLC9A3R1	3.41E-05	0.265028816	0.125866171	0.558055216	4.74E-04

SLC8B1	1.17E-02	9.135949036	2.340540077	35.66081419	1.45E-03
SLC7A7	8.56E-04	3.180898689	1.700080114	5.951552746	2.94E-04
SLC7A6	3.16E-02	8.23339766	2.599559239	26.07705029	3.38E-04
SLC7A3	2.88E-03	34.1522048	1.214492978	960.3786215	3.81E-02
SLC7A2	2.82E-03	0.297480702	0.142409231	0.621411741	1.26E-03
SLC6A8	8.68E-03	0.28435445	0.110755203	0.730055573	8.95E-03
SLC6A6	2.77E-03	0.301478253	0.161737181	0.561955738	1.61E-04
SLC6A3	2.82E-05	9.926688741	2.929367483	33.63837071	2.28E-04
SLC6A16	3.55E-03	0.102831694	0.024063419	0.439437017	2.14E-03
SLC6A15	3.38E-03	0.545658459	0.36610124	0.813280921	2.93E-03
SLC5A6	9.14E-03	4.612970224	1.912732644	11.12517965	6.64E-04
SLC5A2	1.88E-02	1.41E-12	8.64E-21	0.000228787	4.67E-03
SLC52A2	3.55E-03	2.928619321	1.664278444	5.153471257	1.94E-04
SLC52A1	4.15E-03	378.5554795	17.14844843	8356.68904	1.70E-04
SLC51B	2.93E-02	138.1838413	3.875360984	4927.224608	6.87E-03
SLC50A1	1.87E-03	5.865269296	2.449386108	14.04490039	7.17E-05
SLC4A2	1.00E-03	2.46055756	1.174874739	5.153182128	1.70E-02
SLC4A1APP1	1.51E-03	10895.26763	19.37865998	6125648.356	4.01E-03
SLC4A11	5.67E-04	3.274943046	1.878635027	5.709066317	2.87E-05
SLC47A1	8.94E-07	4.672091021	2.21248721	9.866016134	5.30E-05
SLC46A1	2.93E-07	18.87882918	4.963954056	71.7996555	1.63E-05
SLC45A4	6.95E-04	2.8661248	1.326304131	6.193655872	7.40E-03
SLC45A3	1.41E-04	5.815451194	2.167117968	15.60573678	4.73E-04
SLC45A2	7.08E-07	3.025624994	1.855681021	4.933178978	9.05E-06
SLC44A3	6.34E-07	0.331299512	0.209866447	0.522996258	2.11E-06
SLC41A3	7.41E-07	0.143503367	0.056515372	0.364382565	4.44E-05
SLC41A1	6.75E-03	2.737205475	1.115397137	6.717153527	2.79E-02
SLC3A1	2.54E-03	0.003722086	2.17E-05	0.637463799	3.30E-02
SLC39A9	5.27E-04	7.688844706	2.533682058	23.33297215	3.17E-04
SLC39A8	3.77E-02	3.869852511	1.326333156	11.29109861	1.33E-02
SLC39A4	6.08E-03	3.57091799	1.880860205	6.779586944	9.97E-05
SLC39A13	6.87E-03	3.882923291	1.134619982	13.28823176	3.07E-02
SLC39A11	2.43E-04	4.794351372	2.141292647	10.73454631	1.38E-04

SLC39A1	9.05E-04	5.131068092	2.107756832	12.49093793	3.15E-04
SLC38A9	1.55E-02	7.336064221	1.965226764	27.38505259	3.02E-03
SLC38A8	1.80E-06	1.855274337	1.210063711	2.844513751	4.59E-03
SLC38A7	1.82E-04	4.759665932	1.826171996	12.40541407	1.41E-03
SLC38A6	8.44E-05	3.899209515	2.150104048	7.071208882	7.45E-06
SLC38A5	1.30E-02	1.313588263	1.069218037	1.613809406	9.40E-03
SLC36A2	9.41E-03	2.31719E+17	320439.3955	1.68E+29	4.11E-03
SLC36A1	4.23E-05	10.72968366	3.159542588	36.43758809	1.42E-04
SLC35F6	1.20E-04	4.22484799	2.096639568	8.513309019	5.56E-05
SLC35F2	1.16E-02	0.40058718	0.193997192	0.827177381	1.34E-02
SLC35E3	1.70E-02	6.34344872	1.677807056	23.98329505	6.48E-03
SLC35B1	3.18E-04	25.07238435	4.105739462	153.1087062	4.83E-04
SLC35A4	3.58E-02	3.988477992	1.258224192	12.64318139	1.88E-02
SLC35A2	7.44E-04	4.077653034	1.289322914	12.89611322	1.67E-02
SLC31A2	2.93E-02	10.73597891	1.24167995	92.82685383	3.10E-02
SLC30A2	4.53E-02	1.6123512	1.024409999	2.537730397	3.90E-02
SLC2A6	1.08E-04	2.350988869	1.269491591	4.353828495	6.55E-03
SLC2A3P4	8.97E-04	78034358.16	48.81299707	1.24749E+14	1.27E-02
SLC2A3	1.39E-03	1.795453038	1.21928458	2.643887789	3.04E-03
SLC2A13P1	3.57E-06	1.53922E+15	3150531.039	7.52E+23	6.13E-04
SLC2A10	4.45E-03	2.007549562	1.267060228	3.180792166	3.00E-03
SLC29A4	4.05E-03	2.399522243	1.340318923	4.295773859	3.22E-03
SLC29A2	3.04E-02	0.409775247	0.17589311	0.954646564	3.87E-02
SLC27A5	5.16E-03	0.143441598	0.037581742	0.547486386	4.49E-03
SLC27A3	1.62E-02	4.49811367	1.793651309	11.28035671	1.35E-03
SLC26A11	2.59E-02	3.938953055	1.268263408	12.23354003	1.77E-02
SLC26A10	1.35E-02	1858.77545	3.196862196	1080761.685	2.05E-02
SLC25A6	1.27E-02	0.377555466	0.198723886	0.717317545	2.93E-03
SLC25A51P3	1.17E-02	2.03E+22	835.3733033	4.94E+41	2.41E-02
SLC25A47	6.13E-03	2006.308479	14.59799569	275741.533	2.47E-03
SLC25A45	2.47E-03	3.369824144	1.30855417	8.678062418	1.18E-02
SLC25A3P1	4.10E-02	1.39E+67	71319.13377	2.70E+129	3.46E-02
SLC25A38	5.32E-07	0.291308472	0.183314346	0.462924086	1.80E-07

SLC25A32	3.44E-03	2.233650921	1.363751524	3.658435096	1.41E-03
SLC25A30	1.37E-02	0.402115979	0.205679055	0.786162989	7.74E-03
SLC25A29	4.82E-02	1.841573015	1.008025933	3.364388809	4.70E-02
SLC25A26	4.86E-06	0.04880672	0.011549917	0.206243557	4.01E-05
SLC25A21-AS1	1.23E-03	0.174006807	0.058463813	0.51789932	1.68E-03
SLC25A21	2.19E-02	0.005329735	9.26E-05	0.306672976	1.14E-02
SLC25A19	1.75E-03	12.06355378	2.730406824	53.29950411	1.02E-03
SLC25A17	1.09E-03	4.691757395	1.317231591	16.71125078	1.71E-02
SLC25A15P3	4.96E-02	4.36477366	1.593945613	11.95225794	4.14E-03
SLC25A15P2	3.02E-02	42.11551821	2.293840249	773.252137	1.18E-02
SLC25A15P1	3.31E-02	4464491877	2.586440432	7.70622E+18	4.06E-02
SLC23A2	2.23E-02	1.77037654	1.033833577	3.031661152	3.74E-02
SLC22A9	2.17E-03	1.77E-06	3.83E-11	0.082030671	1.57E-02
SLC22A4	2.29E-03	2.789980789	1.085961138	7.167837351	3.31E-02
SLC22A31	5.66E-05	2.22327621	1.212613891	4.076282766	9.79E-03
SLC22A18	8.10E-03	2.733331767	1.587184023	4.707143242	2.88E-04
SLC22A17	1.47E-03	1.702145631	1.182847765	2.449427418	4.18E-03
SLC1A5	9.01E-03	2.635639913	1.400246712	4.960981298	2.67E-03
SLC1A4	9.64E-04	1.862461343	1.236252633	2.805868447	2.94E-03
SLC1A1	8.06E-06	2.730950655	1.688969217	4.415765193	4.17E-05
SLC19A3	1.97E-02	0.151648919	0.029076742	0.790920604	2.52E-02
SLC19A1	4.23E-02	2.346546357	1.169352785	4.708826863	1.64E-02
SLC18A2	4.53E-02	440.054433	1.009306113	191862.4108	4.97E-02
SLC17A9	6.44E-03	3.183940575	1.729616994	5.861111229	1.99E-04
SLC17A5	2.07E-02	1.60989005	1.047176266	2.474985402	3.00E-02
SLC17A3	2.07E-03	4.06E+73	1.13295E+18	1.45E+129	9.41E-03
SLC16A8	6.22E-04	2.167709024	1.407467989	3.338592742	4.46E-04
SLC16A6	6.71E-05	1.752061389	1.305114022	2.352069673	1.90E-04
SLC16A14P1	3.57E-06	3.54E+51	1.14E+22	1.10E+81	6.13E-04
SLC12A9	4.79E-05	24.75141741	5.662223634	108.1964796	2.01E-05
SLC12A3	3.28E-03	2.03509E+12	10582.90349	3.91E+20	3.59E-03
SLC11A2	4.76E-03	3.094601274	1.570769273	6.096730571	1.09E-03
SLC10A5P1	4.27E-02	3.10539E+15	2.678857304	3.60E+30	4.38E-02

SLAMF8	2.21E-05	2.007714576	1.375299027	2.930939193	3.05E-04
SLAMF6	6.17E-05	2.254313398	1.355085369	3.750264754	1.75E-03
SLAMF1	6.02E-04	4.833374627	1.764790383	13.2375553	2.18E-03
SLA2	1.26E-04	2.448046167	1.397085556	4.289594155	1.76E-03
SLA	1.92E-02	2.306959811	1.230867085	4.323832878	9.11E-03
SKIV2L	1.02E-02	2.315662925	1.079656755	4.966666262	3.10E-02
SKI	6.81E-04	0.266944606	0.113314799	0.628862454	2.52E-03
SKAP1	1.78E-03	2.956996067	1.441833358	6.064380247	3.09E-03
SIX4	6.75E-04	61.25237758	5.333701039	703.4240823	9.53E-04
SIX2	4.95E-04	3.750758101	1.261898617	11.14842836	1.74E-02
SIVA1	2.03E-03	5.383165304	1.669497188	17.35760258	4.83E-03
SIT1	3.96E-04	1.805779727	1.265966656	2.575771175	1.11E-03
SIRT3	1.93E-06	0.012583209	0.00276311	0.057303969	1.54E-08
SIRT2	7.65E-03	0.106278875	0.02075842	0.544126152	7.14E-03
SIRPG-AS1	1.72E-05	11781.17776	5.273425286	26319923.37	1.72E-02
SIRPG	6.41E-04	1.862267718	1.26192679	2.748210974	1.74E-03
SIRPD	2.39E-02	6845383861	3.085840242	1.51853E+19	3.92E-02
SIRPA	2.26E-03	0.258273122	0.094152492	0.708478383	8.55E-03
SIPA1L2	1.76E-05	2.352764043	1.469570503	3.766745884	3.66E-04
SIPA1	2.54E-02	2.19156303	1.121310758	4.283334018	2.17E-02
SIL1	6.27E-04	3.115079781	1.638001519	5.924122735	5.31E-04
SIK2	3.49E-02	0.398588944	0.172467711	0.921176174	3.14E-02
SIGLEC9	5.26E-03	3.722720323	1.315625208	10.53388649	1.33E-02
SIGLEC8	9.41E-03	1.860949641	1.146934051	3.01947053	1.19E-02
SIGLEC7	9.49E-03	3.851055941	1.218224058	12.17397716	2.17E-02
SIGLEC29P	5.16E-03	1.67E+50	879192.4813	3.19E+94	2.62E-02
SIGLEC19P	6.16E-04	4217.916441	5.099062465	3489037.293	1.49E-02
SIGLEC12	6.78E-05	7.440829517	2.817072448	19.65371673	5.12E-05
SIGLEC10	5.09E-03	2.143071801	1.339744714	3.428083496	1.47E-03
SIGLEC1	3.58E-02	1.898432591	1.099011174	3.279353647	2.15E-02
SIAH2-AS1	1.40E-03	0.014894228	0.00086991	0.255012591	3.70E-03
SIAH2	8.92E-04	0.094286426	0.02811596	0.316188029	1.31E-04
SHROOM4	7.75E-03	5.900584432	1.725712228	20.17537807	4.66E-03

SHQ1	3.49E-02	0.3407939	0.116975038	0.992865523	4.85E-02
SHPK	4.57E-05	4.269403155	1.776463548	10.26072464	1.18E-03
SHMT1P1	2.35E-02	7848755676	15995.41504	3.85129E+15	6.55E-04
SHMT1	2.24E-02	1.733529602	1.07396604	2.798156338	2.43E-02
SHKBP1	1.05E-04	3.203554433	1.455022604	7.053334415	3.84E-03
SHISA3	1.80E-02	88.74841001	2.85864941	2755.245275	1.05E-02
SHISA2	6.22E-03	15.01645008	2.152722175	104.7482001	6.26E-03
SHFM1	1.23E-05	23.8437651	5.884438397	96.61502009	8.89E-06
SHE	7.01E-05	0.168747039	0.057900217	0.491804087	1.11E-03
SHC4	2.13E-03	0.309108117	0.14002338	0.682370531	3.66E-03
SHC1	1.38E-04	4.46499807	2.115508974	9.423835123	8.64E-05
SH3RF3	3.33E-02	2.579063007	1.332517251	4.991729742	4.92E-03
SH3RF2	1.37E-02	100.4770011	4.637239839	2177.076905	3.31E-03
SH3PXD2B	7.99E-05	4.577700791	2.159675912	9.703004244	7.22E-05
SH3PXD2A-AS1	1.20E-02	2.89089936	1.595827677	5.236968394	4.62E-04
SH3PXD2A	3.67E-05	3.498004687	1.751426383	6.986326637	3.88E-04
SH3KBP1	1.73E-02	3.156494585	1.479562109	6.734058682	2.95E-03
SH3GLB2	1.28E-02	0.097474497	0.024445496	0.388671899	9.70E-04
SH3D19	1.01E-03	0.295866452	0.162181311	0.5397475	7.18E-05
SH3BP2	1.70E-02	8.280189572	2.291428625	29.92087059	1.26E-03
SH2D5	1.56E-04	4.16892046	2.016225904	8.620015129	1.17E-04
SH2D4B	1.16E-03	2.16E-18	5.67E-30	8.24E-07	2.79E-03
SH2D3C	8.16E-03	4.129932958	1.808759625	9.429857901	7.60E-04
SH2D2A	1.88E-02	2.987861015	1.569450807	5.688176659	8.62E-04
SH2D1A	4.97E-04	2.057571367	1.199580141	3.529234758	8.77E-03
SH2B2	4.32E-03	5.289722747	1.959989243	14.27618383	1.01E-03
SGSM2	2.75E-06	2.585630943	1.575186469	4.244251397	1.72E-04
SGSM1	6.64E-05	4.560127212	1.73375382	11.99406741	2.10E-03
SGOL1P2	5.28E-04	8.83E+69	6.30494E+15	1.24E+124	1.13E-02
SGK1	7.00E-05	1.422893672	1.081227465	1.872525871	1.18E-02
SGCD	1.42E-02	2.486669042	1.370422254	4.512129676	2.73E-03
SGCA	4.93E-02	0.271678305	0.100518616	0.734282908	1.02E-02
SFXN5	1.24E-05	3.177140649	1.632679053	6.182612976	6.66E-04

SFXN4	1.83E-03	4.085280687	1.450233366	11.50816047	7.73E-03
SFXN3	5.06E-06	5.245919633	2.397416979	11.47888458	3.35E-05
SFXN1	3.40E-02	2.901911264	1.473276719	5.715890894	2.07E-03
SFTA1P	3.09E-04	23.04638519	5.286372618	100.4726509	2.96E-05
SFT2D2	2.43E-03	3.104967414	1.435781775	6.714685206	3.99E-03
SFMBT1	5.11E-03	0.080640193	0.019634412	0.331196097	4.77E-04
SETP12	4.56E-03	9.07E-08	1.72E-13	0.047805219	1.59E-02
SETMAR	3.08E-04	0.397244138	0.187616054	0.841094893	1.59E-02
SETD4	2.46E-02	4.243408022	1.141503498	15.77438148	3.10E-02
SETBP1	8.43E-04	18.67940668	3.934088205	88.69151268	2.30E-04
SERPINE1	2.99E-03	2.180509845	1.463803314	3.248129814	1.26E-04
SERPINB9	2.61E-04	0.331005886	0.203739918	0.537768435	8.00E-06
SERPINB8	2.26E-04	6.218488816	2.527826648	15.29756923	6.92E-05
SERPINB6	5.25E-03	0.111864689	0.033513677	0.373391101	3.68E-04
SERPINB2	3.71E-05	7547060.071	247.3399813	2.30283E+11	2.65E-03
SERPINB10	8.25E-03	7.94E+48	1163676.988	5.41E+91	2.53E-02
SERINC5	4.91E-02	2.67427409	1.460953991	4.895254713	1.43E-03
SEPW1	4.30E-03	3.923199935	1.518477696	10.13613685	4.77E-03
7-Sep	1.40E-02	2.136387708	1.062949711	4.29385548	3.31E-02
3-Sep	1.69E-02	3.053376119	1.652231585	5.642735441	3.67E-04
2-Sep	1.75E-02	1.851934411	1.172150648	2.925955864	8.28E-03
SEPSECS-AS1	4.58E-03	7.358898699	1.566052348	34.57955293	1.15E-02
SEPHS2	1.21E-02	1.974350344	1.129507312	3.451114692	1.70E-02
SENP2	7.10E-03	0.175350275	0.061229303	0.502173267	1.18E-03
SEMA6B	3.22E-03	2.307774448	1.390207692	3.830954851	1.22E-03
SEMA6A	3.24E-03	0.436656819	0.291278525	0.654594012	6.04E-05
SEMA4D	4.31E-03	3.299604945	1.035810542	10.51098859	4.34E-02
SEMA4C	5.52E-03	0.230503428	0.103004794	0.515819005	3.56E-04
SEMA3E	1.77E-02	7.091507362	1.809504166	27.7918546	4.94E-03
SEMA3C	1.27E-02	0.44594451	0.24683301	0.805672248	7.45E-03
SEMA3B	1.82E-05	0.33621562	0.198144964	0.570496171	5.34E-05
SELPLG	2.78E-02	1.762687287	1.143540882	2.717057622	1.02E-02
SEL1L3	3.70E-05	1.993560432	1.376155149	2.887961579	2.64E-04

SEL1L2	3.82E-03	1.01E-12	5.13E-22	0.001980862	1.14E-02
SECTM1	1.08E-02	1.714204807	1.263388698	2.325886028	5.37E-04
SEC61G	2.65E-04	6.277223654	2.057226095	19.15372204	1.25E-03
SEC24D	9.43E-04	3.791053934	1.949703244	7.371424332	8.57E-05
SEC22C	1.58E-02	0.337259449	0.156093334	0.728691821	5.69E-03
SEC14L5	2.12E-03	0.442142217	0.245715113	0.7955951	6.47E-03
SEC14L4	1.27E-02	4.623603308	1.280145469	16.69943617	1.94E-02
SEC13	1.01E-02	0.241126681	0.072110652	0.806289703	2.09E-02
SDS	2.26E-03	2.226065457	1.436532761	3.449533176	3.42E-04
SDR42E1	2.34E-02	0.022919591	0.000646321	0.812766197	3.81E-02
SDR16C6P	1.06E-02	9.87E+31	104.487991	9.33E+61	3.64E-02
SDK2	2.99E-04	3.288759944	1.537628989	7.034168871	2.15E-03
SDHAF2	1.87E-02	11.06786642	2.183119075	56.11130811	3.70E-03
SDHAF1	1.32E-02	0.086707077	0.014601528	0.514885653	7.14E-03
SDHA	2.59E-05	5.6932193	2.236198487	14.49457469	2.64E-04
SDF2L1	1.04E-03	5.915179261	2.685630149	13.02835601	1.02E-05
SDCBP	2.26E-04	2.718179215	1.549483932	4.768360672	4.88E-04
SDC4P	1.99E-03	1.71E+31	245877.2047	1.19E+57	1.78E-02
SDC4	1.24E-02	0.326671694	0.149384454	0.714360785	5.07E-03
SDC3	2.82E-02	0.476984199	0.272266256	0.835630274	9.66E-03
SDC2	7.54E-04	1.747468326	1.27337276	2.398076702	5.47E-04
SCX	1.75E-03	2.623361515	1.690795423	4.07028878	1.68E-05
SCT	4.20E-02	0.283703775	0.089543496	0.898868543	3.23E-02
SCRN2	2.01E-04	0.295440225	0.133077025	0.655897791	2.73E-03
SCRN1	3.01E-02	0.523977513	0.33036721	0.831052313	6.03E-03
SCO2	7.65E-05	4.871082248	2.013041433	11.78686234	4.45E-04
SCNM1	2.90E-02	7.814429914	1.997595573	30.56940839	3.13E-03
SCN8A	3.61E-04	0.012211319	0.000530379	0.281150404	5.91E-03
SCN4B	7.13E-06	2.1623726	1.289514911	3.626057536	3.46E-03
SCN4A	1.74E-02	38.5309442	1.125539508	1319.041802	4.28E-02
SCN1B	3.95E-05	1.977137782	1.31520327	2.972220263	1.05E-03
SCN11A	4.81E-02	2.05E-10	7.67E-19	0.055003873	2.43E-02
SCML4	6.01E-04	87.15601842	3.499423769	2170.692105	6.46E-03

SCMH1	8.80E-03	0.321670663	0.119228956	0.867843006	2.51E-02
SCLY	1.94E-05	775.6582158	31.16423001	19305.64842	4.97E-05
SCIMP	6.88E-03	3.333766934	1.41118237	7.875666683	6.05E-03
SCGB1B2P	1.11E-06	0.110465917	0.033517845	0.364066334	2.94E-04
SCD5	3.62E-05	1.407795156	1.057179793	1.874692664	1.93E-02
SCCPDH	3.85E-04	1.837517788	1.203421573	2.805726354	4.84E-03
SCARNA21	2.04E-03	4.575239418	1.692513809	12.36788476	2.73E-03
SCARB1	8.77E-03	4.210795307	1.663682868	10.65755828	2.41E-03
SCAP	2.90E-06	0.124568534	0.048838616	0.317726439	1.30E-05
SCAMP4	3.69E-03	3.592776831	1.49347044	8.642986841	4.30E-03
SATB2	2.39E-02	5.716153148	1.748232652	18.68996485	3.93E-03
SATB1-AS1	1.16E-04	3.99E-05	2.66E-08	0.059785692	6.63E-03
SATB1	4.66E-06	0.141993963	0.052320342	0.385362263	1.27E-04
SASH3	4.78E-02	1.804884378	1.235429876	2.636821142	2.27E-03
SASH1	1.03E-02	0.394143039	0.199619964	0.778222439	7.31E-03
SAPCD2P3	3.62E-05	47755189857	40185.38129	5.67509E+16	5.70E-04
SAP30	2.73E-05	3.154503536	1.678162867	5.929634575	3.60E-04
SAP18	6.80E-03	33.12668752	4.681411367	234.4116635	4.55E-04
SAMSN1	8.36E-03	2.237896544	1.200210305	4.172752824	1.13E-02
SAMD4A	3.20E-02	2.285826847	1.222214553	4.275030404	9.65E-03
SALL4P5	7.23E-05	1.49E+75	7.56E+20	2.93E+129	6.65E-03
SAE1	9.37E-05	4.694428919	1.684303798	13.08413773	3.11E-03
SAAL1	1.92E-02	3.242383415	1.166868729	9.009625465	2.41E-02
S1PR5	3.32E-04	203.4655207	16.29776203	2540.116736	3.68E-05
S1PR4	1.84E-03	3.170772865	1.590092391	6.322777607	1.05E-03
S1PR2	4.21E-03	2.281316685	1.040366407	5.002473918	3.95E-02
S100A9	6.95E-03	1.932076698	1.205739844	3.095958375	6.19E-03
S100A6	8.02E-06	3.642884901	2.155306671	6.15717966	1.38E-06
S100A4	8.65E-04	1.733196369	1.392331131	2.157510946	8.55E-07
S100A3	3.19E-04	5.936124568	2.714134405	12.98298818	8.17E-06
S100A13	6.10E-06	7.058110872	3.092278993	16.11010171	3.47E-06
S100A11	2.28E-02	2.615656048	1.277674664	5.354772037	8.53E-03
S100A1	2.83E-03	1.660187837	1.108310487	2.486869597	1.39E-02

RYR1	2.63E-02	0.387004982	0.208252309	0.719189413	2.68E-03
RXRB	3.30E-04	0.147337118	0.063370462	0.342560648	8.64E-06
RUVBL1	7.28E-04	0.146275637	0.050359491	0.424876451	4.10E-04
RUNX2	1.97E-03	200.8339709	4.31188072	9354.220695	6.82E-03
RUNX1	3.93E-04	14.75821316	4.000897592	54.43899788	5.30E-05
RTN4RL1	1.49E-02	2.409416701	1.283800689	4.52195492	6.19E-03
RTN4R	1.97E-02	4.868015207	1.748800391	13.55075866	2.45E-03
RTN2	2.08E-02	0.447985477	0.274158613	0.732025106	1.35E-03
RTKN	8.77E-03	0.214386883	0.059892007	0.767410177	1.79E-02
RTF1	2.91E-02	2.001402477	1.076842417	3.719775347	2.82E-02
RSPH10B2	1.26E-02	9.20E-19	5.93E-32	1.43E-05	7.36E-03
RSAD2	4.03E-02	1.957603664	1.108451693	3.457265778	2.06E-02
RRP9	4.26E-03	0.237336808	0.102449537	0.549819571	7.92E-04
RRM2	3.79E-06	4.050201444	2.062755016	7.952535133	4.84E-05
RREB1	1.80E-04	0.115263263	0.037507582	0.354211579	1.62E-04
RPUSD4	7.41E-05	0.24340915	0.103691036	0.571389936	1.17E-03
RPUSD3	1.15E-03	0.141515273	0.04550164	0.440128588	7.31E-04
RPSAP64	1.58E-02	31491795.23	2.256438329	4.39513E+14	3.97E-02
RPSAP54	4.51E-02	0.150335984	0.024386537	0.926778073	4.12E-02
RPSA	1.20E-04	0.36502703	0.203707155	0.654099425	7.08E-04
RPS9	2.09E-02	0.385306645	0.192004358	0.773217922	7.28E-03
RPS6KA2	1.87E-02	1.8662627	1.299480136	2.680253718	7.29E-04
RPS4XP6	3.31E-02	0.171564176	0.03065596	0.960148251	4.48E-02
RPS4XP3	2.54E-02	0.062765304	0.006532292	0.603078244	1.65E-02
RPS4XP22	5.53E-03	0.654360031	0.475305772	0.900866505	9.32E-03
RPS4XP2	2.50E-02	0.0287391	0.001209763	0.682725161	2.81E-02
RPS4XP12	1.11E-02	1.33E+21	273944.8973	6.42E+36	8.30E-03
RPS4X	1.64E-02	0.36863453	0.192014218	0.707715389	2.71E-03
RPS3AP34	2.23E-02	0.001313123	2.66E-06	0.647564002	3.60E-02
RPS3	8.04E-03	0.387749724	0.193333798	0.777669759	7.63E-03
RPS2P5	6.33E-03	0.514130524	0.381026203	0.693732332	1.35E-05
RPS2P46	4.71E-02	0.657483182	0.440800896	0.980678894	3.98E-02
RPS29	7.65E-04	0.294782848	0.130180949	0.667508788	3.40E-03

RPS27L	3.99E-02	2.510895249	1.093678168	5.764579686	2.99E-02
RPS27AP3	1.02E-02	0.000205422	4.83E-08	0.874456866	4.64E-02
RPS27	1.64E-02	0.406991058	0.182346974	0.908387549	2.82E-02
RPS26P47	4.43E-02	1.528030433	1.044438617	2.235533009	2.90E-02
RPS25	2.35E-04	0.417814576	0.20024542	0.871775345	2.00E-02
RPS23P1	7.80E-04	0.005303883	0.000148848	0.188992579	4.06E-03
RPS20P22	5.48E-03	66893.1966	250.4595449	17865958.17	9.72E-05
RPS20P14	3.00E-03	4.005088714	2.090931739	7.671573062	2.86E-05
RPS20	1.57E-03	5.033577861	1.807062673	14.02104446	1.99E-03
RPS2	2.15E-02	0.448908166	0.233739072	0.862151712	1.62E-02
RPS19BP1	9.03E-04	13.5649793	3.326691723	55.31280888	2.77E-04
RPS19	2.61E-02	0.506621722	0.27350663	0.938425404	3.06E-02
RPS18	8.11E-05	0.479195959	0.276284724	0.831130883	8.84E-03
RPS17	1.62E-02	0.442504807	0.221473466	0.884126245	2.10E-02
RPS15A	3.98E-02	0.471499977	0.226245714	0.982614098	4.48E-02
RPS14P8	1.18E-02	1.55628355	1.06869923	2.266323787	2.11E-02
RPS14P7	5.28E-04	1.06E+20	33344.79934	3.35E+35	1.13E-02
RPS14	1.54E-02	0.450386965	0.206125763	0.984100265	4.55E-02
RPS13	2.57E-02	0.35978645	0.160881714	0.804605361	1.28E-02
RPS12P15	3.66E-05	4.58E+20	302557.7884	6.95E+35	7.64E-03
RPS12	7.39E-06	0.350460362	0.206751874	0.594057324	9.86E-05
RPS10P21	1.99E-03	5.84E+24	18817.15008	1.82E+45	1.78E-02
RPS10	2.64E-03	0.355544693	0.179962056	0.702437119	2.91E-03
RPP25	4.43E-04	1.912924238	1.253520403	2.919201899	2.63E-03
RPN2	1.94E-03	2.910699514	1.28806436	6.577444359	1.02E-02
RPLP1	4.47E-03	0.464253023	0.232209688	0.928173457	2.99E-02
RPLP0P2	2.77E-02	1009.700648	38.84520033	26245.08023	3.16E-05
RPL7P9	4.56E-03	1.987603877	1.206389422	3.274704751	7.01E-03
RPL7P8	2.20E-02	396.9362197	7.957168539	19800.80751	2.70E-03
RPL7P61	9.90E-03	144435250.5	5.120272097	4.0743E+15	3.18E-02
RPL7P5	2.25E-02	359173474.1	386.2180407	3.34023E+14	4.96E-03
RPL7P11	3.58E-02	641.3295363	3.153099836	130444.1963	1.72E-02
RPL7P1	3.37E-02	2.544033639	1.188720369	5.4446002	1.62E-02

RPL7A	1.73E-02	0.383142644	0.175588262	0.836037013	1.60E-02
RPL41	2.96E-04	0.2875318	0.133521314	0.619186057	1.45E-03
RPL3L	1.99E-03	0.00014418	7.58E-07	0.027410245	9.55E-04
RPL39P40	1.89E-02	0.32292729	0.143033713	0.729073119	6.52E-03
RPL39P28	1.99E-03	218533943.5	27.50038963	1.7366E+15	1.78E-02
RPL38	1.26E-02	0.443339595	0.2021778	0.972164088	4.23E-02
RPL37A	1.24E-02	0.441830594	0.218844173	0.892024089	2.27E-02
RPL36P16	4.30E-02	9.29E-07	1.05E-12	0.826207374	4.69E-02
RPL35P2	1.81E-03	9.602705306	2.356627885	39.12876946	1.60E-03
RPL35A	1.30E-05	0.340702883	0.186277524	0.623147935	4.73E-04
RPL34-AS1	9.26E-04	1.16E-11	3.25E-19	0.000415648	4.55E-03
RPL34	4.89E-02	0.510184588	0.264389215	0.984489151	4.48E-02
RPL32P3	1.12E-03	0.145623499	0.043665364	0.485652736	1.72E-03
RPL32P1	2.89E-03	191.8513438	12.55492811	2931.672551	1.58E-04
RPL32	4.31E-05	0.405819249	0.250198491	0.658234437	2.58E-04
RPL30P6	1.06E-02	20087686091	4.468753624	9.0297E+19	3.64E-02
RPL30P4	3.27E-03	13.55869336	2.339494337	78.5802995	3.64E-03
RPL30P3	5.96E-03	4162.575421	2.450795281	7069963.891	2.81E-02
RPL30P11	1.53E-02	1.57E-08	5.15E-15	0.047696921	1.83E-02
RPL30P1	2.76E-06	1.69E+20	3664251.549	7.84E+33	3.71E-03
RPL29	5.38E-04	0.439779508	0.264289502	0.731796059	1.57E-03
RPL26P34	3.56E-03	9037174906	4.294503958	1.90175E+19	3.63E-02
RPL26L1	1.43E-03	4.634864893	1.839084922	11.68079425	1.15E-03
RPL24P2	2.25E-03	0.233334037	0.091094578	0.597673033	2.43E-03
RPL24	1.92E-04	0.387823794	0.210608812	0.714154807	2.36E-03
RPL23	5.74E-03	0.376723176	0.17467579	0.812478657	1.28E-02
RPL22	1.15E-02	0.316915872	0.154674513	0.649335618	1.69E-03
RPL21P126	1.99E-03	8.33E+24	20004.58907	3.47E+45	1.78E-02
RPL21P121	3.53E-03	93031876719	1239.668601	6.98165E+18	6.34E-03
RPL17P51	3.27E-02	12438.20504	1.665577068	92886091.92	3.83E-02
RPL17P50	2.28E-04	7.831065527	2.918287177	21.01424005	4.38E-05
RPL15P3	3.07E-04	0.472792399	0.259312502	0.862020347	1.45E-02
RPL15P21	2.58E-02	1.90E-08	9.58E-15	0.037551723	1.62E-02

RPL15P20	9.56E-04	0.000521967	2.10E-06	0.129548302	7.22E-03
RPL15	2.11E-04	0.300400368	0.155721976	0.579496763	3.34E-04
RPL14P1	3.25E-02	0.491077625	0.281995415	0.855181402	1.20E-02
RPL14	4.31E-05	0.383648751	0.216468448	0.679943732	1.03E-03
RPL13A	1.26E-02	0.392136508	0.17751955	0.866220316	2.06E-02
RPL12P44	1.70E-02	1.09E-08	2.13E-15	0.055356713	2.00E-02
RPL12P4	4.40E-02	0.753136692	0.578965491	0.979704121	3.46E-02
RPL12	9.01E-03	0.450501718	0.23650736	0.858120429	1.53E-02
RPL10A	5.02E-04	0.294911627	0.147178518	0.590934523	5.74E-04
RPL10	1.53E-03	0.341320956	0.170361596	0.683839539	2.43E-03
RPEP3	2.12E-05	4.38756E+15	46561.89621	4.13E+26	5.21E-03
RPA3	1.23E-02	4.180006279	1.047489697	16.68030964	4.28E-02
RPA1	1.37E-02	0.415074382	0.192052007	0.897083792	2.53E-02
RP6-65G23.3	4.38E-02	0.12760318	0.035113851	0.463707946	1.76E-03
RP6-29D12.3	7.46E-05	129813745.5	1125.343549	1.49746E+13	1.68E-03
RP6-24A23.6	1.75E-03	2.09E-06	1.12E-10	0.038865432	9.12E-03
RP6-24A23.3	3.02E-02	0.255923902	0.08243343	0.79454468	1.84E-02
RP6-109B7.5	2.17E-04	735.4198376	20.63970375	26203.97773	2.94E-04
RP6-109B7.4	6.65E-05	4.363460184	1.538142745	12.37842511	5.62E-03
RP5-998N21.7	1.53E-02	117.2930562	2.127332106	6467.09604	1.99E-02
RP5-998N21.10	2.23E-03	4.485816727	1.917568166	10.4937869	5.37E-04
RP5-991G20.1	8.02E-04	0.107416764	0.025647889	0.449875667	2.27E-03
RP5-981L23.2	4.76E-04	2.11E-05	1.63E-08	0.027174417	3.21E-03
RP5-968P14.2	1.91E-03	0.023805177	0.002189588	0.258809638	2.14E-03
RP5-968J1.1	2.33E-03	0.043748671	0.004171861	0.458775172	9.06E-03
RP5-945F2.3	2.76E-06	6.03E+37	1.81544E+12	2.00E+63	3.71E-03
RP5-943J3.2	3.44E-02	0.169760949	0.038664358	0.74535778	1.88E-02
RP5-943J3.1	3.21E-02	35.27127714	1.134252558	1096.813035	4.22E-02
RP5-934G17.6	3.13E-02	4.19E-10	2.76E-18	0.063629146	2.47E-02
RP5-912I13.1	2.92E-02	98.70523644	3.589614733	2714.141886	6.61E-03
RP5-907C10.4	2.15E-02	1.29E-14	1.44E-27	0.116313757	3.56E-02
RP5-906C1.1	3.92E-02	3.82597737	1.33375949	10.9750693	1.26E-02
RP5-887A10.1	1.29E-02	17.38401648	1.412220882	213.992041	2.58E-02

RP5-884M6.1	2.10E-02	1.09E+35	3656.036699	3.25E+66	2.91E-02
RP5-884G6.2	1.01E-04	0.039429367	0.006733992	0.230869729	3.36E-04
RP5-882C2.2	1.22E-02	3.715448153	1.04041058	13.26837236	4.33E-02
RP5-877J2.1	2.83E-03	2.52954E+14	401409.6851	1.59E+23	1.34E-03
RP5-875H18.9	3.79E-05	7.523102945	2.171177551	26.06745721	1.46E-03
RP5-875H18.4	1.24E-02	0.000178624	1.25E-07	0.256226816	2.00E-02
RP5-875H18.10	8.68E-03	0.023478874	0.001131996	0.486978162	1.53E-02
RP5-858L17.1	3.69E-02	0.332122249	0.113329951	0.973310125	4.45E-02
RP5-858B6.3	1.73E-03	0.014369939	0.000961365	0.214793633	2.11E-03
RP5-849L7.1	4.96E-02	27482.23727	4.704167785	160554087.4	2.09E-02
RP5-837G17.1	1.88E-02	1.1998E+12	1.170901497	1.23E+24	4.87E-02
RP5-827C21.6	3.83E-02	8.123117382	2.414289137	27.33104126	7.15E-04
RP5-827C21.4	5.04E-03	0.453115507	0.208206604	0.986105431	4.60E-02
RP5-1193P9.4	1.88E-02	73158530.72	1.108182012	4.82969E+15	4.87E-02
RP5-1185I7.1	3.31E-03	4.94E+36	5.61462E+15	4.34E+57	5.95E-04
RP5-1180D12.1	9.97E-03	2.364676814	1.162032227	4.811997728	1.76E-02
RP5-1173A5.1	2.76E-06	1.91E+23	35816792.41	1.02E+39	3.71E-03
RP5-1171I10.5	7.09E-03	2.336934145	1.340209597	4.074930676	2.77E-03
RP5-1166F10.1	1.11E-02	0.014438793	0.000228359	0.912942936	4.52E-02
RP5-1157M23.2	2.92E-02	0.012503363	0.000335915	0.465397136	1.76E-02
RP5-1142A6.8	8.21E-03	1.36E-19	3.32E-36	0.005605165	2.60E-02
RP5-1139B12.2	1.57E-02	41.65447663	2.892434847	599.873641	6.14E-03
RP5-1136G13.2	2.64E-02	0.182199407	0.03381501	0.981712663	4.75E-02
RP5-1111A8.3	2.61E-02	39340578.61	3.188640068	4.85373E+14	3.58E-02
RP5-1103G7.10	1.48E-03	1.90E+58	350707767.7	1.03E+108	2.16E-02
RP5-1098D14.1	1.40E-03	97.10550071	1.148528681	8210.050321	4.33E-02
RP5-1091N2.9	9.68E-04	10.270334	2.943923111	35.82965873	2.59E-04
RP5-1074L1.1	2.68E-03	8264.696591	13.02035707	5246031.992	6.15E-03
RP5-1071N3.1	2.23E-04	7841.695665	47.20603693	1302634.047	5.87E-04
RP5-1063M23.2	5.74E-03	1.08908E+11	4842.19676	2.44949E+18	3.26E-03
RP5-1054A22.4	1.12E-02	4.40590202	1.772659983	10.9507592	1.41E-03
RP5-1050D4.2	1.28E-03	39.37610153	5.223490713	296.827822	3.65E-04
RP5-1039K5.12	5.36E-03	0.052343993	0.010006402	0.27381407	4.75E-04

RP5-1028K7.2	1.41E-02	2.695328483	1.271333794	5.714310169	9.71E-03
RP5-1024G6.5	4.07E-03	0.08754039	0.019292602	0.397215479	1.60E-03
RP5-1024C24.1	3.39E-02	0.072147746	0.006566708	0.792679877	3.16E-02
RP5-1009E24.8	8.33E-03	7.580666503	1.502041157	38.25894143	1.42E-02
RP4-816N1.6	1.02E-02	0.096812915	0.022541443	0.415800371	1.69E-03
RP4-797C5.2	4.82E-06	5.09151E+13	228218.6672	1.14E+22	1.29E-03
RP4-794I6.4	1.94E-02	2.845785424	1.409313167	5.746412413	3.54E-03
RP4-791M13.4	4.44E-02	30540.99337	3.984785317	234078426.3	2.36E-02
RP4-785G19.5	6.39E-03	3.380823815	1.247153348	9.164847038	1.67E-02
RP4-781L3.1	3.54E-02	32.94864222	1.822067605	595.8138002	1.80E-02
RP4-781K5.9	3.18E-02	2157967986	18.63927694	2.49839E+17	2.33E-02
RP4-781B1.5	2.05E-02	0.020204763	0.001204151	0.339021024	6.69E-03
RP4-775C13.1	2.92E-02	132403261.5	310.74541	5.64147E+13	4.69E-03
RP4-765C7.2	1.59E-02	1.311322115	1.072291105	1.603636998	8.30E-03
RP4-758J18.7	1.85E-02	0.233378963	0.073370436	0.742339053	1.37E-02
RP4-756H11.5	3.35E-03	3.503387314	1.445240156	8.492514284	5.52E-03
RP4-753F5.1	1.76E-02	0.078890489	0.01008708	0.616998126	1.55E-02
RP4-742C19.12	2.79E-03	16.58208435	2.586617727	106.3031149	3.05E-03
RP4-740C4.5	1.40E-03	0.057601431	0.0125689	0.26397893	2.38E-04
RP4-739H11.3	1.53E-02	9.892199108	1.82665052	53.57105922	7.84E-03
RP4-736H5.1	7.93E-03	1634496.254	1.724306861	1.54936E+12	4.16E-02
RP4-735N21.1	2.76E-06	5.20E+23	49605009.36	5.46E+39	3.71E-03
RP4-725G10.3	3.15E-03	6.717150353	1.54126633	29.27469964	1.12E-02
RP4-724E13.2	3.64E-03	38.10162685	1.217111234	1192.770166	3.83E-02
RP4-714D9.2	1.75E-14	4.64E+22	2.02268E+12	1.07E+33	1.80E-05
RP4-713A8.1	3.07E-03	0.001730698	1.57E-05	0.191059501	8.06E-03
RP4-710M3.2	1.19E-03	0.089398769	0.014283723	0.559527774	9.87E-03
RP4-673M15.1	2.66E-03	6.881862545	1.576297199	30.04511594	1.03E-02
RP4-671O14.7	1.29E-02	6.59E+44	6.61002E+16	6.56E+72	1.70E-03
RP4-655J12.4	3.30E-05	0.031914974	0.003495336	0.291407025	2.27E-03
RP4-647J21.1	4.16E-05	11.4013463	3.333283698	38.99778994	1.05E-04
RP4-639F20.1	3.00E-02	0.391184847	0.203359028	0.752489751	4.92E-03
RP4-635G19.1	2.32E-03	1.23E+23	41325179.73	3.66E+38	3.45E-03

RP4-633O19__A.1	7.58E-03	0.001008028	5.52E-06	0.184221588	9.42E-03
RP4-631H13.5	2.10E-04	118618.9366	73.17874627	192275118.5	1.95E-03
RP4-631H13.2	5.03E-03	3.89738E+11	436.9138547	3.48E+20	1.11E-02
RP4-616B8.5	8.12E-03	339.1704394	27.32076823	4210.591224	5.80E-06
RP4-604G5.1	1.52E-05	0.000159085	1.06E-06	0.023894438	6.26E-04
RP4-591L5.1	2.76E-06	9.84E+29	5397170882	1.79E+50	3.71E-03
RP4-583P15.16	1.24E-02	77.99710033	7.645284542	795.7254732	2.36E-04
RP4-576H24.5	1.04E-09	2.16901E+16	30165476.46	1.56E+25	3.00E-04
RP4-568C11.4	7.66E-03	7723.567639	1.949446471	30600223.16	3.42E-02
RP4-562J12.1	3.91E-02	1.44E-05	2.19E-10	0.942466898	4.88E-02
RP4-545C24.1	3.15E-02	0.007178584	0.000233254	0.220926828	4.75E-03
RP4-539M6.19	2.62E-02	2.58E+30	15.72949178	4.24E+59	4.13E-02
RP3-527G5.1	1.82E-03	0.133523279	0.035154102	0.507151795	3.11E-03
RP3-509I19.1	5.69E-03	8.58E+20	61095.63977	1.20E+37	1.11E-02
RP3-508I15.9	1.84E-05	0.019971884	0.002270479	0.175679316	4.19E-04
RP3-492J12.2	2.88E-02	11339608.45	309.7446168	4.15138E+11	2.45E-03
RP3-472M2.2	1.27E-02	3101203.793	132.9561137	72335635409	3.58E-03
RP3-467K16.4	4.16E-03	2077.032466	8.757181333	492631.5558	6.19E-03
RP3-462D8.2	4.98E-02	0.561518298	0.359788686	0.876355515	1.11E-02
RP3-461P17.9	7.23E-05	3.42499E+15	20637.41268	5.68E+26	6.65E-03
RP3-455J7.4	2.78E-02	145.4572415	1.506507871	14044.27386	3.27E-02
RP3-453C12.8	3.70E-04	22111.68971	4.728091311	103408921.2	2.03E-02
RP3-452M16.1	7.74E-03	41051986070	2544.039319	6.62437E+17	3.90E-03
RP3-450M14.1	2.76E-06	6.23E+31	20736599671	1.87E+53	3.71E-03
RP3-441A12.1	1.44E-04	0.062612624	0.009582166	0.409128884	3.81E-03
RP3-439F8.1	4.66E-05	6.099735298	2.742674725	13.56587071	9.25E-06
RP3-430A16.1	1.27E-02	4.93E-07	5.78E-13	0.421225038	3.72E-02
RP3-419C19.3	3.57E-06	5.61244E+17	39311734.33	8.01E+27	6.13E-04
RP3-419C19.2	1.99E-03	4.94E+29	133342.2361	1.83E+54	1.78E-02
RP3-388M5.9	5.45E-03	0.001917457	2.00E-05	0.183539616	7.18E-03
RP3-370M22.8	4.27E-03	31408.00728	53.30029753	18507643.8	1.46E-03
RP3-337O18.9	1.52E-02	23.76567626	2.359367538	239.3893104	7.18E-03
RP3-333H23.9	1.28E-02	0.199765893	0.054778263	0.728508176	1.47E-02

RP3-333H23.8	7.92E-06	9.45E+28	1.50698E+16	5.93E+41	9.09E-06
RP3-326L13.2	5.28E-04	1.79E+70	7.39368E+15	4.32E+124	1.13E-02
RP3-326I13.1	3.19E-06	2.06E+32	2338958167	1.82E+55	5.78E-03
RP3-323P24.3	9.17E-04	0.029312494	0.003546346	0.242283798	1.05E-03
RP13-942N8.1	1.35E-03	0.109404792	0.026308628	0.454961334	2.34E-03
RP13-93L13.2	1.05E-02	22783.62821	8.965688154	57897810.56	1.21E-02
RP13-884E18.4	3.41E-02	49523.64017	4.847921877	505905622.6	2.17E-02
RP13-766D20.4	3.20E-07	43.06262787	10.20332569	181.7436761	3.03E-07
RP13-735L24.1	2.81E-08	0.216464402	0.10051665	0.466159956	9.23E-05
RP13-650G11.1	1.14E-02	0.008793376	0.00011047	0.699947373	3.40E-02
RP13-582O9.7	1.69E-02	2.851815173	1.445346836	5.626919143	2.51E-03
RP13-57D9.3	5.89E-04	4157669972	4838.713069	3.57248E+15	1.49E-03
RP13-516M14.2	3.54E-02	0.00156352	5.16E-06	0.473307107	2.67E-02
RP13-476E20.1	3.34E-03	0.014121325	0.000567362	0.351472125	9.39E-03
RP13-46H24.1	1.60E-04	0.086125656	0.019295213	0.384428441	1.32E-03
RP13-465B17.4	1.34E-03	0.084746575	0.017050046	0.421229473	2.56E-03
RP13-463N16.6	2.26E-02	1.29646E+12	1300.248399	1.29E+21	8.33E-03
RP13-452N2.1	1.70E-02	2.34233E+18	908041.4768	6.04E+30	3.72E-03
RP13-314C10.5	2.61E-02	3.547591085	1.381662368	9.108884196	8.49E-03
RP13-270P17.1	2.44E-02	7.388879692	1.779860227	30.67406208	5.89E-03
RP13-20L14.6	2.04E-02	3.518580087	1.302614122	9.5042773	1.31E-02
RP13-140E4.1	7.49E-03	1.0589E+17	50.78261707	2.21E+32	2.94E-02
RP13-1039J1.2	5.16E-03	9.24E+148	4.26988E+17	2.00E+280	2.62E-02
RP13-1032I1.7	1.90E-02	0.283128776	0.10064619	0.796472314	1.68E-02
RP11-99A1.2	5.28E-04	3.56E+48	92672809499	1.37E+86	1.13E-02
RP11-999E24.3	1.31E-03	0.380434856	0.202781327	0.713727847	2.61E-03
RP11-98L5.5	2.76E-06	1.8415E+12	9546.013981	3.55E+20	3.71E-03
RP11-98D18.9	3.73E-02	0.00446659	6.06E-05	0.32901878	1.36E-02
RP11-98D18.17	1.55E-02	0.000369497	1.85E-07	0.736789089	4.15E-02
RP11-983P16.4	2.40E-04	3.609385847	1.539025847	8.464878099	3.16E-03
RP11-982M15.8	1.73E-02	6968959.078	4.976113889	9.7599E+12	2.91E-02
RP11-982M15.6	2.06E-05	7.41507326	2.641613486	20.81429087	1.42E-04
RP11-97O12.7	8.10E-03	0.058131568	0.008112745	0.416539562	4.63E-03

RP11-977P2.1	2.65E-02	1268223.64	16.56787703	97078895487	1.43E-02
RP11-973N13.5	2.35E-03	571.6437003	3.042057192	107419.5847	1.75E-02
RP11-96H17.3	4.99E-03	0.201158717	0.04526958	0.893863596	3.51E-02
RP11-96D1.9	1.48E-03	3.11E+41	1221232.792	7.93E+76	2.16E-02
RP11-96A1.5	4.76E-02	4073641657	31.64566713	5.24386E+17	2.02E-02
RP11-968O1.5	1.19E-02	5.277224935	1.889513433	14.73876953	1.50E-03
RP11-964E11.2	3.78E-06	7.13E+41	1.99302E+19	2.55E+64	2.76E-04
RP11-95I16.6	1.32E-05	35757.32306	1.948234247	656279476.9	3.63E-02
RP11-959I15.1	2.76E-06	1.38E+85	4.23E+27	4.51E+142	3.71E-03
RP11-959F10.5	5.16E-03	2.69E+60	14188258.81	5.09E+113	2.62E-02
RP11-94H6.1	1.88E-02	2417014417	1.130388962	5.1681E+18	4.87E-02
RP11-94C24.13	9.64E-03	5.02E-09	5.81E-15	0.00433473	6.14E-03
RP11-94A24.1	5.28E-04	4.15E+24	363780.5378	4.74E+43	1.13E-02
RP11-945A11.1	5.28E-04	2.02E+30	7009632.046	5.85E+53	1.13E-02
RP11-944L7.4	3.53E-03	0.006723655	0.000370298	0.122084353	7.20E-04
RP11-93O14.1	2.46E-03	11178.48355	4.204514501	29720076.93	2.05E-02
RP11-93H12.4	1.14E-02	3.36E+20	694175.9275	1.63E+35	6.15E-03
RP11-93H12.3	2.76E-06	1.74E+24	73324817.56	4.11E+40	3.71E-03
RP11-92K15.1	5.28E-04	2.22805E+16	4928.643989	1.01E+29	1.13E-02
RP11-92F20.1	8.53E-03	22236140503	1876.807963	2.6345E+17	4.14E-03
RP11-92C4.4	1.00E-02	2428284792	614.0136805	9.60332E+15	5.30E-03
RP11-927P21.5	2.45E-03	1.71597E+15	7130.976494	4.13E+26	8.70E-03
RP11-925D8.5	2.18E-02	1.25756E+17	393.6381994	4.02E+31	2.09E-02
RP11-91J19.3	4.97E-02	8.502161886	2.235425519	32.33691131	1.69E-03
RP11-91I8.3	9.98E-03	0.10314675	0.013407077	0.793554909	2.91E-02
RP11-91I20.2	3.16E-02	162.0272213	1.325536426	19805.43116	3.80E-02
RP11-90M2.5	4.71E-02	0.215233767	0.057786597	0.801666425	2.21E-02
RP11-90L1.8	6.27E-03	1.83E-06	2.52E-11	0.132601524	2.07E-02
RP11-90J7.4	5.16E-03	2.58628E+17	115.0350354	5.81E+32	2.62E-02
RP11-903H12.5	6.72E-04	6.98E-15	1.38E-24	3.54E-05	4.25E-03
RP11-902B17.1	3.00E-02	0.001601566	1.36E-05	0.188015489	8.11E-03
RP11-89N17.2	4.85E-03	2.55674E+12	6759.508718	9.67E+20	4.58E-03
RP11-89H19.1	6.11E-03	1.2965E+18	242955617.6	6.92E+27	2.63E-04

RP11-895K13.2	1.31E-03	15885.25907	46.60398473	5414589.699	1.15E-03
RP11-893F2.18	2.03E-02	0.001591884	7.41E-06	0.34186108	1.87E-02
RP11-893F2.14	4.12E-02	0.288194382	0.12403905	0.669595598	3.82E-03
RP11-893F2.13	1.47E-02	0.191071072	0.051556669	0.708117015	1.33E-02
RP11-88I21.1	1.99E-03	1.71E+31	245877.2047	1.19E+57	1.78E-02
RP11-88E10.5	8.38E-03	0.083078633	0.014931931	0.462234876	4.49E-03
RP11-88E10.4	1.00E-03	48.59477785	4.603886984	512.9258043	1.24E-03
RP11-889L3.4	1.08E-02	9.72305393	2.716722303	34.79846933	4.72E-04
RP11-887P2.3	1.14E-02	39.22063379	6.402023359	240.2768669	7.26E-05
RP11-882I15.1	4.79E-02	4.05361E+13	118.7141819	1.38E+25	2.07E-02
RP11-881M11.8	2.33E-02	8.32819E+11	4.402247986	1.58E+23	3.83E-02
RP11-881M11.2	7.86E-03	0.011693864	0.000765192	0.178708603	1.39E-03
RP11-881M11.1	1.34E-02	36801.26273	14.19181975	95430534.07	8.76E-03
RP11-87C7.3	1.47E-02	17025652056	4.519229798	6.41421E+19	3.63E-02
RP11-876N24.5	2.10E-02	6.392713158	2.206637048	18.5199381	6.30E-04
RP11-876N24.4	4.88E-06	10.86214466	3.501001599	33.70069485	3.64E-05
RP11-875O11.2	1.14E-04	7.80E-13	3.44E-21	0.000176626	4.51E-03
RP11-874J12.4	1.32E-04	124293109.5	3143.227621	4.91494E+12	5.58E-04
RP11-86K22.2	4.89E-03	67.12034941	2.643824218	1704.024524	1.08E-02
RP11-86H7.7	1.04E-05	0.022538202	0.003459586	0.146829868	7.30E-05
RP11-867G23.10	2.47E-04	5.195224839	1.841820464	14.65417594	1.84E-03
RP11-865I6.2	1.14E-03	3006266.094	458.8938051	19694394931	8.78E-04
RP11-864I4.1	2.96E-03	22.49157511	3.717717021	136.0703216	7.00E-04
RP11-864G5.8	1.88E-02	76202.54453	1.065843953	5448103145	4.87E-02
RP11-85L21.6	9.37E-03	99798406687	6.083273238	1.64E+21	3.48E-02
RP11-857B24.1	2.48E-03	0.001225169	2.48E-06	0.604363741	3.41E-02
RP11-856M7.6	1.99E-03	3.36E+44	48416042.41	2.34E+81	1.78E-02
RP11-84A14.4	4.82E-03	0.231090244	0.061251264	0.871862833	3.06E-02
RP11-848P1.9	3.74E-03	0.042296821	0.007064308	0.25324788	5.32E-04
RP11-848P1.5	4.31E-03	0.130366212	0.044271738	0.383887103	2.18E-04
RP11-834C11.10	9.98E-04	433.3526575	2.89502974	64867.90901	1.75E-02
RP11-831H9.11	4.98E-02	1036.796104	9.998313185	107512.7516	3.37E-03
RP11-831F12.4	7.23E-05	63837532.41	147.1673659	2.76911E+13	6.65E-03

RP11-831A10.1	3.42E-02	1.79502E+13	1.329443656	2.42E+26	4.79E-02
RP11-82L18.2	2.13E-02	54.55477579	1.517951705	1960.683961	2.86E-02
RP11-81M19.3	1.15E-02	59099.52396	20.71503975	168609559.8	6.80E-03
RP11-817O13.8	2.65E-03	3.068901297	1.002573614	9.393978697	4.95E-02
RP11-817I4.1	5.86E-03	4.066462155	1.399425411	11.81636	9.95E-03
RP11-816B4.1	5.16E-03	3.18437E+11	22.98313821	4.41E+21	2.62E-02
RP11-815N9.2	2.75E-02	5.98429235	1.737679776	20.60894961	4.57E-03
RP11-815J21.4	7.61E-03	19.407887	2.63505461	142.9443156	3.60E-03
RP11-815I9.4	1.27E-02	0.061393722	0.011255499	0.33487535	1.26E-03
RP11-815D16.1	5.28E-04	1.35E+23	167799.9409	1.09E+41	1.13E-02
RP11-814E24.1	1.88E-02	1.65232E+13	1.188451369	2.30E+26	4.87E-02
RP11-812E19.12	2.76E-06	1.61075E+12	9140.180973	2.84E+20	3.71E-03
RP11-810K23.9	2.44E-05	2635.014801	6.551707815	1059769.941	1.00E-02
RP11-810D13.1	5.92E-04	1.07909E+11	8235.92534	1.41386E+18	2.38E-03
RP11-80I3.1	2.76E-06	2.22E+135	8.27E+43	5.97E+226	3.71E-03
RP11-809O17.1	3.03E-05	3.042042716	1.762668307	5.250008667	6.45E-05
RP11-809H16.2	4.47E-03	1.62994E+19	456128.9764	5.82E+32	5.46E-03
RP11-809C18.3	5.64E-05	23.7631347	3.418244633	165.1978227	1.36E-03
RP11-807H7.2	8.12E-04	4.026968794	1.046964204	15.48904691	4.27E-02
RP11-807H22.7	2.91E-02	53.30773366	5.783678129	491.3334395	4.50E-04
RP11-806O11.1	2.17E-05	0.010960067	0.000276091	0.435085708	1.63E-02
RP11-806L2.5	3.61E-02	55.99805646	2.119975314	1479.159831	1.60E-02
RP11-805F19.3	1.49E-02	166.8524413	2.206099994	12619.43576	2.04E-02
RP11-805F19.2	8.15E-03	2.283537021	1.028621608	5.069445639	4.24E-02
RP11-805F19.1	5.54E-04	6.96386E+13	16595.67718	2.92E+23	4.81E-03
RP11-804H8.5	1.99E-03	5.69321E+12	159.0301998	2.04E+23	1.78E-02
RP11-803B1.8	3.43E-03	0.012764948	0.000487051	0.334552044	8.87E-03
RP11-79N23.1	5.07E-03	0.012196726	0.000339816	0.437766095	1.59E-02
RP11-799B12.1	4.92E-03	2.462241134	1.122556215	5.400737463	2.45E-02
RP11-794G24.1	3.72E-02	19027645.99	321.8963027	1.12475E+12	2.79E-03
RP11-793H13.8	1.08E-03	7007.227376	19.95046173	2461157.851	3.07E-03
RP11-791G16.5	2.14E-02	222134228.6	3.036449809	1.62504E+16	3.75E-02
RP11-78J21.7	1.11E-02	0.016245624	0.001434274	0.184009688	8.78E-04

RP11-78F17.1	3.55E-03	48533.91114	46.83589885	50293484.03	2.32E-03
RP11-788M5.3	2.70E-02	707092784.4	96.03120192	5.20643E+15	1.15E-02
RP11-787B4.2	5.16E-03	1.39E+23	548.2544833	3.52E+43	2.62E-02
RP11-783K16.5	1.79E-05	7.2739268	3.02352662	17.49943617	9.41E-06
RP11-780O17.1	8.79E-03	1.23384E+16	1529.606316	9.95E+28	1.45E-02
RP11-77P16.4	1.81E-05	0.059074385	0.00967791	0.360592621	2.18E-03
RP11-778D9.4	5.44E-03	0.374334279	0.148946439	0.940782156	3.66E-02
RP11-778D9.12	8.32E-03	0.050905007	0.005625679	0.460623425	8.06E-03
RP11-775C24.3	4.48E-02	2888.081326	2.69974751	3089553.269	2.52E-02
RP11-775B15.2	1.89E-02	16742.54309	2.913362601	96216224.22	2.77E-02
RP11-774O3.3	7.23E-04	0.136848139	0.048342209	0.38739258	1.80E-04
RP11-774O3.1	5.28E-04	2.48E+30	7336044.014	8.36E+53	1.13E-02
RP11-773H22.4	1.92E-02	155.4839936	11.84163684	2041.548191	1.22E-04
RP11-767C1.2	7.13E-06	0.14667228	0.03841658	0.559986278	4.98E-03
RP11-764E7.1	4.45E-03	1.75E+26	8062.69313	3.82E+48	2.13E-02
RP11-762I7.5	2.34E-02	87922660.94	16218.807	4.76631E+11	3.05E-05
RP11-762H8.5	1.50E-03	143425.3477	26.61128524	773011532.1	6.76E-03
RP11-760H22.2	2.64E-02	2.855432627	1.206088912	6.760277297	1.70E-02
RP11-759A24.2	2.60E-02	4.01E+64	2307.607914	6.98E+125	3.87E-02
RP11-758N13.3	1.17E-03	4.0256692	1.688767169	9.596356919	1.68E-03
RP11-757G1.5	2.68E-04	0.004378825	0.000193258	0.099214844	6.47E-04
RP11-752L20.3	3.99E-02	420872.2362	1080.704213	163905569.2	2.09E-05
RP11-751H17.1	1.81E-02	16239.87379	52.53015077	5020611.911	9.20E-04
RP11-74K19.1	1.99E-03	4.60E+22	8153.582262	2.59E+41	1.78E-02
RP11-74E22.8	2.94E-03	0.029001227	0.002500194	0.336402382	4.64E-03
RP11-74E22.4	1.10E-02	0.000151518	1.97E-07	0.116437783	9.48E-03
RP11-74C3.1	1.43E-02	2.09202E+16	25.55121929	1.71E+31	3.20E-02
RP11-744K17.1	2.05E-02	5.28186E+19	2.210650841	1.26E+39	4.61E-02
RP11-744I24.2	2.39E-02	1.36E-09	4.64E-18	0.401214361	4.02E-02
RP11-73M18.8	4.74E-02	4.562522274	1.779582679	11.69746691	1.58E-03
RP11-73M14.1	9.43E-03	237.9620851	1.799578735	31466.22755	2.81E-02
RP11-73K9.3	2.77E-02	208.7674368	10.77710568	4044.113878	4.12E-04
RP11-73E17.2	3.81E-03	13.17207785	1.453036135	119.4076532	2.19E-02

RP11-739L10.1	2.47E-02	4.980944137	1.32909189	18.66673379	1.72E-02
RP11-738O11.12	5.15E-03	28.80481643	2.470572871	335.8401038	7.32E-03
RP11-737G21.1	7.23E-05	1.59771E+11	1293.308917	1.97376E+19	6.65E-03
RP11-736N17.10	1.94E-02	32.99299848	2.82759806	384.9691241	5.28E-03
RP11-736K20.5	1.20E-03	22.94888203	2.78230713	189.2857841	3.61E-03
RP11-736I10.2	1.83E-02	1.0711E+15	2.408549409	4.76E+29	4.43E-02
RP11-734K21.3	6.35E-05	135.2328362	13.98117549	1308.038798	2.25E-05
RP11-734K2.4	1.03E-02	0.001242389	2.75E-06	0.562024793	3.20E-02
RP11-730G20.1	1.43E-04	5.20E+23	71976948.27	3.76E+39	3.38E-03
RP11-728F11.3	1.92E-03	4322.386368	17.54140606	1065081.32	2.89E-03
RP11-726G1.1	1.12E-08	0.131888577	0.04901245	0.354901594	6.04E-05
RP11-723O4.6	4.02E-03	1.16E-07	2.65E-13	0.050736174	1.60E-02
RP11-720L3.1	3.82E-09	1.10103E+14	83834.9645	1.45E+23	2.54E-03
RP11-71E19.1	2.50E-05	0.055614178	0.01098447	0.28157359	4.80E-04
RP11-717A5.1	3.60E-03	6028.094288	6.849509895	5305185.525	1.19E-02
RP11-716D16.1	2.76E-06	5.91E+100	5.00E+32	7.00E+168	3.71E-03
RP11-715J22.6	1.37E-02	0.186564376	0.042675108	0.815610516	2.57E-02
RP11-714G18.1	3.03E-02	2.591600745	1.267341667	5.299592522	9.08E-03
RP11-713M15.2	1.47E-02	2.816344436	1.459234027	5.435588696	2.03E-03
RP11-711M9.2	3.57E-04	28031743.45	1074.79934	7.31093E+11	9.49E-04
RP11-711K1.8	1.67E-02	2.29493E+11	11505.08502	4.57772E+18	2.29E-03
RP11-710E1.1	3.43E-06	7.46E+24	6.14572E+11	9.06E+37	1.95E-04
RP11-70F11.11	5.16E-03	2.67E+110	1.17291E+13	6.09E+207	2.62E-02
RP11-707O23.1	1.99E-02	7.750122084	1.52067874	39.49840999	1.37E-02
RP11-707M3.3	9.99E-03	5.656662285	1.947640701	16.42902009	1.45E-03
RP11-707M1.7	5.28E-04	1.61E+35	89664569.14	2.89E+62	1.13E-02
RP11-705O24.1	3.34E-02	8.53E+66	435.8785081	1.67E+131	4.13E-02
RP11-704J17.5	1.61E-02	2221954.83	151.3912318	32611421462	2.83E-03
RP11-703I16.1	4.07E-03	0.107963765	0.023435004	0.497383087	4.29E-03
RP11-702F3.4	2.05E-02	2.23002E+12	14.40800696	3.45E+23	3.05E-02
RP11-702F3.3	3.12E-03	2.01273E+18	1399063.619	2.90E+30	3.17E-03
RP11-6O2.2	4.56E-02	101823.9219	3.681663347	2816148600	2.71E-02
RP11-6I2.3	3.79E-03	4.460128822	1.296996178	15.33755414	1.77E-02

RP11-699F16.2	3.28E-03	2.95965E+13	3098.297057	2.83E+23	8.16E-03
RP11-694I15.7	2.73E-05	0.019083605	0.001876723	0.194053173	8.21E-04
RP11-693N9.2	6.05E-03	23.71300141	3.536022204	159.0223148	1.11E-03
RP11-693J15.5	2.87E-02	2.29E+36	294509156.5	1.79E+64	1.06E-02
RP11-68I3.10	3.85E-02	70429.21595	38.15641025	129998457	3.63E-03
RP11-68E19.1	5.16E-03	4.17E+68	132245231.4	1.32E+129	2.62E-02
RP11-689C9.1	8.80E-05	3025.717895	16.6818751	548797.3457	2.52E-03
RP11-687M24.8	7.03E-05	0.447652342	0.2954371	0.678291992	1.50E-04
RP11-685M7.3	5.17E-03	0.023987539	0.00133503	0.431003213	1.14E-02
RP11-680H20.1	6.67E-03	24.68243244	2.614851501	232.985495	5.12E-03
RP11-680A11.5	3.37E-05	5.901865227	2.378627588	14.64374387	1.29E-04
RP11-67L3.2	3.82E-09	1.33E+20	11379000.21	1.56E+33	2.54E-03
RP11-67L2.2	1.30E-02	0.140120154	0.03605397	0.544562982	4.55E-03
RP11-679B19.1	2.41E-04	14.86556001	2.393380939	92.33167644	3.77E-03
RP11-674P19.2	2.15E-02	46194556526	15.74558816	1.36E+20	2.73E-02
RP11-673E1.4	1.36E-02	5138467.315	3.438521961	7.67884E+12	3.32E-02
RP11-673C5.1	3.59E-02	2.360063177	1.100693456	5.060353696	2.73E-02
RP11-672L10.6	9.46E-05	0.211686383	0.092447035	0.484722144	2.40E-04
RP11-670E13.6	7.42E-03	4.201475705	1.465538913	12.04498765	7.56E-03
RP11-669N7.2	2.73E-02	2.17991E+16	531.0047176	8.95E+29	1.87E-02
RP11-668G10.2	5.67E-03	2.00E+23	10629270.13	3.76E+39	5.01E-03
RP11-667K14.8	3.58E-03	45009.86987	10.63364057	190516914	1.19E-02
RP11-667K14.3	1.74E-02	0.136480224	0.042647761	0.436760357	7.92E-04
RP11-666F17.1	4.59E-03	11465.22543	30.37374426	4327796.83	2.02E-03
RP11-666A20.4	3.79E-02	0.062103666	0.004522775	0.852765187	3.76E-02
RP11-666A20.3	4.03E-06	0.281756472	0.138479032	0.573276028	4.74E-04
RP11-663N22.1	1.46E-03	2.72896E+16	733127.4165	1.02E+27	2.31E-03
RP11-662J14.1	1.17E-03	8998410170	660.3391907	1.22621E+17	6.25E-03
RP11-661A12.8	9.20E-04	116.382324	11.77317926	1150.483233	4.71E-05
RP11-661A12.4	2.15E-03	0.00479968	2.49E-05	0.923827736	4.66E-02
RP11-65J3.14	1.01E-02	5.59E+31	100546827.3	3.11E+55	8.78E-03
RP11-65J21.3	3.16E-03	9.1112748	1.205445378	68.866935	3.23E-02
RP11-65F13.3	1.88E-04	2.33E+59	6.91617E+12	7.87E+105	1.24E-02

RP11-65E22.2	3.04E-03	4431749.647	3.969721701	4.94755E+12	3.12E-02
RP11-65B23.5	2.14E-03	0.001948504	7.95E-06	0.477320627	2.62E-02
RP11-659E9.2	4.99E-03	1.85911E+12	24797.01026	1.39E+20	2.26E-03
RP11-655M14.12	1.01E-03	0.083595033	0.008094691	0.86329792	3.72E-02
RP11-654C20.1	5.16E-03	4.43E+78	2032485420	9.67E+147	2.62E-02
RP11-64K7.1	1.63E-03	209.8255784	12.84730053	3426.927957	1.76E-04
RP11-64C12.9	5.97E-03	1.07872E+15	1114.533609	1.04E+27	1.40E-02
RP11-64B16.2	3.49E-05	2.336453334	1.261459337	4.327538763	6.96E-03
RP11-646E18.2	2.16E-03	7.82683E+14	3609.429535	1.70E+26	1.00E-02
RP11-644C3.1	1.73E-02	1.1998E+13	124.3452663	1.16E+24	1.96E-02
RP11-643N23.1	7.23E-05	1.35996E+14	8423.623733	2.20E+24	6.65E-03
RP11-642D6.1	9.45E-03	1.59435E+17	51.45009487	4.94E+32	2.95E-02
RP11-642D21.1	1.43E-07	1.16E+34	1.37204E+15	9.72E+52	4.19E-04
RP11-642A1.2	1.89E-02	52.9097867	3.311143946	845.461742	5.00E-03
RP11-642A1.1	4.08E-03	1.17142E+15	6998227.934	1.96E+23	3.29E-04
RP11-640L9.2	2.31E-04	368.1885216	1.202287215	112754.0789	4.31E-02
RP11-63P12.7	2.76E-06	3.22E+21	9525589.127	1.09E+36	3.71E-03
RP11-63N8.3	2.70E-03	191666.4366	24.85903429	1477773532	7.73E-03
RP11-63N3.2	5.16E-03	4.64666E+12	31.56350731	6.84E+23	2.62E-02
RP11-63H19.5	3.12E-02	301452.0785	1.292278647	70320248500	4.54E-02
RP11-63E9.1	6.67E-05	0.406779529	0.225577138	0.733538809	2.79E-03
RP11-638I2.9	4.22E-02	1.810036881	1.273720963	2.572175231	9.35E-04
RP11-638I2.10	3.39E-03	3.647484427	1.606437998	8.281765409	1.98E-03
RP11-632L2.2	1.01E-05	77466.70246	9.624921304	623494966.9	1.41E-02
RP11-632C17__A.1	2.67E-02	0.489449288	0.300003298	0.798526574	4.23E-03
RP11-630D6.5	3.34E-02	3.24459E+17	8176.717479	1.29E+31	1.16E-02
RP11-629O1.2	1.31E-03	4.065340077	2.097313476	7.88007617	3.28E-05
RP11-629G13.1	1.62E-02	378.741606	1.123197237	127711.5001	4.56E-02
RP11-627G18.2	7.08E-03	9846040371	383.0055492	2.53115E+17	8.21E-03
RP11-625L16.3	1.48E-02	5856926.59	35.04005381	9.78982E+11	1.11E-02
RP11-625L16.1	1.55E-02	5.68E-13	4.49E-25	0.718104816	4.73E-02
RP11-620J15.4	2.38E-04	0.000284421	2.24E-06	0.03604858	9.50E-04
RP11-620J15.2	7.84E-05	1.41E-07	7.42E-12	0.002680989	1.70E-03

RP11-61K12.2	3.77E-03	8.21E-07	5.39E-12	0.12503091	2.14E-02
RP11-61G19.1	4.15E-02	261374079.5	1.752171042	3.89896E+16	4.36E-02
RP11-619A14.3	4.70E-05	0.243960137	0.10904275	0.545809309	5.95E-04
RP11-617J18.1	1.88E-02	4.62E+21	1.327078193	1.61E+43	4.87E-02
RP11-616M22.5	1.88E-02	108145.1552	1.067962687	10951107884	4.87E-02
RP11-616M22.11	1.02E-02	14.13928215	3.38356786	59.08535246	2.83E-04
RP11-613M10.9	1.64E-02	266916957.8	1.541517836	4.62172E+16	4.50E-02
RP11-613M10.6	1.89E-03	0.350422206	0.169518916	0.7243777	4.65E-03
RP11-612B6.2	1.14E-03	2.150074694	1.465944853	3.153475508	8.95E-05
RP11-60E8.3	5.16E-03	5.32159E+17	125.2904002	2.26E+33	2.62E-02
RP11-603K19.1	4.44E-02	450732898.9	59.81496584	3.39648E+15	1.36E-02
RP11-5O17.1	2.69E-03	0.048922876	0.004976983	0.48090331	9.66E-03
RP11-599J14.2	1.12E-08	0.597307929	0.481926737	0.740313277	2.53E-06
RP11-599B13.7	3.37E-02	0.051994783	0.004937344	0.547553013	1.38E-02
RP11-598P20.5	2.23E-03	3.08156E+16	51625.47536	1.84E+28	6.06E-03
RP11-598F7.6	2.71E-02	19.12264901	3.265003505	111.9985644	1.07E-03
RP11-598F7.5	4.29E-04	5.859704706	2.485969982	13.81196857	5.31E-05
RP11-595O22.1	1.08E-02	8.55E+51	1.30454E+15	5.60E+88	5.70E-03
RP11-58E21.3	3.54E-02	24.15595516	1.620512666	360.0775126	2.09E-02
RP11-589P10.5	2.82E-06	0.149421378	0.061056143	0.365675708	3.14E-05
RP11-588H23.5	5.16E-03	5.76E+50	1017782.398	3.27E+95	2.62E-02
RP11-586K2.1	3.98E-02	4.35E+21	1640.812057	1.15E+40	2.13E-02
RP11-57B24.2	2.76E-06	2.82E+21	9120794.922	8.70E+35	3.71E-03
RP11-579O24.3	1.16E-02	842650844.6	1.208940249	5.87341E+17	4.79E-02
RP11-576N17.5	3.87E-02	407.5481765	5.49034482	30252.29228	6.24E-03
RP11-574K11.24	4.15E-05	0.439194856	0.246574944	0.78228598	5.21E-03
RP11-573D15.1	9.32E-03	0.142267039	0.039352921	0.514317866	2.94E-03
RP11-572O6.1	7.54E-04	0.037785329	0.004134687	0.345305704	3.71E-03
RP11-572M11.4	2.60E-05	0.000391632	3.73E-06	0.0411399	9.55E-04
RP11-572C15.3	3.30E-03	323.2822022	3.375167605	30964.79775	1.30E-02
RP11-56P9.10	1.17E-02	4.53E+91	6.32E+21	3.25E+161	1.01E-02
RP11-567P19.1	3.21E-03	3.79E-06	9.80E-11	0.146551718	2.05E-02
RP11-567G24.3	1.99E-03	7.87E+25	29474.22138	2.10E+47	1.78E-02

RP11-566K11.4	3.87E-03	1459644236	26329.4244	8.09194E+13	1.53E-04
RP11-565N2.2	2.76E-06	1.23141E+17	351172.537	4.32E+28	3.71E-03
RP11-565N2.1	2.76E-06	5.51613E+16	270614.6429	1.12E+28	3.71E-03
RP11-565F19.2	2.06E-03	65.78911044	6.192652634	698.9261804	5.16E-04
RP11-563N6.6	1.50E-02	91841.26845	58.26061227	144777376.4	2.35E-03
RP11-562A8.1	1.34E-03	4150.711317	20.68517322	832886.6406	2.07E-03
RP11-561P12.5	2.76E-06	1.55E+33	58790150327	4.07E+55	3.71E-03
RP11-560J1.2	2.06E-03	0.076859667	0.015031401	0.393004515	2.06E-03
RP11-559N14.5	2.72E-03	1736.82079	46.86573128	64365.71831	5.18E-05
RP11-557H15.4	4.03E-04	1.764655364	1.20145247	2.591869951	3.78E-03
RP11-557H15.3	1.26E-03	2.189497053	1.076994413	4.451181256	3.04E-02
RP11-556N21.1	1.92E-03	397.0867979	2.636714894	59800.9005	1.93E-02
RP11-556E13.1	1.43E-04	14.14597291	2.557818323	78.23407466	2.40E-03
RP11-555G19.2	1.99E-03	5.21E+57	9151783161	2.97E+105	1.78E-02
RP11-554A11.7	9.15E-03	0.070671413	0.006527273	0.765166149	2.92E-02
RP11-553N16.5	2.42E-02	511568.7212	19.06870674	13724190114	1.15E-02
RP11-553E24.3	1.99E-03	6.11056E+17	1174.208053	3.18E+32	1.78E-02
RP11-552J9.9	3.02E-02	8.28996E+11	1.137993845	6.04E+23	4.89E-02
RP11-552E10.1	1.37E-02	22477700779	94.67146001	5.33685E+18	1.54E-02
RP11-551L14.4	1.27E-02	7.55E-05	2.92E-08	0.195287851	1.79E-02
RP11-551L14.1	3.35E-05	0.009709875	0.000642513	0.146739001	8.23E-04
RP11-550I24.2	1.26E-02	0.00361751	0.000106413	0.122976916	1.78E-03
RP11-548H18.2	1.23E-06	1.10E-09	2.08E-14	5.84E-05	2.02E-04
RP11-547D24.1	1.93E-02	6979953.953	259.9115088	1.87447E+11	2.46E-03
RP11-546K22.3	3.19E-03	6.969134622	2.176577146	22.31431928	1.08E-03
RP11-546D6.3	1.26E-02	2.903549907	1.425115724	5.915731555	3.33E-03
RP11-546B8.6	2.19E-05	1.92E+124	3.49E+44	1.05E+204	2.25E-03
RP11-545H22.1	7.55E-03	105613.6063	23.90887677	466531068.7	6.91E-03
RP11-543B16.2	2.87E-02	13.89557718	2.745589142	70.32627795	1.47E-03
RP11-541P9.3	9.56E-03	3.89E-10	1.60E-17	0.009470484	1.25E-02
RP11-541H12.1	2.62E-02	7.34E-12	3.32E-22	0.162591696	3.49E-02
RP11-540O11.6	1.15E-02	114.4652583	7.49202619	1748.832029	6.55E-04
RP11-53I6.3	2.82E-02	0.00946744	0.000368973	0.242924249	4.88E-03

RP11-53B5.1	5.60E-03	321193.9213	1.198956581	86046097664	4.68E-02
RP11-536C10.7	1.88E-02	9.68E+56	2.105042077	4.45E+113	4.87E-02
RP11-535M15.2	8.60E-03	0.006688786	0.000172032	0.260067634	7.34E-03
RP11-535M15.1	4.70E-02	0.36999587	0.196478192	0.696753885	2.08E-03
RP11-534I8.1	2.98E-05	5.02456E+17	366951.5236	6.88E+29	4.25E-03
RP11-533F5.2	3.01E-02	424530.9967	2.175265365	82852680884	3.71E-02
RP11-532F6.4	2.66E-03	0.019119238	0.000652695	0.560055561	2.17E-02
RP11-532F12.5	1.93E-04	0.291412185	0.139094837	0.610526338	1.08E-03
RP11-530I17.2	5.28E-04	9.71E+27	2098333.564	4.50E+49	1.13E-02
RP11-529K1.3	4.45E-02	8.42428E+18	37.52828738	1.89E+36	3.25E-02
RP11-527H14.6	1.73E-02	217.1961219	19.03405681	2478.407826	1.48E-05
RP11-527H14.4	4.02E-03	2.439723157	1.610618949	3.695628372	2.56E-05
RP11-523J2.1	1.88E-02	2.08E+81	2.892573472	1.49E+162	4.87E-02
RP11-522L3.9	1.99E-03	1.50E+25	22152.49014	1.02E+46	1.78E-02
RP11-521D12.1	4.82E-03	14.28456289	1.944709624	104.925041	8.96E-03
RP11-520P18.5	1.52E-02	3.921916532	1.597770295	9.626808893	2.86E-03
RP11-51F16.9	7.06E-04	0.145578536	0.046980594	0.451103489	8.39E-04
RP11-519G16.3	2.08E-04	5.84E-18	2.38E-30	1.44E-05	6.41E-03
RP11-515O17.2	3.07E-03	6.82E-33	1.36E-60	3.42E-05	2.29E-02
RP11-515C16.1	1.48E-03	227100560.2	53.51780213	9.63692E+14	1.35E-02
RP11-514O12.4	6.00E-03	20.02625519	2.209213337	181.5356127	7.71E-03
RP11-513O17.2	3.30E-02	649.5003371	21.87882504	19281.23138	1.81E-04
RP11-513I15.6	2.03E-04	9223.863637	81.65011317	1042002.97	1.54E-04
RP11-513H8.1	2.42E-02	4.55609E+12	191.5569706	1.08E+23	1.68E-02
RP11-513G11.4	4.65E-03	0.000328635	5.49E-07	0.19664548	1.40E-02
RP11-513G11.2	1.78E-04	1.15E-18	3.12E-32	4.20E-05	9.53E-03
RP11-510I5.4	5.16E-03	2.10446E+17	112.2622184	3.95E+32	2.62E-02
RP11-510I21.1	2.76E-06	2.00E+43	1.12289E+14	3.56E+72	3.71E-03
RP11-50C13.1	1.44E-03	3.525882334	2.051758512	6.05911766	5.08E-06
RP11-507K2.3	1.36E-02	0.357160254	0.130275773	0.979180122	4.54E-02
RP11-507K13.6	2.56E-02	16.0815786	1.271972542	203.3197744	3.19E-02
RP11-506N2.1	7.23E-05	2.64E+77	3.19E+21	2.19E+133	6.65E-03
RP11-506H21.5	1.34E-02	0.006563019	0.000271185	0.158833207	1.99E-03

RP11-504P24.3	3.06E-02	0.146299627	0.030686616	0.69748913	1.59E-02
RP11-504I13.3	6.32E-03	0.002418752	1.51E-05	0.388330344	2.01E-02
RP11-500C11.3	9.54E-04	0.151011861	0.039240061	0.581155616	5.97E-03
RP11-4M23.4	2.81E-02	1392.864943	1.337379397	1450652.488	4.12E-02
RP11-4L24.1	2.76E-06	3.39E+72	3.43E+23	3.36E+121	3.71E-03
RP11-4K3__A.3	3.88E-02	0.1124648	0.013521242	0.935441549	4.32E-02
RP11-4B16.3	7.99E-07	2.102398728	1.480720977	2.985086645	3.26E-05
RP11-499E18.1	6.96E-03	0.178072458	0.041172619	0.770167186	2.09E-02
RP11-498P14.5	2.57E-04	0.004320269	0.000144614	0.129066231	1.68E-03
RP11-497E19.2	6.43E-03	2.60366E+14	430.2913298	1.58E+26	1.65E-02
RP11-496H1.1	1.81E-03	0.028973634	0.003426742	0.244976575	1.15E-03
RP11-496B10.3	3.75E-02	0.000563666	1.82E-06	0.174539376	1.06E-02
RP11-495P10.5	3.44E-02	1.42E+22	91879037.02	2.18E+36	2.21E-03
RP11-495K9.5	4.62E-03	0.0762826	0.016511194	0.352429688	9.82E-04
RP11-493L12.5	2.54E-02	61.34506703	2.330190782	1614.982463	1.36E-02
RP11-490G8.1	1.84E-02	0.368819707	0.143313628	0.949162887	3.86E-02
RP11-48B3.4	1.17E-03	2.165354121	1.481461642	3.164954349	6.62E-05
RP11-48B3.3	8.08E-03	5.762613985	1.988635574	16.6987458	1.25E-03
RP11-489O18.1	2.35E-02	3.005862318	1.351215348	6.68672709	6.98E-03
RP11-489C13.1	9.83E-04	4.19945E+11	254.1497806	6.94E+20	1.35E-02
RP11-488I20.9	2.77E-03	6.76E+73	1.90502E+18	2.40E+129	9.19E-03
RP11-488C13.1	5.81E-03	2.511095264	1.131097864	5.574760265	2.37E-02
RP11-486M3.2	2.76E-06	5.25E+42	7.27402E+13	3.78E+71	3.71E-03
RP11-485G7.6	8.45E-04	0.004268942	0.000145139	0.125561809	1.56E-03
RP11-485G7.5	4.07E-06	1.04E-06	5.87E-10	0.00185198	3.09E-04
RP11-483P21.2	1.04E-03	158.0894019	4.372315689	5716.02345	5.68E-03
RP11-483M24.2	2.76E-06	5.95E+23	51806467.58	6.83E+39	3.71E-03
RP11-483L5.1	3.57E-02	1.704939958	1.098810683	2.645424099	1.73E-02
RP11-481J2.3	3.09E-02	0.028093013	0.001514689	0.521042597	1.65E-02
RP11-480I12.5	3.16E-05	0.003889616	9.94E-05	0.152235422	3.02E-03
RP11-479O9.3	2.15E-02	0.119698231	0.016343351	0.876666376	3.67E-02
RP11-478H16.1	1.81E-02	9347.315457	7.613397599	11476125.49	1.18E-02
RP11-478C6.6	2.71E-02	0.160719448	0.035471145	0.728218411	1.77E-02

RP11-477H21.2	1.48E-02	0.035562559	0.003069931	0.411962181	7.60E-03
RP11-476K15.1	1.77E-03	504068912.6	602.195898	4.21932E+14	3.98E-03
RP11-475O6.1	1.75E-04	5.94E-05	6.31E-08	0.055888259	5.34E-03
RP11-474P2.4	1.17E-02	0.000912351	1.57E-06	0.528697975	3.11E-02
RP11-474D1.2	2.39E-03	2.11E-05	2.95E-08	0.015135381	1.33E-03
RP11-473C18.3	3.74E-03	1114728.142	2393.833762	519091530.1	8.90E-06
RP11-472N13.3	1.51E-02	3.762855701	1.72522801	8.207079262	8.66E-04
RP11-472E5.3	1.99E-03	1.11E+20	2881.023089	4.26E+36	1.78E-02
RP11-46J23.1	3.94E-03	0.045468539	0.007926774	0.260810766	5.24E-04
RP11-46H11.3	1.57E-03	0.001081208	6.58E-06	0.177618594	8.69E-03
RP11-46D6.1	6.31E-04	0.037810337	0.00455258	0.31402446	2.43E-03
RP11-469J4.3	3.10E-02	147463.4988	1.63696266	13284043675	4.09E-02
RP11-467P22.5	2.76E-06	8.01E+70	1.02E+23	6.31E+118	3.71E-03
RP11-467J12.4	7.98E-03	6.348331755	1.218634121	33.07089092	2.82E-02
RP11-467J12.1	1.76E-03	329.6514226	2.887709811	37631.91855	1.65E-02
RP11-466F5.6	1.10E-05	6.79E-11	3.40E-17	0.000135763	1.56E-03
RP11-466F5.10	6.35E-03	2.60E-06	1.76E-10	0.038315262	8.64E-03
RP11-466C23.5	1.46E-02	59.87881193	3.581146428	1001.207906	4.40E-03
RP11-466A19.5	6.21E-04	7.25895E+11	85161.5797	6.18734E+18	7.96E-04
RP11-463O9.6	1.88E-02	33297464874	1.147333797	9.66E+20	4.87E-02
RP11-463J10.4	7.23E-05	1.95E+20	431810.0485	8.79E+34	6.65E-03
RP11-463D19.2	1.12E-02	8.89936E+15	1291359.521	6.13E+25	1.49E-03
RP11-462G12.1	2.21E-02	5.600615651	1.66675415	18.81914958	5.33E-03
RP11-460I13.2	3.56E-02	122.2368383	2.015556406	7413.260478	2.18E-02
RP11-460B17.2	1.88E-02	4.83E+21	1.32740724	1.76E+43	4.87E-02
RP11-45J1.1	3.06E-05	9978889.608	385.365705	2.58399E+11	1.88E-03
RP11-45A16.4	4.71E-03	107.3647453	3.603274878	3199.086641	6.93E-03
RP11-459E5.1	2.14E-02	126878.0826	16.82047822	957050545	9.89E-03
RP11-457P14.5	4.49E-03	248.3745696	6.745650997	9145.140602	2.72E-03
RP11-457D2.3	5.25E-05	6.38264E+12	4221.013875	9.65E+21	6.26E-03
RP11-456O19.3	1.88E-02	3.51532E+17	1.257569042	9.83E+34	4.87E-02
RP11-456K23.1	4.04E-05	241.0628779	16.34074671	3556.221275	6.49E-05
RP11-456J20.2	5.16E-03	1.39579E+11	20.84562904	9.35E+20	2.62E-02

RP11-455O6.2	1.44E-03	0.03953738	0.002843227	0.549799396	1.62E-02
RP11-455F5.3	3.53E-02	0.134671818	0.029555107	0.613650244	9.57E-03
RP11-453D21.1	2.62E-02	33838.74676	6.95058244	164743141	1.61E-02
RP11-452L6.8	3.00E-03	0.034085528	0.003005663	0.386544762	6.39E-03
RP11-452K12.4	3.01E-03	0.004116698	2.53E-05	0.670332976	3.45E-02
RP11-452K12.3	1.01E-04	0.010480836	0.000952144	0.115368973	1.96E-04
RP11-452I5.2	4.88E-04	0.309419793	0.141930055	0.674561907	3.18E-03
RP11-452H21.2	2.72E-02	3483856.754	2.954806774	4.10763E+12	3.47E-02
RP11-452G18.2	7.03E-03	0.492053267	0.345955842	0.69984775	7.96E-05
RP11-452F19.3	4.62E-02	0.519413193	0.276084692	0.977200375	4.22E-02
RP11-452D12.1	3.19E-02	5.56E+22	6.319465423	4.89E+44	4.22E-02
RP11-450H6.3	5.44E-05	5982986.769	764.0661089	46849520297	6.47E-04
RP11-44N21.3	3.93E-03	1206265557	465.7497196	3.12416E+15	5.51E-03
RP11-44N21.1	2.48E-02	3.35096937	1.484788606	7.562689849	3.59E-03
RP11-44M6.7	1.11E-03	0.024736408	0.003847282	0.1590447	9.76E-05
RP11-44K6.4	2.79E-02	118.4294589	3.564633166	3934.636773	7.56E-03
RP11-44K6.2	3.10E-02	16.63731681	2.759609513	100.3041587	2.16E-03
RP11-44D19.1	4.38E-03	0.023526752	0.000703833	0.786419599	3.62E-02
RP11-449J1.1	5.02E-03	1227.852277	6.50192464	231873.0681	7.81E-03
RP11-447M12.2	2.76E-06	8.9386E+13	33645.68368	2.37E+23	3.71E-03
RP11-447G11.1	4.58E-03	9517427265	1.250751818	7.24216E+19	4.78E-02
RP11-446H18.6	3.52E-03	1.83E-06	9.06E-12	0.370049845	3.41E-02
RP11-446H18.5	1.81E-03	1.39E-17	5.77E-30	3.32E-05	7.61E-03
RP11-445P17.8	1.12E-02	2742857.945	12.23960308	6.14666E+11	1.84E-02
RP11-445P17.5	9.78E-03	545024.7823	50.77902331	5849896157	5.28E-03
RP11-444B24.2	2.76E-06	1.60E+22	16039842.56	1.60E+37	3.71E-03
RP11-441F2.5	2.80E-02	4.51E-05	1.91E-08	0.106464943	1.16E-02
RP11-440J4.2	2.76E-06	2.86E+59	1.96016E+19	4.16E+99	3.71E-03
RP11-43P5.1	5.28E-04	4.79E+20	46912.69038	4.89E+36	1.13E-02
RP11-43N5.1	1.19E-02	39165324367	37670.33819	4.07196E+16	5.59E-04
RP11-43F13.3	3.71E-03	3.18375088	1.523027555	6.655342271	2.08E-03
RP11-439H13.2	2.31E-03	1.56E+22	10673220499	2.28E+34	3.49E-04
RP11-438F14.3	2.76E-06	7.53E+77	1.86E+25	3.05E+130	3.71E-03

RP11-438B23.2	2.30E-02	4.72E-06	3.72E-10	0.060008375	1.10E-02
RP11-436F23.1	3.12E-02	10547266617	1.59531612	6.97322E+19	4.55E-02
RP11-435O5.5	4.94E-04	0.009907371	0.000734489	0.133638468	5.09E-04
RP11-435O5.2	1.54E-02	0.024611169	0.002276294	0.266094643	2.29E-03
RP11-433O3.1	7.23E-05	5.34511E+14	12319.65715	2.32E+25	6.65E-03
RP11-433J20.1	8.62E-05	0.000197935	1.92E-07	0.204563884	1.60E-02
RP11-432M24.4	3.23E-02	8313910.028	1.540061397	4.4882E+13	4.40E-02
RP11-432J24.5	2.43E-04	2.329470931	1.518635389	3.573230849	1.07E-04
RP11-432I13.5	7.23E-05	4725621879	486.4307557	4.59089E+16	6.65E-03
RP11-430H10.1	4.99E-05	0.028252789	0.003041047	0.262481978	1.71E-03
RP11-42O15.2	6.97E-04	6.89E+24	1741471.565	2.73E+43	8.85E-03
RP11-42A4.1	5.28E-04	1.37E+31	10801365.01	1.74E+55	1.13E-02
RP11-429O1.1	3.39E-03	1604762294	75.16280702	3.42625E+16	1.38E-02
RP11-429K17.1	1.34E-02	3.36993E+12	1223.688793	9.28E+21	9.29E-03
RP11-429J17.7	1.78E-02	53.74441291	4.624343684	624.6209445	1.45E-03
RP11-429E11.2	4.42E-05	1.871150186	1.335671869	2.621304753	2.70E-04
RP11-428K3.1	2.92E-02	0.000848358	2.46E-06	0.292650707	1.77E-02
RP11-428J1.5	2.81E-04	0.249873705	0.100164231	0.623344958	2.95E-03
RP11-428C19.4	6.33E-03	1439118064	280.1199352	7.39348E+15	7.48E-03
RP11-426C22.5	3.98E-02	45749.2199	30.92351422	67682835.36	3.96E-03
RP11-424I19.1	2.55E-03	24194462055	211.3800478	2.76929E+18	1.16E-02
RP11-422P22.1	1.88E-02	8.9241E+18	1.280853956	6.22E+37	4.87E-02
RP11-422N16.3	4.20E-03	5.71E-16	1.48E-28	0.002210202	1.76E-02
RP11-420L9.5	1.00E-05	0.172904556	0.072501452	0.41235016	7.57E-05
RP11-420K14.6	1.57E-02	2291462500	11367.8744	4.61898E+14	5.43E-04
RP11-41O4.1	2.60E-02	2.289343819	1.200341844	4.366335428	1.19E-02
RP11-419N10.6	1.88E-02	1.42351E+18	1.267585835	1.60E+36	4.87E-02
RP11-419K12.1	9.56E-04	3.952768758	1.402751236	11.13838323	9.32E-03
RP11-419C5.3	1.78E-03	2670.758349	11.52617317	618848.0817	4.51E-03
RP11-419C23.1	7.38E-06	7.01E-05	8.24E-08	0.059678773	5.45E-03
RP11-417O11.5	1.33E-05	2.54058E+17	978274001.7	6.60E+25	5.03E-05
RP11-417J1.1	7.36E-03	31948794332	799.633724	1.27649E+18	6.76E-03
RP11-417E7.2	3.02E-02	2677.558179	23.45257456	305694.2761	1.09E-03

RP11-417E7.1	1.12E-02	5.126660505	2.364488906	11.11557253	3.48E-05
RP11-416N13.1	1.65E-02	2151104.306	83.03069501	55729387017	4.92E-03
RP11-416H1.1	3.60E-02	1240.626224	1.069267558	1439446.485	4.79E-02
RP11-415F23.3	2.13E-03	0.05635798	0.007124704	0.44580403	6.42E-03
RP11-414H23.3	5.28E-04	3.49E+26	990190.4195	1.23E+47	1.13E-02
RP11-414H17.5	3.22E-03	22.4270901	3.895274473	129.1242437	4.97E-04
RP11-414B7.1	3.82E-09	4.84E+47	5.24323E+16	4.47E+78	2.54E-03
RP11-413E6.1	3.66E-06	0.233597906	0.119042355	0.458391315	2.36E-05
RP11-411K7.1	2.47E-02	9.10883E+19	221238684.6	3.75E+31	7.57E-04
RP11-411B10.5	1.12E-03	3.569E+16	174455.5792	7.30E+27	4.13E-03
RP11-410N8.1	3.18E-02	1206623.945	1.611123432	9.03681E+11	4.25E-02
RP11-40E6.1	4.30E-04	2.23E-06	9.64E-10	0.005163701	9.94E-04
RP11-409K20.7	5.30E-03	9.782665019	1.62317793	58.95874576	1.28E-02
RP11-408E5.5	1.36E-02	8.778513102	1.149715766	67.02725541	3.62E-02
RP11-408B11.2	1.03E-02	4.613540246	1.38764827	15.33872384	1.26E-02
RP11-407H12.8	7.44E-03	3.25E+23	35420237.25	2.99E+39	3.89E-03
RP11-404P21.8	2.78E-02	2666338284	221.5401865	3.20906E+16	9.08E-03
RP11-404J23.1	5.16E-03	3.06E+24	790.531389	1.18E+46	2.62E-02
RP11-404I7.1	5.16E-03	1.09E+21	309.0137969	3.87E+39	2.62E-02
RP11-404F10.6	1.47E-02	542816.9565	20.23112439	14564205258	1.11E-02
RP11-403P17.5	4.52E-02	4042561.036	7.659088382	2.13371E+12	2.36E-02
RP11-403I13.8	7.04E-04	57.22158261	6.967061749	469.9699292	1.65E-04
RP11-403I13.10	3.82E-09	2.83655E+17	1316464.219	6.11E+28	2.54E-03
RP11-403B2.3	1.99E-03	7.02E+27	63983.0541	7.70E+50	1.78E-02
RP11-403A3.3	1.04E-02	4.384758746	1.492118984	12.88510465	7.20E-03
RP11-403A3.2	4.78E-02	1045.06352	16.19787407	67425.99407	1.08E-03
RP11-403A21.1	2.23E-02	2589456044	14.13614443	4.74336E+17	2.56E-02
RP11-402G3.6	1.76E-02	0.000447115	7.79E-07	0.256467834	1.73E-02
RP11-401F2.2	2.45E-02	35618.23899	2.310793303	549014464.9	3.32E-02
RP11-400K9.2	2.42E-03	1.96E+62	2.16E+27	1.78E+97	4.79E-04
RP11-400D2.3	7.23E-05	5.23348E+18	158135.0636	1.73E+32	6.65E-03
RP11-3L21.2	1.34E-03	957.0582744	3.210110887	285336.1061	1.82E-02
RP11-3B7.7	1.68E-02	287.7343984	1.676911123	49371.18189	3.10E-02

RP11-398C13.2	5.87E-05	0.032133332	0.00574944	0.179591592	9.01E-05
RP11-397G5.2	1.92E-02	111965.5875	61.64106925	203375654.3	2.39E-03
RP11-397E7.4	7.78E-04	0.000916886	8.43E-06	0.099705121	3.46E-03
RP11-397D12.4	8.56E-03	68166.92685	6.972104683	666474490.5	1.76E-02
RP11-397C18.2	4.67E-04	1.33959E+15	595277.198	3.01E+24	1.52E-03
RP11-396O20.2	2.02E-05	0.063421883	0.010336581	0.389135925	2.89E-03
RP11-396M11.1	9.71E-03	6.435975184	1.584000876	26.15009701	9.24E-03
RP11-395G23.3	4.72E-05	1.764470149	1.179226906	2.640166104	5.75E-03
RP11-394O4.3	2.77E-02	5.52E-05	1.66E-08	0.183896017	1.78E-02
RP11-394I13.3	6.99E-03	0.060334386	0.009195563	0.395868961	3.44E-03
RP11-391L3.4	5.16E-03	4.88E+43	148127.2702	1.61E+82	2.62E-02
RP11-391J2.3	7.23E-05	5.83302E+16	45354.76706	7.50E+28	6.65E-03
RP11-38L15.8	2.99E-02	0.020272683	0.000675158	0.608718991	2.47E-02
RP11-38C17.1	2.71E-02	86.39797375	2.741488468	2722.83103	1.13E-02
RP11-389C8.2	2.92E-06	28.1609572	3.937401183	201.4119145	8.83E-04
RP11-387H17.4	6.66E-05	0.000145609	3.68E-07	0.057600906	3.79E-03
RP11-387D10.4	1.03E-02	5628.075523	59.61161854	531360.0749	1.98E-04
RP11-383J24.1	1.68E-03	20.42258794	3.078227327	135.4942484	1.78E-03
RP11-383H13.1	3.42E-02	0.476277684	0.266463384	0.851300576	1.23E-02
RP11-381O6.1	1.47E-02	1466022421	2295.839095	9.36138E+14	1.97E-03
RP11-380M21.3	5.28E-04	57464.4348	11.88694001	277797420.1	1.13E-02
RP11-380D23.1	3.82E-03	264.9727521	3.76541955	18646.14512	1.01E-02
RP11-380D15.2	5.16E-03	2.36E+49	697316.4019	8.00E+92	2.62E-02
RP11-37L2.1	2.66E-03	0.0028276	5.83E-05	0.137025518	3.04E-03
RP11-379F4.9	1.80E-03	0.000260523	7.69E-07	0.088214165	5.49E-03
RP11-379B18.5	4.66E-03	0.004251104	0.000122694	0.147292301	2.54E-03
RP11-378I6.3	1.48E-06	8.53E+39	8.27729E+18	8.80E+60	1.96E-04
RP11-378A13.1	1.02E-02	4.33511222	1.444530492	13.00990049	8.90E-03
RP11-377D9.3	1.53E-02	193987677.2	48968.93915	7.68471E+11	6.34E-06
RP11-374F3.5	1.41E-03	4417.778637	25.14119633	776286.3721	1.46E-03
RP11-373N22.3	3.74E-02	2.92E-06	2.59E-10	0.032945905	7.43E-03
RP11-371A19.2	3.97E-05	0.033399255	0.004327777	0.257755947	1.11E-03
RP11-370I10.12	1.96E-02	0.01369108	0.00021382	0.87665048	4.32E-02

RP11-370F5.4	3.31E-02	0.171052107	0.042967826	0.680947266	1.22E-02
RP11-370A5.1	6.11E-04	6016.503436	20.62522249	1755050.817	2.66E-03
RP11-368M16.5	5.16E-03	2.03514E+15	64.83468381	6.39E+28	2.62E-02
RP11-368L12.1	2.73E-04	0.138341834	0.025185345	0.759904754	2.29E-02
RP11-368J21.3	9.08E-03	18.08126603	1.892347716	172.7653848	1.19E-02
RP11-368E13.1	1.88E-02	79611.3275	1.066108574	5944951217	4.87E-02
RP11-367O10.1	2.12E-03	4.15E-07	2.66E-13	0.648154652	4.34E-02
RP11-367G18.1	7.58E-03	2.35E-06	9.34E-10	0.005938777	1.18E-03
RP11-363D14.1	3.35E-02	6.439869878	1.425141674	29.10021144	1.55E-02
RP11-361D14.2	5.16E-03	1.61E+139	2.98924E+16	8.71E+261	2.62E-02
RP11-359M6.2	1.99E-03	7.21163E+12	165.6535816	3.14E+23	1.78E-02
RP11-359I18.5	3.44E-02	0.013556684	0.000516799	0.35561939	9.87E-03
RP11-357H14.17	3.80E-03	5.28722E+11	6640.40468	4.20979E+19	3.64E-03
RP11-357H14.16	5.90E-03	5.33E+33	5.98913E+13	4.74E+53	9.21E-04
RP11-355N15.3	1.83E-02	17260.01401	2.475775829	120329183.3	3.07E-02
RP11-353N4.6	7.54E-05	4.913117021	1.927763437	12.52161878	8.53E-04
RP11-353N14.7	6.55E-05	2.715967553	1.766256766	4.176334886	5.34E-06
RP11-353K11.1	3.48E-02	0.193275097	0.064708645	0.577283964	3.24E-03
RP11-352M15.1	9.46E-03	4.44969E+15	10.63490608	1.86E+30	3.59E-02
RP11-351A11.1	1.20E-02	8.809205659	1.980893887	39.17529598	4.27E-03
RP11-350N15.5	2.47E-02	6.095272723	2.290148947	16.22267827	2.96E-04
RP11-350G8.3	8.56E-03	0.005008798	6.61E-05	0.379637991	1.65E-02
RP11-349J5.2	7.23E-05	4.89579E+12	3345.951882	7.16E+21	6.65E-03
RP11-349H17.2	6.59E-03	1.13E-18	1.27E-35	0.100693491	3.80E-02
RP11-348P10.2	2.64E-04	0.025646769	0.002376286	0.276800286	2.54E-03
RP11-348F1.3	3.89E-03	0.048119909	0.005492016	0.421616673	6.15E-03
RP11-347P5.1	3.84E-02	314265188.8	5.868889694	1.68282E+16	3.12E-02
RP11-347C18.1	1.82E-02	160867.8077	14.44524644	1791485639	1.17E-02
RP11-347C12.9	5.16E-03	5710741782	14.27996066	2.2838E+18	2.62E-02
RP11-346L1.2	1.38E-02	2910.537153	2.914264669	2906814.406	2.36E-02
RP11-345P4.9	9.84E-04	0.364707352	0.172450261	0.771303283	8.30E-03
RP11-345J4.3	2.54E-02	2.48E+45	400336570.9	1.54E+82	1.56E-02
RP11-344B23.2	5.16E-03	4.32E+23	626.9879961	2.97E+44	2.62E-02

RP11-344A16.2	2.81E-03	4.74968E+11	74842.31832	3.01426E+18	7.67E-04
RP11-343H5.4	6.98E-04	1.595220021	1.073559923	2.370363183	2.08E-02
RP11-343B18.2	1.35E-04	22767.39904	64.56117973	8028887.659	8.01E-04
RP11-342M1.3	3.56E-02	0.035982071	0.002920389	0.443334545	9.46E-03
RP11-341G23.4	1.60E-02	1.92274474	1.275303638	2.898876178	1.80E-03
RP11-340I6.5	5.16E-03	2.67903E+15	66.97868914	1.07E+29	2.62E-02
RP11-340F14.6	7.49E-04	2.096925419	1.314752803	3.344428094	1.88E-03
RP11-33I11.3	1.74E-02	273.0447802	10.6955264	6970.52667	6.90E-04
RP11-33E12.2	1.86E-04	0.161442988	0.058902054	0.442494561	3.93E-04
RP11-338K17.5	1.91E-02	72.13498226	4.021289166	1293.976994	3.68E-03
RP11-338C15.5	5.28E-04	5.92E+97	1.22E+22	2.88E+173	1.13E-02
RP11-338C15.3	1.84E-02	48.06960669	1.782686513	1296.182515	2.12E-02
RP11-337A23.5	2.09E-03	27960.433	5.439163571	143732727.2	1.89E-02
RP11-336A10.7	3.43E-03	3217.100043	2.109473231	4906311.459	3.08E-02
RP11-335M9.1	1.85E-09	8.294E+12	488571.7651	1.41E+20	4.61E-04
RP11-335L23.5	6.69E-03	0.111685993	0.015296653	0.815456912	3.07E-02
RP11-335E6.3	2.40E-02	7.88E-17	3.25E-32	0.191427396	4.02E-02
RP11-334C17.3	3.02E-02	0.194387757	0.058226788	0.648955595	7.75E-03
RP11-331F9.4	1.88E-03	3.53E-05	1.03E-07	0.012100177	5.76E-04
RP11-330M2.3	1.99E-03	2.85E+20	3391.707978	2.40E+37	1.78E-02
RP11-330A16.1	4.51E-03	282.3702068	2.947324954	27052.64433	1.53E-02
RP11-32P22.1	2.34E-02	502819.8153	4.235105359	59698105533	2.77E-02
RP11-329B9.4	5.21E-04	0.018635963	0.000775408	0.447892323	1.41E-02
RP11-327P2.5	6.35E-03	0.236220051	0.075997664	0.734231942	1.26E-02
RP11-327L3.3	2.16E-02	179065.9961	4.749198319	6751588126	2.45E-02
RP11-327E2.5	1.59E-02	37.89612853	3.964886735	362.2087221	1.60E-03
RP11-326K13.4	2.85E-02	0.045990641	0.004684168	0.45155067	8.24E-03
RP11-326I19.2	4.10E-02	0.044857849	0.002079818	0.967501248	4.76E-02
RP11-326C3.2	4.15E-05	6.603904533	2.996363427	14.55482826	2.85E-06
RP11-326C3.13	3.25E-03	0.018747098	0.000827855	0.424535108	1.25E-02
RP11-325O24.6	1.38E-02	2.41501E+14	34.3680854	1.70E+27	2.82E-02
RP11-325O24.5	3.82E-09	1.14E+27	3070344592	4.25E+44	2.54E-03
RP11-325F22.2	2.97E-03	419.3672745	6.386047463	27539.55587	4.68E-03

RP11-324J13.2	2.76E-06	2.25E+34	1.40101E+11	3.60E+57	3.71E-03
RP11-324I22.3	1.62E-02	3.345689496	1.275098283	8.778647385	1.41E-02
RP11-322E11.6	1.05E-02	417.9320646	10.77339282	16212.83225	1.22E-03
RP11-322E11.5	8.46E-03	28.42184934	2.610663875	309.4237936	6.00E-03
RP11-322D14.2	2.52E-02	974.5229662	2.296434789	413551.9181	2.58E-02
RP11-321G12.1	3.40E-04	901588.5913	3494.994697	232578890.2	1.30E-06
RP11-321E2.10	4.88E-04	5602233.468	520.2722636	60324222574	1.04E-03
RP11-319G6.1	2.80E-03	0.056819685	0.009857735	0.327506952	1.33E-03
RP11-318K15.2	1.12E-03	2.86539E+14	782.9634929	1.05E+26	1.43E-02
RP11-317G22.2	1.82E-03	571321595.4	163.9106612	1.99138E+15	8.71E-03
RP11-317B3.2	8.33E-03	1.70345E+15	339907.9957	8.54E+24	2.09E-03
RP11-316O14.1	3.84E-02	14.9389767	1.346654075	165.7240926	2.76E-02
RP11-315A16.2	2.76E-06	1.4089E+12	8751.598966	2.27E+20	3.71E-03
RP11-315A16.1	9.93E-04	50.62014662	7.599133123	337.1962568	4.99E-05
RP11-314D7.3	5.97E-03	5576432.657	321.3387265	96772030929	1.81E-03
RP11-314D7.2	3.27E-02	3.83E+30	2354304370	6.23E+51	4.71E-03
RP11-314D7.1	5.97E-03	3.26853E+17	3555.244078	3.00E+31	1.40E-02
RP11-314A20.1	7.31E-03	0.230896031	0.080403898	0.663064584	6.46E-03
RP11-313P22.1	1.25E-02	2.425538058	1.473807081	3.991862263	4.91E-04
RP11-313I2.7	1.88E-02	8.71E+45	1.822219667	4.16E+91	4.87E-02
RP11-313H3.1	2.76E-06	7.41E+44	3.62622E+14	1.51E+75	3.71E-03
RP11-313D6.3	7.44E-05	0.081036308	0.020664276	0.31778918	3.13E-04
RP11-313C15.1	5.28E-04	5.82E+75	1.30046E+17	2.61E+134	1.13E-02
RP11-312J18.7	2.98E-02	3.08242E+11	1.656224342	5.74E+22	4.57E-02
RP11-311H10.7	1.99E-03	9612573074	52.84011678	1.7487E+18	1.78E-02
RP11-311B14.1	2.37E-02	5.43E+21	5.855134129	5.03E+42	4.22E-02
RP11-30L8.1	1.99E-03	21992446464	60.95360282	7.93501E+18	1.78E-02
RP11-30L15.4	1.39E-02	17.08519975	2.15076476	135.7210495	7.27E-03
RP11-308B16.2	2.76E-06	1.40E+22	15358230.75	1.28E+37	3.71E-03
RP11-307P5.2	6.75E-04	5.13808E+15	38516.36824	6.85E+26	5.64E-03
RP11-307C19.2	8.49E-04	0.666974878	0.46661161	0.953374236	2.63E-02
RP11-307C18.1	8.97E-05	3.952926502	1.839257846	8.495615754	4.30E-04
RP11-305P14.1	1.96E-02	71793096212	5.136640879	1.00E+21	3.60E-02

RP11-305O4.3	2.93E-02	299083.6741	118.2534141	756435193.2	1.61E-03
RP11-305L7.6	1.25E-02	4.791998332	1.376025881	16.68809311	1.38E-02
RP11-305L7.3	5.35E-03	19.74689708	2.609919049	149.4069114	3.86E-03
RP11-305L7.1	3.49E-02	3.827307097	1.041636843	14.06275106	4.32E-02
RP11-305B6.1	4.84E-02	31687.95272	23.27958963	43133335.4	4.88E-03
RP11-304M2.3	6.86E-04	1.32E-10	3.21E-17	0.000540805	3.41E-03
RP11-304L19.3	1.28E-03	3.280254479	1.67478863	6.4247328	5.33E-04
RP11-304L19.1	1.14E-02	4.431644409	2.021672921	9.71446566	2.01E-04
RP11-303E16.2	2.72E-02	3.361542332	1.440551029	7.844197548	5.04E-03
RP11-302F12.5	5.16E-03	3.76E+24	810.0556704	1.74E+46	2.62E-02
RP11-302F12.10	2.09E-03	40942341.72	859.0800257	1.95124E+12	1.43E-03
RP11-302B13.1	2.51E-02	3.266378655	1.227594818	8.691165327	1.78E-02
RP11-301G19.1	5.16E-03	1.59E+72	350899682.7	7.20E+135	2.62E-02
RP11-2N1.2	1.46E-05	222670212.1	118.7104166	4.17672E+14	9.10E-03
RP11-2K6.1	1.62E-02	17.42213107	3.133397272	96.86950771	1.10E-03
RP11-2E11.5	8.21E-03	6.44E-25	8.40E-49	0.49319504	4.71E-02
RP11-299J3.8	2.33E-02	0.056478753	0.009503653	0.335644584	1.57E-03
RP11-299H22.3	1.30E-02	8189593.429	97.91460691	6.84979E+11	5.91E-03
RP11-299H21.1	1.50E-02	0.049476118	0.005883058	0.416090772	5.66E-03
RP11-298E9.7	1.21E-02	6381747.352	89.44789519	4.55312E+11	5.99E-03
RP11-298D21.3	2.08E-03	0.221583412	0.079273872	0.619361802	4.06E-03
RP11-297N6.4	2.40E-02	0.01798464	0.001229113	0.263155047	3.33E-03
RP11-297L6.2	9.37E-03	2.65E+20	28.58231763	2.45E+39	3.48E-02
RP11-296A16.1	1.06E-02	1.39E+68	19966.91839	9.69E+131	3.64E-02
RP11-295P9.8	2.62E-02	4982587.251	104.7083976	2.37098E+11	5.01E-03
RP11-295P22.2	4.94E-03	50.11626037	2.527775819	993.6164174	1.02E-02
RP11-295M3.4	1.49E-03	14.33746762	3.102579709	66.25550254	6.50E-04
RP11-295G20.2	4.98E-02	4.927434265	1.644274108	14.76615628	4.40E-03
RP11-293G6__B.8	5.16E-03	1.59941E+16	82.75148753	3.09E+30	2.62E-02
RP11-291L19.1	2.21E-03	9609.386089	42.70643511	2162210.467	9.05E-04
RP11-291B21.2	1.05E-04	3.510639294	1.289192233	9.559930576	1.40E-02
RP11-290L1.3	3.96E-02	0.00011403	2.13E-08	0.610893865	3.82E-02
RP11-289I10.2	1.14E-02	1.789002283	1.063035759	3.010744596	2.85E-02

RP11-288E14.2	4.38E-02	11.2054901	1.321864322	94.98933152	2.67E-02
RP11-288C17.3	5.21E-04	298.9732532	1.760278362	50778.90411	2.96E-02
RP11-286H15.1	1.36E-02	0.001264283	7.31E-06	0.218758173	1.11E-02
RP11-286E11.1	7.89E-06	0.057610299	0.009235974	0.359349943	2.24E-03
RP11-286B14.2	1.99E-03	1.30861E+15	406.4891055	4.21E+27	1.78E-02
RP11-284M14.1	1.40E-02	0.082543177	0.014223296	0.479029331	5.43E-03
RP11-284H19.1	7.20E-03	19335076.81	21.5490324	1.73486E+13	1.64E-02
RP11-284A20.2	5.28E-04	4.81E+41	2602084473	8.89E+73	1.13E-02
RP11-283G6.4	1.29E-03	4.786697794	1.89145393	12.1136843	9.49E-04
RP11-282I1.1	4.13E-02	4.18E-07	4.62E-13	0.37854774	3.58E-02
RP11-281O15.8	6.70E-03	0.00161056	9.90E-06	0.262094957	1.33E-02
RP11-281O15.4	5.12E-06	0.010647268	0.000917355	0.123577363	2.82E-04
RP11-281O15.2	2.30E-02	3.96E-11	4.78E-21	0.32854848	3.98E-02
RP11-280H21.1	4.12E-04	7.41E+21	349839.0233	1.57E+38	8.65E-03
RP11-27M24.2	2.62E-02	2485.578379	15.41276747	400842.9952	2.57E-03
RP11-279O9.4	1.30E-02	3.085461785	1.203048045	7.913295284	1.90E-02
RP11-279O22.1	1.99E-03	6.11056E+17	1174.208053	3.18E+32	1.78E-02
RP11-279N8.1	1.14E-02	6706.209684	2.302744978	19530277.45	3.04E-02
RP11-279N21.1	3.82E-09	8501669.851	268.9472835	2.68746E+11	2.54E-03
RP11-278L15.3	4.92E-03	1.15928E+17	16.34217087	8.22E+32	3.49E-02
RP11-276H19.2	5.72E-03	4.46E-18	5.42E-34	0.036679671	3.26E-02
RP11-276H19.1	5.85E-05	0.00062254	3.47E-06	0.111829741	5.32E-03
RP11-275I4.2	1.59E-02	1035745480	302.1255203	3.55074E+15	6.85E-03
RP11-274M17.2	3.56E-03	23150592333	142.1179992	3.77116E+18	1.34E-02
RP11-274E7.2	3.08E-03	3.174978363	1.518891375	6.636740305	2.13E-03
RP11-273B19.2	1.52E-02	2.933241596	1.36330769	6.311052393	5.91E-03
RP11-26J3.3	6.55E-04	3.364829235	1.846032393	6.133194532	7.45E-05
RP11-269F21.2	5.28E-04	3.52431E+13	1148.316208	1.08E+24	1.13E-02
RP11-267M23.7	1.05E-05	1187.788677	16.3733698	86166.86475	1.20E-03
RP11-266J6.2	3.95E-04	0.026914062	0.003099849	0.233678063	1.04E-03
RP11-265O12.1	2.39E-02	3.94E-18	4.01E-32	0.00038784	1.48E-02
RP11-265D19.7	1.06E-02	1.08E+34	140.5557304	8.36E+65	3.64E-02
RP11-263K4.3	2.79E-02	3.54E+26	211572335	5.92E+44	4.30E-03

RP11-263E1.1	1.16E-03	1834166.738	115.2080203	29200810982	3.48E-03
RP11-262D11.2	3.29E-02	4834.528299	1.361727632	17163978.56	4.20E-02
RP11-262A16.1	1.63E-02	25.84959474	3.701604331	180.5167405	1.04E-03
RP11-260A9.6	2.02E-03	314.8602884	1.959068602	50604.14989	2.65E-02
RP11-25O10.2	8.53E-03	4.47E-16	3.97E-30	0.050241951	3.23E-02
RP11-25I15.2	4.24E-02	2.72E+24	300.1951213	2.46E+46	2.92E-02
RP11-259K5.2	6.69E-05	0.076095902	0.021961349	0.263671712	4.86E-05
RP11-258O13.1	2.07E-02	1.24E+23	15.46185507	9.94E+44	3.88E-02
RP11-257I8.1	5.51E-05	1737507494	8337.632786	3.62085E+14	6.62E-04
RP11-256I23.2	2.39E-02	0.100324168	0.016807152	0.598848561	1.17E-02
RP11-255M6.1	9.45E-03	1.10349E+17	49.61329616	2.45E+32	2.95E-02
RP11-255H23.2	6.85E-03	1.06E-06	8.08E-11	0.013949922	4.47E-03
RP11-254F7.2	2.31E-03	0.129760683	0.042223971	0.398774308	3.64E-04
RP11-254F19.2	2.59E-02	2439.850168	7.991012593	744945.4965	7.54E-03
RP11-253M7.1	1.41E-02	0.000556364	1.45E-06	0.213522883	1.36E-02
RP11-252M18.3	1.46E-13	9.98152E+16	55372531.96	1.80E+26	3.19E-04
RP11-246K15.1	1.43E-07	2.0141E+17	19204925.06	2.11E+27	7.13E-04
RP11-245G13.2	6.27E-03	2.407369656	1.380988342	4.196580438	1.95E-03
RP11-245A18.1	3.32E-06	7.52587E+16	10062904.23	5.63E+26	8.08E-04
RP11-244K5.6	4.24E-04	5.25947E+18	103438.6915	2.67E+32	7.43E-03
RP11-244B22.11	5.28E-04	1.52E+31	11049991.98	2.09E+55	1.13E-02
RP11-244B22.1	4.05E-02	31853628223	23.80752487	4.2619E+19	2.41E-02
RP11-243M5.4	4.06E-02	4.07E-07	3.19E-12	0.052031815	1.42E-02
RP11-242J7.1	4.39E-04	8762.707939	92.85417223	826942.3826	9.12E-05
RP11-242F24.1	3.81E-04	5.06E+39	1.31315E+11	1.95E+68	6.48E-03
RP11-242D8.1	1.73E-05	0.13484043	0.052748407	0.34469177	2.86E-05
RP11-241M13.2	5.16E-03	9.21159E+19	230.5817424	3.68E+37	2.62E-02
RP11-23E19.2	7.04E-03	5.82E-09	2.45E-16	0.13847021	2.87E-02
RP11-23E10.2	5.28E-04	2.00E+29	4153149.205	9.58E+51	1.13E-02
RP11-237P21.1	5.16E-03	5.71065E+12	32.34320791	1.01E+24	2.62E-02
RP11-236B18.5	6.71E-05	0.146867587	0.046276584	0.46611237	1.13E-03
RP11-235C23.5	2.75E-02	0.000393644	2.25E-06	0.068931396	2.93E-03
RP11-231N3.1	9.65E-03	0.424987484	0.201026935	0.898458515	2.51E-02

RP11-230L22.4	1.10E-02	2.00801E+14	923.777453	4.36E+25	1.34E-02
RP11-230C9.1	2.33E-03	3780752.007	14.17299467	1.00854E+12	1.75E-02
RP11-22P4.2	2.91E-02	3.24363E+13	456447.2148	2.31E+21	7.44E-04
RP11-22P4.1	3.72E-02	338.2452053	2.605583241	43909.48527	1.90E-02
RP11-229P13.25	1.68E-02	110.6464528	8.651106657	1415.152766	2.95E-04
RP11-227F8.2	1.06E-02	3.46463E+13	7.152310831	1.68E+26	3.64E-02
RP11-226P1.1	9.71E-05	3.06188E+13	3515.627805	2.67E+23	7.83E-03
RP11-226L15.5	2.95E-03	2.740928275	1.374171455	5.467067287	4.21E-03
RP11-226L15.1	5.98E-04	13979.17134	32.49112015	6014481.202	2.04E-03
RP11-225H22.4	1.44E-04	226.1070483	15.86464163	3222.537166	6.36E-05
RP11-225B17.2	1.19E-02	0.473268152	0.239330113	0.935873637	3.15E-02
RP11-223J6.2	5.16E-03	6.46E+35	17308.15431	2.41E+67	2.62E-02
RP11-223C24.1	4.39E-02	32.10877357	3.9108899	263.6160482	1.24E-03
RP11-222K16.2	3.32E-04	2.301016402	1.355705054	3.905478161	2.02E-03
RP11-221N13.2	5.28E-04	6.38E+19	29758.22651	1.37E+35	1.13E-02
RP11-220I1.5	3.78E-04	4.60E-16	2.33E-25	9.12E-07	1.22E-03
RP11-21J18.1	5.45E-03	1.28E+30	39770282.22	4.10E+52	8.75E-03
RP11-21B23.2	5.28E-04	1.04058E+19	19755.57983	5.48E+33	1.13E-02
RP11-219H23.1	1.99E-03	1.69E+25	22608.98816	1.27E+46	1.78E-02
RP11-219E7.4	1.51E-03	5.36E-11	4.09E-19	0.007008514	1.31E-02
RP11-219E7.3	4.40E-04	0.001127967	1.05E-05	0.12107624	4.44E-03
RP11-219E7.2	4.76E-03	3.88E-07	2.44E-12	0.061800085	1.57E-02
RP11-219B4.5	4.97E-03	15720.7872	14.07541448	17558498.94	6.97E-03
RP11-219B17.3	3.18E-03	0.006207401	0.000149048	0.258518756	7.56E-03
RP11-218M22.2	2.95E-02	75744647.31	26.68952243	2.14963E+14	1.67E-02
RP11-216B9.6	4.89E-03	5.869461217	1.650892374	20.86785033	6.25E-03
RP11-215P8.3	5.44E-03	37.42947597	3.14351392	445.6686712	4.15E-03
RP11-213H15.1	9.10E-03	3.72E-05	1.09E-08	0.126573379	1.40E-02
RP11-213G2.2	1.37E-02	4.82E-08	9.72E-14	0.023910814	1.18E-02
RP11-211G23.2	1.42E-02	1.926777503	1.264304126	2.936375408	2.28E-03
RP11-20I20.4	3.85E-04	2.618563344	1.710705272	4.008214682	9.34E-06
RP11-20G13.1	2.40E-02	1.92E-05	2.55E-09	0.143965506	1.70E-02
RP11-204I15.1	1.43E-04	1.83E+30	10564025516	3.17E+50	3.38E-03

RP11-204C16.4	3.14E-02	4.008768199	1.495662034	10.74455466	5.78E-03
RP11-203P23.2	3.27E-02	1.520807823	1.006016231	2.299024967	4.68E-02
RP11-1L9.1	3.11E-02	6.03465E+14	3.260694848	1.12E+29	4.23E-02
RP11-1K3.1	1.99E-03	3.45E+58	12683341901	9.39E+106	1.78E-02
RP11-1H8.1	7.23E-05	4.37E+41	3.67701E+11	5.20E+71	6.65E-03
RP11-1D12.1	7.23E-05	1.29E+33	1569830094	1.05E+57	6.65E-03
RP11-19O2.1	2.30E-02	102896.71	6.280202026	1685890500	1.97E-02
RP11-19E11.1	1.18E-02	4922618.232	722.3005878	33548595518	6.23E-04
RP11-197K6.1	9.95E-03	49.89796908	1.797678475	1385.012589	2.11E-02
RP11-196G18.3	5.64E-03	10097.30801	1.411640533	72224923.24	4.17E-02
RP11-196G11.6	1.58E-03	6372.213664	16.46617045	2465971.497	3.96E-03
RP11-196G11.5	5.13E-03	5.276591773	1.699612461	16.38162898	4.01E-03
RP11-195M16.3	1.98E-02	1.77231E+15	423088.4155	7.42E+24	1.90E-03
RP11-195F19.9	4.83E-03	0.211191643	0.079270054	0.562657751	1.87E-03
RP11-195F19.30	4.36E-03	7.57E-09	2.76E-14	0.002073769	3.42E-03
RP11-193M21.1	2.43E-02	0.000150634	7.81E-08	0.290618919	2.26E-02
RP11-192H6.2	2.76E-06	334.0566915	6.590722743	16931.96293	3.71E-03
RP11-191F9.4	5.16E-03	1.13514E+14	46.07312153	2.80E+26	2.62E-02
RP11-18O11.1	1.99E-03	1.0378E+17	864.6786625	1.25E+31	1.78E-02
RP11-18H21.1	7.29E-03	6569.299282	4.272918048	10099817.64	1.89E-02
RP11-18F14.4	2.08E-02	7.27163E+15	1.576096522	3.35E+31	4.72E-02
RP11-18B16.2	1.11E-02	2.404442579	1.417411804	4.078803421	1.14E-03
RP11-187C18.4	4.65E-02	0.026685905	0.000866637	0.821725608	3.82E-02
RP11-187C18.3	2.68E-02	4.902534027	2.036621497	11.80132878	3.90E-04
RP11-186B7.4	1.47E-02	17.72554784	1.12001583	280.5273263	4.13E-02
RP11-184C23.1	7.23E-05	1.57E+24	5248204.48	4.68E+41	6.65E-03
RP11-183E9.2	4.87E-04	6.18E+31	5652003654	6.77E+53	4.70E-03
RP11-182L21.6	4.59E-03	0.108193639	0.020942669	0.558948029	7.95E-03
RP11-181E10.3	3.92E-02	8231.484691	78.72857772	860644.7897	1.44E-04
RP11-180M15.3	3.13E-05	48749.93043	213.2656141	11143642.29	9.82E-05
RP11-17M16.2	3.90E-03	55.31005473	5.985286812	511.1203942	4.05E-04
RP11-17A4.2	2.98E-03	3.15E+27	7172.321906	1.38E+51	2.26E-02
RP11-179G5.1	3.67E-03	0.035806984	0.002836111	0.452076864	1.01E-02

RP11-179A18.1	9.76E-04	0.004814238	3.28E-05	0.706379266	3.60E-02
RP11-178L8.7	3.93E-03	0.371612851	0.159719852	0.864614569	2.16E-02
RP11-178L8.1	1.94E-02	22.71107115	1.188846132	433.859975	3.80E-02
RP11-177J6.1	3.20E-02	4.454325523	1.004122329	19.75956045	4.94E-02
RP11-177B4.1	3.37E-02	0.023878832	0.001201929	0.474402807	1.43E-02
RP11-175P19.2	3.04E-02	6.68E-05	1.23E-08	0.363535938	2.85E-02
RP11-175D17.3	3.49E-05	1.73E-05	3.93E-08	0.007628468	4.16E-04
RP11-174I12.2	1.85E-09	3.54E+36	9.30011E+18	1.35E+54	4.61E-05
RP11-174E22.2	2.92E-02	4.7557E+18	20.95557772	1.08E+36	3.49E-02
RP11-173D3.1	3.82E-09	1.37E+33	4.16059E+11	4.54E+54	2.54E-03
RP11-172C16.4	6.73E-03	2.10795E+14	27.70302174	1.60E+27	2.93E-02
RP11-171G2.1	3.75E-02	5656218917	13.90337317	2.30108E+18	2.64E-02
RP11-170M17.1	4.41E-02	59353690.55	5.362399796	6.56956E+14	3.05E-02
RP11-170L3.7	2.11E-02	19.50842695	1.976861592	192.5166252	1.10E-02
RP11-170K4.2	3.15E-03	2.95E-11	9.82E-20	0.0088676	1.49E-02
RP11-16P20.4	1.31E-03	8.90E-10	3.14E-15	0.000252077	1.14E-03
RP11-16F15.4	1.53E-09	1.46E+25	3.901712673	5.44E+49	4.47E-02
RP11-16E18.3	3.06E-03	4.789162962	2.05262659	11.17401577	2.91E-04
RP11-16C1.3	1.99E-03	1.18E+21	4332.36842	3.20E+38	1.78E-02
RP11-16C1.1	3.86E-02	0.092466602	0.009602076	0.890439959	3.94E-02
RP11-169D4.2	1.01E-05	15.13457551	4.294109281	53.34176682	2.37E-05
RP11-168L22.2	2.60E-03	2651468153	444.8695893	1.5803E+16	6.41E-03
RP11-168J18.6	5.22E-03	10857520.1	193.5217139	6.0916E+11	3.69E-03
RP11-168E17.1	3.11E-02	1.87E+24	7.032624944	4.95E+47	4.23E-02
RP11-168C9.1	2.76E-06	1.36E+28	1345023267	1.37E+47	3.71E-03
RP11-167P22.3	2.76E-06	8689575309	1678.887118	4.49755E+16	3.71E-03
RP11-167J8.3	3.39E-03	31549415.27	105.1913084	9.46243E+12	7.28E-03
RP11-166N17.3	8.23E-04	1823691.306	25.28987492	1.31509E+11	1.15E-02
RP11-164J13.1	1.44E-02	0.657634308	0.440504055	0.981790922	4.04E-02
RP11-164C12.2	1.80E-08	1.17E+121	1.03E+56	1.32E+186	2.65E-04
RP11-164C1.2	2.16E-03	329085.9393	5.949620339	18202431291	2.26E-02
RP11-163E9.2	6.67E-04	0.104591888	0.018398714	0.594577596	1.09E-02
RP11-161H23.8	4.32E-02	4.778117265	1.820787548	12.5387526	1.49E-03

RP11-160E2.6	1.67E-02	4.92E-07	6.55E-13	0.3688995	3.53E-02
RP11-15P13.1	1.14E-03	64056838.81	618.6535134	6.6326E+12	2.28E-03
RP11-15N24.4	1.27E-05	2.02E-05	7.62E-08	0.005375846	1.48E-04
RP11-15I11.3	4.98E-02	2.310879647	1.247709026	4.279976046	7.73E-03
RP11-15A1.2	2.67E-04	0.021017666	0.002116815	0.208682508	9.74E-04
RP11-159H10.3	7.41E-03	3.803739806	1.686986842	8.576496366	1.28E-03
RP11-159H10.1	6.12E-04	366915.073	27.89238259	4826646500	8.10E-03
RP11-159D12.6	1.32E-02	4.058569684	1.332308061	12.36349788	1.37E-02
RP11-158I3.3	5.28E-04	3.64471E+13	1157.062491	1.15E+24	1.13E-02
RP11-158I23.1	2.30E-06	2.86E-05	4.03E-08	0.020299521	1.79E-03
RP11-158I13.2	2.71E-04	3.12E+28	586357068.5	1.66E+48	4.64E-03
RP11-157P1.4	4.66E-03	4.068841998	1.964915497	8.425540553	1.58E-04
RP11-156K23.3	2.26E-02	2.997476414	1.264465117	7.105664466	1.27E-02
RP11-156K13.1	5.16E-05	1.31E-07	1.88E-12	0.009087442	5.33E-03
RP11-155D18.13	3.53E-05	0.055459341	0.010874498	0.282839593	5.03E-04
RP11-155D18.12	9.44E-03	1.99E-13	1.80E-24	0.022110408	2.42E-02
RP11-152N13.16	1.56E-02	5.608675323	1.879318775	16.73863918	2.00E-03
RP11-150O12.6	3.32E-05	3.078910902	1.599634764	5.926160493	7.62E-04
RP11-150C16.1	9.15E-04	9.84E-05	4.41E-08	0.219380529	1.90E-02
RP11-14I17.2	6.24E-07	4.91625E+11	124.4078248	1.94E+21	1.69E-02
RP11-149P24.1	3.96E-02	8.42851E+14	525.0029473	1.35E+27	1.65E-02
RP11-149I23.3	1.35E-05	0.000150978	6.27E-08	0.363627224	2.68E-02
RP11-148O7.2	5.16E-03	49426.66144	3.593694196	679800430.5	2.62E-02
RP11-148K1.1	6.42E-03	9.11E-38	1.74E-72	0.004771193	3.65E-02
RP11-148E17.1	5.16E-03	8.057405156	1.280118834	50.71543056	2.62E-02
RP11-148B18.3	3.42E-02	156543.1986	9.227265708	2655800082	1.61E-02
RP11-147K16.3	6.83E-04	1.36E+23	6177174.924	2.99E+39	5.53E-03
RP11-145O15.3	9.76E-05	4.85E-14	1.11E-23	0.000212307	6.80E-03
RP11-145O15.2	2.41E-06	0.000933413	1.47E-05	0.059258206	9.87E-04
RP11-145M4.3	1.15E-02	57874577.53	68.59005437	4.88331E+13	1.03E-02
RP11-145E17.2	8.62E-03	4.11198E+12	282.1611024	5.99E+22	1.50E-02
RP11-144O23.22	1.01E-04	4.50E+23	3.79871E+11	5.33E+35	1.23E-04
RP11-143M1.3	9.60E-07	6.31E+41	5.13863E+18	7.75E+64	3.88E-04

RP11-143A12.3	5.10E-03	33.76373351	2.689709586	423.8337501	6.40E-03
RP11-142G1.3	3.66E-05	3.77E-05	1.58E-08	0.090013483	1.03E-02
RP11-141M3.6	2.54E-02	6.76E-13	7.15E-24	0.063994812	2.98E-02
RP11-141M1.3	5.24E-05	4.37E-13	2.69E-20	7.10E-06	7.81E-04
RP11-141C7.4	2.34E-02	0.006567591	0.000142565	0.302551853	1.01E-02
RP11-141C7.3	3.87E-02	0.016435242	0.000731215	0.369408866	9.68E-03
RP11-140K17.3	1.38E-02	0.334207972	0.127102575	0.878778169	2.63E-02
RP11-1398P2.1	1.22E-02	3.555922804	1.708722395	7.400024151	6.92E-04
RP11-138A9.1	3.00E-02	64067.51703	20.82453816	197106255.5	6.92E-03
RP11-137N23.1	1.29E-03	0.00850334	0.000318289	0.227173584	4.45E-03
RP11-136O12.2	5.75E-03	4273.499995	24.77732585	737077.2097	1.47E-03
RP11-136B18.1	2.62E-04	138.0391397	2.311407612	8243.809523	1.82E-02
RP11-1360M22.2	1.99E-03	9.72E+43	39082225.46	2.42E+80	1.78E-02
RP11-135F9.4	3.19E-02	334.9427468	3.182557582	35250.46782	1.44E-02
RP11-134O21.1	2.18E-02	4.98E-18	9.45E-33	0.002622844	2.12E-02
RP11-134N1.2	4.64E-03	6299.397794	2.792520927	14210247.16	2.64E-02
RP11-133N21.10	3.20E-02	0.004352311	3.73E-05	0.507668879	2.51E-02
RP11-132M7.2	1.06E-02	9700.975918	8.722406706	10789331.08	1.03E-02
RP11-131H24.4	1.12E-02	29.09305718	2.125808195	398.1572645	1.16E-02
RP11-12M5.3	5.78E-04	2.794800029	1.402735832	5.568337977	3.48E-03
RP11-12K11.1	4.53E-02	561.9781594	1.140171493	276992.9379	4.53E-02
RP11-12D5.6	2.28E-03	4.66279E+12	172.9993708	1.26E+23	1.73E-02
RP11-129M16.4	9.65E-05	5.50142831	2.302875699	13.14257364	1.24E-04
RP11-129B9.1	7.87E-03	0.042001251	0.002764965	0.638020786	2.24E-02
RP11-128M1.1	1.35E-02	0.254945801	0.074867451	0.868165814	2.88E-02
RP11-1281K21.2	1.35E-02	1301803995	4.769767036	3.55299E+17	3.42E-02
RP11-127B20.3	2.07E-03	0.107331966	0.027297303	0.422025235	1.40E-03
RP11-127B16.1	7.55E-03	2136.793198	10.7116367	426254.6706	4.55E-03
RP11-125D12.2	1.04E-09	3.59E+40	6.97478E+18	1.85E+62	2.51E-04
RP11-1259L22.2	1.60E-04	2.62779E+15	494851.5314	1.40E+25	1.89E-03
RP11-124N2.1	7.90E-06	0.020715523	0.002451707	0.175034357	3.70E-04
RP11-124N14.3	3.13E-04	0.137919302	0.040576356	0.468788617	1.51E-03
RP11-123C21.2	4.11E-02	510952.7951	2.651172995	98474433518	3.43E-02

RP11-121M22.1	4.27E-05	0.000314132	4.63E-06	0.02130447	1.78E-04
RP11-120J4.1	6.83E-03	0.011843387	0.000685991	0.204471854	2.27E-03
RP11-120B7.1	4.34E-02	24.82884729	6.078709475	101.4148908	7.69E-06
RP11-11N7.4	3.41E-02	0.001203794	4.02E-06	0.360835929	2.09E-02
RP11-1191J2.5	5.59E-04	0.223991828	0.084553698	0.593378415	2.61E-03
RP11-118M12.2	1.89E-02	559.1018009	5.289042196	59102.35015	7.80E-03
RP11-117D22.1	5.16E-03	3.1784E+17	117.8763353	8.57E+32	2.62E-02
RP11-116D17.1	2.68E-02	5.4551E+15	6.218863274	4.79E+30	3.90E-02
RP11-1167A19.2	2.89E-04	0.000206751	1.43E-06	0.029939813	8.32E-04
RP11-1166P10.8	1.92E-02	12.25224346	2.714201695	55.30814824	1.12E-03
RP11-1166P10.7	5.28E-04	2.881E+13	1097.209411	7.56E+23	1.13E-02
RP11-115L11.1	1.81E-03	6415.322493	78.34456803	525325.0318	9.61E-05
RP11-115J23.1	7.46E-05	2.29E+65	3.99E+24	1.32E+106	1.67E-03
RP11-115H18.1	1.33E-02	2.33E-13	2.26E-25	0.240002265	3.93E-02
RP11-1151B14.1	0.00E+00	3.04E+84	4.74E+44	1.95E+124	3.19E-05
RP11-114N19.3	4.38E-04	0.107229187	0.021036443	0.546579977	7.21E-03
RP11-114H24.7	4.96E-03	208782968	24.70884568	1.76416E+15	1.86E-02
RP11-114H24.3	2.76E-06	5.16E+33	86899311015	3.06E+56	3.71E-03
RP11-1149M10.2	2.96E-04	45.60304458	8.496188171	244.7730245	8.37E-06
RP11-113E21.3	1.63E-02	3315194.516	2.158963739	5.09064E+12	3.88E-02
RP11-113D6.3	2.76E-06	2.64E+57	4.28884E+18	1.63E+96	3.71E-03
RP11-113D6.10	1.65E-05	1.15E+94	2.94E+39	4.48E+148	7.33E-04
RP11-1137G4.4	1.43E-04	9.76524E+17	41427.87857	2.30E+31	8.37E-03
RP11-1134I14.6	3.36E-02	1013661.267	2.817284799	3.64716E+11	3.41E-02
RP11-111M22.3	1.02E-03	0.455644383	0.264736754	0.784219798	4.55E-03
RP11-110I1.12	5.89E-04	0.053364932	0.013070631	0.217878993	4.45E-05
RP11-10N23.5	1.97E-02	14.89479109	2.82424289	78.55372583	1.45E-03
RP11-10J21.4	3.66E-03	8.945999525	2.268027331	35.28657103	1.75E-03
RP11-10H3.1	2.46E-02	1.08E+24	58590.74731	1.97E+43	1.45E-02
RP11-10C24.3	4.44E-03	0.159070243	0.037229839	0.6796522	1.31E-02
RP11-10A14.9	2.31E-04	0.062727255	0.012410819	0.317038582	8.09E-04
RP11-10A14.5	6.54E-06	0.094895992	0.024654342	0.365260173	6.16E-04
RP11-10A14.4	2.28E-05	0.145483498	0.054728053	0.386738557	1.11E-04

RP11-109E10.1	5.16E-03	5.13E+21	371.0631301	7.09E+40	2.62E-02
RP11-109A6.3	1.10E-02	0.001426452	2.73E-06	0.745542293	4.02E-02
RP11-1094M14.8	9.14E-05	2.485921081	1.38429123	4.464236633	2.30E-03
RP11-108K3.3	2.37E-02	1457.190303	2.935273082	723409.2091	2.15E-02
RP11-108H9.1	5.28E-04	3.88E+40	1473143151	1.02E+72	1.13E-02
RP11-1084J3.1	4.32E-03	42677102.73	99.52701682	1.82999E+13	7.93E-03
RP11-1081L13.4	3.61E-07	0.169341184	0.074173814	0.386611324	2.48E-05
RP11-107N15.1	2.92E-04	5.478941856	1.7620964	17.03584654	3.30E-03
RP11-107M16.2	7.84E-03	7.363509787	1.632602339	33.21156357	9.38E-03
RP11-1078H9.2	3.67E-05	22172925756	1259575.938	3.90321E+14	1.79E-06
RP11-1074O12.1	2.45E-02	119338845.9	109.5840439	1.29962E+14	8.74E-03
RP11-1072C15.6	8.26E-04	1.51E-05	5.72E-09	0.040047109	5.78E-03
RP11-1072A3.4	1.84E-04	0.046037599	0.008885946	0.238518259	2.45E-04
RP11-1070N10.7	1.72E-03	4.81368E+12	233.0320609	9.94E+22	1.60E-02
RP11-106M3.3	1.25E-02	4.871922274	1.151492327	20.61292645	3.14E-02
RP11-1060J15.9	1.87E-03	4.911381573	1.436118778	16.7964303	1.12E-02
RP11-1060J15.7	1.14E-03	20408.34364	4.195227953	99279585	2.20E-02
RP11-105N14.2	4.75E-03	0.012562699	0.000456919	0.345403342	9.63E-03
RP11-105C19.1	5.25E-05	3.24574E+14	31.71064038	3.32E+27	2.88E-02
RP11-104J23.2	5.28E-04	2.50138E+17	8510.651925	7.35E+30	1.13E-02
RP11-104F15.9	1.66E-02	5.43E-05	2.80E-08	0.105360035	1.10E-02
RP11-1042B17.3	9.47E-03	0.080382433	0.006941044	0.930888095	4.37E-02
RP11-1041F24.1	5.16E-03	2.59104E+11	22.4290476	2.99E+21	2.62E-02
RP11-103J17.2	2.75E-03	8.01E+57	59004290513	1.09E+105	1.60E-02
RP11-1038A11.2	1.43E-02	3.29E+27	1241424.755	8.70E+48	1.18E-02
RP11-1038A11.1	3.37E-02	17619526.14	318.8086835	9.73774E+11	2.75E-03
RP11-102K13.5	2.10E-02	4.37E-08	1.58E-13	0.012079792	8.03E-03
RP11-102F4.2	1.45E-02	2.27115E+12	18.4557656	2.79E+23	2.90E-02
RP11-1028N23.4	1.99E-03	4.76E+40	10486504.15	2.16E+74	1.78E-02
RP11-101O6.2	1.04E-03	1792.822103	64.76813476	49626.4267	9.79E-06
RP11-1017G21.3	1.61E-02	68825903.21	15.55033324	3.04624E+14	2.08E-02
RP11-100N3.2	7.49E-03	6865.018519	8.45375976	5574854.338	9.75E-03
RP11-100E13.1	2.24E-04	8.638543026	3.515084047	21.22976993	2.60E-06

RP11-1007O24.3	2.67E-02	78.61323815	1.359431903	4546.046917	3.50E-02
RP11-1007I13.2	1.99E-03	3.57E+45	72803294.94	1.76E+83	1.78E-02
RP1-93H18.6	5.16E-03	1.21E+24	708.3341554	2.07E+45	2.62E-02
RP1-92O14.3	4.22E-02	0.296502211	0.119756886	0.73410026	8.58E-03
RP1-90K10.4	4.63E-03	1.83599E+11	142.7490696	2.36E+20	1.54E-02
RP1-80N2.4	2.52E-04	4.84E-05	5.32E-08	0.043964299	4.25E-03
RP1-80N2.3	7.51E-04	0.006791469	0.00019506	0.236460401	5.85E-03
RP1-7G5.6	1.61E-03	4.04E-10	9.74E-18	0.016788447	1.57E-02
RP1-79C4.4	1.27E-02	383567317.5	40493.19863	3.6333E+12	2.33E-05
RP1-73A14.2	5.16E-03	1.71448E+14	48.37736534	6.08E+26	2.62E-02
RP1-71H24.1	1.69E-02	2.05122E+13	437.0447351	9.63E+23	1.45E-02
RP1-69M21.2	3.86E-02	2.58E+32	121.9879646	5.44E+62	3.62E-02
RP1-67A8.3	1.33E-06	0.011066898	0.000711194	0.172212161	1.30E-03
RP1-66N13.4	1.99E-03	5.10653E+16	765.0644827	3.41E+30	1.78E-02
RP1-66C13.1	1.06E-02	281.4191032	1.427483893	55479.93363	3.64E-02
RP1-59D14.5	3.87E-05	2.414070753	1.466119734	3.974939742	5.33E-04
RP1-49C23.1	7.23E-05	5.86E+41	3.98899E+11	8.62E+71	6.65E-03
RP1-47M23.3	1.17E-02	516.9775526	2.740156864	97536.67513	1.94E-02
RP1-40G4P.1	1.63E-03	0.014684361	0.001067261	0.202040979	1.60E-03
RP1-40E16.9	1.51E-02	0.0007413	3.04E-06	0.180947268	1.02E-02
RP1-40E16.12	1.25E-02	0.213785443	0.062952265	0.72601384	1.34E-02
RP1-313L4.4	9.79E-03	20.80528297	4.319877157	100.201877	1.54E-04
RP1-309I22.2	1.26E-02	1.48E-17	8.82E-32	0.002490488	2.04E-02
RP1-302G2.5	1.49E-04	141659.3902	133.8227243	149954971.8	8.44E-04
RP1-302D9.5	2.76E-06	1.23235E+12	8379.534727	1.81E+20	3.71E-03
RP1-301L19.1	2.22E-02	2.63E+66	2.52084E+18	2.75E+114	6.71E-03
RP1-29C18.9	3.38E-02	6.193444405	2.582463783	14.85354948	4.39E-05
RP1-290I10.7	8.13E-06	0.012059702	0.000951774	0.152805563	6.50E-04
RP1-290I10.3	6.33E-03	0.019488095	0.001106394	0.343264465	7.13E-03
RP1-28H20.3	2.50E-02	4.825183001	1.346579247	17.2900266	1.57E-02
RP1-278O22.1	7.08E-04	2.672732603	1.375414494	5.193706769	3.73E-03
RP1-266L20.2	3.88E-04	0.309925758	0.173509695	0.553594283	7.56E-05
RP1-257C22.2	1.01E-02	27.69477625	2.372714418	323.2587225	8.07E-03

RP1-249H1.3	3.46E-02	2766.20736	49.67349766	154043.9776	1.11E-04
RP1-239B22.5	8.37E-04	3.815426687	2.04209725	7.128691252	2.68E-05
RP1-232L24.2	4.63E-04	1594022896	3381.173827	7.51487E+14	1.48E-03
RP1-228P16.3	3.59E-04	8.98E-05	7.71E-08	0.10461574	9.69E-03
RP1-225E12.3	8.14E-03	4.26E+35	5.10149E+11	3.56E+59	3.51E-03
RP1-205F14P.1	5.28E-04	1.26E+46	25912298521	6.15E+81	1.13E-02
RP1-19N1.1	1.99E-03	2.09964E+15	441.0503321	1.00E+28	1.78E-02
RP1-199J3.5	5.98E-05	11.99573809	3.866505796	37.21647912	1.70E-05
RP1-197B17.7	1.70E-02	0.000440629	1.06E-06	0.182965268	1.20E-02
RP1-182O16.2	9.61E-04	0.0003653	1.19E-06	0.111855622	6.73E-03
RP1-178F10.1	1.85E-02	0.007132696	0.000290507	0.175125956	2.47E-03
RP1-172I22.1	3.11E-02	5.22995E+16	3.814772502	7.17E+32	4.23E-02
RP1-172B20.6	7.23E-05	1.01E+50	7.72571E+13	1.31E+86	6.65E-03
RP1-15D23.2	5.28E-04	5.96E+35	120531952.7	2.95E+63	1.13E-02
RP1-159A19.4	2.29E-04	0.105279846	0.025770111	0.430104697	1.72E-03
RP1-149A16.16	2.62E-03	6.265186587	1.681463583	23.34428374	6.25E-03
RP1-149A16.12	1.59E-03	1296.215305	13.72469118	122419.8122	2.01E-03
RP1-146A15.1	1.88E-02	9.19498E+17	1.264447054	6.69E+35	4.87E-02
RP1-144F13.3	7.43E-05	80946691.23	6814.168341	9.6158E+11	1.42E-04
RP1-131F15.2	3.48E-02	1.00E-22	5.89E-39	1.70E-06	7.89E-03
RP1-127L4.10	1.88E-02	1.69E+24	1.372245426	2.07E+48	4.87E-02
RP1-120G22.11	7.83E-05	0.34007706	0.175123885	0.660403389	1.45E-03
RP1-117B12.4	2.58E-02	5179.991728	5.198976809	5161075.975	1.52E-02
RP1-102E24.8	1.48E-03	15.75717671	4.406127591	56.35075533	2.23E-05
RP1-101A2.1	4.32E-04	6.283135821	2.181375851	18.09765875	6.62E-04
RORA-AS2	1.43E-02	1.86912E+19	45.81701761	7.63E+36	3.20E-02
ROR1-AS1	1.95E-03	2.74E+37	3.98398E+15	1.89E+59	7.79E-04
ROR1	2.72E-04	26.31981552	2.22508011	311.3293251	9.47E-03
ROPN1B	1.77E-04	0.332733151	0.177866359	0.622441199	5.74E-04
ROMO1	1.20E-06	5.173841742	2.565700303	10.43326781	4.37E-06
ROBO3	1.40E-02	0.122349287	0.034101773	0.438960993	1.27E-03
ROBO1	1.96E-05	0.368882394	0.190577514	0.714009843	3.08E-03
RNY4P19	1.65E-03	29416.90595	7.274817047	118952043.7	1.52E-02

RNY3P1	2.12E-02	999.1116793	2.677267332	372851.8761	2.23E-02
RNY1P4	8.79E-03	29.93563142	1.920259416	466.6775858	1.53E-02
RNU7-97P	8.72E-05	51.24874699	3.680071519	713.6910395	3.39E-03
RNU7-52P	7.23E-05	1324.297443	7.363972146	238154.5833	6.65E-03
RNU7-18P	2.35E-02	55.08712971	2.045748845	1483.364817	1.70E-02
RNU7-149P	5.16E-03	972.2536046	2.257410736	418743.9426	2.62E-02
RNU6ATAC17P	2.99E-02	11.30621331	2.76562556	46.22117369	7.36E-04
RNU6ATAC	1.96E-02	8.862381398	1.171070037	67.06840886	3.46E-02
RNU6-98P	1.06E-02	2785.870226	1.649684714	4704579.518	3.64E-02
RNU6-979P	4.04E-04	770.3004073	15.63396957	37953.42666	8.30E-04
RNU6-960P	5.28E-04	1074.688309	4.838568789	238697.6423	1.13E-02
RNU6-952P	5.16E-03	153888.6285	4.110719364	5760964900	2.62E-02
RNU6-927P	5.28E-04	61467.31421	12.06913258	313049068.9	1.13E-02
RNU6-871P	1.06E-02	1738.627493	1.601325169	1887702.521	3.64E-02
RNU6-851P	1.25E-02	14.48193806	1.200524491	174.6957531	3.54E-02
RNU6-824P	4.59E-02	168.0527106	2.425992453	11641.30312	1.78E-02
RNU6-806P	1.65E-03	7965.116921	1.964419337	32296102.14	3.41E-02
RNU6-8	6.00E-03	2.548003146	1.166953887	5.563476075	1.89E-02
RNU6-763P	1.06E-02	2604.445929	1.642689018	4129289.549	3.64E-02
RNU6-746P	5.16E-03	170623.4664	4.161250176	6996062734	2.62E-02
RNU6-727P	3.82E-09	5029885.181	223.7397857	1.13077E+11	2.54E-03
RNU6-722P	2.07E-02	0.420264025	0.182884721	0.965755089	4.11E-02
RNU6-711P	1.88E-02	21414.74729	1.058197057	433370513.2	4.87E-02
RNU6-64P	2.76E-06	4187837.146	140.8359099	1.24528E+11	3.71E-03
RNU6-62P	4.38E-03	614.6686659	6.804597553	55523.8669	5.20E-03
RNU6-5P	2.25E-02	7.752335701	1.065509148	56.40374737	4.31E-02
RNU6-596P	5.16E-03	344.8250353	1.996782013	59547.96478	2.62E-02
RNU6-577P	2.91E-02	404.3298115	2.521369598	64838.8069	2.05E-02
RNU6-574P	3.97E-02	11.56033653	2.153194164	62.06657205	4.31E-03
RNU6-562P	2.30E-03	1198.673639	5.129947883	280084.4231	1.08E-02
RNU6-552P	5.16E-03	478959.3352	4.701932769	48788882373	2.62E-02
RNU6-516P	9.79E-03	2.438511084	1.405024458	4.23219416	1.53E-03
RNU6-483P	2.85E-02	0.066073273	0.007640522	0.571384683	1.36E-02

RNU6-46P	1.99E-03	747281.1575	10.32094815	54106378635	1.78E-02
RNU6-425P	3.82E-09	2609503.562	177.7520549	38309030217	2.54E-03
RNU6-420P	1.88E-02	23374.24647	1.058722759	516051433.9	4.87E-02
RNU6-366P	1.88E-02	21414.74729	1.058197057	433370513.2	4.87E-02
RNU6-35P	8.58E-04	4416.441892	14.86846442	1311834.123	3.86E-03
RNU6-336P	2.76E-06	5475908.546	153.6401068	1.95168E+11	3.71E-03
RNU6-314P	7.08E-03	1156.587834	6.059296643	220767.4415	8.48E-03
RNU6-299P	2.85E-02	702.1034528	2.250491074	219040.7525	2.53E-02
RNU6-264P	5.28E-04	92065.22442	13.22231934	641037727.8	1.13E-02
RNU6-17P	5.28E-04	124641.8747	14.15881059	1097238841	1.13E-02
RNU6-178P	2.92E-02	0.001757746	3.27E-06	0.946185709	4.80E-02
RNU6-173P	2.98E-02	15.10638668	1.470200809	155.2188769	2.24E-02
RNU6-132P	1.37E-05	739.3709519	8.498222744	64327.49776	3.74E-03
RNU6-1329P	2.76E-06	5475908.546	153.6401068	1.95168E+11	3.71E-03
RNU6-1327P	3.69E-04	68550.44722	68.83507141	68266999.9	1.57E-03
RNU6-1322P	5.16E-03	138794.7513	4.060800768	4743887741	2.62E-02
RNU6-1315P	1.58E-02	26.97061859	1.67216373	435.0137811	2.02E-02
RNU6-1310P	9.83E-04	1137.861761	4.46154438	290197.581	1.28E-02
RNU6-1297P	1.99E-03	523942.3689	9.707462829	28278821224	1.78E-02
RNU6-128P	5.28E-04	124641.8747	14.15881059	1097238841	1.13E-02
RNU6-1286P	5.16E-03	138794.7513	4.060800768	4743887741	2.62E-02
RNU6-1266P	5.24E-03	109.9173016	5.803699979	2081.743238	1.74E-03
RNU6-1237P	9.37E-03	79054.96766	2.234087574	2797422977	3.48E-02
RNU6-1214P	7.23E-05	121216.4512	25.81830472	569108940.8	6.65E-03
RNU6-1152P	5.28E-04	124641.8747	14.15881059	1097238841	1.13E-02
RNU6-1147P	3.04E-02	817.2673278	1.751388454	381369.355	3.25E-02
RNU6-1146P	2.68E-03	155.6663928	4.399581507	5507.802458	5.53E-03
RNU6-1104P	9.60E-07	1022164.853	487.1595827	2144720176	3.91E-04
RNU6-1092P	2.76E-06	374389.6799	64.33401837	2178748289	3.71E-03
RNU6-1045P	1.05E-03	7004.833826	16.58578358	2958418.979	4.10E-03
RNU6-1019P	5.16E-03	138794.7513	4.060800768	4743887741	2.62E-02
RNU6-1011P	4.40E-02	3.270738976	1.527444224	7.003681892	2.29E-03
RNU4ATAC15P	5.16E-03	986329.722	5.12160771	1.89949E+11	2.62E-02

RNU4-61P	5.97E-03	4455.899488	5.446237809	3645643.277	1.41E-02
RNU4-53P	1.81E-04	52.66961579	2.920124262	949.9898562	7.23E-03
RNU2-71P	4.52E-03	332.8428314	2.027006534	54654.16542	2.57E-02
RNU2-68P	1.88E-02	36343717.18	1.103792778	1.19666E+15	4.87E-02
RNU2-31P	5.28E-04	15819572.16	42.2843648	5.91847E+12	1.13E-02
RNU2-13P	1.99E-03	1520022.877	11.66651885	1.98043E+11	1.78E-02
RNU2-10P	7.23E-05	86013.54793	23.47174437	315201559.4	6.65E-03
RNU1-83P	1.41E-02	11732.00025	2.698124312	51013153.59	2.84E-02
RNU1-78P	1.06E-02	137840.545	2.110232807	9003753418	3.64E-02
RNU1-61P	1.00E-02	66.59767631	4.121711507	1076.070094	3.10E-03
RNU1-47P	2.87E-02	7.092328633	2.269788178	22.16115404	7.52E-04
RNU1-106P	4.26E-04	3.53739124	1.780922364	7.026211269	3.08E-04
RNU1-101P	3.82E-09	69326060.86	561.3391905	8.56185E+12	2.54E-03
RNPEPL1	1.61E-02	2.483757739	1.320683435	4.67110614	4.76E-03
RNPEP	3.29E-02	0.213811571	0.061495655	0.743392157	1.53E-02
RNMTL1	3.88E-03	8.794789322	1.851789618	41.76949608	6.24E-03
RNFT1P2	2.76E-06	3.32E+44	2.79458E+14	3.95E+74	3.71E-03
RNF5	3.61E-02	0.298770571	0.124652488	0.716101663	6.75E-03
RNF44	4.28E-02	0.132136556	0.042279636	0.412966412	4.99E-04
RNF43	3.45E-09	0.105102435	0.032602358	0.338825854	1.62E-04
RNF26	3.02E-03	0.23592355	0.076645147	0.726202818	1.18E-02
RNF224	1.55E-02	0.004255992	6.39E-05	0.283303352	1.08E-02
RNF219-AS1	2.21E-02	2.29E-07	5.97E-13	0.08768164	1.98E-02
RNF216-IT1	2.06E-02	451.6333668	1.155432187	176533.6818	4.47E-02
RNF215	1.69E-02	5.679842303	1.3012307	24.79238201	2.09E-02
RNF208	2.49E-06	0.21324258	0.110087656	0.413056282	4.63E-06
RNF149	5.98E-03	4.146766776	1.69689705	10.13359926	1.81E-03
RNF148	9.13E-03	1.23E-06	5.20E-12	0.290420389	3.11E-02
RNF145	2.76E-02	1.861868788	1.143344672	3.031942569	1.25E-02
RNF144B	5.82E-03	0.389808251	0.196393678	0.773703483	7.07E-03
RNF139	1.25E-03	2.091452924	1.297404986	3.371480287	2.46E-03
RNF135	1.47E-04	11.82480081	3.250496296	43.01678927	1.78E-04
RNF125	4.00E-05	5.171726739	2.098268873	12.74705916	3.57E-04

RNF123	1.86E-04	0.181969092	0.071033061	0.466159701	3.85E-04
RNF122	1.22E-02	0.309134094	0.147482023	0.647969741	1.88E-03
RNF115	2.81E-02	4.540128701	1.670520962	12.33912599	3.02E-03
RNF112	1.26E-02	9.88E-05	7.35E-08	0.132890158	1.21E-02
RNASET2	1.47E-02	3.511951226	1.495514816	8.247194401	3.93E-03
RNASEH2C	2.45E-06	7.122771238	3.021927022	16.78858217	7.19E-06
RNASEH2B	1.65E-03	4.600760518	1.484428399	14.25935893	8.18E-03
RNASE7	1.49E-02	3.51598E+12	11.92994322	1.04E+24	3.20E-02
RNASE6	2.39E-03	1.863826424	1.257044374	2.763505418	1.95E-03
RNASE4	1.36E-02	0.003584668	4.59E-05	0.280249544	1.13E-02
RNASE3	6.04E-03	0.000210711	7.10E-07	0.062507121	3.56E-03
RNA5SP92	1.88E-02	30395.19002	1.060301342	871325480.9	4.87E-02
RNA5SP75	2.76E-06	9361901.014	182.8432363	4.79346E+11	3.71E-03
RNA5SP74	7.94E-03	13.40299945	1.190973457	150.8349268	3.56E-02
RNA5SP67	7.23E-05	1046166.775	46.97933374	23296731448	6.65E-03
RNA5SP515	1.61E-02	0.027115993	0.001468529	0.500689605	1.53E-02
RNA5SP48	5.28E-04	848410.4502	21.83608083	32963804154	1.13E-02
RNA5SP454	1.88E-02	134593.6023	1.069288948	16941573948	4.87E-02
RNA5SP453	1.99E-03	3091542.217	13.18730028	7.2476E+11	1.78E-02
RNA5SP441	2.88E-02	2460.955858	18.56782029	326172.0353	1.74E-03
RNA5SP424	1.88E-02	47086.46402	1.062937255	2085856982	4.87E-02
RNA5SP401	1.88E-02	134593.6023	1.069288948	16941573948	4.87E-02
RNA5SP385	3.69E-04	112447.7704	83.23441002	151914347.2	1.57E-03
RNA5SP345	4.98E-02	8.24112767	1.395484983	48.66851746	1.99E-02
RNA5SP332	1.99E-03	2120.336646	3.750703171	1198662.568	1.78E-02
RNA5SP301	2.49E-02	39.64492472	1.399296127	1123.221901	3.10E-02
RNA5SP288	1.64E-06	450174.3569	33.26908859	6091448854	7.32E-03
RNA5SP209	1.88E-02	138.1410846	1.028350747	18556.85845	4.87E-02
RNA5SP199	5.16E-03	29491.341	3.38063771	257270748.4	2.62E-02
RNA5SP18	1.69E-02	16.51559809	4.073587799	66.95939645	8.62E-05
RNA5SP138	1.99E-03	1710961.822	11.90723299	2.4585E+11	1.78E-02
RNA5SP124	1.71E-02	0.000129178	2.04E-08	0.817374836	4.50E-02
RNA5SP122	3.44E-02	5.056708233	1.50142594	17.03067562	8.90E-03

RNA5SP101	3.93E-03	0.219833513	0.070179658	0.688615116	9.31E-03
RN7SL847P	7.49E-03	34787229.38	5.818114534	2.07997E+14	2.92E-02
RN7SL801P	2.76E-06	1.19828E+18	734782.587	1.95E+30	3.71E-03
RN7SL79P	1.88E-02	2.08813E+11	1.159345399	3.76E+22	4.87E-02
RN7SL751P	4.32E-02	0.000689087	5.54E-07	0.857110552	4.52E-02
RN7SL749P	3.30E-02	367.0801382	10.66105403	12639.25945	1.07E-03
RN7SL693P	8.16E-03	3.88E-05	1.72E-08	0.087375335	9.91E-03
RN7SL681P	5.16E-03	2.68078E+13	38.83878675	1.85E+25	2.62E-02
RN7SL672P	2.81E-03	118391712.2	314.5446549	4.45616E+13	4.54E-03
RN7SL645P	7.23E-05	7.6381E+11	1997.21809	2.92E+20	6.65E-03
RN7SL62P	6.64E-04	63853342.15	149.5824743	2.72575E+13	6.59E-03
RN7SL615P	5.55E-16	1.63E+22	5.06708E+12	5.24E+31	4.67E-06
RN7SL57P	1.37E-05	1.74297E+14	847418.4435	3.58E+22	7.86E-04
RN7SL531P	5.03E-04	290.7583996	7.416798692	11398.50904	2.44E-03
RN7SL526P	1.20E-02	0.061456648	0.008127231	0.464724008	6.88E-03
RN7SL51P	2.76E-06	1.23141E+17	351172.537	4.32E+28	3.71E-03
RN7SL494P	2.95E-02	664607.3584	1.597279664	2.76535E+11	4.23E-02
RN7SL493P	3.31E-03	814960.4444	24.18336071	27463533044	1.05E-02
RN7SL48P	2.30E-02	1058170.785	5.746013775	1.9487E+11	2.49E-02
RN7SL480P	4.70E-04	728962.5854	46.3348784	11468389887	6.18E-03
RN7SL460P	4.33E-02	90533270.04	5.262273656	1.55755E+15	3.11E-02
RN7SL455P	3.57E-06	127640883	1491.591051	1.09227E+13	1.28E-03
RN7SL42P	1.99E-03	2.99327E+15	468.8861991	1.91E+28	1.78E-02
RN7SL368P	1.63E-03	3944379.534	395.2901367	39358760726	1.23E-03
RN7SL285P	3.42E-02	2195425.237	1.140269891	4.22697E+12	4.80E-02
RN7SL282P	5.47E-03	1312570.774	973.8173896	1769163352	1.27E-04
RN7SL233P	1.01E-03	6032.216181	78.2344205	465110.2651	8.62E-05
RN7SL22P	2.76E-06	1.44658E+16	175279.9548	1.19E+27	3.71E-03
RN7SL217P	4.64E-03	6233008640	638.1644903	6.08783E+16	6.02E-03
RN7SL187P	5.33E-05	41882444594	22803.03029	7.69257E+16	8.89E-04
RN7SL184P	7.23E-05	1.1803E+13	4272.330352	3.26E+22	6.65E-03
RN7SL170P	8.92E-03	860366.8555	5.73656163	1.29037E+11	2.46E-02
RN7SL149P	9.83E-04	32244283.71	36.4828889	2.84981E+13	1.33E-02

RN7SL135P	7.23E-05	2289883.732	58.39810697	89790025398	6.65E-03
RN7SL130P	5.18E-03	26.99792353	4.598907761	158.4915186	2.63E-04
RN7SKP79	5.28E-04	1.32613E+15	2605.817374	6.75E+26	1.13E-02
RN7SKP72	7.23E-05	4544060.91	70.64214132	2.92297E+11	6.65E-03
RN7SKP42	5.16E-03	6.11545E+13	42.82087623	8.73E+25	2.62E-02
RN7SKP34	2.76E-06	5.09373E+14	59178.1866	4.38E+24	3.71E-03
RN7SKP29	3.69E-04	275847.5788	11.31179214	6726775545	1.51E-02
RN7SKP25	1.99E-03	1.56605E+16	623.8824008	3.93E+29	1.78E-02
RN7SKP238	4.12E-04	63568334334	543.069894	7.44091E+18	8.68E-03
RN7SKP237	6.74E-03	37218.11857	207.5480103	6674062.296	7.03E-05
RN7SKP230	1.61E-02	15764.62171	23.24305173	10692369.51	3.66E-03
RN7SKP221	2.76E-06	1.04818E+18	703555.0411	1.56E+30	3.71E-03
RN7SKP213	3.37E-05	1.11983E+13	193875.2821	6.47E+20	9.84E-04
RN7SKP185	1.60E-02	3376.213902	2.385494529	4778388.788	2.82E-02
RN7SKP17	2.12E-04	59409538.5	1337.819017	2.63824E+12	1.04E-03
RMDN1	2.04E-03	2.446638494	1.501057899	3.987880764	3.31E-04
RLN2	1.34E-02	0.012821803	0.000332929	0.493795287	1.93E-02
RLN1	2.09E-02	0.001980743	4.36E-06	0.90082817	4.62E-02
RIPK4	7.96E-04	0.525984812	0.31207357	0.886521798	1.59E-02
RIOK3P1	1.88E-02	2.06E+58	2.141890149	1.99E+116	4.87E-02
RING1	5.59E-03	0.231439181	0.085474571	0.626667015	3.98E-03
RIMS2	4.86E-04	1.495961521	1.126360865	1.98684182	5.41E-03
RIMBP3	5.15E-03	25225.61687	71.29074671	8925867.322	7.12E-04
RILP	6.28E-03	2.562124329	1.263867523	5.193962941	9.07E-03
RIBC1	2.40E-05	0.013676717	0.001162836	0.160858956	6.43E-04
RHOXF2	2.79E-04	5.30E+56	3.62676E+18	7.75E+94	3.58E-03
RHOQ	3.64E-02	1.868266971	1.134576999	3.076407752	1.40E-02
RHOH	3.05E-02	5.250166954	1.831404781	15.0508797	2.03E-03
RHOF	1.36E-05	809.9837088	36.08350798	18182.09052	2.45E-05
RHOD	1.80E-02	0.439156348	0.249481014	0.773037975	4.34E-03
RHEB	1.91E-02	3.636745128	1.422491509	9.297711126	7.02E-03
RHBDL2	5.28E-04	9.556557886	1.698138147	53.78113599	1.04E-02
RHBDF1	1.82E-02	2.387635493	1.076123939	5.297534086	3.23E-02

RHBDD3	1.47E-02	3.936100434	1.320195367	11.73529844	1.40E-02
RHBDD2	2.56E-04	3.12798616	1.572094868	6.223732181	1.16E-03
RHBDD1	2.93E-04	5.515172719	1.682564021	18.07784413	4.82E-03
RGS5	4.29E-02	1.785800093	1.108257227	2.877564787	1.72E-02
RGS19	1.39E-04	6.780972547	2.678801146	17.16498769	5.36E-05
RGS11	1.40E-03	2.492658718	1.426390541	4.355993191	1.34E-03
RGP1	1.57E-02	0.171667067	0.051442831	0.572860813	4.16E-03
RGMB	7.57E-04	2.180320065	1.324593744	3.588870633	2.17E-03
RGL2	7.77E-03	0.302011453	0.132845613	0.686593372	4.27E-03
RGAG1	6.65E-06	6.98E-09	4.97E-15	0.009801281	9.31E-03
RFX5	3.62E-02	3.150808862	1.244220009	7.9789719	1.55E-02
RFX1	3.67E-02	0.183605773	0.041573325	0.810882454	2.53E-02
RFTN2	3.97E-02	0.349324415	0.174542517	0.69912792	2.97E-03
RFTN1	2.39E-03	2.157050191	1.304204897	3.567587835	2.75E-03
RFPL2	5.14E-04	5.082009564	1.850809708	13.95433637	1.61E-03
RFC5P1	2.76E-06	2.09E+51	4.49235E+16	9.73E+85	3.71E-03
RFC2	9.34E-05	18.39215234	4.301165172	78.64642582	8.57E-05
REXO4	1.44E-04	0.081565521	0.024139515	0.275603478	5.47E-05
REXO1	1.33E-03	0.092792854	0.013921864	0.618488571	1.40E-02
RERG	7.96E-03	75.15224405	1.454998446	3881.694721	3.18E-02
RER1	1.25E-02	3.88809085	1.317704882	11.47240984	1.39E-02
REPS1	2.16E-04	0.113670436	0.037088882	0.348378471	1.42E-04
RENBP	3.58E-04	3.145419451	1.67393266	5.910431022	3.70E-04
REM1	1.52E-02	2.792593901	1.050224803	7.4256299	3.96E-02
RELT	1.03E-04	6.342960722	2.469615628	16.29126017	1.24E-04
RELL2	1.28E-03	2.162557408	1.059179771	4.415354854	3.42E-02
RELB	1.04E-02	2.939336762	1.439023543	6.003863271	3.09E-03
REG3G	7.52E-03	52.2225526	4.413536958	617.9159767	1.70E-03
REEP5	7.61E-04	5.065531632	2.05712621	12.47352281	4.17E-04
REEP4	2.12E-02	2.956910645	1.30065145	6.722262571	9.67E-03
REEP1	6.45E-06	2.221931509	1.361986939	3.624836251	1.39E-03
RECQL5	7.06E-03	0.191204263	0.049773704	0.734505721	1.60E-02
RECQL	1.44E-02	1.83107389	1.118574978	2.997413366	1.61E-02

RDX	1.30E-02	1.619557771	1.059885349	2.474765197	2.58E-02
RDH14	1.33E-02	3.564142916	1.496953809	8.485976419	4.09E-03
RDH13	3.79E-02	19.79970966	1.652996144	237.162382	1.84E-02
RCSD1	8.12E-04	2.742286941	1.384567376	5.431398862	3.81E-03
RCOR2	2.04E-03	2.24754393	1.361983053	3.70889616	1.53E-03
RCAN3	4.12E-04	0.373914028	0.179561351	0.778629142	8.57E-03
RBX1	6.80E-04	11.23728761	3.303158396	38.22905769	1.08E-04
RBPMS2	2.19E-04	3.469584892	1.788596891	6.730426171	2.33E-04
RBPJL	9.64E-03	8.79E-17	3.37E-31	0.022941245	2.90E-02
RBPJ	4.40E-02	3.520309439	1.558745438	7.950354332	2.46E-03
RBP7	5.99E-05	0.446182477	0.301614821	0.660043169	5.36E-05
RBMS2P1	4.46E-03	0.002754409	4.20E-05	0.180739147	5.76E-03
RBMS2	1.10E-02	0.093592849	0.023424518	0.37395098	8.03E-04
RBMS1	7.62E-03	4.890431073	2.032613164	11.76629007	3.95E-04
RBM44	3.02E-02	13773.66123	6.184692033	30674727.64	1.54E-02
RBM15B	1.10E-07	0.13206141	0.046630196	0.374011208	1.38E-04
RBKS	2.08E-04	0.086432332	0.022544598	0.331367546	3.56E-04
RBAKDN	3.46E-03	3.470981127	1.67698943	7.184129945	8.00E-04
RASSF7	2.30E-02	0.229549338	0.065333206	0.806525537	2.17E-02
RASSF5	5.16E-04	2.60122862	1.408518681	4.803905286	2.26E-03
RASSF1-AS1	1.49E-03	0.082525802	0.009729442	0.699989556	2.22E-02
RASL11B	3.07E-02	2.861497903	1.527827649	5.359354669	1.02E-03
RASL11A	3.77E-02	6.885481097	1.768744479	26.8042391	5.40E-03
RASGRP2	4.47E-03	11.52326262	3.042523529	43.64323895	3.21E-04
RASGEF1A	3.38E-02	3.420709422	1.191218809	9.822924945	2.23E-02
RASD2	3.49E-04	2.771479028	1.541303821	4.983505455	6.61E-04
RASAL3	1.91E-05	2.30645688	1.43664383	3.702896451	5.40E-04
RASA4CP	2.00E-02	0.160146202	0.035535685	0.721719759	1.71E-02
RASA3	2.66E-03	2.539794991	1.402589458	4.599035421	2.09E-03
RARS	5.00E-03	2.246102367	1.001694603	5.036441074	4.95E-02
RARRES2P5	3.75E-03	7.65164E+16	4304.865101	1.36E+30	1.25E-02
RARRES2P1	1.06E-04	0.014951013	0.000918748	0.243301468	3.15E-03
RARRES2	1.88E-03	1.757297068	1.209944219	2.552260622	3.07E-03

RARB	5.17E-03	0.405205586	0.224632402	0.730934478	2.69E-03
RAPSN	5.47E-03	0.005915778	4.66E-05	0.751594509	3.79E-02
RAPGEFL1	4.41E-04	6.619045444	1.951947935	22.44514918	2.42E-03
RAPGEF3	2.69E-05	0.168252871	0.05868297	0.4824062	9.12E-04
RAP2C-AS1	6.99E-04	0.04015023	0.005336891	0.302056194	1.79E-03
RAP1GDS1	3.64E-02	2.109603884	1.221014625	3.644860968	7.46E-03
RAP1GAP	1.04E-03	1.817733778	1.24618829	2.651409994	1.92E-03
RAMP2-AS1	1.21E-04	0.01413116	0.001008655	0.19797613	1.56E-03
RAMP1	2.28E-02	1.466859551	1.072932376	2.005417109	1.63E-02
RALB	1.41E-02	4.971483472	1.272786844	19.41852873	2.11E-02
RAI2	1.49E-02	1.770564568	1.071164494	2.926626963	2.59E-02
RAI14	1.51E-02	2.14187268	1.127824076	4.067672146	1.99E-02
RAF1	1.41E-04	0.131218757	0.046365268	0.371363373	1.30E-04
RAET1K	4.31E-04	6050.761267	4.139930307	8843557.547	1.92E-02
RADIL	1.39E-02	0.47985203	0.287227899	0.801656008	5.04E-03
RAD54L2	1.12E-04	0.035845171	0.006173459	0.20812909	2.08E-04
RAD51AP2	1.88E-02	2.47E+33	5983513.478	1.02E+60	1.39E-02
RAC2	1.41E-05	2.031751062	1.456992415	2.833242189	2.94E-05
RAC1P2	4.14E-02	6.681414237	1.594081076	28.0044076	9.38E-03
RABL2A	2.59E-04	0.030546986	0.005277254	0.176818927	9.86E-05
RABIF	8.90E-03	4.274394811	1.626970912	11.22973427	3.20E-03
RABEP2	5.49E-03	5.329941993	1.296320773	21.91454633	2.04E-02
RABAC1	1.09E-02	3.233565008	1.413070333	7.399449565	5.46E-03
RAB8A	4.27E-02	10.15328883	1.705782234	60.4351904	1.09E-02
RAB7B	4.14E-04	5.025984954	2.502555931	10.09389019	5.67E-06
RAB7A	9.67E-04	0.285033354	0.115573492	0.702964075	6.43E-03
RAB6C	4.68E-02	1175.43904	1.432992427	964176.0217	3.89E-02
RAB6B	4.39E-02	0.576302104	0.345889511	0.960202912	3.44E-02
RAB5B	1.34E-05	0.120377311	0.041796442	0.346696903	8.76E-05
RAB5A	3.05E-02	0.389258615	0.179808686	0.842686037	1.67E-02
RAB4B	6.74E-03	4.493632655	1.872033557	10.78652376	7.70E-04
RAB43P1	2.42E-02	0.042274905	0.006381176	0.280068677	1.04E-03
RAB43	3.54E-03	0.00178993	3.52E-05	0.09097314	1.60E-03

RAB42	2.96E-04	3.841204949	1.803972122	8.179092835	4.83E-04
RAB34	5.38E-03	0.468668186	0.30726815	0.7148475	4.34E-04
RAB31	3.64E-05	2.718533487	1.730868704	4.269777544	1.41E-05
RAB2B	4.82E-02	3.048089994	1.303827769	7.125828141	1.01E-02
RAB2A	3.74E-04	2.300124416	1.521882701	3.476333837	7.72E-05
RAB1A	2.54E-02	2.102432242	1.109536055	3.983846501	2.27E-02
RAB19	4.50E-03	312590.751	5.078319128	19241204646	2.45E-02
RAB15	3.87E-05	3.197949618	1.70381928	6.002327759	2.96E-04
RAB11FIP3	3.16E-03	5.481675933	1.778011723	16.90020974	3.06E-03
RAB11FIP1	1.45E-03	0.313938573	0.163469387	0.602910608	5.02E-04
RAB11AP1	7.23E-05	7.73E+27	55684517.24	1.07E+48	6.65E-03
RAB11A	1.72E-02	2.876301703	1.164376703	7.105184654	2.20E-02
QRICH1	2.16E-05	0.131205276	0.04562735	0.377291788	1.64E-04
QRFPR	3.64E-02	0.00022889	1.42E-07	0.367660075	2.60E-02
QPCT	2.77E-03	1.440193462	1.118987838	1.853601209	4.61E-03
QDPR	3.34E-04	1.892246332	1.155594965	3.098487176	1.13E-02
QARS	3.22E-04	0.217927649	0.091641293	0.518243014	5.67E-04
PYGO1	2.18E-02	2.153654247	1.001213806	4.632603537	4.96E-02
PYCRL	9.61E-03	2.527927628	1.373731786	4.651867386	2.88E-03
PYCR2	1.91E-02	4.246010082	1.247710442	14.4493474	2.07E-02
PXT1	3.82E-02	1.46322E+11	8376.263968	2.55605E+18	2.51E-03
PXDN	1.47E-02	2.672298128	1.680074337	4.250512686	3.31E-05
PXDC1	8.12E-05	0.358443049	0.193616497	0.663587148	1.09E-03
PWWP2B	5.53E-03	3.68159284	1.170645529	11.57833477	2.58E-02
PVT1	4.48E-05	4.223491335	2.183506628	8.169372526	1.87E-05
PVRL1	1.79E-04	3.257195274	1.691323198	6.272793437	4.13E-04
PUS1	3.55E-03	5.835767665	1.269876905	26.81849249	2.34E-02
PTTG2	5.42E-03	804859024.2	2075.924679	3.12053E+14	1.79E-03
PTRH2	1.20E-04	6.609009772	2.564546261	17.03186674	9.24E-05
PTPRH	9.58E-03	3.152153576	1.575310316	6.307374535	1.18E-03
PTPRE	3.95E-03	6.474010101	1.984080805	21.12454628	1.97E-03
PTPRC	3.00E-03	1.833133171	1.213517716	2.769120859	3.98E-03
PTPRA	2.36E-03	4.405069791	1.814030386	10.6969762	1.05E-03

PTPN9	3.99E-03	3.354960045	1.133284347	9.931979505	2.88E-02
PTPN7	3.15E-04	2.236360595	1.372467229	3.64402778	1.23E-03
PTPN6	3.86E-04	1.832422287	1.247788159	2.690978764	2.01E-03
PTPN23	4.49E-03	0.232538434	0.10351553	0.522376916	4.12E-04
PTPN22	2.14E-02	5.275495036	1.741072851	15.98488418	3.28E-03
PTPN21	4.61E-02	0.324158848	0.141028539	0.745090035	7.98E-03
PTPN18	1.90E-02	7.078848949	2.392922899	20.94095989	4.05E-04
PTPN13	1.34E-02	1.571158594	1.034274942	2.386734153	3.42E-02
PTPN1	2.68E-04	4.67585364	2.0406646	10.7139641	2.66E-04
PTPLB	4.18E-03	0.450299682	0.255449798	0.793775547	5.81E-03
PTPLAD2	4.99E-04	4.140909953	1.607812365	10.66488578	3.24E-03
PTPLA	3.19E-05	4.273693684	2.044098349	8.935214747	1.13E-04
PTP4A3	3.14E-05	1.901607003	1.412433256	2.560198282	2.28E-05
PTP4A2P2	4.68E-03	110.528764	2.240967773	5451.487447	1.80E-02
PTMS	3.53E-02	2.03359822	1.129912841	3.660036039	1.79E-02
PTK2	2.32E-02	2.177976075	1.233330879	3.846153422	7.30E-03
PTHLH	1.17E-02	3.36652095	1.420739689	7.977156824	5.82E-03
PTGS2	1.49E-04	657.3488805	32.69619749	13215.83499	2.26E-05
PTGIR	4.95E-02	83.25724575	4.168491686	1662.896196	3.80E-03
PTGER4	3.88E-02	1.3956731	1.131105857	1.72212299	1.88E-03
PTGER1	1.27E-02	3.240297122	1.739928231	6.034458922	2.11E-04
PTDSS1	2.72E-03	3.546277025	1.777829225	7.073840705	3.27E-04
PTCD2P1	5.28E-04	1.34E+50	2.10251E+11	8.51E+88	1.13E-02
PSTPIP2	3.74E-02	3.935588443	1.681164887	9.213169102	1.59E-03
PSTPIP1	6.52E-04	3.256870566	1.768618355	5.997453242	1.50E-04
PSPH	6.29E-03	4.001928819	1.51551959	10.56761943	5.12E-03
PSPC1P1	8.45E-03	2.668405725	1.21947434	5.838900317	1.40E-02
PSMG3	9.69E-05	6.135084914	2.832158164	13.28995936	4.23E-06
PSMG1	4.37E-04	3.655845901	1.409942675	9.479257199	7.66E-03
PSME4	2.86E-02	2.705278446	1.4669961	4.988787269	1.44E-03
PSME3	4.79E-04	7.630880971	2.152569756	27.05154815	1.65E-03
PSME2P2	6.26E-04	3.418272893	1.657141036	7.051053177	8.77E-04
PSME2	2.15E-04	2.235654703	1.409266138	3.546634534	6.33E-04

PSME1	2.59E-02	2.222316549	1.140087721	4.331851622	1.90E-02
PSMD8	2.02E-03	8.589071893	2.052668955	35.9396267	3.23E-03
PSMD2	1.05E-02	0.229072956	0.086765024	0.604787698	2.93E-03
PSMD11	1.23E-03	4.944691969	1.681177918	14.54336178	3.69E-03
PSMC5	3.44E-03	8.491909243	2.845645716	25.34135651	1.26E-04
PSMC4	3.11E-04	8.418456151	2.954058543	23.99085967	6.69E-05
PSMC3	4.63E-03	6.224712459	2.072055754	18.69980821	1.12E-03
PSMC2	3.66E-02	3.834472653	1.609648807	9.134402771	2.41E-03
PSMB9	7.91E-03	1.456169362	1.178738559	1.798896959	4.92E-04
PSMB8-AS1	2.71E-04	1.83538925	1.338635956	2.516482308	1.62E-04
PSMB8	8.44E-04	1.714953307	1.24653111	2.359399475	9.20E-04
PSMB6	6.56E-03	5.273573884	1.850522844	15.02849943	1.86E-03
PSMB5	4.31E-03	8.018780784	2.31453686	27.78130104	1.02E-03
PSMB4	4.87E-04	28.44810536	5.690740572	142.2125448	4.55E-05
PSMB3	6.89E-06	7.301730792	2.867805501	18.5909653	3.05E-05
PSMB10	7.49E-03	2.127812137	1.38652106	3.265427854	5.49E-04
PSMA7	2.87E-02	3.003714856	1.533218308	5.884552052	1.35E-03
PSMA6	1.61E-02	3.228305287	1.335862901	7.801665141	9.24E-03
PSMA4	1.92E-02	2.225917504	1.234883077	4.01228977	7.77E-03
PSMA3	4.99E-02	2.513181371	1.42954815	4.418235652	1.37E-03
PSEN1	1.35E-02	5.920589425	1.865379907	18.79154965	2.54E-03
PSD2	4.16E-05	1.828270584	1.050967156	3.180473632	3.27E-02
PSAP	5.91E-03	2.709163181	1.213933478	6.046101598	1.50E-02
PRUNE	3.17E-03	12.97618041	2.538426225	66.33293355	2.08E-03
PRSS58	1.88E-02	1.43E+44	1.780247319	1.15E+88	4.87E-02
PRSS42	3.59E-02	6.49E-13	2.64E-23	0.015959857	2.15E-02
PRRX2	2.05E-02	1.91104067	1.195534484	3.054764617	6.81E-03
PRRX1	1.72E-02	4.777383036	2.343220571	9.740179371	1.69E-05
PRRT4	1.37E-02	0.524849941	0.32809313	0.83960143	7.16E-03
PRRT3-AS1	7.17E-04	0.333018152	0.180172102	0.615528642	4.51E-04
PRRT3	2.00E-03	0.071744194	0.01393119	0.369475215	1.63E-03
PRRT1	1.48E-05	0.132454864	0.046650901	0.376076144	1.47E-04
PRRC2A	1.18E-03	0.327211485	0.158864083	0.673955711	2.44E-03

PRR5L	3.49E-05	5.375498893	2.311846274	12.49909593	9.36E-05
PRR36	8.49E-03	0.15198113	0.027153364	0.850659392	3.20E-02
PRR34-AS1	4.97E-03	2.87403146	1.453843788	5.681529821	2.40E-03
PRR34	2.05E-02	9.657201483	1.233985663	75.57749113	3.08E-02
PRR30	7.54E-03	1.93E+51	5.67074E+12	6.60E+89	9.09E-03
PRR29	8.70E-03	19.90190586	1.693856489	233.8367267	1.74E-02
PRPF31	4.28E-04	22.58934118	3.173068947	160.8153947	1.85E-03
PROSP	4.06E-02	7.16876E+13	64830.81833	7.93E+22	2.68E-03
PROSER2	6.83E-03	4.198881956	1.688418599	10.4420845	2.02E-03
PROS1	5.28E-04	1.532808386	1.191113095	1.972526001	9.03E-04
PROB1	1.55E-02	2.692630558	1.165445097	6.22102177	2.04E-02
PRLR	3.21E-04	342.0687473	17.11639374	6836.196322	1.34E-04
PRL	1.52E-03	0.007018493	5.07E-05	0.97233176	4.87E-02
PRKRIP1	2.27E-03	5.064694842	1.025265679	25.01901154	4.65E-02
PRKDC	3.75E-03	2.094288376	1.390730275	3.153770275	4.02E-04
PRKCDBP	9.10E-03	2.18233677	1.423247679	3.346285997	3.46E-04
PRKCD	1.27E-06	0.265343472	0.139333031	0.505315631	5.42E-05
PRKCA	4.03E-04	1.848712534	1.217006582	2.80831516	3.97E-03
PRKAR2B	1.91E-02	1.953453129	1.049946871	3.634449735	3.45E-02
PRKAR2A-AS1	1.49E-03	0.029265933	0.002694045	0.317921473	3.71E-03
PRKAR1B	5.14E-04	3.250248872	1.551871361	6.80734112	1.78E-03
PRIMA1	4.75E-02	1.68123188	1.113711857	2.537946071	1.34E-02
PRICKLE2	3.05E-04	0.238132157	0.095793547	0.591970187	2.01E-03
PRH1-PRR4	1.79E-02	1879.567334	2.79340877	1264681.847	2.33E-02
PRF1	4.33E-06	1.591678247	1.214531336	2.085940122	7.56E-04
PREX1	1.42E-04	2.288621543	1.337985452	3.91468275	2.50E-03
PRELID2P1	8.95E-03	26091.17293	9.0324954	75366692.7	1.24E-02
PRELID1P1	6.69E-03	56.95125181	6.548481957	495.2972466	2.49E-04
PRELID1	9.29E-03	11.09087623	2.83020136	43.46246786	5.55E-04
PREB	9.96E-04	8.120183198	2.657770465	24.80928133	2.38E-04
PRDX5	3.90E-02	3.413167136	1.459622618	7.981316368	4.62E-03
PRDX4	2.24E-04	3.632287712	1.687989794	7.81611007	9.70E-04
PRDX2P2	4.46E-11	2.48E+69	3.03E+37	2.03E+101	2.03E-05

PRDM6	3.05E-04	1409392.884	94.73231031	20968435112	3.87E-03
PRDM12	1.89E-02	860941.3905	251.4556472	2947716968	9.98E-04
PRDM11	5.14E-03	0.095353326	0.022879131	0.39740394	1.25E-03
PRDM1	4.48E-03	5.089788971	1.975920912	13.11082423	7.50E-04
PRCP	4.04E-04	0.256736138	0.110734173	0.595240317	1.53E-03
PRAF2	1.61E-02	2.42525939	1.361724023	4.319438455	2.63E-03
PQLC3	8.57E-03	4.295800425	1.66190744	11.10404878	2.63E-03
PPP6R3	4.66E-02	1.872562292	1.049354102	3.341569382	3.38E-02
PPP6R2	2.83E-03	6.460942756	1.633595346	25.55331796	7.82E-03
PPP5C	1.25E-03	6.876309447	1.969598693	24.00673385	2.51E-03
PPP2R5C	1.17E-02	4.127822998	1.321034091	12.89817032	1.47E-02
PPP2R3C	4.33E-02	2.032110864	1.024551771	4.030518204	4.24E-02
PPP2R3B	2.89E-05	0.041004011	0.00959291	0.17526787	1.64E-05
PPP1R9B	6.87E-03	2.974916448	1.337160041	6.618600315	7.54E-03
PPP1R7	3.52E-04	9.393717525	2.69335209	32.76286426	4.41E-04
PPP1R42	1.78E-02	1.07E-05	1.71E-10	0.668921656	4.22E-02
PPP1R3G	4.80E-02	28.79911037	1.001749087	827.9406177	4.99E-02
PPP1R3C	1.25E-06	0.321366084	0.200820352	0.514271382	2.22E-06
PPP1R3B	1.05E-04	0.274259664	0.14429176	0.521293547	7.88E-05
PPP1R35	4.06E-03	15.92924989	4.333022004	58.55982313	3.08E-05
PPP1R26	2.23E-03	0.266155978	0.103588507	0.683850037	5.97E-03
PPP1R14C	6.91E-05	4.647228067	1.823490179	11.84362217	1.29E-03
PPP1R14BP3	4.21E-03	2.102705065	1.050131515	4.210299878	3.59E-02
PPP1R13L	6.13E-05	0.045941508	0.009362062	0.225444158	1.47E-04
PPME1	1.39E-03	4.875416612	1.51510864	15.68843745	7.89E-03
PPM1M	2.95E-03	0.400510902	0.208034252	0.771070058	6.18E-03
PPM1K	9.01E-05	2.497552443	1.625994588	3.836278576	2.92E-05
PPIF	5.07E-05	6.416161341	2.201192892	18.70218939	6.61E-04
PPIC	1.65E-03	4.130512771	2.019545438	8.448007869	1.02E-04
PPIB	1.49E-03	4.166246027	2.099184982	8.268735776	4.50E-05
PPIAP9	7.14E-03	16.04232094	3.228216585	79.7208162	6.92E-04
PPIAP6	3.13E-03	45.09104674	4.932625957	412.1947445	7.42E-04
PPIAP31	3.36E-03	12.79115663	2.670732594	61.26172586	1.43E-03

PPIAP25	8.78E-03	4158127.398	136.2993278	1.26853E+11	3.82E-03
PPIAP22	1.70E-03	10.26668755	2.394270756	44.0237901	1.72E-03
PPIAP21	4.87E-02	7.915447455	1.06917464	58.60063086	4.28E-02
PPIAP19	7.74E-04	571.0910711	4.995666392	65285.58672	8.66E-03
PPIAP13	8.08E-03	69.06013341	5.754820973	828.7489826	8.37E-04
PPIAP11	2.19E-02	3.031235935	1.317076966	6.976351063	9.12E-03
PPIA	3.09E-04	14.06755122	3.579028291	55.29321961	1.53E-04
PPEF1	8.64E-03	8.691390461	1.495000213	50.52860027	1.61E-02
PPCDC	1.13E-04	20.7581795	4.64002867	92.86624	7.26E-05
PPARG	2.91E-03	0.52151403	0.332253343	0.818582833	4.65E-03
PPARD	6.14E-03	0.202480918	0.061066053	0.671379923	9.02E-03
PPAPDC1B	6.37E-04	3.97011871	1.964513391	8.023280801	1.22E-04
PPAP2C	9.28E-03	1.795275475	1.033316974	3.119095218	3.79E-02
PPAP2B	7.31E-03	2.035479012	1.199792214	3.453243619	8.40E-03
PPAN	2.19E-02	6.118578767	1.166617405	32.09021738	3.22E-02
PPA2	2.07E-02	2.83088193	1.324869301	6.048817418	7.23E-03
PP12613	2.61E-02	927117.591	6.405349714	1.34192E+11	2.34E-02
POU2AF1	7.03E-04	4.040557912	1.525263708	10.70379381	4.96E-03
POTEM	2.24E-02	4.23E+27	1441.809897	1.24E+52	2.69E-02
POR	9.45E-05	4.309650188	2.129703293	8.720972922	4.86E-05
POPDC2	3.59E-02	0.058290627	0.007236214	0.469554517	7.58E-03
POP7	1.76E-04	32.45402375	7.467140578	141.0531443	3.45E-06
POP4	2.26E-03	9.442849216	2.142756221	41.6134138	3.01E-03
PON3	2.25E-02	0.030361905	0.001225126	0.752449205	3.29E-02
POMT1	2.89E-03	5.52801573	1.544021608	19.79179421	8.60E-03
POMP	1.37E-06	8.417336598	3.357778745	21.10072187	5.54E-06
POMGNT2	9.85E-07	0.112116484	0.043802278	0.286973799	5.04E-06
POMC	2.27E-03	2.006530951	1.38489171	2.9072067	2.32E-04
POLR3K	2.89E-03	34.30137165	5.780688221	203.5370274	9.98E-05
POLR3GP1	1.52E-03	83302.96297	12.19752852	568917189.3	1.19E-02
POLR3C	9.36E-04	6.922281146	2.770264902	17.29725422	3.46E-05
POLR2K	2.32E-02	2.520409056	1.466295892	4.332319175	8.23E-04
POLR2J4	1.44E-04	9.938424601	3.270139508	30.20430269	5.14E-05

POLR2J	3.87E-04	14.30129049	4.055384346	50.43342193	3.51E-05
POLR2H	3.54E-03	0.114985766	0.024939485	0.530152344	5.54E-03
POLR2E	4.71E-02	4.393902016	1.450345844	13.31156634	8.86E-03
POLL	5.09E-03	0.120051804	0.038475135	0.3745909	2.61E-04
POLE	4.35E-03	4.213756106	1.251160441	14.19141777	2.03E-02
POLD4	1.19E-03	9.766309094	1.793438535	53.18319611	8.40E-03
POLD1	5.50E-03	0.106732566	0.024715248	0.460923583	2.72E-03
POLA1	1.40E-02	1.963265052	1.380880337	2.791269859	1.72E-04
POFUT1	5.29E-03	4.716779866	1.485528587	14.97649557	8.50E-03
PNPLA2	2.96E-02	0.239824415	0.081772007	0.703367225	9.30E-03
PNP	1.47E-02	3.153063835	1.623311822	6.124400384	6.98E-04
PNOC	2.64E-03	24.29555388	3.659184602	161.3129707	9.56E-04
PNKP	4.12E-05	4.767874476	2.209632417	10.28796774	6.88E-05
PMVK	6.09E-03	11.31149058	3.448111395	37.10721741	6.28E-05
PMP22	3.08E-02	0.594208442	0.374871444	0.941879352	2.68E-02
PMM2	9.32E-06	7.720088511	2.445495511	24.37124352	4.93E-04
PMM1	1.18E-02	0.190179757	0.057631292	0.627581622	6.43E-03
PML	2.15E-02	0.282178977	0.129159379	0.6164862	1.51E-03
PMEPA1	1.46E-03	1.783669986	1.089088843	2.921229651	2.15E-02
PLXND1	2.63E-03	0.462359173	0.258919038	0.825648073	9.12E-03
PLXNC1	1.08E-02	1.755742271	1.132159023	2.722789699	1.19E-02
PLXNB1	1.22E-06	0.256626349	0.136786173	0.481460087	2.27E-05
PLXNA3	1.93E-02	5.920112923	1.840856266	19.03882322	2.85E-03
PLSCR4	3.86E-03	0.187579382	0.068664881	0.512431159	1.10E-03
PLOD3	1.43E-02	3.05238698	1.459543798	6.383546893	3.03E-03
PLN	1.94E-04	1.434838879	1.157312922	1.778916116	9.94E-04
PLIN5	3.75E-03	0.159042954	0.03063082	0.825791194	2.87E-02
PLIN3	4.56E-03	3.308354642	1.000442968	10.9403642	4.99E-02
PLIN2	9.52E-06	0.372587934	0.230460041	0.602368062	5.63E-05
PLEKHO1	3.95E-02	2.395848864	1.455483872	3.943768728	5.90E-04
PLEKHH3	3.07E-04	8.651943624	2.467432261	30.33766303	7.49E-04
PLEKHG6	7.32E-05	89.47132719	3.984400864	2009.114711	4.64E-03
PLEKHG4B	8.78E-06	3.202669964	2.026722152	5.060928006	6.17E-07

PLEKHG4	8.93E-07	0.141785966	0.055223032	0.3640376	4.90E-05
PLEKHG3	7.14E-03	0.234680515	0.086850808	0.63413278	4.26E-03
PLEKHG2	8.36E-04	7.078809964	2.91463255	17.1924075	1.54E-05
PLEKHA8P1	1.14E-02	3.199583387	1.225866041	8.351103228	1.75E-02
PLEK2	3.35E-03	4.129261254	1.841052432	9.261441013	5.80E-04
PLEK	7.08E-03	1.794126003	1.172146542	2.746148198	7.12E-03
PLCL2	1.65E-03	3.386150341	1.227953046	9.337502085	1.84E-02
PLCH1-AS1	7.88E-04	0.054434542	0.005710692	0.518872176	1.14E-02
PLCG1	2.96E-02	2.354629548	1.123913896	4.93301162	2.32E-02
PLCE1-AS1	1.49E-02	0.485810706	0.238695548	0.98875762	4.65E-02
PLCD1	4.95E-06	0.146407396	0.06324262	0.338934812	7.25E-06
PLCB3	2.28E-03	5.538095907	1.503355079	20.40137204	1.01E-02
PLBD1-AS1	3.72E-02	11.54906452	3.248312515	41.0615945	1.57E-04
PLAUR	3.13E-02	4.863674775	1.619839609	14.60350283	4.81E-03
PLAU	1.42E-02	3.101230624	1.496903196	6.425018936	2.32E-03
PLAC8	1.00E-02	9.545706219	1.082110855	84.20625929	4.23E-02
PLA2R1	1.03E-03	4.55048836	1.458752091	14.19497147	9.04E-03
PLA2G7	5.59E-03	1.843455156	1.250091356	2.718462851	2.03E-03
PLA2G4C	4.33E-04	1.923598996	1.192429905	3.103103234	7.33E-03
PLA2G4A	4.95E-02	1.982284346	1.036368562	3.791557726	3.86E-02
PLA2G2C	5.82E-04	1.90E-25	3.23E-44	1.12E-06	9.84E-03
PLA2G1B	5.58E-03	0.057186605	0.008490117	0.385189964	3.28E-03
PLA2G16	4.99E-03	2.289430327	1.312194275	3.994447562	3.54E-03
PLA1A	3.94E-02	0.728157886	0.549828076	0.964326724	2.69E-02
PKMP4	3.21E-02	69.39624302	4.34353337	1108.73755	2.71E-03
PKM	1.03E-03	1.909491303	1.113993376	3.273050914	1.86E-02
PKDREJ	4.03E-02	1.84E-07	2.75E-13	0.123569721	2.35E-02
PKD1	9.39E-03	0.071182707	0.014790292	0.342588071	9.80E-04
PITPNC1	3.89E-04	3.971985074	1.952109893	8.08185312	1.41E-04
PISD	1.31E-03	10.028678	2.584604949	38.91286459	8.60E-04
PIP5KL1	2.37E-04	7.84E-05	2.69E-07	0.022823678	1.09E-03
PIM2	2.71E-03	2.653703619	1.55715186	4.522450943	3.33E-04
PIK3R5	1.38E-02	2.402238841	1.056638779	5.46142311	3.65E-02

PIK3R4	2.69E-02	0.298733829	0.137820287	0.647523688	2.21E-03
PIK3R3	8.69E-04	1.731039196	1.028972932	2.912123929	3.87E-02
PIK3CG	1.85E-02	10.69266541	1.810267191	63.15813159	8.93E-03
PIK3C2B	1.18E-03	4.66297353	1.481951318	14.67208934	8.47E-03
PIGZ	1.94E-04	0.0867231	0.024039032	0.312861854	1.88E-04
PIGW	1.21E-02	2.747292387	1.056307891	7.145279826	3.82E-02
PIGU	6.90E-04	4.268597075	1.82286796	9.995743731	8.29E-04
PIGT	3.48E-04	2.92328486	1.546142578	5.527041616	9.64E-04
PIGH	1.57E-03	3.789730402	1.607930953	8.932010729	2.32E-03
PIGB	1.57E-02	3.191570398	1.314381128	7.749747306	1.04E-02
PIFO	6.13E-04	0.068698305	0.00879474	0.536622703	1.07E-02
PICK1	9.32E-04	3.936237844	1.408000121	11.00423795	8.99E-03
PI15	6.44E-03	2239.026797	67.2476144	74548.97908	1.61E-05
PHYKPL	4.66E-02	2.565900304	1.116463687	5.897051956	2.65E-02
PHYHIP	4.33E-03	1.884750769	1.159835677	3.062748916	1.05E-02
PHYHD1	2.20E-05	0.375942345	0.228995566	0.617185079	1.10E-04
PHTF1	2.89E-03	2.344682465	1.288844936	4.265475009	5.25E-03
PHPT1	2.44E-02	6.560716101	2.084473314	20.64933884	1.30E-03
PHLDA2	4.08E-04	2.414400047	1.546224993	3.770038392	1.06E-04
PHLDA1	2.33E-03	0.397002032	0.249142245	0.632612963	1.02E-04
PHKG1P4	5.28E-04	4.07841E+16	5649.84967	2.94E+29	1.13E-02
PHKA2-AS1	8.74E-03	0.010594841	0.000402619	0.278801102	6.42E-03
PHKA2	7.48E-05	0.126793007	0.041811787	0.384496045	2.64E-04
PHKA1	3.24E-03	0.531626487	0.296939777	0.951798121	3.35E-02
PHF1	2.35E-04	0.099240709	0.030057553	0.327662018	1.50E-04
PHC2	1.78E-02	0.385597954	0.199930431	0.743687599	4.46E-03
PHB2	4.51E-02	0.283668948	0.086390584	0.931444935	3.78E-02
PHB	4.31E-02	4.734377883	1.316090683	17.03099507	1.73E-02
PHACTR3	1.64E-04	2.134067976	1.233987836	3.690673434	6.68E-03
PGR	2.45E-02	23502843.82	1967.762398	2.80717E+11	3.95E-04
PGP	4.06E-03	4.803670822	1.702102433	13.55691228	3.03E-03
PGM1	2.92E-02	1.83276625	1.170447399	2.869870216	8.10E-03
PGF	5.81E-03	2.450601166	1.434882437	4.185322728	1.03E-03

PGBD1	3.44E-02	0.292770295	0.114516042	0.748492908	1.03E-02
PGAP1	1.50E-02	2.049366314	1.082033969	3.881488391	2.77E-02
PGAM5	4.37E-02	6.687457082	1.704659587	26.23519826	6.43E-03
PGAM1P9	7.46E-05	6.73E+32	2.26255E+12	2.00E+53	1.67E-03
PGAM1P12	3.36E-02	1.43107E+12	5.328460548	3.84E+23	3.71E-02
PGAM1	4.77E-04	2.545121987	1.273291995	5.087321646	8.20E-03
PFN4	3.72E-02	5.707874622	1.067017349	30.53355482	4.18E-02
PFN1	2.06E-04	8.850723271	2.54117023	30.82646786	6.15E-04
PFKP	3.87E-07	2.200567947	1.458543415	3.320092663	1.71E-04
PFDN2	5.32E-05	9.774399295	3.667161427	26.05254322	5.17E-06
PEX2	2.39E-02	2.456313295	1.318249012	4.57688566	4.65E-03
PET100	3.85E-02	3.668824095	1.054278249	12.7672844	4.10E-02
PES1P2	2.43E-02	2.64227E+13	5.612498703	1.24E+26	3.79E-02
PES1P1	1.46E-03	4.38E+55	1.21838E+19	1.58E+92	2.85E-03
PERP	4.99E-02	0.649921743	0.441135231	0.95752559	2.93E-02
PER3	1.69E-02	0.43326836	0.205864423	0.911869421	2.76E-02
PEPD	6.66E-03	0.345673154	0.144310523	0.828005658	1.72E-02
PELI3	4.77E-03	0.218976747	0.070607285	0.679119947	8.54E-03
PELI2	8.08E-03	0.336026227	0.144047136	0.783865813	1.16E-02
PEG3	6.82E-05	0.461454175	0.218222457	0.975793047	4.30E-02
PECAM1	2.10E-03	3.080670543	1.661131124	5.713294309	3.56E-04
PEBP1	4.86E-02	0.234529606	0.075635069	0.727230595	1.20E-02
PEAK1	7.22E-03	5.008873307	1.790283124	14.01387941	2.14E-03
PEA15	1.30E-03	2.080771773	1.219817086	3.549393775	7.16E-03
PDZD4	4.82E-02	1.729665621	1.097286992	2.726491047	1.83E-02
PDZD11	1.77E-04	12.76380803	2.802216005	58.1378434	9.95E-04
PDXK	4.57E-06	7.566155439	2.750042392	20.81666388	8.89E-05
PDS5A	2.87E-02	2.128272623	1.147567271	3.94708395	1.65E-02
PDLIM7	3.75E-02	2.389447555	1.056969518	5.401725898	3.63E-02
PDLIM4	2.26E-04	1.933351971	1.192227653	3.135181301	7.52E-03
PDLIM1	1.15E-03	2.345560286	1.373602221	4.005273849	1.79E-03
PDK2	2.24E-03	0.135854997	0.043906431	0.420361655	5.33E-04
PDIA3	2.30E-04	4.293474602	1.876740907	9.822306365	5.59E-04

PDHA1	1.55E-02	3.182283507	1.213602402	8.344519013	1.86E-02
PDGFRB	2.47E-02	1.917563861	1.177188272	3.123588001	8.92E-03
PDF	1.86E-02	2.829055413	1.023202593	7.822062397	4.51E-02
PDE6G	5.54E-05	0.325599722	0.175370387	0.604521556	3.79E-04
PDE4DIP	2.12E-03	2.459989706	1.351336281	4.478196463	3.23E-03
PDE4B	5.84E-05	0.073886005	0.018812285	0.290190263	1.90E-04
PDE4A	3.01E-02	2.570682181	1.000339951	6.606161101	4.99E-02
PDE3A	2.69E-03	0.568013197	0.38813314	0.831258553	3.60E-03
PDCL3	7.11E-03	4.699834047	1.095261615	20.16727306	3.73E-02
PDCD6	1.29E-03	4.743628528	1.754222114	12.82734463	2.16E-03
PDCD5P2	1.05E-02	287508.8786	16.8173519	4915242052	1.15E-02
PDCD4-AS1	3.62E-04	0.29978194	0.163802427	0.548643957	9.36E-05
PDCD4	1.28E-02	0.53098117	0.326387787	0.863822158	1.08E-02
PDCD1	7.43E-05	1.905041787	1.288915389	2.815688479	1.22E-03
PDAP1	1.83E-03	28.95501889	5.266284882	159.2001074	1.09E-04
PCSK4	3.55E-05	0.065838729	0.017907868	0.242057749	4.21E-05
PCSK1	1.04E-03	1.72934076	1.060117413	2.821026643	2.83E-02
PCOLCE2	2.11E-04	0.087675148	0.023039426	0.333642493	3.57E-04
PCOLCE	1.62E-03	1.582222773	1.11335738	2.248540271	1.05E-02
PCNA	8.05E-03	2.65146189	1.404266829	5.00634923	2.64E-03
PCK1	1.40E-05	2821.165653	5.044499864	1577753.168	1.38E-02
PCGF1	1.93E-02	5.062554985	1.286408676	19.92326657	2.03E-02
PCED1B-AS1	3.76E-02	2.516246415	1.290011975	4.908090892	6.79E-03
PCED1B	1.22E-02	2.221361694	1.344754703	3.669403621	1.83E-03
PCED1A	4.91E-02	0.320755502	0.115853275	0.888055107	2.86E-02
PCDHGC3	2.21E-03	1.917549322	1.152118024	3.191509311	1.23E-02
PCDHB9	9.16E-05	0.001250043	1.88E-05	0.083328903	1.81E-03
PCDHB16	1.32E-03	0.204760167	0.068486294	0.612191487	4.54E-03
PCDHB10	1.44E-02	0.040672962	0.002422363	0.682923898	2.61E-02
PCDHAC1	7.84E-04	2.12E-06	8.11E-11	0.05539324	1.18E-02
PCDHA7	2.13E-04	7.84E-15	1.23E-28	0.498090412	4.52E-02
PCDHA3	8.49E-03	3.50E-19	2.77E-37	0.441419934	4.57E-02
PCDHA2	5.14E-03	3.17E-17	2.99E-32	0.033579368	3.14E-02

PCDHA13	3.44E-03	1.23E-05	4.22E-10	0.360394508	3.12E-02
PCDHA12	2.93E-02	2.94E-09	1.06E-16	0.081500392	2.47E-02
PCDHA11	1.51E-03	2.65E-07	1.66E-12	0.042112969	1.32E-02
PCDHA10	1.41E-04	1.55E-13	1.38E-24	0.017302036	2.31E-02
PCDH20	2.21E-04	0.019284651	0.000465304	0.799257679	3.77E-02
PCDH17	1.61E-02	7.909975689	1.541492253	40.58905602	1.32E-02
PCBP4	2.74E-03	0.390176579	0.202965105	0.750068652	4.77E-03
PCBP3	2.46E-02	0.346041332	0.123259686	0.971482303	4.39E-02
PCBD2	1.32E-02	6.369739631	1.869415442	21.70388778	3.07E-03
PCAT6	7.57E-03	0.404406791	0.234385248	0.697760863	1.14E-03
PC	4.58E-02	0.417663148	0.231884737	0.752281098	3.64E-03
PBXIP1	1.91E-03	0.23658941	0.095168729	0.588161147	1.92E-03
PBX3	1.93E-03	1.701637233	1.012496425	2.859831602	4.48E-02
PBX1	1.30E-03	0.356640669	0.178121701	0.71407676	3.61E-03
PBDC1	4.82E-02	2.891627023	1.399026645	5.976660181	4.15E-03
PATL2	3.12E-02	4.265177569	1.680687032	10.82399004	2.27E-03
PATE3	5.16E-03	1.45E+60	13186932.37	1.59E+113	2.62E-02
PASK	1.80E-02	0.074227663	0.009953731	0.553535731	1.12E-02
PARVG	2.70E-05	3.017480876	1.51195434	6.022133473	1.73E-03
PARVA	2.12E-04	0.138113514	0.051889177	0.367616984	7.39E-05
PART1	6.03E-04	2215.1559	1.492152176	3288482.062	3.87E-02
PARP8	5.12E-06	4.74678717	2.482126496	9.077695468	2.50E-06
PARP3	4.68E-02	0.305971068	0.13090276	0.715174335	6.26E-03
PARP2	9.03E-04	3.65538366	1.389678075	9.61505398	8.62E-03
PARP12	2.92E-02	2.168567431	1.303028118	3.609043149	2.90E-03
PARP10	7.74E-03	2.23596487	1.233247768	4.053961439	8.04E-03
PARL	1.28E-02	0.221261215	0.07807525	0.627042827	4.54E-03
PARK7	3.42E-02	4.679417639	1.692283772	12.93928938	2.94E-03
PARD3B	8.11E-05	0.051558821	0.00897575	0.296166007	8.87E-04
PAQR8	1.98E-02	1.574517489	1.103629039	2.246321216	1.23E-02
PAQR7	9.75E-03	0.322884332	0.113993083	0.914566822	3.33E-02
PAPSS2	1.46E-03	4.555196531	2.35573565	8.80821048	6.58E-06
PAPPA-AS2	2.27E-03	289182761.5	3220.781959	2.59647E+13	8.14E-04

PANX2	5.35E-07	2.00733799	1.37029552	2.940537823	3.47E-04
PANX1	7.50E-03	2.38437307	1.322706052	4.298184717	3.85E-03
PAM16	1.60E-03	11.73960793	2.554272199	53.95603272	1.55E-03
PAM	2.08E-05	2.383878765	1.459322716	3.894188655	5.21E-04
PALMD	4.63E-04	0.490140457	0.288065638	0.833968499	8.55E-03
PALM	7.71E-05	0.481750691	0.311978312	0.743909817	9.86E-04
PAIP2B	2.36E-05	0.010614549	0.000790177	0.142586584	6.05E-04
PAIP2	4.83E-02	2.009084738	1.051852777	3.837439586	3.46E-02
PAICSP3	1.87E-02	1.69E+24	620976.3976	4.61E+42	1.00E-02
PAG1	4.71E-03	3.066597318	1.666392505	5.64333978	3.17E-04
PAFAH1B1	3.40E-02	2.293799418	1.142163655	4.606621607	1.96E-02
PAF1	2.83E-02	8.799606203	1.836268396	42.16870992	6.53E-03
PAEP	1.99E-04	1.884522741	1.3468192	2.636898821	2.18E-04
PADI3	6.58E-05	39.62784484	1.431610165	1096.922979	2.99E-02
PADI1	5.97E-05	5.932487777	2.892536553	12.16731771	1.19E-06
PACSIN3	6.37E-05	0.168248055	0.068743283	0.411784345	9.51E-05
PACS1	2.61E-02	0.316356129	0.116740035	0.85729973	2.37E-02
PACRG	2.50E-03	0.018405527	0.000908153	0.373024656	9.26E-03
PABPC4	1.12E-02	0.247745459	0.09228892	0.665061556	5.61E-03
PABPC1P12	3.46E-02	1.22E+46	16.83442255	8.87E+90	4.41E-02
P4HB	2.54E-04	4.718742905	1.714543884	12.98685604	2.67E-03
P4HA2	1.08E-05	5.769449878	2.494646692	13.34319285	4.19E-05
P4HA1	3.89E-04	2.602581446	1.543090573	4.389522107	3.35E-04
P3H4	2.91E-02	3.814820556	1.411557319	10.30978741	8.30E-03
P2RY8	2.67E-03	5.711540394	2.250919891	14.49260536	2.45E-04
P2RY6	1.43E-03	3.249881716	1.49448347	7.067144854	2.94E-03
P2RY11	2.56E-05	0.23067336	0.0742783	0.716362639	1.12E-02
P2RY10	4.55E-03	3.19944452	1.385452219	7.388522751	6.46E-03
P2RX6	3.51E-06	2.903939242	1.696828896	4.969778121	1.01E-04
P2RX5	2.02E-02	37.57949125	1.572677483	897.9706126	2.51E-02
P2RX4	6.44E-08	7.27149145	3.102656205	17.04171665	4.98E-06
OXLD1	3.92E-02	5.564870489	1.530016332	20.24016535	9.17E-03
OXCT2P1	6.84E-03	5.889157379	1.943338307	17.84669942	1.72E-03

OXCT2	4.15E-02	15.17130948	2.249005986	102.3423827	5.24E-03
OVOL1	1.50E-04	0.370086941	0.223533715	0.612723428	1.11E-04
OTUD7A	7.59E-03	0.055026745	0.008764991	0.345458727	1.98E-03
OTUD6A	8.80E-03	2.08E+26	41475.44866	1.04E+48	1.75E-02
OSR2	5.71E-05	12.54514122	2.639266554	59.63041812	1.47E-03
OSGIN2	3.62E-03	3.119460347	1.654453132	5.881721679	4.38E-04
OSGEP	2.92E-02	10.43947603	2.263109635	48.15615567	2.64E-03
OSBPL9P1	1.85E-09	1.21E+99	2.70E+51	5.39E+146	4.59E-05
OSBPL2	7.69E-04	0.351550566	0.159360704	0.77552243	9.61E-03
OSBP2	5.62E-06	0.213507065	0.095698818	0.476340958	1.62E-04
ORMDL2	6.71E-08	8.259972195	3.361937989	20.29399141	4.15E-06
ORMDL1	1.23E-02	3.35140268	1.33810637	8.39387673	9.83E-03
ORAI2	2.29E-06	25.55600149	6.13346424	106.4829249	8.55E-06
ORAI1	4.71E-02	1.721900737	1.022911522	2.898532362	4.08E-02
OR7E62P	1.65E-03	3.327E+19	1244.189166	8.90E+35	1.98E-02
OR7E22P	1.03E-05	9.68E-16	3.26E-25	2.87E-06	1.89E-03
OR7E159P	6.31E-04	67795745.19	164.5293372	2.79358E+13	6.27E-03
OR7E122P	4.87E-04	5.68E-06	2.53E-10	0.127303096	1.81E-02
OR7E10P	1.99E-03	1.78E+51	700479619.2	4.52E+93	1.78E-02
OR6X1	2.76E-06	1.03E+62	1.32E+20	8.00E+103	3.71E-03
OR6V1	1.65E-03	1.10E+44	346315851.7	3.47E+79	1.50E-02
OR6D1P	1.99E-03	3.81E+48	242536496.1	6.00E+88	1.78E-02
OR6C69P	1.06E-02	2.33E+26	46.13090091	1.18E+51	3.64E-02
OR5M14P	7.23E-05	3.81537E+13	5918.171134	2.46E+23	6.65E-03
OR5B12	5.62E-03	2.24E+30	7397709.308	6.76E+53	1.13E-02
OR52B2	1.37E-05	8.01E+51	4.14E+21	1.55E+82	7.83E-04
OR51M1	1.50E-04	2.98E+44	1.69583E+15	5.24E+73	2.88E-03
OR51K1P	3.82E-09	4.14E+54	1.41317E+19	1.21E+90	2.54E-03
OR51E1	3.45E-02	26.55060521	3.07374074	229.3409551	2.88E-03
OR4F15	4.76E-04	5.76795E+16	1883523.351	1.77E+27	1.73E-03
OR4F13P	4.30E-05	439011.295	39.11536759	4927242896	6.32E-03
OR2W3	1.61E-03	0.06784936	0.006638415	0.693469049	2.33E-02
OR2N1P	1.15E-03	162.2462	3.099419052	8493.149516	1.17E-02

OR2M1P	1.99E-03	1.57E+49	309794529.5	8.01E+89	1.78E-02
OR2L2	6.80E-03	3.49E-09	5.08E-17	0.239820413	3.44E-02
OR2L1P	9.66E-05	1.80E-34	5.06E-61	6.43E-08	1.27E-02
OR2I1P	5.79E-03	1.667890285	1.238457566	2.246227953	7.57E-04
OR2D3	1.88E-02	8.50E+40	1.706779715	4.23E+81	4.87E-02
OR1X5P	2.76E-06	3.04E+52	1.07051E+17	8.62E+87	3.71E-03
OR1K1	1.13E-03	1.05941E+19	2434338.935	4.61E+31	3.17E-03
OR1AA1P	1.88E-02	1.21E+35	1.581309304	9.30E+69	4.87E-02
OR11K1P	5.16E-03	6.66E+40	67855.85958	6.55E+76	2.62E-02
OR10J3	2.00E-02	5.23E+25	2067.025601	1.32E+48	2.44E-02
OR10D4P	1.88E-02	2.58E+36	1.608990049	4.15E+72	4.87E-02
OR10AE3P	6.80E-03	2.39248E+12	12129.30568	4.72E+20	3.45E-03
OR10AB1P	8.98E-03	843.8077199	9.225704728	77176.91917	3.45E-03
OR10A3	5.16E-03	7.10E+47	460587.2246	1.09E+90	2.62E-02
OPRD1	8.75E-05	13428.02658	48.26435113	3735922.968	9.33E-04
OPN5	4.94E-02	8.67E+24	3.96303E+12	1.89E+37	7.47E-05
OPA3	4.58E-04	7.12295145	1.203695961	42.15054216	3.04E-02
OOSP1P2	5.16E-03	1.17397E+16	79.77754722	1.73E+30	2.62E-02
OOSP1	6.34E-03	30.54062655	3.809381412	244.8507433	1.29E-03
ONECUT1	7.67E-04	108.2768678	4.215708778	2780.998574	4.67E-03
OLIG1	2.45E-05	2.011122282	1.344524819	3.008209871	6.71E-04
OLFML2B	2.06E-03	2.122823015	1.303561806	3.456972684	2.48E-03
OLFM1	1.14E-02	2.259407563	1.339749457	3.810356116	2.24E-03
OIP5-AS1	1.34E-02	2.090657154	1.049659363	4.164062638	3.59E-02
OGG1	6.28E-06	0.042429571	0.006762943	0.266196039	7.45E-04
OGFRP1	6.17E-04	5.69E-05	1.02E-07	0.031676295	2.44E-03
OGDHL	1.73E-02	0.477437805	0.281818937	0.80884152	5.98E-03
OGDH	6.68E-03	2.68644751	1.136612364	6.349570402	2.43E-02
OFD1P8Y	7.23E-05	1.96E+61	1.05631E+17	3.65E+105	6.65E-03
ODF3L1	2.23E-03	2.759036579	1.514739561	5.025473052	9.09E-04
ODF1	4.88E-02	1.78886E+13	926.2122993	3.45E+23	1.16E-02
OCLN	3.41E-02	0.003341664	1.55E-05	0.718992781	3.75E-02
OBSL1	1.06E-02	0.174682423	0.059244696	0.515049464	1.56E-03

OARD1	1.27E-03	0.188407788	0.072774923	0.487770969	5.83E-04
OAF	4.79E-04	1.981968893	1.266390546	3.101887251	2.76E-03
NYNRIN	1.37E-04	0.494452029	0.300786343	0.812812199	5.48E-03
NXT1P1	3.91E-02	237739.6175	1.028504787	54953682708	4.95E-02
NXF5	2.87E-02	5.69E-48	6.24E-93	0.005184617	3.94E-02
NUTM2D	1.72E-02	0.000552465	2.22E-06	0.137433456	7.70E-03
NUP88	5.88E-03	4.912223227	1.929645192	12.50485692	8.41E-04
NUP85	1.02E-02	8.614719828	2.067934664	35.88768978	3.10E-03
NUP50-AS1	9.94E-03	2.144988303	1.120575929	4.105901885	2.12E-02
NUMBL	7.55E-03	5.534296672	1.710434583	17.90681734	4.29E-03
NUMA1	4.72E-02	0.324341871	0.117301172	0.896816692	3.00E-02
NUDT22	6.71E-03	4.837116927	1.927661256	12.13786919	7.85E-04
NUDT19	2.64E-02	2.197603581	1.045581766	4.618922839	3.77E-02
NUDT16L1	4.11E-04	8.275573716	2.594955447	26.39163629	3.55E-04
NUDT16	6.19E-03	0.273791363	0.09695443	0.773164369	1.45E-02
NUDT15	1.73E-04	10.4286997	3.277409964	33.1840626	7.19E-05
NUDT14	2.21E-04	3.048361869	1.75038573	5.308835603	8.22E-05
NUCB2	2.59E-02	2.234033358	1.143069732	4.366229731	1.87E-02
NUAK2	1.10E-03	5.250763866	1.856827769	14.84818443	1.77E-03
NTS	1.17E-05	48.28935433	3.005363121	775.9001651	6.21E-03
NTNG1	1.36E-02	0.449470902	0.204701994	0.986918044	4.63E-02
NTF3	8.86E-03	6.482637079	2.13748374	19.66077342	9.60E-04
NT5M	2.67E-02	3.713196424	1.492495087	9.23810591	4.79E-03
NT5E	5.95E-03	1.394111675	1.097234601	1.771314321	6.54E-03
NT5DC3	9.44E-04	2.358222949	1.455023218	3.82208023	4.97E-04
NSMF	3.27E-02	2.78219476	1.288781537	6.006144142	9.16E-03
NSMCE2	1.94E-02	2.115944	1.221366344	3.665746179	7.51E-03
NSMCE1	1.43E-02	4.482730124	1.757062725	11.43662607	1.69E-03
NSMAF	5.86E-05	2.862949853	1.707778245	4.7995001	6.60E-05
NSF	6.35E-03	1.814556247	1.104657037	2.980666634	1.86E-02
NRTN	1.49E-04	0.158427894	0.06154967	0.407790935	1.34E-04
NRSN2	1.00E-02	2.504989581	1.431548125	4.383347435	1.30E-03
NRROS	1.82E-05	0.519293343	0.351468718	0.767253421	1.00E-03

NRP1	5.50E-03	2.337756362	1.042598662	5.241810689	3.93E-02
NRIP2	2.19E-02	2.448738725	1.21061321	4.953127306	1.27E-02
NRGN	1.44E-02	2.268135918	1.27903021	4.022141544	5.08E-03
NRADDP	1.47E-04	3.56E-05	9.88E-08	0.012855341	6.51E-04
NR6A1	5.52E-07	0.056892103	0.012116758	0.26712685	2.80E-04
NR5A2	4.15E-04	119151.8035	223.9941331	63381804.21	2.62E-04
NR2F2	2.28E-02	2.298780421	1.002725605	5.270027409	4.93E-02
NR0B1	1.55E-02	1764.365037	56.92435074	54686.33273	1.98E-05
NQO1	1.22E-05	2.073706193	1.518667536	2.831598933	4.45E-06
NPTX2	1.04E-03	2.185434734	1.291024563	3.699484201	3.60E-03
NPTX1	1.67E-03	1.623034071	1.095340669	2.404950049	1.58E-02
NPRL2	3.42E-03	0.112101076	0.032635342	0.38506265	5.09E-04
NPM1P45	1.25E-02	1154123505	149.6166574	8.90276E+15	9.91E-03
NPLOC4	9.17E-03	3.754603331	1.065061159	13.23590298	3.96E-02
NPEPL1	4.19E-03	3.740119278	1.102514694	12.68780569	3.43E-02
NPBWR1	3.09E-02	745.6497732	3.801228323	146266.8214	1.41E-02
NPAS1	1.28E-03	4.349194963	1.893584916	9.989251955	5.30E-04
NOXA1	3.84E-04	0.342411533	0.16859091	0.695444722	3.03E-03
NOV	8.19E-04	1.874910375	1.246392131	2.820371555	2.55E-03
NOTUM	5.61E-04	140.6026877	3.143744399	6288.397934	1.08E-02
NOTCH3	7.43E-03	2.440745029	1.451749216	4.103488557	7.62E-04
NOTCH2	3.05E-02	0.331379989	0.152756462	0.718874316	5.18E-03
NOP58	3.99E-02	1.808450451	1.131979515	2.889180405	1.32E-02
NOP56	7.97E-03	5.284569342	1.733816774	16.10704981	3.41E-03
NOP2	1.93E-02	4.206046222	1.346060282	13.14266905	1.35E-02
NOP16	7.85E-03	6.160490226	2.117137743	17.92591906	8.49E-04
NOP10	3.20E-03	4.470527506	1.691507202	11.81527111	2.53E-03
NOLC1	2.98E-02	2.766784033	1.128849684	6.781322608	2.61E-02
NOL10	2.19E-02	3.230312618	1.397262876	7.468114832	6.10E-03
NOG	3.78E-03	7.416863279	1.094403241	50.26470943	4.01E-02
NOD2	3.91E-02	4.085460161	1.484778052	11.24140049	6.42E-03
NOA1	4.98E-03	0.375683964	0.182811305	0.77204438	7.72E-03
NMRK2	9.24E-03	0.7202028	0.530340229	0.978036446	3.55E-02

NMNAT3	4.96E-04	0.104414461	0.037481164	0.290876231	1.54E-05
NME6	3.33E-03	0.241595004	0.082965098	0.70352651	9.19E-03
NME4	8.26E-04	2.489248301	1.117852917	5.543088014	2.56E-02
NME1	7.34E-05	5.714349818	2.602102214	12.54900506	1.41E-05
NMB	1.46E-02	2.656453589	1.14727932	6.150852321	2.26E-02
NLRX1	4.13E-02	0.214630905	0.072381962	0.636435155	5.52E-03
NLRP9	1.65E-02	5.71E-09	3.55E-16	0.091752006	2.50E-02
NLRP6	1.77E-04	37.10588875	6.71228838	205.1233353	3.44E-05
NLRP11	4.32E-05	1.82E-14	3.53E-27	0.094014356	3.42E-02
NLRC5	9.22E-03	1.829370007	1.305327938	2.56379606	4.53E-04
NLN	3.35E-03	3.1716621	1.455020658	6.913606635	3.69E-03
NLE1	7.45E-03	0.302484549	0.12505162	0.731673071	7.97E-03
NKX6-1	1.08E-06	23.11848006	5.381873152	99.30819722	2.41E-05
NKX3-2	4.86E-06	397886.0792	1479.014015	107039778.1	6.27E-06
NKPD1	1.64E-04	8.30685889	1.380237689	49.99421853	2.08E-02
NKIRAS2	2.31E-02	0.208498148	0.057546255	0.755418011	1.70E-02
NKIRAS1	4.69E-07	0.186441856	0.077024072	0.451294829	1.96E-04
NKG7	4.07E-05	1.481788692	1.208241617	1.817267091	1.59E-04
NKD2	2.34E-02	4.904195543	1.642079155	14.64675673	4.39E-03
NKAPP1	3.88E-02	0.046813923	0.003470608	0.631458065	2.11E-02
NIT1	1.99E-03	9.433768769	2.399868844	37.0836904	1.31E-03
NISCH	1.49E-05	0.124960287	0.047541068	0.328454406	2.47E-05
NIPSNAP3B	6.45E-05	0.160463362	0.048281193	0.533302701	2.83E-03
NIPSNAP1	1.33E-02	0.313956765	0.124348701	0.792680981	1.42E-02
NIPA2P1	4.42E-11	8.71E+55	3.71E+28	2.04E+83	6.18E-05
NIPA2	2.45E-02	2.387479122	1.160063454	4.913573079	1.81E-02
NINJ2	3.00E-03	7.744665154	2.305771113	26.0129195	9.28E-04
NID1	1.28E-02	2.873636899	1.576709566	5.237355825	5.67E-04
NICN1	3.24E-07	0.131079324	0.057276185	0.299981381	1.51E-06
NHSL2	1.54E-03	2409.56505	47.21298179	122974.7308	1.04E-04
NHSL1	1.42E-03	0.431527401	0.243977156	0.763251367	3.87E-03
NHP2	3.99E-03	9.366124718	1.938998103	45.24207223	5.37E-03
NHEJ1	9.46E-03	0.041979603	0.004674918	0.376966411	4.64E-03

NGFRAP1	5.82E-03	0.386649637	0.165079145	0.905613741	2.86E-02
NFU1	1.41E-02	3.064491833	1.318956793	7.120104497	9.23E-03
NFIC	5.77E-04	4.031804813	1.451847012	11.19639322	7.46E-03
NFIA	5.83E-06	0.208069148	0.089179994	0.485453833	2.81E-04
NFE2L1	1.04E-02	0.358238969	0.194012775	0.661477882	1.04E-03
NFATC4	1.11E-04	2.660691773	1.58744592	4.459541343	2.04E-04
NFATC2	4.56E-04	4.588841585	2.056238983	10.24076835	1.99E-04
NFATC1	5.70E-04	0.287204392	0.107537678	0.767046163	1.28E-02
NFAM1	8.64E-03	3.21982972	1.582050859	6.553078472	1.26E-03
NF1P8	2.31E-05	5.28E+62	7.48E+23	3.72E+101	1.55E-03
NF1P2	3.32E-06	2.72E+62	2.92E+23	2.54E+101	1.69E-03
NF1P1	5.91E-05	1.18E+33	323343.6471	4.29E+60	1.87E-02
NF1	1.93E-02	2.853517339	1.100894373	7.396314672	3.09E-02
NEXN	1.04E-03	5.63930771	1.776841926	17.89792945	3.33E-03
NEURL1B	1.27E-02	3.98521109	1.67215137	9.4978886	1.81E-03
NEO1	2.45E-02	0.38840015	0.229249984	0.658035711	4.39E-04
NENFP2	7.23E-05	1.39098E+19	207462.1616	9.33E+32	6.65E-03
NENFP1	3.15E-02	17421455.51	1.385324546	2.19087E+14	4.56E-02
NENF	1.38E-02	3.179569538	1.514403412	6.675673314	2.24E-03
NELFCD	8.43E-03	4.710109068	1.744402941	12.71789156	2.23E-03
NELFB	2.89E-02	0.240286573	0.090995421	0.634511453	4.00E-03
NEK4P2	3.56E-02	1.29E+28	27.17809216	6.14E+54	3.89E-02
NEK11	7.83E-04	0.084907448	0.017216741	0.418736322	2.45E-03
NEIL3	1.42E-02	24.95860287	4.325179636	144.0245053	3.21E-04
NEDD9	7.30E-07	0.244218874	0.122502336	0.486871194	6.21E-05
NEDD8	2.16E-02	12.99482851	3.184856041	53.02141317	3.51E-04
NECAB2	2.66E-04	2.192647415	1.54110406	3.119648317	1.28E-05
NDUFV2P1	2.85E-02	6.384465381	2.157291559	18.89470991	8.12E-04
NDUFV2	1.02E-03	6.847562218	2.628894629	17.83605467	8.19E-05
NDUFS5P2	2.69E-05	82183040898	14102.0667	4.78941E+17	1.57E-03
NDUFS4	1.23E-02	2.77388599	1.102005206	6.982220631	3.03E-02
NDUFS3	7.02E-03	8.840136174	2.366365679	33.02448487	1.19E-03
NDUFS2	2.19E-02	5.001921796	1.479807936	16.90707358	9.58E-03

NDUFB9	1.52E-04	3.836601619	2.056637325	7.15707714	2.37E-05
NDUFB8	7.16E-04	13.67552962	4.196000458	44.57104146	1.43E-05
NDUFB4	3.13E-02	0.107283315	0.028850463	0.398943666	8.64E-04
NDUFB3	1.52E-04	10.6300434	3.062954434	36.891774	1.97E-04
NDUFB2	3.21E-05	12.29134608	3.866924424	39.06908229	2.12E-05
NDUFB1	1.78E-05	9.155971962	3.177723202	26.38109654	4.11E-05
NDUFAF6	2.68E-02	3.054798807	1.466240163	6.364438779	2.87E-03
NDUFAF5	2.98E-02	3.457124367	1.254367401	9.528076765	1.65E-02
NDUFAF4P2	1.46E-09	519.3882001	5.200634307	51871.38463	7.77E-03
NDUFAF1	2.89E-04	5.85739421	2.436618773	14.08060519	7.81E-05
NDUFAB1	2.04E-02	4.705030564	1.415447967	15.63979258	1.15E-02
NDUFA9	1.31E-06	13.55914045	3.688319766	49.84662434	8.68E-05
NDUFA8	1.04E-03	3.027857764	1.233460157	7.432686488	1.56E-02
NDUFA6	7.11E-05	5.065635156	2.420359114	10.60200504	1.66E-05
NDUFA5P5	1.66E-03	17147182.17	10.04584036	2.92684E+13	2.29E-02
NDUFA5P2	1.20E-02	290073735.2	274.8444089	3.06147E+14	5.89E-03
NDUFA5P10	1.10E-05	1.50988E+17	8748527.276	2.61E+27	1.01E-03
NDUFA4	1.40E-02	5.123300541	1.631468008	16.08870558	5.14E-03
NDUFA3P6	7.23E-05	1.19149E+11	1192.106984	1.19087E+19	6.65E-03
NDUFA2	7.76E-05	16.81151048	4.329447568	65.28012639	4.56E-05
NDUFA13	5.15E-03	2.05924937	1.126240903	3.765187322	1.90E-02
NDUFA12	7.50E-05	5.894563552	2.012484539	17.26516591	1.21E-03
NDUFA1	1.95E-04	12.72369763	3.669552066	44.11777744	6.09E-05
NDRG3	7.49E-04	2.123044341	1.116584134	4.036701881	2.17E-02
NDOR1	2.87E-03	0.084497383	0.019697997	0.362463644	8.81E-04
NDNL2	3.69E-03	7.986837491	2.145060152	29.73789479	1.95E-03
NDN	6.43E-06	0.680809987	0.546631993	0.847923731	5.97E-04
NDC80	2.44E-03	4.152050825	1.768333074	9.749026529	1.08E-03
NCSTN	5.13E-04	7.353297863	1.829109876	29.56136763	4.95E-03
NCS1	4.86E-05	4.800797513	2.036926585	11.31491775	3.35E-04
NCR3LG1	3.19E-04	4.561722704	2.137948743	9.7333082	8.67E-05
NCR3	4.41E-03	11.24175096	2.639029556	47.88766549	1.07E-03
NCLN	1.82E-03	4.909444344	1.809647461	13.31897194	1.78E-03

NCKIPSD	1.77E-05	0.142355156	0.05625313	0.360246447	3.87E-05
NCKAP5	3.77E-05	0.04017846	0.006819952	0.236703824	3.82E-04
NCKAP1L	1.05E-02	2.189423233	1.264651536	3.790430768	5.13E-03
NCF4	1.11E-03	2.134911888	1.393535521	3.270708713	4.93E-04
NCF1C	9.30E-03	2.303442956	1.394364193	3.805210632	1.12E-03
NCF1B	2.39E-02	3.599021222	1.461200258	8.864598595	5.36E-03
NCF1	4.13E-02	2.51595792	1.404351847	4.507448947	1.93E-03
NCBP2-AS2	2.29E-03	0.221352972	0.088672819	0.552560963	1.23E-03
NCAPH	1.03E-02	8.049292279	2.894318306	22.38561877	6.43E-05
NCAPD2	1.40E-02	0.282918393	0.094607111	0.846054973	2.39E-02
NCALD	9.52E-03	2.124602068	1.265817528	3.566022625	4.34E-03
NBEAL2	1.25E-03	0.35571235	0.146515242	0.863604868	2.24E-02
NATD1	3.56E-07	0.088104541	0.030380832	0.255503537	7.76E-06
NAT8B	7.98E-03	23.29791219	1.813030236	299.3842582	1.57E-02
NAT6	1.94E-04	0.085544057	0.023942459	0.30564053	1.54E-04
NAT1	7.31E-03	5.493814678	1.75728172	17.17539048	3.40E-03
NASP	4.03E-02	2.753813403	1.363283404	5.562664545	4.75E-03
NARS	1.90E-02	2.655904415	1.036754835	6.803757289	4.18E-02
NARF	4.09E-02	0.25621101	0.113182972	0.579981959	1.09E-03
NAPSA	2.56E-05	0.001917729	4.24E-05	0.086661682	1.29E-03
NAPRT	9.82E-05	3.451856858	1.731643024	6.880930769	4.32E-04
NAP1L4P3	1.90E-02	2.71E-08	1.22E-14	0.060053318	1.94E-02
NAP1L4P1	2.66E-04	0.002568035	3.95E-05	0.16698951	5.11E-03
NANS	9.29E-04	3.842393213	1.873250707	7.881478729	2.40E-04
NANOGP1	2.50E-04	4.62E+44	2.10E+20	1.01E+69	3.23E-04
NAMA_1	2.15E-02	1.639211196	1.099660251	2.443494109	1.53E-02
NAMA	5.78E-03	3.588400957	1.71871456	7.49200695	6.69E-04
NAGLU	3.86E-02	2.089222008	1.0816234	4.035460586	2.83E-02
NADK	3.37E-02	2.439761765	1.076702034	5.528398095	3.26E-02
NACC2	1.75E-03	3.053294743	1.482259128	6.289459523	2.47E-03
NACC1	1.26E-02	4.511736754	1.105316217	18.41623983	3.58E-02
NAAA	6.59E-03	3.742592543	1.929138167	7.260754664	9.49E-05
NAA38	2.52E-03	6.003006058	2.531846713	14.23312144	4.72E-05

NAA10	2.60E-02	7.838117736	2.299904423	26.71245337	9.97E-04
N6AMT2	5.68E-05	8.658905744	2.799665162	26.78057708	1.79E-04
MZB1	6.22E-03	1.727319854	1.23565155	2.414623993	1.38E-03
MYOM1	3.49E-03	0.028879477	0.001901522	0.438608774	1.07E-02
MYO9B	1.36E-03	0.23381456	0.099097122	0.551673425	9.07E-04
MYO7B	1.06E-03	0.053920694	0.004267601	0.681282308	2.40E-02
MYO1G	6.30E-06	5.070088023	2.477255485	10.37672243	8.89E-06
MYO1F	2.62E-03	2.725955759	1.470540894	5.053130335	1.45E-03
MYO19	1.69E-02	5.367567725	1.567799081	18.37657875	7.45E-03
MYO15A	2.40E-02	3325.493043	2.234803833	4948489.801	2.96E-02
MYLKP1	4.01E-04	4.04E-25	6.89E-45	2.37E-05	1.56E-02
MYLK-AS1	1.70E-03	0.158931572	0.053653639	0.470783434	9.01E-04
MYLIP	2.67E-02	0.498199303	0.284561806	0.872227193	1.48E-02
MYL6	3.60E-02	7.328303535	2.095565359	25.62746729	1.82E-03
MYH7	1.21E-04	0.019464475	0.001733611	0.218541399	1.41E-03
MYH14	1.07E-05	0.389030426	0.276679749	0.54700307	5.65E-08
MYEOV	3.50E-05	1.815714383	1.381862223	2.385779615	1.86E-05
MYDGF	6.89E-04	11.67428701	2.699698491	50.48303636	1.00E-03
MYCNUT	3.41E-06	1.919093897	1.2237876	3.009444929	4.51E-03
MYCN	1.67E-03	3.379096178	1.279779855	8.922074321	1.40E-02
MYBL2	1.28E-02	2.490925025	1.311887403	4.729603671	5.27E-03
MXRA7	3.44E-04	2.676990224	1.445756722	4.956765237	1.73E-03
MVD	7.06E-03	0.329959996	0.123661786	0.88041425	2.68E-02
MVB12B	2.32E-04	0.061235315	0.016753086	0.223825258	2.41E-05
MUCL1	1.41E-03	46164193.74	25.01784677	8.51845E+13	1.65E-02
MUC7	7.95E-03	0.192094089	0.038571297	0.95667353	4.40E-02
MUC19	4.29E-02	2.27E-118	1.21E-225	4.25E-11	3.16E-02
MUC12	1.08E-04	94724.30918	105.8259944	84787247.24	9.52E-04
MTX1	3.02E-02	6.424772734	2.142502393	19.26611836	9.01E-04
MTUS2-AS1	1.27E-03	2.28382E+17	84268639.65	6.19E+26	3.10E-04
MTUS1	8.30E-06	0.181663138	0.078025445	0.42295812	7.64E-05
MTND5P9	1.10E-05	6.23E+82	2.88E+33	1.35E+132	1.00E-03
MTND5P8	2.57E-04	1.26E+40	1.81571E+17	8.73E+62	5.80E-04

MTND5P26	3.62E-02	1.56E+33	61.66645854	3.97E+64	3.83E-02
MTND2P29	1.40E-02	6.38E-13	8.46E-25	0.48185893	4.42E-02
MTND1P32	1.39E-03	2.8663E+16	2113.811051	3.89E+29	1.40E-02
MTND1P3	5.21E-03	2.08058E+15	746.8731031	5.80E+27	1.58E-02
MTND1P14	3.25E-02	157246707.2	1.008201031	2.45254E+16	4.99E-02
MTMR14	4.00E-06	0.043425996	0.010267315	0.183671894	2.02E-05
MTHFR	3.66E-02	0.431011048	0.216301718	0.858849045	1.67E-02
MTHFD2P7	6.36E-03	5151607889	32.62226123	8.13526E+17	2.02E-02
MTHFD2P1	3.90E-08	5.47E+46	9.9068E+17	3.02E+75	1.44E-03
MTHFD1	3.25E-02	4.12424909	1.222553116	13.91304012	2.24E-02
MTG1	4.62E-02	5.76667911	1.947291921	17.07735116	1.56E-03
MTFR1	6.19E-03	2.19351953	1.369494241	3.513361198	1.08E-03
MTERF3	3.60E-02	2.058328379	1.310935488	3.231826243	1.71E-03
MTDH	3.25E-02	1.7448331	1.21139427	2.513172317	2.79E-03
MTCH2	7.21E-04	5.548604196	1.886866178	16.31647696	1.85E-03
MT2A	6.22E-03	1.454773301	1.090375552	1.940950854	1.08E-02
MT1L	2.45E-02	1.649174087	1.056933815	2.57326914	2.75E-02
MT1E	1.78E-02	1.483006646	1.098058584	2.002906535	1.02E-02
MT1DP	1.27E-04	2.762411025	1.299993196	5.869965084	8.24E-03
MT1A	7.36E-04	2.619763915	1.595044414	4.302803678	1.42E-04
MT-TT	1.34E-02	1.774458753	1.26384133	2.491375927	9.25E-04
MT-TR	3.84E-03	4.207719391	1.545714276	11.45418836	4.92E-03
MT-TP	2.67E-02	0.584486892	0.378368415	0.902889655	1.55E-02
MT-TM	2.79E-04	2.33326245	1.501729264	3.625229789	1.64E-04
MT-TL1	4.63E-03	2.244393802	1.46448282	3.439646727	2.06E-04
MT-RNR1	3.47E-02	0.533158349	0.31297679	0.908239314	2.07E-02
MSX2	4.24E-03	3.072495788	1.182251021	7.984962757	2.12E-02
MSX1	7.94E-05	5.481830689	2.358440829	12.74166701	7.69E-05
MSRB1	5.89E-05	8.37106122	2.279384885	30.74279663	1.37E-03
MSR1	9.40E-03	2.460028014	1.172688261	5.160568267	1.72E-02
MSN	1.12E-03	4.127967891	1.710093439	9.964437333	1.61E-03
MSC	6.09E-05	0.673118291	0.535649057	0.845867696	6.83E-04
MS4A7	1.87E-02	1.586447052	1.078417496	2.333803244	1.91E-02

MS4A6A	1.95E-02	1.6687484	1.112266531	2.503645613	1.34E-02
MS4A4E	1.60E-02	23.11286994	2.674000809	199.7773355	4.32E-03
MS4A12	2.93E-03	11515778.92	6.505538147	2.03847E+13	2.68E-02
MRPS7	1.35E-05	7.679986681	2.425398881	24.31855472	5.27E-04
MRPS6P4	5.16E-03	1.44422E+13	36.09714695	5.78E+24	2.62E-02
MRPS6	5.29E-05	3.533124909	1.468931532	8.497994185	4.82E-03
MRPS5	4.91E-04	11.18866922	1.984677391	63.07640707	6.20E-03
MRPS36	1.73E-02	2.900702242	1.067490896	7.882103281	3.68E-02
MRPS34	1.94E-04	5.161354939	1.85522684	14.35920623	1.67E-03
MRPS28	5.95E-04	2.313501745	1.494143921	3.582178564	1.70E-04
MRPS26	6.35E-03	3.233727703	1.136117284	9.204150844	2.79E-02
MRPS21P1	1.68E-02	1296.203747	1.077153734	1559799.777	4.76E-02
MRPS17	2.19E-03	15.23364081	2.579951873	89.94889201	2.65E-03
MRPS16	3.51E-04	14.09527217	3.452992642	57.53753862	2.27E-04
MRPS14	8.76E-03	4.63577532	1.378976583	15.58432034	1.32E-02
MRPS12	4.83E-03	7.939032356	2.273078334	27.72814022	1.17E-03
MRPS11	7.82E-06	9.168190862	3.356149316	25.04528725	1.55E-05
MRPL57	5.92E-03	21.85996649	4.619142174	103.4517053	1.01E-04
MRPL55	3.69E-02	4.824079237	1.718445672	13.54231958	2.81E-03
MRPL53P1	1.62E-02	160.5825517	5.158839634	4998.557377	3.79E-03
MRPL52	1.73E-05	13.4077528	4.388263021	40.96560169	5.23E-06
MRPL51	1.76E-04	10.04921816	2.177792999	46.37115908	3.10E-03
MRPL48P1	4.06E-03	2.32E-05	4.40E-09	0.122555789	1.47E-02
MRPL46	2.30E-02	7.227249157	1.53503358	34.02735357	1.23E-02
MRPL42P3	1.99E-03	1.31455E+17	900.6863199	1.92E+31	1.78E-02
MRPL42	4.13E-02	2.559480026	1.055079302	6.208953196	3.77E-02
MRPL41	2.16E-02	3.209538781	1.261446702	8.166131135	1.44E-02
MRPL40	7.91E-05	13.17059819	4.398897433	39.43366704	4.07E-06
MRPL4	4.13E-02	4.037660176	1.000654084	16.29204334	4.99E-02
MRPL39	4.62E-02	2.481751717	1.106079177	5.568400268	2.75E-02
MRPL36	2.48E-04	7.735309974	2.322000421	25.76873796	8.62E-04
MRPL35	1.58E-02	3.183172544	1.493439616	6.78473193	2.71E-03
MRPL27	1.29E-02	5.75912112	2.092300792	15.85215481	7.01E-04

MRPL24	4.30E-05	5.989013124	2.822593681	12.70755987	3.11E-06
MRPL23-AS1	1.01E-03	1.06E-06	7.49E-11	0.015116459	4.81E-03
MRPL22	7.92E-03	5.015353389	1.843328055	13.64584538	1.59E-03
MRPL17	7.00E-03	5.002598978	1.759864986	14.22040709	2.52E-03
MRPL16	2.57E-02	3.866163501	1.284233344	11.63902206	1.62E-02
MRPL15	3.40E-04	2.749785209	1.655788742	4.566596272	9.29E-05
MRPL13	5.10E-04	2.176069742	1.401071067	3.379756839	5.38E-04
MRPL12	7.94E-03	4.098031894	1.680383053	9.99406973	1.93E-03
MROH6	2.84E-02	1.998040262	1.0264283	3.889375312	4.17E-02
MROH2A	4.06E-02	3.34E+64	8.21761E+13	1.35E+115	1.25E-02
MROH1	1.77E-06	4.531424596	2.042634191	10.05261195	2.02E-04
MRM1	8.70E-05	3.716442124	1.551176783	8.904170183	3.23E-03
MRGBP	3.99E-04	6.84688295	2.491617747	18.81500731	1.91E-04
MRC2	7.12E-05	1.98156917	1.374559679	2.856635791	2.48E-04
MRAP	5.27E-03	3.646582115	1.35603428	9.806213098	1.04E-02
MR1	2.98E-03	2.854984351	1.456765487	5.595228414	2.24E-03
MPZ	3.42E-06	0.158928328	0.05733932	0.44050424	4.06E-04
MPV17L2	9.73E-04	11.4924978	3.717539914	35.52820107	2.23E-05
MPV17	3.89E-03	4.427609319	1.749747274	11.20374615	1.68E-03
MPRIPP1	8.65E-04	0.025573068	0.002481328	0.263561208	2.07E-03
MPRIP	7.77E-05	0.164738335	0.068035766	0.398889008	6.42E-05
MPL	7.61E-07	2.66E-13	1.02E-19	6.92E-07	1.22E-04
MPG	2.85E-03	9.554928368	2.372135607	38.48711509	1.50E-03
MPEG1	1.40E-03	1.856923833	1.251849581	2.754457223	2.09E-03
MPDU1	1.93E-02	2.468510043	1.344310467	4.532838195	3.57E-03
MOS	1.99E-03	6.55E+53	1942213354	2.21E+98	1.78E-02
MORN1	3.74E-03	0.073795528	0.009550031	0.5702369	1.25E-02
MORC2	1.40E-04	9.011234215	2.187263578	37.12508309	2.34E-03
MON1A	1.61E-02	0.140519543	0.040980146	0.481836795	1.80E-03
MOB2	1.50E-02	0.138113218	0.034894055	0.5466622	4.80E-03
MMP3	1.82E-03	2.88059E+11	4350087.323	1.9075E+16	3.18E-06
MMP23A	3.33E-02	4.878236028	1.06752488	22.29192703	4.09E-02
MMP2	1.34E-02	1.584047224	1.191368785	2.10615356	1.55E-03

MMP16	4.41E-02	46.83432302	7.663317484	286.2277098	3.11E-05
MMP13	3.90E-03	3.67043E+11	217618.2417	6.19068E+17	2.73E-04
MMP12	3.82E-06	2.045757485	1.46243103	2.861757992	2.92E-05
MMP11	5.74E-05	10.32135725	3.119415564	34.15076102	1.32E-04
MMP1	1.44E-03	3.739756965	1.719442329	8.133905933	8.78E-04
MMAA	2.13E-02	4.519164031	1.829289404	11.16435895	1.08E-03
MLLT4-AS1	1.07E-02	5.241143283	1.175171844	23.37494984	2.99E-02
MLLT1	2.97E-03	0.105493962	0.030629246	0.363344757	3.65E-04
MLIP	1.70E-06	0.375653507	0.237351454	0.594542629	2.92E-05
MKS1	5.31E-03	0.064694497	0.012451906	0.336123489	1.13E-03
MKRN2	1.81E-06	0.159866386	0.071419289	0.357848162	8.21E-06
MIRLET7BHG	8.10E-05	76.63124391	8.852726304	663.3377494	8.14E-05
MIR941-5	1.99E-03	11840.70576	5.047015424	27779251.93	1.78E-02
MIR887	8.40E-04	31.03935275	3.572046376	269.7169403	1.85E-03
MIR8084	1.88E-02	4831.522732	1.04929707	22246904.7	4.87E-02
MIR8082	1.26E-03	99.04318224	2.479002054	3957.056806	1.46E-02
MIR8071-1	4.94E-03	2.568478761	1.446436117	4.560922578	1.28E-03
MIR8069-1	5.16E-03	15867.51075	3.141520386	80145237.51	2.62E-02
MIR7158	5.28E-04	3275.571987	6.223656464	1723965.952	1.13E-02
MIR6894	7.23E-05	733.1642296	6.24873197	86022.21861	6.65E-03
MIR6870	2.18E-03	33.926981	3.344201401	344.1898086	2.87E-03
MIR6815	2.92E-02	24.10145407	1.24578732	466.2754862	3.53E-02
MIR6813	1.23E-02	7.005182933	1.113049229	44.08842542	3.81E-02
MIR6731	2.76E-06	49947.18666	33.46417072	74549029.65	3.71E-03
MIR664A	5.28E-04	9968.452964	8.00247482	12417415.45	1.13E-02
MIR635	1.69E-05	2.027007511	1.325882595	3.09888633	1.10E-03
MIR615	1.99E-03	203243.1119	8.243734519	5010806986	1.78E-02
MIR613	1.85E-09	7640180.453	3691.64668	15812010851	4.73E-05
MIR6076	2.07E-02	2132.196087	1.491260499	3048602.278	3.87E-02
MIR6074	5.16E-03	549.9018446	2.110177831	143301.6849	2.62E-02
MIR602	1.86E-02	11.79825935	1.120086489	124.2751565	3.99E-02
MIR5691	1.06E-02	200.7314471	1.397368728	28834.99038	3.64E-02
MIR5687	5.16E-03	6259.853835	2.814051935	13925034.41	2.62E-02

MIR5579	1.99E-03	2250.004046	3.789325123	1335994.681	1.78E-02
MIR556	7.23E-05	121216.4512	25.81830472	569108940.8	6.65E-03
MIR497HG	1.04E-03	1658.657928	30.575007	89980.22866	2.74E-04
MIR4777	1.96E-02	5.578945406	1.288856074	24.14903609	2.15E-02
MIR4740	1.61E-03	4.051497156	2.140945563	7.6669998	1.71E-05
MIR4659B	1.39E-03	31.83798921	2.441980616	415.0964796	8.26E-03
MIR4655	2.51E-07	2.458755121	1.732432137	3.489589357	4.75E-07
MIR4644	2.20E-03	30.96367621	5.845859522	164.0048381	5.44E-05
MIR4537	7.58E-03	7.11343639	1.629082442	31.06102918	9.08E-03
MIR4477A	2.76E-06	167311.8575	49.53794236	565087210.4	3.71E-03
MIR4470	1.21E-03	94.14279166	4.597022122	1927.957923	3.18E-03
MIR4466	1.99E-03	1399.270022	3.491074946	560846.337	1.78E-02
MIR4449	4.97E-02	8.0153969	1.185373118	54.19946385	3.28E-02
MIR4436A	2.76E-06	286247.1266	58.9673668	1389538349	3.71E-03
MIR4435-1HG	9.58E-05	3.770702505	1.994109758	7.13009769	4.44E-05
MIR4428	5.16E-03	4139.559902	2.679630109	6394896.117	2.62E-02
MIR4421	1.17E-03	297.7335543	12.74110718	6957.422782	3.96E-04
MIR4294	2.33E-02	155.207168	1.309647532	18393.70092	3.84E-02
MIR4269	5.79E-04	122.6577101	11.31856107	1329.224956	7.63E-05
MIR4265	3.04E-05	106764.2924	104.2506144	109338579.8	1.06E-03
MIR4257	2.89E-02	12.86218851	3.342978333	49.48757564	2.03E-04
MIR3928	1.65E-03	512.587544	2.728611288	96292.93532	1.95E-02
MIR375	3.82E-09	9693573.19	281.608484	3.33674E+11	2.54E-03
MIR3690	1.88E-02	1416.294492	1.042018287	1925004.688	4.87E-02
MIR3680-1	1.69E-02	68.87313756	4.061732661	1167.853592	3.38E-03
MIR320A	2.27E-02	16.9546189	3.07858787	93.3736876	1.15E-03
MIR3180-1	4.05E-02	2501.375125	11.71022845	534308.7493	4.25E-03
MIR3174	4.18E-04	27.71345521	4.966562824	154.6412734	1.52E-04
MIR3173	1.90E-02	15.8026292	1.256262344	198.7825957	3.26E-02
MIR3156-2	1.42E-02	66.40107728	5.773277721	763.708811	7.60E-04
MIR3145	1.45E-02	0.047265001	0.002952007	0.756766682	3.10E-02
MIR3143	2.33E-02	56.45935051	1.844577507	1728.123784	2.08E-02
MIR3135B	5.25E-05	18596.90774	16.01390605	21596540.92	6.33E-03

MIR302B	1.38E-02	72.92493611	1.598011808	3327.914274	2.78E-02
MIR29A	2.84E-04	313.4281197	9.941050119	9881.972733	1.10E-03
MIR2276	5.28E-04	20228.57215	9.3895937	43579641.9	1.13E-02
MIR219A1	1.07E-03	0.152326661	0.050242396	0.461829324	8.84E-04
MIR212	4.12E-04	65827.63634	12.22179309	354553352	1.14E-02
MIR19B2	2.76E-06	1432286.796	99.43014078	20632028188	3.71E-03
MIR199B	5.16E-03	189177.6257	4.212400734	8495909182	2.62E-02
MIR199A1	8.03E-03	2.631270242	1.317700798	5.25429073	6.11E-03
MIR1913	6.20E-04	24.30677586	3.494731294	169.0600229	1.26E-03
MIR191	1.99E-03	1520022.877	11.66651885	1.98043E+11	1.78E-02
MIR155HG	2.11E-02	3.740615241	1.953188874	7.163773338	6.91E-05
MIR146B	6.03E-04	202.6301811	3.691180124	11123.54014	9.35E-03
MIR130B	5.28E-04	9968.452964	8.00247482	12417415.45	1.13E-02
MIR1302-11	1.99E-03	29263412.68	19.43712727	4.40573E+13	1.78E-02
MIR1286	2.02E-02	161.6143713	3.602633743	7250.030638	8.78E-03
MIR1277	2.76E-06	6261612.512	160.4719614	2.44328E+11	3.71E-03
MINOS1-NBL1	3.52E-02	2.8447E+14	30158.42497	2.68E+24	4.51E-03
MINOS1	2.72E-02	3.173679543	1.28799599	7.820087887	1.21E-02
MIMT1	1.97E-02	1.35E-08	8.38E-16	0.216113663	3.23E-02
MILR1	9.48E-04	5.056061575	1.744195561	14.65647501	2.84E-03
MIEN1	3.15E-04	7.566102139	2.627861408	21.7842164	1.76E-04
MIEF2	6.99E-03	0.16993297	0.051120841	0.56488144	3.83E-03
MID1	1.75E-04	0.254219672	0.112808059	0.572899154	9.54E-04
MICALL2	2.70E-04	3.315540205	1.535699692	7.158174809	2.27E-03
MICALCL	1.15E-02	349486268.9	1.607760895	7.59694E+16	4.46E-02
MICAL1	4.41E-02	2.262047164	1.438282071	3.557617437	4.11E-04
MIB2	4.03E-06	0.223318781	0.107329991	0.464653705	6.07E-05
MGST3	4.75E-04	17.65171587	4.995776203	62.36930169	8.28E-06
MGST2	3.88E-05	3.684220755	1.957583268	6.933795764	5.30E-05
MGST1	4.99E-02	2.14863923	1.327058113	3.47886087	1.87E-03
MGRN1	3.21E-05	0.081251569	0.017080259	0.386517417	1.61E-03
MGLL	7.23E-04	1.862811105	1.443259818	2.404324689	1.77E-06
MGC39584	2.94E-02	1528.629633	1.565228047	1492886.968	3.68E-02

MGAT5B	1.77E-02	3.08538566	1.461827679	6.512125067	3.11E-03
MGAT5	1.03E-02	2.125647744	1.091581271	4.139296315	2.66E-02
MFSD9	2.20E-02	5.83019144	1.467325352	23.16536832	1.23E-02
MFSD10	3.83E-02	4.706574489	2.041564261	10.85042672	2.78E-04
MFNG	4.39E-04	3.116714468	1.528610508	6.354731323	1.76E-03
MFF	3.44E-04	7.722672222	2.356273281	25.3110141	7.38E-04
MFAP3L	1.47E-02	0.221901386	0.095526277	0.515462621	4.63E-04
MFAP2	3.38E-02	0.445579305	0.258997486	0.766574685	3.50E-03
MEX3D	4.88E-02	0.18294994	0.062864116	0.532429029	1.83E-03
METTL2B	4.37E-02	4.524810408	1.49260712	13.71687764	7.64E-03
METTL2A	5.22E-03	6.410005979	1.574973276	26.08817387	9.48E-03
METTL21B	4.70E-02	0.351286433	0.146691645	0.841235083	1.89E-02
METRNL	1.21E-02	5.926657916	1.998924629	17.57208528	1.33E-03
Metazoa_SRP	2.08E-03	0.080825291	0.020605061	0.317044805	3.09E-04
MET	1.27E-02	2.039205072	1.210504108	3.435227767	7.41E-03
MESDC1	3.36E-02	3.283391979	1.414041267	7.624008677	5.67E-03
MEP1A	2.10E-02	211.5181659	3.084111946	14506.58579	1.31E-02
MEMO1P3	1.99E-03	7.70566E+15	552.0075713	1.08E+29	1.78E-02
MELK	3.55E-02	3.129841437	1.270485949	7.71036266	1.31E-02
MEIS3	3.18E-02	2.859910764	1.390832961	5.880713075	4.28E-03
MEIS2	3.09E-02	1.81862552	1.030330778	3.210035897	3.91E-02
MEIS1	2.24E-02	0.161643653	0.031027883	0.842102913	3.05E-02
MEI1	2.21E-02	16.37501029	3.053589202	87.81173375	1.10E-03
MEGF10	1.54E-06	0.109641019	0.02661058	0.451743364	2.21E-03
MEF2C-AS1	2.52E-04	0.031133679	0.002490426	0.389212972	7.10E-03
MEF2BNB	3.47E-04	5.27559485	1.708415658	16.29105943	3.84E-03
MEDAG	2.09E-02	25.74123671	2.499654652	265.081125	6.33E-03
MED6	4.09E-02	6.974854036	1.5647339	31.09064668	1.09E-02
MED31	1.21E-02	4.261350335	1.443287181	12.58176953	8.68E-03
MED29	2.94E-03	6.876556192	2.0654735	22.8940362	1.68E-03
MED22	5.34E-04	0.136611551	0.041647521	0.448111088	1.02E-03
MED18	2.53E-02	3.549092787	1.193937574	10.55001524	2.27E-02
MED15	9.51E-03	2.546390726	1.154253013	5.617577479	2.06E-02

MED11	4.49E-04	8.532210446	2.566418653	28.36583774	4.69E-04
MECOM	1.91E-02	179.8405494	5.038111003	6419.593217	4.42E-03
ME3	9.68E-03	0.3080018	0.139714068	0.678994679	3.50E-03
ME2	2.37E-02	3.064675166	1.490964972	6.299432951	2.32E-03
ME1	1.16E-04	1.806568577	1.259836699	2.590565925	1.30E-03
MDP1	1.77E-04	15.21537678	3.358564074	68.93055646	4.13E-04
MDK	2.06E-04	1.486874635	1.091088345	2.026230223	1.20E-02
MDH1	2.65E-03	2.721430818	1.318320636	5.617894079	6.78E-03
MDGA1	1.92E-03	2.630881753	1.421979125	4.867538964	2.06E-03
MDC1	2.25E-02	0.275074249	0.109830411	0.688933437	5.86E-03
MCU	2.14E-03	6.168544752	2.354164054	16.16325093	2.14E-04
MCTS1	3.44E-04	9.687156403	2.069375361	45.3475	3.93E-03
MCOLN2	3.31E-02	3.665158168	1.490001548	9.015684859	4.68E-03
MCM6	3.35E-03	2.683394642	1.197521674	6.012923988	1.65E-02
MCM4	2.76E-04	2.488575396	1.46687525	4.221904692	7.23E-04
MCM2	3.11E-05	0.139807754	0.056475976	0.346097747	2.10E-05
MCIDAS	2.85E-02	4.792412297	1.677475532	13.69153539	3.44E-03
MCF2	4.46E-03	0.535434452	0.301284251	0.951560032	3.32E-02
MCCC2	3.44E-05	5.830071939	1.940401833	17.51685565	1.68E-03
MC1R	1.16E-03	3.469880154	1.622983575	7.418478209	1.33E-03
MBP	2.32E-03	2.725494203	1.374116707	5.405886278	4.11E-03
MBOAT2	5.79E-04	2.529407844	1.285906681	4.975403066	7.18E-03
MBNL1-AS1	2.19E-02	4.47E-07	1.32E-11	0.015161282	6.01E-03
MBD5	3.07E-03	6.621617929	1.790918948	24.48230504	4.61E-03
MB21D1	2.04E-02	6.910363828	2.537073548	18.82213004	1.56E-04
MATN2	7.46E-03	0.373198753	0.216949406	0.641980597	3.69E-04
MATK	2.67E-04	2.153011418	1.540109869	3.009823039	7.24E-06
MAST4-AS1	4.00E-03	0.004092078	4.74E-05	0.35344192	1.56E-02
MAST2	2.84E-02	2.982640911	1.424172239	6.246538555	3.76E-03
MARVELD2	1.77E-02	0.1390052	0.025816535	0.748452325	2.16E-02
MARK2P17	1.39E-02	1896020115	9.52743413	3.7732E+17	2.84E-02
MARK1	2.66E-02	2.41106719	1.230930609	4.72264233	1.03E-02
MARCKS	5.50E-03	0.355101627	0.219180808	0.575311161	2.60E-05

9-Mar	3.20E-04	0.183647746	0.079535294	0.424044379	7.21E-05
8-Mar	9.63E-03	0.093447605	0.02749214	0.317634604	1.46E-04
2-Mar	2.12E-04	0.37960051	0.21286575	0.676936272	1.03E-03
MAPT	2.91E-03	1.579753234	1.005515873	2.481930268	4.73E-02
MAPKAPK3	1.37E-04	0.156524677	0.056640435	0.432552723	3.49E-04
MAPK8IP2	2.41E-02	0.608473049	0.3962055	0.93446318	2.32E-02
MAPK12	6.22E-07	3.702009252	2.026682403	6.762220109	2.06E-05
MAPK1	9.03E-03	2.895607401	1.163501743	7.206299663	2.23E-02
MAP4	6.27E-04	0.162432977	0.0609421	0.432943268	2.79E-04
MAP3K3	8.32E-03	0.227894235	0.081632866	0.636211674	4.75E-03
MAP3K14-AS1	2.90E-02	16.60927706	1.849968634	149.1204118	1.21E-02
MAP3K13	1.64E-04	0.07540929	0.018918835	0.300576703	2.49E-04
MAP3K12	1.44E-04	2.68333032	1.585022241	4.542688058	2.38E-04
MAP3K11	4.48E-03	0.387107747	0.18126014	0.826725655	1.42E-02
MAP3K10	1.39E-02	3.529859372	1.18465624	10.51774073	2.36E-02
MAP2K5	3.25E-04	0.383501792	0.186276445	0.789544941	9.29E-03
MAP2K3	3.61E-05	3.311033053	1.35148454	8.111776015	8.82E-03
MAP2	6.08E-03	1.647985664	1.164925849	2.3313559	4.77E-03
MAP1LC3C	1.31E-02	0.459748503	0.271733125	0.777853955	3.78E-03
MAP1A	2.11E-05	1.492376162	1.128916249	1.972853708	4.93E-03
MANSC1	3.10E-04	0.294251716	0.141439045	0.612165279	1.06E-03
MANF	4.46E-02	2.676114077	1.027236669	6.971700646	4.39E-02
MANEAL	2.13E-08	0.123166083	0.044418203	0.341524039	5.71E-05
MANBA	2.09E-02	3.201160341	1.520270415	6.740529465	2.19E-03
MAN2A1	9.75E-04	5.480716598	2.329242929	12.89614494	9.75E-05
MAN1C1	8.94E-04	1.976368504	1.220401939	3.200611487	5.61E-03
MAN1B1-AS1	2.51E-06	0.121446129	0.038229538	0.385805406	3.50E-04
MAMSTR	2.90E-05	0.223978307	0.095591903	0.52479635	5.73E-04
MALSU1	4.05E-05	14.04697223	3.921301211	50.31937567	4.93E-05
MALL	2.67E-03	3.384535142	1.518243398	7.544955007	2.87E-03
MAL2	9.75E-03	1.470627387	1.17540565	1.839998737	7.42E-04
MAGIX	5.41E-04	28.51477849	3.500219814	232.2975799	1.74E-03
MAGI2-AS2	3.82E-09	4.82E+27	5085902191	4.57E+45	2.54E-03

MAGEE1	1.08E-02	0.25919442	0.074097441	0.90666758	3.46E-02
MAGEB6	3.82E-09	2.40E+111	1.13E+39	5.09E+183	2.54E-03
MAGEA10	7.23E-05	3.29E+123	2.02E+34	5.35E+212	6.65E-03
MAGEA1	4.12E-02	5.64631E+13	1.630609346	1.96E+27	4.65E-02
MAFB	7.16E-04	2.347958028	1.459717016	3.776695647	4.32E-04
MAFA	2.09E-02	97.23305451	3.374677142	2801.532263	7.60E-03
MAF1	3.85E-02	2.426687726	1.109976129	5.30535132	2.63E-02
MAD1L1	3.22E-02	2.872664784	1.589928308	5.19029878	4.72E-04
MACC1	5.60E-03	1711.838651	1.130722078	2591610.815	4.63E-02
MAATS1	3.32E-02	0.009955616	0.000137348	0.721627249	3.49E-02
LZTS2	1.35E-02	0.241029232	0.063150184	0.919951254	3.73E-02
LZTFL1	4.52E-03	0.186282834	0.069218174	0.501332126	8.78E-04
LYZ	9.43E-04	1.43310598	1.161542329	1.768160057	7.88E-04
LYRM4	1.14E-04	0.212163172	0.093991084	0.478909378	1.90E-04
LYPLAL1-AS1	4.96E-02	0.029397077	0.002407382	0.358974302	5.74E-03
LYPD8	6.67E-03	0.416624801	0.206831433	0.839215889	1.43E-02
LYPD5	2.18E-03	0.11783202	0.026017693	0.533651667	5.52E-03
LYN	2.99E-04	2.993490503	1.766010149	5.074141502	4.66E-05
LYL1	6.99E-03	4.507155087	1.508167367	13.46962374	7.03E-03
LY96	2.91E-02	1.395661556	1.138281658	1.711238308	1.35E-03
LY9	4.20E-06	37.47594284	5.98307201	234.7366519	1.08E-04
LY86-AS1	4.13E-04	1.42E-68	2.20E-126	9.13E-11	2.14E-02
LY6G5C	7.44E-03	0.236906638	0.090248668	0.621890119	3.45E-03
LY6D	9.60E-04	102.9803354	1.802443239	5883.652401	2.47E-02
LURAP1L	2.94E-02	2.449974962	1.090452373	5.504483701	3.00E-02
LURAP1	1.96E-04	0.299191819	0.172456573	0.519062527	1.76E-05
LTBR	7.87E-08	6.738031986	2.873860442	15.79794008	1.14E-05
LTBP1	5.34E-03	0.393118985	0.209453725	0.73783618	3.66E-03
LTB4R	1.17E-05	0.128235342	0.035987285	0.456947583	1.54E-03
LTB	2.61E-04	1.758741407	1.259228759	2.456401441	9.26E-04
LTA4H	1.78E-05	0.194630703	0.090294627	0.419527845	2.96E-05
LTA	5.08E-03	6.283977511	2.016407876	19.58352465	1.53E-03
LST1	3.62E-04	2.023369159	1.303289854	3.141298724	1.69E-03

LSM4	6.34E-03	22.31664455	3.735845959	133.3118735	6.61E-04
LSM3	8.18E-05	0.075033699	0.020078265	0.2804055	1.18E-04
LSM2	1.59E-02	0.25341163	0.103225315	0.622109549	2.74E-03
LSM12	1.76E-02	5.221279166	1.434642538	19.00247302	1.22E-02
LSG1	4.87E-02	0.37196275	0.153590079	0.900815263	2.84E-02
LRWD1	1.41E-03	9.422998452	2.131726508	41.65304483	3.09E-03
LRRTM2	5.14E-06	2.697943938	1.00875506	7.215727368	4.80E-02
LRRN3	4.97E-03	2.647504893	1.314455757	5.332459552	6.42E-03
LRRN1	4.03E-03	0.330691357	0.125468666	0.871586325	2.52E-02
LRRK2	2.27E-02	0.076942629	0.012367216	0.478698529	5.96E-03
LRRIQ4	2.96E-02	5.93E-11	4.85E-19	0.007244395	1.32E-02
LRRIQ1	1.48E-02	9.45E-07	2.22E-12	0.401819051	3.59E-02
LRRC75A-AS1	3.49E-03	0.458034516	0.251727475	0.833423597	1.06E-02
LRRC75A	4.56E-04	8.668075865	3.382094346	22.21568398	6.88E-06
LRRC66	3.24E-02	310.7928101	1.276534219	75667.51392	4.07E-02
LRRC59	4.54E-04	5.299235558	1.956367034	14.35410483	1.04E-03
LRRC56	2.35E-03	0.384171337	0.221534339	0.666206498	6.59E-04
LRRC48	5.25E-06	0.013925643	0.001515008	0.128001656	1.59E-04
LRRC46	4.97E-04	0.024158933	0.002692735	0.216751379	8.82E-04
LRRC4	5.50E-04	2.286493776	1.133490889	4.61234743	2.09E-02
LRRC3C	2.33E-03	9.29E-08	2.67E-13	0.032279054	1.29E-02
LRRC39	4.76E-02	1.764845027	1.146470726	2.71675316	9.85E-03
LRRC37A5P	2.63E-02	0.050850536	0.006305178	0.410103715	5.16E-03
LRRC37A4P	1.84E-05	60.63742824	10.29276739	357.2312056	5.72E-06
LRRC27	1.09E-02	0.047181331	0.011870069	0.187537076	1.44E-05
LRRC25	1.02E-02	2.461892624	1.283860097	4.720853389	6.68E-03
LRRC23	4.27E-02	0.272030537	0.098794183	0.749038165	1.18E-02
LRRC20	1.21E-03	3.752523414	1.786406648	7.882545659	4.79E-04
LRR1	3.65E-04	8.430251587	2.870574297	24.75781305	1.05E-04
LRP8	3.04E-03	2.861750763	1.414606991	5.789323455	3.45E-03
LRP12	1.21E-02	3.033908473	1.657149457	5.554478255	3.22E-04
LRIG3	9.60E-04	0.136031162	0.033756073	0.548182155	5.03E-03
LRFN4	1.53E-04	3.956193525	1.919972501	8.151922593	1.93E-04

LRCOL1	4.75E-02	8.427075607	1.596925373	44.47020788	1.20E-02
LRCH4	2.31E-03	0.152770077	0.040391951	0.577805616	5.64E-03
LRCH3	1.41E-03	0.105432569	0.029327417	0.379031892	5.69E-04
LPP-AS1	5.87E-03	597983040.9	354.1057511	1.00982E+15	5.74E-03
LPO	1.28E-02	1.43E-18	1.53E-31	1.34E-05	7.01E-03
LPIN1	1.11E-02	0.353057423	0.17927503	0.695297858	2.60E-03
LPHN1	5.24E-03	2.566808737	1.147472471	5.741756125	2.17E-02
LPAR1	5.28E-04	12.52926883	3.715855365	42.24668669	4.57E-05
LOXL4	6.26E-03	1.590872987	1.149417056	2.201878639	5.12E-03
LOXL3	3.15E-02	3.222108506	1.329812453	7.80710333	9.56E-03
LOXL1-AS1	3.29E-02	0.206188325	0.05865671	0.724787085	1.38E-02
LONRF3	2.30E-02	33.62270307	3.519287396	321.2258717	2.27E-03
LOC440461	2.39E-02	189272672.1	1514.44152	2.3655E+13	1.46E-03
LOC401913	6.54E-05	1.10E-05	7.99E-09	0.015203649	1.97E-03
LNP1	6.55E-07	0.527890246	0.378042211	0.737134911	1.77E-04
LMO4	1.14E-02	0.560592899	0.316147131	0.994044757	4.77E-02
LMO3	2.73E-03	0.034981621	0.002837825	0.431215308	8.89E-03
LMCD1-AS1	3.14E-03	2.14E-08	6.27E-14	0.007297862	6.59E-03
LMCD1	1.96E-08	0.097639	0.033671022	0.283132907	1.84E-05
LMAN2L	1.35E-02	0.046968785	0.009112504	0.242092255	2.57E-04
LMAN2	9.03E-03	7.02219312	1.856486844	26.56156513	4.09E-03
LMAN1	2.31E-02	2.059978163	1.212797626	3.498943221	7.50E-03
LLNLR-285B5.1	1.17E-02	0.000492969	1.49E-06	0.162624717	1.01E-02
LLNLF-158E9.1	1.99E-03	7.64E+137	6.27E+23	9.30E+251	1.78E-02
LL22NC03-88E1.18	5.25E-04	65.2029279	7.330701268	579.9474909	1.79E-04
LL22NC03-80A10.6	1.20E-02	5.798361258	1.935858564	17.36748433	1.69E-03
LL22NC03-30E12.13	2.76E-06	37911960871	2707.805994	5.30805E+17	3.71E-03
LL22NC03-24A12.9	1.50E-02	888629.7242	4.388083462	1.79956E+11	2.80E-02
LL22NC03-23C6.15	2.76E-06	8689575309	1678.887118	4.49755E+16	3.71E-03
LL22NC03-104C7.1	1.38E-05	0.031316842	0.00351355	0.27913211	1.91E-03
LL21NC02-21A1.1	2.57E-03	2.960694976	1.414570165	6.196733791	3.97E-03
LL0XNC01-138C3.2	1.99E-03	1016628173	35.85659923	2.88241E+16	1.78E-02
LL0XNC01-116E7.2	1.58E-02	7709.095821	19.06587615	3117095.585	3.47E-03

LL09NC01-251B2.3	4.70E-04	0.011588558	0.00081766	0.164242658	9.83E-04
LKAAEAR1	1.71E-02	6.355787741	2.009723318	20.10029811	1.64E-03
LIX1L	1.29E-03	3.014861569	1.367966511	6.6444538	6.20E-03
LIPE	3.49E-03	0.049293038	0.010849165	0.223962272	9.72E-05
LIPA	7.90E-06	2.648771591	1.511116254	4.64291938	6.70E-04
LINGO4	2.75E-02	5.27E-15	1.36E-26	0.002040381	1.57E-02
LINGO2	5.21E-05	0.006153198	0.0003268	0.115856483	6.76E-04
LINGO1	3.71E-05	2.418808689	1.532094831	3.818716282	1.50E-04
LINC01583	2.30E-03	8.95E+45	9.58805E+18	8.35E+72	8.40E-04
LINC01569	1.73E-02	4.54487179	1.285630201	16.06671931	1.88E-02
LINC01567	3.17E-02	1.51E-25	1.02E-47	0.002254531	2.82E-02
LINC01562	3.77E-02	2.99E-07	3.22E-13	0.278163525	3.22E-02
LINC01540	4.79E-03	2.239287744	1.228943751	4.080259651	8.45E-03
LINC01521	2.47E-02	0.105990454	0.023057561	0.487214425	3.93E-03
LINC01518	1.07E-02	820669905.3	25.7040629	2.6202E+16	1.99E-02
LINC01486	6.03E-03	0.007547124	0.000229309	0.248393941	6.12E-03
LINC01448	7.23E-05	1.49E+50	8.61181E+13	2.57E+86	6.65E-03
LINC01431	4.72E-02	0.036129269	0.003049767	0.428007874	8.47E-03
LINC01430	1.88E-02	8.19525E+15	1.231039006	5.46E+31	4.87E-02
LINC01392	7.23E-05	2.82E+24	6176627.454	1.28E+42	6.65E-03
LINC01385	7.23E-05	3.77E+33	2116100318	6.71E+57	6.65E-03
LINC01375	2.76E-06	4.14E+108	1.75E+35	9.75E+181	3.71E-03
LINC01358	1.28E-05	75.7551401	8.684339903	660.8264203	9.01E-05
LINC01356	7.66E-03	0.115765157	0.026601506	0.503789965	4.06E-03
LINC01354	1.38E-03	2.64E-08	9.94E-14	0.007029873	6.18E-03
LINC01282	5.84E-03	5.93E+24	33394.50793	1.05E+45	1.65E-02
LINC01281	1.82E-03	49044.6463	5.23835606	459185535.2	2.06E-02
LINC01278	1.19E-04	0.141371818	0.055152514	0.362376788	4.63E-05
LINC01272	6.83E-04	2.613324798	1.659787572	4.114662996	3.36E-05
LINC01271	1.04E-05	244.5338582	10.03933919	5956.249378	7.36E-04
LINC01270	2.29E-06	7.70170167	2.843609709	20.85947605	5.93E-05
LINC01267	1.56E-04	1.11E-43	5.51E-73	2.23E-14	4.06E-03
LINC01264	1.99E-03	1.64967E+13	191.0840547	1.42E+24	1.78E-02

LINC01255	1.88E-02	3.85E+75	2.683924607	5.53E+150	4.87E-02
LINC01252	1.20E-03	12.16753171	3.873510213	38.22084355	1.88E-05
LINC01234	1.50E-02	0.213233247	0.05252648	0.865628483	3.06E-02
LINC01233	3.81E-03	4.03E-09	6.42E-16	0.025260985	1.55E-02
LINC01224	1.80E-02	1.09E-30	4.52E-55	2.63E-06	1.60E-02
LINC01220	4.93E-03	4.579184709	1.567641532	13.37610172	5.40E-03
LINC01214	1.06E-02	12554318276	4.338143995	3.63314E+19	3.64E-02
LINC01194	5.28E-04	1.92E+82	3.8604E+18	9.59E+145	1.13E-02
LINC01193	1.47E-04	6917.865797	1.386824773	34508229.25	4.18E-02
LINC01189	5.16E-03	1.02E+149	4.32228E+17	2.43E+280	2.62E-02
LINC01181	3.29E-02	5850041597	5278.457524	6.48352E+15	1.54E-03
LINC01152	3.96E-02	0.19215838	0.047929921	0.77039232	1.99E-02
LINC01102	5.41E-03	1.95E-05	1.44E-09	0.262209824	2.54E-02
LINC01096	8.07E-05	3547484445	50727.10547	2.48085E+14	1.12E-04
LINC01065	1.06E-02	1.42912E+17	12.09494795	1.69E+33	3.64E-02
LINC01057	1.50E-06	0.085069172	0.026176662	0.276458627	4.17E-05
LINC01050	2.57E-03	7406.44894	65.74542817	834361.9843	2.19E-04
LINC01044	1.10E-03	817.8453603	23.1036999	28950.81897	2.28E-04
LINC01043	8.34E-05	1.70E+61	1.69E+33	1.71E+89	1.82E-05
LINC01023	3.47E-03	0.327418348	0.155570865	0.689092875	3.27E-03
LINC01010	2.44E-02	8.04891729	1.264395872	51.2379635	2.72E-02
LINC01006	1.29E-02	0.071823061	0.021419771	0.240831336	1.99E-05
LINC00998	2.94E-02	2.738909023	1.225391083	6.121819181	1.41E-02
LINC00972	1.99E-03	1.30E+42	18563434.31	9.13E+76	1.78E-02
LINC00968	6.85E-05	45.00426174	4.08074965	496.3263489	1.88E-03
LINC00963	1.14E-06	3.171856059	1.862893235	5.400562238	2.13E-05
LINC00959	6.57E-03	51.19538955	4.530453588	578.5221856	1.47E-03
LINC00950	1.71E-05	0.000698158	9.29E-06	0.052487297	9.77E-04
LINC00945	4.62E-05	187441.4691	14.94330068	2351174287	1.17E-02
LINC00942	8.79E-05	2711902.531	595.8317889	12343106682	5.67E-04
LINC00941	1.72E-03	2973.540706	37.72397507	234385.2766	3.32E-04
LINC00935	1.88E-02	7.65E-16	4.83E-30	0.121182739	3.69E-02
LINC00930	9.53E-03	3.318410743	1.496351458	7.359133311	3.16E-03

LINC00929	3.82E-04	3.26E+56	2.35E+21	4.51E+91	1.62E-03
LINC00921	3.56E-04	0.041733328	0.005916502	0.294375048	1.44E-03
LINC00919	1.96E-03	0.005173421	5.02E-05	0.533511811	2.60E-02
LINC00900	9.16E-06	9.925374091	3.266966862	30.1542853	5.17E-05
LINC00898	2.42E-02	3.11E+107	2474275.237	3.90E+208	3.72E-02
LINC00888	1.55E-04	0.279769681	0.116434179	0.672234523	4.40E-03
LINC00884	1.18E-03	0.000135992	5.61E-07	0.032980167	1.48E-03
LINC00882	4.31E-02	0.009334075	0.000131739	0.661346801	3.15E-02
LINC00881	4.19E-03	1.45E-10	8.10E-19	0.026043174	1.95E-02
LINC00869	5.29E-05	65.36347941	6.212381695	687.7208535	4.99E-04
LINC00866	4.50E-02	0.002791984	2.67E-05	0.29242692	1.32E-02
LINC00862	1.25E-02	10.28289142	1.296399801	81.56269062	2.74E-02
LINC00861	4.85E-02	12.8757073	1.659970707	99.87154468	1.45E-02
LINC00824	2.43E-02	1.06E+66	2.43848E+12	4.63E+119	1.58E-02
LINC00706	5.28E-04	1.01E+31	10088548.16	1.02E+55	1.13E-02
LINC00698	4.72E-02	2.33E+24	27602.67489	1.96E+44	1.65E-02
LINC00689	2.79E-03	0.159754384	0.049447006	0.516137687	2.17E-03
LINC00668	1.89E-02	1095.239597	3.782928965	317095.506	1.55E-02
LINC00665	3.73E-05	0.147355498	0.051368531	0.422703204	3.69E-04
LINC00664	4.89E-02	1.67E-14	8.24E-27	0.033985004	2.82E-02
LINC00638	2.77E-04	0.047552005	0.006745063	0.33523679	2.24E-03
LINC00634	8.11E-04	0.007136591	0.000261757	0.194573074	3.38E-03
LINC00622	1.53E-02	2.274957371	1.151400584	4.494900481	1.80E-02
LINC00613	2.76E-06	1.82E+32	29349020548	1.13E+54	3.71E-03
LINC00608	7.79E-03	4.58E+25	2286.089667	9.17E+47	2.41E-02
LINC00557	2.76E-06	7.16E+74	1.95E+24	2.64E+125	3.71E-03
LINC00547	1.65E-03	2.82E+127	1.36317E+17	5.82E+237	2.35E-02
LINC00544	5.88E-03	1.23152E+11	203.7024167	7.44533E+19	1.33E-02
LINC00528	4.53E-02	153.6408118	9.177444706	2572.121086	4.62E-04
LINC00518	9.50E-06	0.439960511	0.298074702	0.649385035	3.58E-05
LINC00505	2.74E-03	5.63745994	1.82129697	17.44962798	2.70E-03
LINC00494	2.19E-02	6.36302317	2.173077322	18.63167199	7.36E-04
LINC00493	1.48E-03	0.266298287	0.078519409	0.903149669	3.37E-02

LINC00486	8.22E-05	1.88E-05	8.12E-09	0.043392586	5.89E-03
LINC00475	4.71E-02	25898.61943	40.31610981	16636984.36	2.07E-03
LINC00472	6.36E-03	0.043258919	0.003322917	0.563160037	1.65E-02
LINC00456	3.77E-02	5.47E-07	5.36E-12	0.055753031	1.43E-02
LINC00454	9.42E-03	1.45E+64	51203974457	4.13E+117	1.86E-02
LINC00421	1.39E-03	3.968270103	1.061532081	14.83437749	4.05E-02
LINC00412	2.80E-02	0.047240988	0.003022568	0.738349202	2.95E-02
LINC00411	1.06E-02	5.92815E+13	7.39889416	4.75E+26	3.64E-02
LINC00404	4.03E-04	2.361980061	1.630609407	3.421389441	5.46E-06
LINC00403	6.71E-05	2.170223161	1.537699438	3.06293184	1.04E-05
LINC00375	2.76E-06	2.86E+35	3.19683E+11	2.55E+59	3.71E-03
LINC00365	1.77E-03	4.72E-12	6.08E-20	0.000366774	4.90E-03
LINC00354	8.44E-04	6.148359538	1.810300365	20.88179716	3.60E-03
LINC00346	2.30E-05	2.704763153	1.360438462	5.377489622	4.54E-03
LINC00322	1.68E-03	0.548482238	0.306430272	0.981733181	4.32E-02
LINC00284	3.86E-03	3.88E+31	2.58536E+12	5.83E+50	1.24E-03
LINC00269	6.95E-03	8.37E+25	516.6689494	1.35E+49	2.86E-02
LINC00264	3.53E-05	25460597.56	241.4846432	2.6844E+12	3.86E-03
LINC00240	3.48E-04	7.06E-07	1.52E-10	0.003275295	1.01E-03
LINC00239	8.19E-04	159.293515	3.557873843	7131.906594	8.94E-03
LINC00158	3.63E-02	7557.752731	54.48252929	1048402.618	3.87E-04
LINC00152	8.57E-06	2.861568556	1.791223338	4.571498386	1.09E-05
LINC00094	7.91E-03	0.180161761	0.055593109	0.583854017	4.28E-03
LINC00087	3.07E-07	0.111001531	0.036198089	0.340386475	1.21E-04
LINC00086	5.38E-07	0.102921212	0.036039835	0.293918551	2.17E-05
LIMS2	1.72E-05	0.395037334	0.254192926	0.613921473	3.65E-05
LIMD1-AS1	3.66E-02	4.12E-06	8.38E-11	0.202662753	2.45E-02
LILRB5	2.65E-03	2.370354359	1.299735321	4.322864583	4.88E-03
LILRB3	2.01E-02	11.74844402	2.069597862	66.69215284	5.42E-03
LILRB2	9.22E-04	2.561023728	1.330899587	4.928127259	4.86E-03
LILRA6	1.44E-02	5.574119723	1.170739921	26.5394646	3.09E-02
LILRA5	7.34E-03	5.139350292	1.710078049	15.4454479	3.55E-03
LIG3	9.79E-03	3.224826171	1.235066105	8.420200173	1.68E-02

LIF	2.00E-03	3.820825767	1.590186	9.180504385	2.73E-03
LHFPL3-AS1	1.34E-03	2.255948704	1.411225769	3.606300754	6.76E-04
LHFPL3	1.28E-03	2.062160971	1.368384951	3.107683891	5.43E-04
LHFPL2	4.82E-05	4.152049407	1.871808734	9.210083254	4.61E-04
LHFP	6.44E-06	2.439071857	1.607967667	3.69974574	2.74E-05
LHB	6.44E-06	4.254682472	2.007769216	9.016137311	1.57E-04
LGR6	1.08E-02	21.76174322	4.049910154	116.9343146	3.30E-04
LGMNP1	1.08E-03	21.25752039	3.450186918	130.973244	9.85E-04
LGMN	2.32E-02	2.323946888	1.288039328	4.192984658	5.10E-03
LGI4	2.68E-03	0.3481051	0.18450333	0.656774923	1.12E-03
LGALS9	7.13E-03	1.672720014	1.125257302	2.486535514	1.10E-02
LGALS7B	1.66E-02	727.5557793	8.297841787	63792.17941	3.89E-03
LGALS3BP	3.21E-02	2.090051325	1.152094986	3.791627075	1.53E-02
LGALS17A	1.51E-02	1.99599769	1.228682396	3.242503344	5.24E-03
LGALS1	8.04E-05	2.763341637	1.646532703	4.637658875	1.19E-04
LFNG	1.20E-04	2.706384862	1.663465202	4.403169366	6.09E-05
LETMD1	3.98E-03	0.301469664	0.141567226	0.641984458	1.88E-03
LETM1	1.38E-03	4.882451046	1.994234527	11.95362326	5.19E-04
LEO1	4.46E-02	2.490647194	1.210699992	5.123749473	1.32E-02
LEKR1	3.58E-05	6.25E-05	7.93E-08	0.049261588	4.45E-03
LEF1	3.87E-05	3.803227978	1.512676272	9.562219834	4.51E-03
LDLRAD3	8.47E-04	3.009267606	1.761110702	5.142034236	5.57E-05
LDB2	3.70E-02	2.786577812	1.26116546	6.157015991	1.13E-02
LCTL	3.65E-06	160.160751	8.307185672	3087.864794	7.73E-04
LCP2	9.66E-05	2.012864392	1.282894493	3.158188833	2.34E-03
LCP1	3.77E-05	1.678194793	1.236095329	2.278414697	9.05E-04
LCN1P1	3.19E-02	4466625914	1517.905939	1.31436E+16	3.46E-03
LCN1	2.72E-02	276583558.4	714.6332657	1.07046E+14	3.07E-03
LCMT2	1.17E-02	3.591343549	1.080744725	11.9341304	3.69E-02
LCMT1-AS1	8.82E-04	0.003738495	0.00015251	0.09164239	6.17E-04
LCMT1	1.00E-03	0.067797926	0.016674709	0.275660502	1.70E-04
LCK	2.51E-05	1.818813014	1.270896248	2.602951095	1.07E-03
LCE2A	4.80E-03	2.275704002	1.087022836	4.764231747	2.92E-02

LBX1-AS1	3.59E-04	1082459356	80187.30179	1.46123E+13	1.81E-05
LBP	6.37E-03	39038.90288	2.606009808	584815887.4	3.11E-02
LAYN	1.79E-03	4.605593066	1.988789454	10.66552693	3.64E-04
LAX1	2.11E-03	6.94809925	2.174674813	22.19921935	1.07E-03
LAT2	1.32E-02	3.04182124	1.341474703	6.89739168	7.74E-03
LARS2	2.46E-03	0.077344703	0.021857955	0.2736854	7.20E-05
LARS	4.97E-02	2.695932609	1.061588822	6.846391447	3.70E-02
LARP1	2.92E-03	4.748958808	1.235140241	18.25914905	2.34E-02
LARGE	2.56E-02	0.270090957	0.106758388	0.683310475	5.71E-03
LAPTM5	4.15E-05	1.63131277	1.220683972	2.180073972	9.40E-04
LAPTM4B	2.46E-02	2.074304159	1.275714223	3.37280691	3.26E-03
LAMTOR4	3.04E-02	11.40348303	2.394868431	54.29919389	2.24E-03
LAMTOR2	6.77E-06	12.06537071	4.30425976	33.82072144	2.19E-06
LAMC1	2.29E-04	2.794166238	1.583840263	4.92938912	3.89E-04
LAMB2	1.04E-04	0.289798519	0.148388913	0.565966688	2.87E-04
LAMA5	3.86E-03	6.484377191	2.574118263	16.33458266	7.32E-05
LAMA4	4.41E-03	0.257729926	0.107765492	0.61638205	2.31E-03
LAMA1	1.97E-05	4.106893752	2.226186573	7.576443276	6.14E-06
LAIR1	1.02E-02	2.212830007	1.181543756	4.144253322	1.31E-02
LAG3	4.77E-05	1.496242268	1.182389577	1.893403805	7.94E-04
LA16c-OS12.2	3.66E-03	0.202174186	0.071556587	0.571217876	2.56E-03
LA16c-60G3.7	7.68E-05	1.10E+39	2.10606E+15	5.76E+62	1.25E-03
LA16c-349E10.1	3.27E-02	34.5251964	4.078364499	292.2713718	1.15E-03
LA16c-313D11.9	9.72E-03	0.024194212	0.002110824	0.277313418	2.78E-03
L3MBTL4-AS1	3.99E-04	7.142724965	3.241650868	15.7384376	1.07E-06
KTN1-AS1	5.52E-03	0.120851541	0.026562778	0.54983311	6.26E-03
KRTCAP2	1.63E-07	10.81732397	4.605351828	25.40837321	4.62E-08
KRTAP5-AS1	2.16E-02	0.30901276	0.111408448	0.857106329	2.41E-02
KRTAP5-1	4.76E-03	0.153673067	0.03932467	0.600524083	7.08E-03
KRTAP4-2	5.28E-04	6.41E+33	43287076.31	9.48E+59	1.13E-02
KRTAP19-6	5.28E-04	7.2445E+14	2273.169273	2.31E+26	1.13E-02
KRTAP19-4	5.16E-03	1.71448E+14	48.37736534	6.08E+26	2.62E-02
KRTAP10-6	5.28E-04	1.71E+67	1.53796E+15	1.91E+119	1.13E-02

KRTAP1-1	5.28E-04	19821153001	211.8128212	1.85484E+18	1.13E-02
KRT8P5	2.77E-02	1.23E+21	277.0458673	5.47E+39	2.66E-02
KRT8P12	4.09E-02	0.442121531	0.209957617	0.931004319	3.17E-02
KRT42P	1.35E-03	2.14E-54	4.18E-107	0.109675898	4.60E-02
KRT38	3.43E-03	1.17E+40	14123048358	9.74E+69	8.67E-03
KRT36	4.54E-02	7.795154296	1.344601085	45.1914186	2.20E-02
KRT19P1	3.46E-02	5779728.244	47.94101917	6.96799E+11	9.10E-03
KRT18P54	5.16E-03	6.93E+33	10119.35437	4.75E+63	2.62E-02
KRT18P42	3.56E-02	1.45E+28	20365.55451	1.03E+52	2.07E-02
KRT18P36	8.41E-07	3.58E+61	6.49E+26	1.98E+96	5.15E-04
KRT18P34	1.70E-02	0.000218276	4.81E-07	0.099023022	6.92E-03
KRT16P6	3.70E-02	2.238246942	1.375727832	3.641526513	1.18E-03
KRT16P5	1.06E-02	3.56035E+15	9.580919416	1.32E+30	3.64E-02
KRT16P1	1.28E-03	2.53E+26	4031917017	1.59E+43	2.06E-03
KRT12	1.92E-03	1.31E+24	2.49069E+12	6.88E+35	5.51E-05
KRT10	2.01E-02	4.13843183	1.603032872	10.68388448	3.33E-03
KREMEN2	1.76E-04	0.193357559	0.067361713	0.555020711	2.26E-03
KRBA1	1.42E-05	0.212233786	0.081291279	0.554096092	1.55E-03
KPTN	5.13E-05	10.93191833	3.844908052	31.08184559	7.26E-06
KPNB1	1.28E-02	3.47945083	1.294711346	9.350793226	1.34E-02
KPNA2	1.45E-03	4.179478538	1.901602923	9.185956035	3.71E-04
KMT2B	2.96E-03	0.088584826	0.018561125	0.422779951	2.37E-03
KMO	1.52E-04	64.38495384	1.687851241	2456.035331	2.50E-02
KLK15	4.98E-03	1.42E-25	3.42E-47	0.000589191	2.43E-02
KLK13	2.90E-02	0.284623245	0.105131795	0.77056034	1.34E-02
KLK1	3.69E-02	116.7221285	4.173303796	3264.573093	5.10E-03
KLHL38	3.30E-03	2.000384266	1.342257211	2.981200013	6.59E-04
KLHL35	4.64E-02	0.226888352	0.07686267	0.669744162	7.24E-03
KLHL34	1.22E-03	3.02E-07	1.01E-11	0.009039053	4.31E-03
KLHL30	4.02E-04	2.861939903	1.541106775	5.314816691	8.70E-04
KLHL2P1	5.19E-03	0.002326076	4.44E-05	0.121792351	2.68E-03
KLHL26	6.51E-03	0.316601348	0.11914864	0.841271992	2.11E-02
KLHL25	4.20E-02	5.486497097	1.000915995	30.07410268	4.99E-02

KLHL21	3.04E-04	0.275553523	0.135927361	0.558605297	3.50E-04
KLHDC3	2.68E-02	0.323925087	0.146449337	0.716476181	5.38E-03
KLF4	3.18E-04	1.664199328	1.122298497	2.467756492	1.13E-02
KLF2	4.81E-02	1.664443486	1.001341929	2.766659458	4.94E-02
KLF15	2.27E-04	0.322187112	0.156772941	0.662132981	2.06E-03
KLF11	2.95E-03	0.203981094	0.089540494	0.464686812	1.54E-04
KLC4	3.35E-02	0.121412812	0.037069721	0.397657997	4.95E-04
KLC3	8.26E-05	0.332310967	0.178059533	0.620189084	5.39E-04
KIT	5.61E-04	1.61819422	1.171864213	2.23451873	3.46E-03
KIRREL-IT1	1.10E-02	34226.49651	19.87897233	58929256.69	6.03E-03
KIRREL	5.93E-05	4.443470044	2.066404214	9.554967947	1.35E-04
KIR3DL3	3.27E-02	1.32E+41	2712.959651	6.39E+78	3.25E-02
KIR3DL1	4.49E-03	57194019.84	11.83629133	2.76367E+14	2.29E-02
KIR2DL3	1.32E-02	215138.0123	3.691594407	12537770735	2.83E-02
KIFC3	4.54E-02	0.345152536	0.165937784	0.717921318	4.42E-03
KIF9	1.42E-05	0.019155982	0.002641456	0.138920224	9.13E-05
KIF7	2.93E-02	0.250440569	0.100671662	0.623020196	2.91E-03
KIF6	1.04E-04	0.058825498	0.008867529	0.390237156	3.34E-03
KIF26A	3.42E-02	2.909197773	1.202236762	7.039737886	1.79E-02
KIF20A	2.39E-02	4.003178441	1.99191191	8.045254184	9.82E-05
KIF19	4.94E-02	481.4594306	1.880860597	123243.1493	2.90E-02
KIF13B	2.60E-02	0.477933294	0.237174637	0.963088787	3.89E-02
KIAA1644	2.63E-03	1.697799434	1.136609351	2.536071796	9.73E-03
KIAA1598	2.17E-02	2.031527125	1.214346117	3.398621202	6.94E-03
KIAA1549L	2.88E-03	0.21636529	0.077707883	0.60243488	3.39E-03
KIAA1549	3.04E-02	0.107154363	0.026902262	0.426806396	1.54E-03
KIAA1429	4.60E-02	2.301771137	1.270080063	4.171508964	6.00E-03
KIAA1217	6.84E-06	0.26264322	0.127977841	0.539010974	2.68E-04
KIAA1045	5.88E-04	0.236746451	0.11160866	0.502191155	1.73E-04
KIAA0922	2.67E-02	0.206227643	0.08115492	0.524057449	9.07E-04
KIAA0895	8.37E-03	0.033635025	0.002440827	0.463496636	1.13E-02
KIAA0513	4.67E-03	1.905902272	1.140579725	3.18475192	1.38E-02
KIAA0196	7.97E-04	2.0760217	1.40226124	3.073511535	2.63E-04

KIAA0020	5.24E-03	1.94622572	1.216633313	3.113341145	5.47E-03
KHDRBS3	2.07E-03	3.91059044	2.078174886	7.358725047	2.36E-05
KDM6B	2.81E-02	0.141019575	0.034233139	0.580914312	6.69E-03
KDM5B	6.23E-04	0.111241168	0.032559985	0.380055382	4.60E-04
KDM4D	4.49E-02	0.162434471	0.029707595	0.888155283	3.60E-02
KDM4B	1.14E-03	0.025602541	0.003989566	0.164301092	1.12E-04
KDF1	1.05E-02	0.074017508	0.008032551	0.682048777	2.16E-02
KDELR3	5.85E-08	4.876110019	2.568753255	9.256026778	1.27E-06
KDELR2	1.00E-04	9.844181938	2.916498737	33.22748499	2.29E-04
KDELC1	2.53E-02	0.339455564	0.141476555	0.814481804	1.55E-02
KCTD9P6	1.76E-02	4.28E+20	3073834.548	5.97E+34	4.25E-03
KCTD6	9.04E-03	0.111007691	0.030340519	0.406146892	8.96E-04
KCTD17	4.27E-06	3.595316022	1.953629664	6.616554577	3.92E-05
KCTD13	4.86E-02	0.27236279	0.094040213	0.788827321	1.65E-02
KCTD12	2.00E-02	1.849604044	1.06249618	3.219809333	2.97E-02
KCTD11	4.79E-03	3.159314268	1.155731088	8.636322712	2.50E-02
KCP	1.83E-03	7259910.696	8002.009368	6586633544	5.46E-06
KCNV1	1.80E-02	7.39E+27	45444824.85	1.20E+48	6.88E-03
KCNS3	6.73E-03	0.365604376	0.170559702	0.783693675	9.70E-03
KCNRG	4.93E-03	1729.187651	35.69170601	83775.48363	1.66E-04
KCNQ5-IT1	5.49E-03	0.002111	2.61E-05	0.170550781	5.97E-03
KCNQ5	7.37E-03	0.514656986	0.334072603	0.792857035	2.59E-03
KCNN3	4.80E-04	15603.2681	335.1342818	726460.9697	8.34E-07
KCNMB4	2.97E-03	3.148931989	1.329832549	7.456406959	9.10E-03
KCNMB1	2.05E-02	13.65537348	1.873533084	99.52811955	9.90E-03
KCNK9	9.38E-03	12.49480821	2.46588745	63.31198619	2.29E-03
KCNK6	5.64E-05	5.150930516	1.939249063	13.68162847	1.01E-03
KCNK2	8.61E-04	0.413866665	0.235249761	0.72810113	2.21E-03
KCNK1	7.98E-03	1.971932593	1.259033016	3.088495776	3.02E-03
KCNJ8	1.17E-02	3.804559529	1.740204675	8.31779929	8.14E-04
KCNJ14	3.87E-04	0.00351352	0.00014188	0.087008682	5.58E-04
KCNIP3	1.34E-05	6.889167408	2.930830382	16.19357703	9.61E-06
KCNIP2-AS1	8.69E-04	0.000435786	6.78E-06	0.02802209	2.70E-04

KCNG2	2.36E-02	0.569822932	0.335643189	0.967390921	3.73E-02
KCNE1	2.60E-02	2.27511E+12	28123.59581	1.84E+20	2.19E-03
KCNA5	6.90E-04	0.280063212	0.134773596	0.581979001	6.48E-04
KB-68A7.1	2.77E-02	0.423112291	0.184001532	0.972948484	4.29E-02
KB-1980E6.3	3.56E-02	98.38933235	6.679022027	1449.382961	8.27E-04
KB-1742H10.3	9.39E-03	13.09217801	3.274807119	52.34052538	2.75E-04
KB-1732A1.1	3.35E-02	17.26030006	2.119420639	140.5657531	7.77E-03
KB-1568E2.1	7.23E-05	2.60E+88	3.60E+24	1.88E+152	6.65E-03
KB-1517D11.3	6.93E-04	60.31611473	7.584622106	479.6591901	1.07E-04
KB-1517D11.2	1.77E-02	8.93188E+11	24.13073891	3.31E+22	2.67E-02
KB-1507C5.2	1.70E-02	7.686453347	2.307201076	25.60746252	8.95E-04
KB-1410C5.5	4.44E-03	3.30714395	1.255633279	8.710505916	1.55E-02
KB-1043D8.6	9.55E-03	13.71383264	2.40506298	78.19720619	3.20E-03
KATNAL2	9.38E-04	10.6180367	3.830195134	29.43523749	5.59E-06
KAT8	6.40E-04	0.036579064	0.00716504	0.18674396	6.97E-05
JUP	6.44E-07	0.18428842	0.083325232	0.407586285	2.97E-05
JUNB	6.98E-03	1.641859596	1.06497058	2.531246386	2.48E-02
JTB	5.60E-03	9.244751983	2.791810876	30.61290432	2.72E-04
JPH1	7.48E-05	3.853672549	2.105842951	7.052184072	1.21E-05
JHDM1D-AS1	8.74E-06	5.90292845	2.605105799	13.37548912	2.10E-05
JDP2	5.60E-03	2.877789728	1.126679798	7.350512305	2.72E-02
JARID2-AS1	1.26E-03	0.105082003	0.014935524	0.739326401	2.36E-02
JARID2	1.34E-05	0.180530523	0.075211013	0.433331081	1.27E-04
JAGN1	9.21E-05	0.137325002	0.047315707	0.398560173	2.60E-04
JAG2	9.08E-06	4.209973945	2.08970613	8.48151822	5.76E-05
JAG1	3.92E-02	2.537025895	1.481304766	4.345156069	6.96E-04
JADE1	1.79E-02	0.346805036	0.137144954	0.876982562	2.53E-02
ITPR2	1.44E-03	2.048310558	1.421513093	2.951486105	1.20E-04
ITPKA	3.86E-04	6.182401564	1.567458587	24.38475211	9.27E-03
ITPA	1.89E-05	11.53340622	4.104027851	32.41192892	3.51E-06
ITM2C	1.31E-03	5.096732511	1.94873728	13.33000736	9.00E-04
ITK	4.35E-03	5.180588684	1.580943696	16.97625234	6.60E-03
ITIH6	7.12E-05	2.01730191	1.2163136	3.345771187	6.56E-03

ITGB3	1.49E-03	136.4267608	14.68578496	1267.365763	1.54E-05
ITGB2-AS1	1.97E-02	3.693634344	1.579228607	8.63898653	2.58E-03
ITGB1BP1	1.08E-02	3.999811232	1.671953136	9.568743014	1.84E-03
ITGAX	5.75E-05	2.124152641	1.467897934	3.073799847	6.45E-05
ITGAL	2.86E-04	1.800472276	1.281421622	2.529768783	7.01E-04
ITGA9-AS1	2.82E-02	0.084603849	0.009735668	0.735215215	2.52E-02
ITGA7	4.92E-04	2.195734691	1.138783861	4.233683842	1.89E-02
ITGA6	1.13E-03	4.613594536	2.148024539	9.909223176	8.85E-05
ITGA5	1.11E-04	4.115574622	2.266905665	7.471839139	3.32E-06
ITGA4	1.61E-02	2.596384125	1.11973383	6.020368719	2.62E-02
ITGA2	1.10E-02	6.509424955	1.502576745	28.19996609	1.23E-02
ITGA1	4.42E-02	3.074771468	1.237838907	7.637681712	1.55E-02
ITFG2	4.58E-03	4.521603602	1.541660997	13.26160496	5.99E-03
ISY1	1.67E-03	0.086927328	0.017711873	0.426626836	2.62E-03
ISOC2	2.25E-03	8.91960966	2.830341149	28.10948656	1.87E-04
ISM2	3.06E-05	353.2750945	5.368828512	23245.90776	6.02E-03
ISM1	4.53E-06	1.931502593	1.426377418	2.615508503	2.08E-05
ISG20	3.08E-07	3.335132048	2.108978455	5.274167573	2.59E-07
ISG15	1.34E-04	1.354763441	1.087462614	1.68776743	6.78E-03
ISCA1P4	1.28E-05	3.044881742	1.681564299	5.513500036	2.37E-04
IRX1	3.22E-03	1.744313524	1.242776017	2.448252645	1.30E-03
IRS4	4.52E-02	0.011620918	0.000360946	0.374144273	1.19E-02
IRF8	1.86E-02	2.200177552	1.341950012	3.607273905	1.77E-03
IRF6	8.45E-04	0.155296276	0.045121128	0.534493146	3.14E-03
IRF5	2.32E-04	8.469533569	3.115227737	23.02656658	2.83E-05
IRF2BPL	6.56E-03	3.264567584	1.326607458	8.033575754	1.00E-02
IRF2BP2	4.42E-04	0.295929669	0.130170987	0.672764114	3.66E-03
IRF1	1.29E-04	1.599408248	1.243578986	2.057052082	2.54E-04
IRAK1BP1	1.48E-02	0.140065647	0.023759305	0.825713794	2.99E-02
IRAK1	5.49E-03	3.201995896	1.439611734	7.121904799	4.33E-03
IQUB	1.32E-02	6.00E-07	5.45E-13	0.659726406	4.35E-02
IQSEC1	1.01E-06	0.11106423	0.036668787	0.336396813	1.02E-04
IQGAP1	4.96E-02	2.166150213	1.364397692	3.439031576	1.05E-03

IQCJ-SCHIP1	4.40E-03	121.6829055	2.502722667	5916.248602	1.54E-02
IQCH	3.64E-04	0.000425713	2.39E-06	0.075982569	3.34E-03
IQCG	5.35E-03	0.221595261	0.049425146	0.993511679	4.90E-02
IQCE	3.92E-03	5.339721963	1.889009721	15.09395655	1.58E-03
IPO9	6.65E-04	7.783861314	2.350889654	25.77258224	7.81E-04
IP6K3	1.47E-02	1.733822252	1.078334389	2.787761972	2.31E-02
IP6K2	3.54E-03	0.187433013	0.072837634	0.482321191	5.17E-04
IP6K1	2.81E-04	0.170050918	0.072551464	0.398576583	4.57E-05
INSR	1.55E-02	0.151305402	0.042151124	0.543124893	3.78E-03
INPPL1	5.41E-03	4.051268304	1.480082208	11.0890968	6.47E-03
INPP1	2.27E-02	0.318105072	0.1321083	0.765968805	1.06E-02
INHBB	3.78E-02	2.469529194	1.16476223	5.235896463	1.84E-02
INAFM2	4.15E-03	2.967839985	1.326979724	6.637685578	8.08E-03
IMPDH2	3.10E-02	0.368607017	0.195455404	0.69515158	2.05E-03
IMPAD1	1.43E-02	2.307944708	1.407668929	3.783992573	9.15E-04
IMPA1	1.70E-03	2.115178641	1.434983112	3.117793266	1.54E-04
IMP3	2.80E-04	6.753774147	2.151197394	21.20375628	1.07E-03
ILKAP	3.05E-04	4.010829624	1.81550059	8.860781631	5.93E-04
IL9R	1.37E-02	77.99354164	1.991492524	3054.489266	1.99E-02
IL6	3.52E-02	69.43067512	7.973577645	604.574115	1.23E-04
IL4I1	1.11E-02	2.16263588	1.340751031	3.488338881	1.57E-03
IL37	3.24E-03	97006.55225	82.81231933	113633711.2	1.45E-03
IL36B	2.85E-02	1285257.145	223.7042333	7384240808	1.45E-03
IL32	3.31E-04	1.782158085	1.344115312	2.36295756	5.95E-05
IL31RA	8.82E-07	5.20441E+17	12628708.68	2.14E+28	1.07E-03
IL2RG	4.07E-04	1.649452392	1.257269559	2.163969671	3.03E-04
IL2RB	1.15E-03	1.796478208	1.244560234	2.593152075	1.76E-03
IL27RA	7.03E-03	4.551804528	2.023368097	10.23981968	2.49E-04
IL27	5.07E-04	22.674751	3.728995934	137.8774185	7.01E-04
IL24	3.00E-03	2.175901664	1.050730167	4.505959952	3.63E-02
IL21R	9.09E-04	4.31742191	1.691102417	11.02247372	2.22E-03
IL21	1.31E-03	1689.690289	1.232976102	2315578.759	4.37E-02
IL2	3.42E-03	8.80E+21	4566944822	1.69E+34	4.63E-04

IL1RAPL1	8.78E-03	5.18E-21	1.11E-35	2.42E-06	6.72E-03
IL1R2	1.48E-04	7.484034607	2.459581623	22.77248028	3.92E-04
IL19	1.38E-02	3.59E+49	3.32237E+16	3.88E+82	3.28E-03
IL18RAP	3.17E-02	12.25803276	1.057910902	142.0340474	4.50E-02
IL18BP	6.42E-03	1.692047389	1.252364272	2.28609553	6.13E-04
IL18	2.22E-02	2.149484084	1.164748024	3.966765112	1.44E-02
IL17REL	1.73E-02	11539512.29	1283.134897	1.03777E+11	4.64E-04
IL17RE	3.85E-04	0.034428044	0.004802844	0.246789237	8.01E-04
IL17RD	7.26E-04	0.030953264	0.00177401	0.540078506	1.72E-02
IL17RC	9.71E-04	0.213127573	0.07958521	0.570751305	2.10E-03
IL17RA	5.10E-03	5.629778483	1.072284989	29.55781913	4.11E-02
IL15RA	9.94E-04	3.407468612	1.53963838	7.541278841	2.49E-03
IL13RA1	3.04E-02	2.012384377	1.032899816	3.920700556	3.99E-02
IL12RB2	3.35E-05	0.444426125	0.286216656	0.690087654	3.04E-04
IL12RB1	7.38E-03	1.936939947	1.282966065	2.924267805	1.66E-03
IL12B	2.99E-02	13323842.64	28.06825795	6.32475E+12	1.39E-02
IL11RA	2.91E-03	0.191546213	0.075903709	0.483374955	4.67E-04
IL11	1.35E-03	234.1393435	15.2682361	3590.541291	8.97E-05
IL10RB-AS1	6.24E-03	90.28478716	6.616399744	1231.990676	7.33E-04
IL10RB	2.92E-03	11.01887235	3.567765416	34.03125873	3.04E-05
IL10RA	2.20E-02	1.866722758	1.207963086	2.884735381	4.94E-03
IKZF3	4.21E-04	2.318032626	1.338214615	4.015256741	2.71E-03
IKZF1	7.29E-05	3.61847513	1.684828567	7.771332065	9.75E-04
IKBKE	4.05E-05	6.300174828	2.570607981	15.44078411	5.72E-05
IGSF10	1.31E-03	1.03E-24	6.87E-44	1.55E-05	1.42E-02
IGLVV-58	2.76E-06	1.56607E+18	801457.1415	3.06E+30	3.71E-03
IGLVI-20	2.76E-06	1050.759012	9.559209255	115500.61	3.71E-03
IGLV7-46	2.50E-02	2.35400357	1.467951966	3.774873386	3.81E-04
IGLV6-57	2.14E-02	1.286719842	1.022581499	1.61908655	3.15E-02
IGLV5-48	1.40E-02	134.6201244	6.343035491	2857.082847	1.66E-03
IGLV4-69	1.89E-02	1.725511584	1.17161604	2.541267894	5.75E-03
IGLV3-31	2.76E-06	6.13662E+17	591363.6312	6.37E+29	3.71E-03
IGLV3-21	2.66E-03	1.395139508	1.065765073	1.826307032	1.54E-02

IGLV3-1	4.62E-02	1.276945024	1.052659138	1.54901861	1.31E-02
IGLV2-8	5.87E-03	1.531084303	1.097100201	2.136741149	1.22E-02
IGLV2-5	7.35E-03	51167.72893	59.3900779	44083735.47	1.66E-03
IGLV2-34	9.11E-03	5.945460097	2.14005475	16.51756609	6.28E-04
IGLV2-33	2.42E-02	40.78278954	1.237312451	1344.232753	3.76E-02
IGLV2-14	1.25E-03	1.279040408	1.060965843	1.541938769	9.87E-03
IGLV2-11	3.06E-02	1.274653085	1.025734219	1.58397805	2.86E-02
IGLJ3	1.64E-02	3.162125022	1.470873935	6.798022875	3.20E-03
IGLJ2	3.33E-02	6.646315204	2.006021435	22.02045552	1.94E-03
IGLC3	1.14E-02	1.259965829	1.075384536	1.476229048	4.25E-03
IGKV7-3	1.84E-04	9590157.975	106.9588341	8.59874E+11	5.73E-03
IGKV6D-41	2.76E-06	18.13493934	2.560738536	128.4301463	3.71E-03
IGKV3OR22-2	2.76E-06	115651.1939	43.9439122	304369774.7	3.71E-03
IGKV3D-20	6.91E-03	2.013812374	1.352460055	2.998565662	5.68E-04
IGKV3-11	4.49E-02	1.315941632	1.067041324	1.622900951	1.03E-02
IGKV2D-24	3.11E-02	17.98527749	1.390909973	232.5601317	2.69E-02
IGKV2D-14	2.76E-06	1.44E+21	7340685.189	2.83E+35	3.71E-03
IGKV2-4	1.82E-04	183.7319354	5.154488105	6549.132212	4.25E-03
IGKV2-26	2.15E-02	26.51493268	1.163611797	604.1891777	3.99E-02
IGKV1OR2-9	9.88E-03	2043.638178	4.3871849	951967.4001	1.50E-02
IGKV1OR10-1	1.83E-02	606.8953665	6.567563206	56081.98571	5.52E-03
IGKV1OR-3	2.76E-06	5.65E+20	5416529.825	5.90E+34	3.71E-03
IGKV1D-43	5.62E-03	4.629017624	1.580078099	13.56123104	5.20E-03
IGKV1D-33	8.68E-03	462.8177931	11.5514938	18543.08311	1.12E-03
IGKV1D-32	2.76E-06	1.01991E+19	1472063.987	7.07E+31	3.71E-03
IGKV1-8	1.11E-02	1.535634188	1.003056632	2.35098626	4.84E-02
IGKV1-37	2.42E-02	69171209.22	5.856499771	8.16982E+14	2.98E-02
IGKV1-33	1.24E-04	12.94781801	2.688111294	62.36571813	1.41E-03
IGKV1-16	4.10E-02	1.388860436	1.127087414	1.711431862	2.05E-03
IGKV1-13	3.29E-02	8.113027773	1.25810587	52.31771124	2.77E-02
IGKC	7.13E-03	1.191963999	1.031268472	1.377699614	1.75E-02
IGJ	1.16E-02	1.225987381	1.008254397	1.4907399	4.11E-02
IGHVII-78-1	2.76E-06	1.44658E+16	175279.9548	1.19E+27	3.71E-03

IGHV7-27	4.16E-02	26.31092599	3.172317039	218.2205681	2.45E-03
IGHV5-78	1.53E-02	26.8533254	2.584543222	279.0052334	5.87E-03
IGHV4OR15-8	9.71E-05	16989.22515	13.56402849	21279354.53	7.44E-03
IGHV4-61	1.51E-02	1.779191009	1.151092055	2.750015198	9.51E-03
IGHV4-28	2.85E-02	1.722984979	1.156620598	2.566681972	7.46E-03
IGHV3OR16-16	2.76E-06	2.89E+20	4359369.516	1.92E+34	3.71E-03
IGHV3OR16-11	9.71E-05	119.4434939	1.986579345	7181.564768	2.21E-02
IGHV3OR16-10	1.67E-02	5.817129792	1.905815158	17.75565635	1.98E-03
IGHV3OR15-7	3.39E-03	20.94315639	2.843067619	154.2755426	2.83E-03
IGHV3-76	2.73E-03	132.543276	6.118810325	2871.100601	1.84E-03
IGHV3-74	1.67E-02	1.799567031	1.230190592	2.632471361	2.47E-03
IGHV3-66	2.30E-02	1.364706481	1.011067788	1.842036508	4.22E-02
IGHV3-54	2.76E-06	60579744275	3152.580225	1.1641E+18	3.71E-03
IGHV3-52	2.28E-02	196.5881313	11.85683698	3259.460633	2.28E-04
IGHV3-48	3.15E-02	2.25234203	1.356394041	3.740096512	1.70E-03
IGHV3-47	1.37E-02	3.580040285	1.607141573	7.974834736	1.80E-03
IGHV3-37	4.11E-09	1529409061	13769.58249	1.69874E+14	3.60E-04
IGHV3-36	1.05E-04	202.2235336	1.846995605	22141.01508	2.67E-02
IGHV3-32	2.11E-02	9.90642828	2.173644649	45.14874191	3.04E-03
IGHV3-25	4.16E-02	15664.37065	16.55575694	14820977.92	5.73E-03
IGHV3-23	4.47E-02	1.235646956	1.042489504	1.464593547	1.47E-02
IGHV3-22	2.42E-02	13497.08817	7.684329678	23706867.98	1.26E-02
IGHV3-11	9.66E-03	1.638546527	1.199414034	2.238455316	1.92E-03
IGHV1OR16-2	2.76E-06	6.46E+20	5656926.799	7.38E+34	3.71E-03
IGHV1OR15-3	9.71E-05	838639.2753	288.0947028	2441266109	8.04E-04
IGHV1-2	4.14E-03	1.57055867	1.098471049	2.245534406	1.33E-02
IGHMBP2	4.33E-05	0.053529646	0.01504242	0.190489497	6.18E-06
IGHJ2P	2.17E-02	9.991601651	1.298438318	76.88628882	2.70E-02
IGHJ2	1.87E-02	1.561679471	1.190164667	2.049164152	1.30E-03
IGHJ1	3.06E-03	3.389551348	1.658603023	6.926948876	8.15E-04
IGHG2	6.40E-03	1.245455399	1.059444657	1.464124757	7.82E-03
IGHD6-13	2.76E-06	50.05881184	3.560000441	703.9000932	3.71E-03
IGHD4-17	2.76E-06	2.651575336	1.372205697	5.123759345	3.71E-03

IGHD3-9	9.08E-04	33.39751954	3.023176772	368.9477644	4.20E-03
IGHD3-22	4.16E-02	2.059709812	1.313062748	3.230922908	1.66E-03
IGHD3-10	2.76E-06	11.11088936	2.184369895	56.51600618	3.71E-03
IGHD2-8	9.71E-05	2.884274929	1.170128381	7.109512087	2.14E-02
IGHD2-15	9.71E-05	10.10738739	1.945198948	52.51867942	5.94E-03
IGHD1OR15-1A	1.99E-03	16.66372267	1.625057574	170.8737326	1.78E-02
IGHD1-1	5.16E-03	12.09112255	1.343112576	108.8480944	2.62E-02
IGHD	4.07E-03	2.575028478	1.455989388	4.554134608	1.15E-03
IGHA2	2.79E-03	1.258793883	1.02889832	1.540056979	2.53E-02
IGFL1	3.83E-02	498.7103814	3.946162515	63026.30556	1.19E-02
IGFBPL1	4.65E-02	0.22790319	0.069484986	0.747497656	1.47E-02
IGFBP7	6.15E-05	2.798404165	1.670042446	4.689141817	9.34E-05
IGFBP4	3.08E-03	2.011279694	1.196813994	3.380012291	8.33E-03
IGFBP2	2.19E-04	2.174092943	1.470209074	3.214971399	9.99E-05
IGF2BP3	3.71E-03	2819785.022	33.62099791	2.36495E+11	1.02E-02
IGF2BP2-AS1	2.27E-02	3.42E-43	6.87E-73	1.70E-13	5.07E-03
IGF2BP2	9.29E-05	0.407941616	0.236594648	0.70338177	1.26E-03
IGF1	1.25E-02	2163.825592	12.2068353	383567.1637	3.65E-03
IFT172	4.09E-04	0.358330781	0.133451392	0.962155185	4.17E-02
IFT122	1.94E-07	0.169308005	0.074004781	0.387342551	2.60E-05
IFRD2	1.42E-04	0.207518832	0.072686656	0.592461786	3.30E-03
IFNL1	5.49E-04	9290745379	3406.672586	2.53379E+16	2.40E-03
IFNG	8.69E-03	2.41186979	1.224075871	4.75225108	1.10E-02
IFNAR2	2.33E-04	8.040886563	2.789903495	23.17494381	1.14E-04
IFNAR1	2.76E-02	2.826961891	1.277008093	6.258154177	1.04E-02
IFITM9P	4.06E-05	10.15698799	2.591267242	39.81233715	8.81E-04
IFITM3	3.10E-03	1.873237437	1.137756362	3.084156339	1.36E-02
IFITM2	1.53E-03	2.336694784	1.184729308	4.608767993	1.43E-02
IFITM1	9.58E-05	1.320112228	1.066349957	1.634263013	1.08E-02
IFIT1B	1.36E-02	23293007563	96.99131383	5.59395E+18	1.53E-02
IFI6	3.50E-02	1.27778346	1.027950827	1.588335285	2.72E-02
IFI35	3.86E-02	1.935164353	1.165721352	3.212483899	1.07E-02
IFI30	4.59E-02	9.193806346	1.863531577	45.35800529	6.44E-03

IFI27	1.40E-03	1.32765701	1.119203012	1.574936019	1.14E-03
IFI16	3.57E-02	1.45962283	1.048942197	2.031092668	2.49E-02
IER5L	4.09E-02	2.155441827	1.288009763	3.607060755	3.46E-03
IDUA	1.66E-04	3.455041975	1.867535178	6.392016167	7.82E-05
IDO1	2.15E-04	1.645622112	1.225615532	2.20956088	9.23E-04
IDI2-AS1	7.29E-03	0.458790053	0.242855456	0.866722599	1.64E-02
IDH3B	1.18E-02	3.732279675	1.485167644	9.379352983	5.09E-03
IDH2	3.94E-05	5.348923508	2.733837167	10.46550359	9.74E-07
ID3	8.38E-03	2.057049859	1.228620287	3.444069876	6.09E-03
ID2	1.59E-02	0.585284584	0.395431425	0.86628938	7.42E-03
ID1	4.81E-02	1.589068408	1.011269829	2.496997669	4.46E-02
ICOS	7.58E-03	3.626149477	1.353926182	9.711725942	1.04E-02
ICAM3	3.79E-06	7.105387422	2.22018225	22.73981355	9.54E-04
IBSP	3.76E-03	3.181593519	1.651135094	6.130653608	5.43E-04
IAH1	3.64E-04	12.42594548	4.211285585	36.66436718	5.01E-06
HYLS1	2.28E-02	6.618021252	2.073138495	21.12652165	1.42E-03
HYKK	3.95E-05	0.101477339	0.024149876	0.426405922	1.79E-03
HYDIN	1.52E-03	3.12E-21	9.11E-36	1.07E-06	5.69E-03
HYAL2	7.14E-03	0.235456447	0.093331047	0.594011749	2.19E-03
HYAL1	1.36E-05	0.038119956	0.006567831	0.221249766	2.71E-04
HVCN1	3.74E-03	3.071315566	1.325002317	7.119217216	8.90E-03
HUS1	1.13E-02	8.934769731	1.793592881	44.50848964	7.52E-03
HTRA3	1.59E-06	2.872887371	1.808825002	4.562896817	7.79E-06
HTRA1	4.97E-06	1.745948064	1.307229076	2.331905475	1.60E-04
HTR7	2.48E-02	1162.304506	4.022162536	335876.9698	1.46E-02
HTR2B	2.73E-04	1.453577216	1.224078472	1.726103981	1.99E-05
HTATIP2	5.39E-07	2.108358095	1.47605274	3.011527797	4.12E-05
HSPH1	1.56E-02	2.218072174	1.25022961	3.935152492	6.46E-03
HSPE1P3	2.23E-02	20.36820657	2.360848916	175.7265516	6.12E-03
HSPE1P2	1.81E-03	8.585253123	1.151408984	64.01424011	3.59E-02
HSPE1P14	1.04E-02	355349.5332	10.81684433	11673764264	1.60E-02
HSPE1P13	2.84E-02	101242.1082	7.329431303	1398466544	1.78E-02
HSPE1-MOB4	5.00E-04	9820208.372	2907.16634	33171989906	1.03E-04

HSPE1	2.44E-02	3.42065892	1.481157536	7.899839932	3.98E-03
HSPB8	7.07E-03	1.461835856	1.130740904	1.889879513	3.76E-03
HSPA9	2.59E-02	2.383715803	1.105719438	5.138827115	2.67E-02
HSPA8P7	2.63E-02	58298375480	4.954576604	6.86E+20	3.62E-02
HSPA8P5	4.44E-02	448018.7676	62.90579371	3190816049	4.04E-03
HSPA8P4	1.41E-02	3.211293376	1.040267773	9.913221779	4.25E-02
HSPA5	8.91E-03	2.25326069	1.228917946	4.131426149	8.63E-03
HSPA4	2.59E-02	2.790629584	1.255612515	6.202242637	1.18E-02
HSPA1L	3.17E-05	0.081981955	0.02044286	0.328772052	4.16E-04
HSPA12A	3.27E-02	0.447030499	0.248700609	0.80352142	7.12E-03
HSP90B3P	4.45E-02	6920693178	5984.402042	8.00347E+15	1.47E-03
HSP90B1	3.35E-02	1.751014024	1.008174051	3.041191258	4.67E-02
HSP90AB1	4.32E-02	0.350412902	0.154214438	0.796223772	1.23E-02
HSH2D	2.13E-02	5.645870743	2.019035236	15.78766723	9.70E-04
HSFY1P1	3.82E-09	1.28E+79	5.46E+27	2.99E+130	2.54E-03
HSD3BP5	8.99E-03	5.601241985	1.648708104	19.02939137	5.76E-03
HSD3BP4	9.92E-04	125.5939242	11.92322122	1322.950694	5.74E-05
HSD3BP2	1.03E-02	5452247398	8.533927164	3.48339E+18	3.02E-02
HSD17B8	2.90E-04	0.379501715	0.224076652	0.642733416	3.13E-04
HSD17B1P1	9.41E-06	0.143026363	0.054067612	0.3783511	8.92E-05
HSD17B14	2.40E-02	3.247851871	1.634450328	6.453877245	7.73E-04
HSD17B1	5.20E-03	0.07312684	0.013206664	0.404911854	2.74E-03
HSD11B2	2.85E-04	13.24188805	4.175517045	41.99422426	1.15E-05
HSBP1P1	8.87E-04	52243.178	27.52870676	99145581.78	4.79E-03
hsa-mir-4538	1.35E-02	4.095671117	1.708337589	9.819207871	1.58E-03
HS6ST2	2.21E-02	1.968643331	1.015879777	3.814975606	4.48E-02
HS3ST5	2.77E-03	1.27E-06	1.26E-11	0.127478646	2.09E-02
HS3ST3A1	5.24E-03	8.1607378	1.225349766	54.34990341	3.00E-02
HRSP12	1.02E-03	2.30968825	1.489839839	3.580693491	1.82E-04
HRH4	4.36E-04	42169065	6.889913156	2.58092E+14	2.77E-02
HRH2	5.20E-04	1.773584906	1.128012627	2.788624296	1.31E-02
HRC	1.69E-02	3.93589306	1.279069803	12.11134384	1.69E-02
HRASLS	3.12E-05	0.19946867	0.07691273	0.517310339	9.15E-04

HPSE	2.32E-05	3.540317434	1.899875521	6.597194077	6.87E-05
HPS1	1.37E-02	0.280674581	0.08966635	0.878570622	2.91E-02
HPGD	4.61E-04	0.469285269	0.269142798	0.818259543	7.65E-03
HP09025	5.07E-03	731.6858424	14.3195572	37386.92228	1.02E-03
HOXD13	8.43E-04	1180.959734	2.596599699	537112.399	2.35E-02
HOXC4	1.88E-04	2.327955499	1.136828497	4.767101478	2.09E-02
HOXC13	6.07E-03	31.82136559	3.413818002	296.6178359	2.38E-03
HOXB9	2.61E-03	453327862.2	236.6114463	8.68539E+14	6.92E-03
HOXB4	1.09E-04	14.40402957	3.118061989	66.54007158	6.34E-04
HOXB-AS1	6.85E-03	280.7556755	14.81754924	5319.621218	1.73E-04
HOXA13	2.91E-03	8.40615272	1.7123557	41.2667786	8.73E-03
HOXA1	9.56E-04	40.92416788	2.143832576	781.2118982	1.36E-02
HOTAIRM1	4.43E-06	9.873288786	3.020112746	32.27754712	1.51E-04
HOMER2	2.67E-02	0.047166753	0.006090104	0.365297936	3.45E-03
HOGA1	4.96E-05	4.134879842	1.864173039	9.171482989	4.79E-04
HNRNPUL1	1.07E-02	0.06140139	0.010387255	0.362957373	2.08E-03
HNRNPLP2	3.87E-03	0.226084411	0.06714167	0.761288195	1.64E-02
HNRNPH3P1	1.88E-02	2.77E+22	1.340636233	5.74E+44	4.87E-02
HNRNPCL4	5.84E-03	6.74E+24	34185.39984	1.33E+45	1.65E-02
HNRNPAB	8.54E-05	4.916715284	1.560077346	15.49544274	6.54E-03
HNRNPA3P14	1.01E-02	5.28091E+15	430.8597562	6.47E+28	1.86E-02
HNRNPA1P21	1.44E-02	4.247092241	1.449852198	12.44112505	8.36E-03
HNRNPA1P10	1.88E-02	0.03486603	0.002186288	0.556029157	1.75E-02
HNRNPA1P1	1.36E-02	9.19E-13	4.23E-23	0.019951488	2.25E-02
HNRNPA1	5.56E-03	0.419474715	0.219245086	0.802567753	8.68E-03
HNMT	8.37E-05	0.207731819	0.103197454	0.41815478	1.07E-05
HN1L	1.06E-02	3.282286815	1.066284717	10.10368672	3.83E-02
HMOX1	2.19E-03	2.334473768	1.59857086	3.409149952	1.14E-05
HMHA1	4.35E-02	2.359098512	1.204488991	4.620503659	1.23E-02
HMGXB3	6.15E-03	2.869293518	1.093032572	7.532113411	3.23E-02
HMGN2P7	4.12E-02	5.61E-06	4.14E-11	0.76217863	4.50E-02
HMGN2P46	3.07E-03	8.94E-07	1.55E-11	0.051514862	1.28E-02
HMGN2P38	1.22E-02	261215.7968	48.03127852	1420609541	4.48E-03

HMGN2P22	2.46E-03	19392.13619	17.0402285	22068656.31	5.96E-03
HMGN2P15	1.98E-05	0.184076144	0.069704791	0.486107572	6.36E-04
HMGN1P28	1.63E-05	1839150896	114122.8447	2.96389E+13	1.59E-05
HMGN1P13	7.27E-04	31.22842258	1.278666868	762.6805711	3.48E-02
HMGB3P8	5.28E-04	4.66E+25	628126.4456	3.46E+45	1.13E-02
HMGB3P6	4.84E-02	4706272902	12.10250156	1.83012E+18	2.73E-02
HMGB3P24	7.05E-03	2.727378669	1.043240741	7.13027599	4.07E-02
HMGB1P5	1.69E-02	2.042081582	1.02360182	4.073944677	4.27E-02
HMGB1P45	1.83E-03	7.55298E+14	1578.471274	3.61E+26	1.25E-02
HMGB1P36	4.52E-04	3351695504	7015.819021	1.60122E+15	1.01E-03
HMGB1P10	1.10E-02	3.822511067	1.295967314	11.27466002	1.51E-02
HMGA2	4.72E-02	1.25996E+11	1587.830416	9.99799E+18	5.89E-03
HMCES	1.17E-08	0.023986881	0.004736966	0.121463905	6.57E-06
HMBS	1.71E-02	8.175696666	1.943486578	34.39283642	4.15E-03
HM13-AS1	9.35E-03	20.41613137	2.025247049	205.8111479	1.05E-02
HM13	4.07E-06	3.964654577	2.131816822	7.373281679	1.35E-05
HLF	1.48E-02	0.234492958	0.069431421	0.791960562	1.95E-02
HLCS	3.57E-04	4.828924242	1.868510155	12.4797338	1.15E-03
HLA-S	2.80E-02	1.670904189	1.060776228	2.631960198	2.68E-02
HLA-J	5.43E-03	1.879448622	1.233777389	2.863018202	3.30E-03
HLA-H	3.17E-02	1.450338195	1.07690496	1.953265107	1.44E-02
HLA-G	2.22E-02	1.450094978	1.004487889	2.093380584	4.73E-02
HLA-F	3.36E-03	1.441030612	1.131384907	1.835422421	3.08E-03
HLA-E	6.87E-03	1.65888716	1.212727435	2.269188056	1.54E-03
HLA-DRB6	3.68E-03	1.418992723	1.111296737	1.811883613	5.01E-03
HLA-DRB5	1.65E-03	1.340249471	1.106705468	1.623077409	2.72E-03
HLA-DRB1	1.94E-04	1.438658973	1.150648011	1.798760022	1.42E-03
HLA-DRA	6.62E-04	1.393716977	1.122037026	1.731179068	2.69E-03
HLA-DQB2	9.59E-03	1.571327296	1.199578866	2.058280235	1.03E-03
HLA-DQB1-AS1	1.84E-02	1.833845461	1.099255193	3.059334354	2.02E-02
HLA-DQB1	1.58E-03	1.454247782	1.147871451	1.842398475	1.92E-03
HLA-DQA2	3.20E-02	1.36509507	1.094487676	1.702608985	5.76E-03
HLA-DQA1	8.07E-04	1.589357392	1.208777566	2.089761582	9.08E-04

HLA-DPB2	2.64E-02	2.661356283	1.380020475	5.132400129	3.49E-03
HLA-DPB1	3.26E-02	1.577304825	1.168976374	2.128264151	2.87E-03
HLA-DPA1	2.36E-03	1.482704213	1.153785486	1.905390395	2.09E-03
HLA-DOB	7.14E-03	2.523463449	1.253916874	5.07838112	9.48E-03
HLA-DOA	4.00E-03	1.634003322	1.168724189	2.284514073	4.08E-03
HLA-DMB	3.10E-03	1.637826382	1.128470574	2.377089238	9.43E-03
HLA-DMA	1.16E-02	1.770259872	1.263790574	2.479698837	8.95E-04
HLA-C	1.09E-02	1.564751817	1.196928235	2.0456099	1.06E-03
HLA-B	1.12E-03	1.467970218	1.179859903	1.82643427	5.74E-04
HLA-A	8.16E-03	1.822901791	1.295026607	2.565948005	5.77E-04
HKR1	3.49E-04	0.213005907	0.083165813	0.545554894	1.27E-03
HK3	3.35E-03	3.022550939	1.407480163	6.490900844	4.56E-03
HJURP	3.11E-02	3.047297004	1.368031831	6.787867664	6.39E-03
HIST3H2BA	1.86E-02	62087551.18	481.0360312	8.01367E+12	2.80E-03
HIST2H2BB	4.69E-03	4.194920831	1.773761876	9.920926262	1.09E-03
HIST2H2AA4	1.42E-03	8.50888E+12	275678.6654	2.63E+20	7.15E-04
HIST1H4G	1.88E-02	3.85064E+11	1.163377043	1.27E+23	4.87E-02
HIST1H2BO	4.07E-02	10.3037697	1.137513939	93.3330718	3.80E-02
HIP1R	2.32E-03	0.302659287	0.144285025	0.634872842	1.57E-03
HIP1	1.88E-04	6.107715202	2.508907618	14.86869613	6.71E-05
HINT2	6.93E-04	3.359405369	1.474294764	7.654917263	3.93E-03
HINFP	2.56E-02	0.195724851	0.050740087	0.754989188	1.79E-02
HILS1	1.12E-04	0.509763682	0.351731503	0.738799367	3.72E-04
HIGD1AP4	1.99E-03	5.721E+14	352.3949806	9.29E+26	1.78E-02
HIF1AN	4.20E-02	0.082544299	0.012835945	0.530818834	8.62E-03
HIC1	9.06E-04	9.243318749	2.416965137	35.3496789	1.16E-03
HHATL	2.37E-02	0.849250555	0.725039181	0.994741421	4.28E-02
HFE	1.69E-04	0.109095348	0.028169127	0.422512036	1.34E-03
HEXIM1	1.16E-02	2.808798436	1.39438071	5.657958831	3.85E-03
HEXB	3.95E-04	4.689149551	1.963593145	11.19790195	5.03E-04
HES6	1.06E-04	2.459543695	1.530424739	3.952729614	2.01E-04
HES2	1.40E-02	22.2278746	2.867315087	172.3139571	3.00E-03
HES1	5.47E-03	1.90923198	1.080143176	3.374707013	2.61E-02

HERC2	4.46E-02	2.616751649	1.312620499	5.216579508	6.28E-03
HEMK1	6.10E-05	0.102959251	0.025096632	0.422391636	1.60E-03
HECTD1	1.16E-02	2.098490453	1.206513751	3.649906332	8.67E-03
HDLBP	3.12E-04	4.816525357	1.663765749	13.94361948	3.75E-03
HDGFP1	2.65E-02	1.968540088	1.021232176	3.79458283	4.31E-02
HDAC8	1.39E-02	5.342745414	1.780855724	16.02877099	2.79E-03
HDAC6	1.88E-03	0.336298823	0.152963406	0.739372253	6.70E-03
HDAC4	1.08E-02	3.371467698	1.716801752	6.62091265	4.16E-04
HDAC11	3.11E-06	0.182527928	0.083645834	0.398303694	1.94E-05
HDAC10	1.22E-02	4.634309909	1.721502386	12.47563088	2.40E-03
HCP5	1.86E-03	1.511690609	1.2295789	1.858529369	8.82E-05
HCN3	3.10E-02	0.094051424	0.021404118	0.413269564	1.75E-03
HCN2	3.50E-05	1.593342112	1.108849779	2.289524815	1.18E-02
HCLS1	8.89E-04	1.693426317	1.143269139	2.508326862	8.59E-03
HCK	3.81E-03	2.093835975	1.327681282	3.302109588	1.48E-03
HCG4P11	2.92E-02	3.837598228	1.597929486	9.216401779	2.63E-03
HCG20	1.38E-03	0.330570609	0.143811602	0.759861693	9.14E-03
HCG17	1.24E-02	24.71761358	1.381872783	442.1249398	2.93E-02
HCG16	1.80E-04	0.054598558	0.011396356	0.261575056	2.75E-04
HCG15	2.98E-04	0.026771429	0.002545549	0.281554	2.56E-03
HBE1	1.45E-02	5084.835984	50.18227646	515232.8433	2.93E-04
HBD	1.85E-03	16.99931136	1.307207396	221.0640696	3.04E-02
HAX1	2.53E-03	23.99959533	6.43140943	89.5574418	2.24E-06
HAVCR2	6.08E-04	2.008263545	1.223933286	3.295214301	5.78E-03
HAVCR1	5.95E-03	3197845.71	2.938178541	3.48046E+12	3.47E-02
HAUS8	4.89E-04	16.52681161	3.09103648	88.36372645	1.04E-03
HAUS4P1	1.09E-03	1.41E+24	966784.1478	2.05E+42	9.17E-03
HAS2	2.79E-02	0.097338656	0.010398067	0.911209201	4.12E-02
HAP1	5.30E-04	3.499045394	1.401588797	8.735314302	7.29E-03
HAGH	1.75E-03	3.914281734	1.370321996	11.18102281	1.08E-02
H3F3BP1	1.90E-02	37.09123704	4.309258449	319.2567542	1.00E-03
H2AFZP6	1.15E-02	1447275.474	4.535055346	4.6187E+11	2.83E-02
H2AFZP5	1.64E-06	9.71E+21	12323537.97	7.65E+36	3.82E-03

H2AFY	1.51E-02	4.691312454	1.309659251	16.80468605	1.76E-02
H1FX-AS1	1.97E-02	0.035277807	0.003229545	0.385355733	6.11E-03
H1FX	6.25E-05	0.319893445	0.176722235	0.579054558	1.67E-04
GZMK	3.09E-04	1.539912443	1.186838169	1.998023314	1.16E-03
GZMH	3.81E-05	1.990316855	1.400253056	2.829032342	1.25E-04
GZMB	1.35E-04	1.901623125	1.298190639	2.785546592	9.67E-04
GZMAP1	3.79E-03	2637.954802	1.872710817	3715899.687	3.32E-02
GZMA	4.37E-05	1.53205821	1.208060494	1.942951011	4.33E-04
GYG2	7.94E-06	0.263966238	0.140857389	0.494671776	3.23E-05
GUSBP5	2.04E-02	12.62760829	1.402824092	113.6682013	2.37E-02
GUSB	1.55E-05	18.36767385	6.215018809	54.28325369	1.41E-07
GULOP	2.34E-02	1.95E-07	6.50E-13	0.058550053	1.64E-02
GUK1	1.28E-03	4.435086728	1.450492105	13.56091096	9.00E-03
GUCY2C	9.39E-03	17830.66581	219.7068974	1447076.296	1.28E-05
GUCD1	1.02E-02	5.196544722	1.222795176	22.08389236	2.56E-02
GTSE1-AS1	1.68E-04	0.013471059	0.000816882	0.222148843	2.60E-03
GTSE1	3.40E-02	3.985979942	1.589261873	9.997116501	3.20E-03
GTPBP6	9.38E-03	0.217459687	0.088362632	0.535166443	8.98E-04
GTPBP1	4.67E-02	0.285625913	0.096779831	0.84296657	2.32E-02
GTF3A	4.65E-03	4.462764982	1.880869568	10.58886359	6.91E-04
GTF2IRD1P1	5.56E-03	11776.58913	113.3111206	1223957.991	7.61E-05
GTF2IRD1	2.72E-04	0.114770523	0.026587854	0.495424447	3.72E-03
GTF2H4	3.39E-03	0.206649526	0.063551195	0.671962609	8.77E-03
GTF2F1	2.11E-02	0.179888079	0.064973302	0.498046426	9.62E-04
GTF2A2	2.36E-02	4.030759443	1.261291731	12.881256	1.87E-02
GSTO2	4.74E-05	0.158258729	0.046136386	0.542864919	3.38E-03
GSTO1	4.55E-05	3.747641721	1.736939112	8.085959016	7.59E-04
GSTM2P1	1.11E-02	10682.7334	5.931715579	19239087.12	1.53E-02
GSTM2	2.59E-02	0.358897923	0.174103718	0.739833246	5.50E-03
GSTK1	1.33E-03	18.66030466	4.919188439	70.78545057	1.69E-05
GSTA3	9.91E-07	0.473968141	0.315908433	0.711110486	3.10E-04
GSS	3.31E-03	3.851728269	1.85357871	8.003874117	3.02E-04
GSDMD	6.80E-03	3.149701453	1.660732289	5.973641452	4.43E-04

GSDMC	7.49E-03	167321075.3	1068.014188	2.62135E+13	1.92E-03
GS1-594A7.3	1.37E-02	2.87E-14	1.89E-27	0.43461504	4.40E-02
GS1-421I3.5	3.82E-09	1.99E+40	1.34763E+14	2.93E+66	2.54E-03
GS1-259H13.2	1.16E-03	6.695262033	2.311604461	19.39195674	4.58E-04
GS1-204I12.3	9.83E-04	1.02E+60	2.0747E+12	5.06E+107	1.37E-02
GRPEL1	4.88E-02	2.974378125	1.133137749	7.807457863	2.68E-02
GRK5	5.82E-03	3.871142577	1.332304415	11.24798859	1.29E-02
GRK4	3.22E-02	3.84196461	1.201072785	12.28958998	2.33E-02
GRIN2D	6.54E-05	6.1410224	2.062906232	18.28108109	1.11E-03
GRIN2A	1.79E-02	3.509109955	2.054038617	5.994947014	4.34E-06
GRIK3	1.51E-04	0.442124083	0.231444114	0.844582746	1.35E-02
GRIK1-AS1	8.06E-04	22.65982327	4.818566135	106.5602456	7.79E-05
GRID1-AS1	9.52E-03	30.24427805	1.22056341	749.4214125	3.74E-02
GRID1	3.55E-05	1.820275206	1.31673396	2.516379106	2.89E-04
GREM1	3.06E-03	5147.441582	12.94590838	2046681.782	5.13E-03
GREB1L	4.88E-04	150.5405226	8.239372696	2750.506595	7.18E-04
GREB1	3.25E-05	2.918774822	1.740891268	4.893612036	4.85E-05
GRAP2	1.98E-04	6.017082215	2.16254921	16.74194428	5.88E-04
GRAMD4P7	1.67E-02	35611.74972	52.9930857	23931362.02	1.60E-03
GRAMD4P6	5.28E-04	1.24742E+11	320.9276733	4.84864E+19	1.13E-02
GRAMD3	3.34E-02	0.371995335	0.171138129	0.808589707	1.25E-02
GRAMD1A	4.24E-03	0.214982071	0.05542055	0.833937792	2.62E-02
GPX8	4.06E-04	2.648039946	1.506579276	4.654328962	7.14E-04
GPX3	1.41E-02	0.73198984	0.542406185	0.987837419	4.14E-02
GPX1	4.85E-03	3.241210829	1.303090149	8.061950009	1.14E-02
GPSM1	1.45E-02	0.518012129	0.271395049	0.988730513	4.61E-02
GPR89B	3.59E-03	144.2470913	14.21547528	1463.702265	2.61E-05
GPR89A	2.32E-03	74.05947935	9.71846527	564.3696127	3.26E-05
GPR68	1.10E-04	4.737268095	1.738289183	12.91022761	2.36E-03
GPR65	2.07E-03	4.230971087	1.313457384	13.62900431	1.57E-02
GPR63	1.74E-03	1806.530595	107.1137544	30468.10197	1.97E-07
GPR62	8.32E-06	0.002508235	0.000145121	0.043351774	3.81E-05
GPR56	6.26E-05	2.297778431	1.344508251	3.926926972	2.35E-03

GPR55	1.99E-03	25.53797966	2.779436962	234.6476693	4.19E-03
GPR42	4.04E-05	849.9968863	36.88972038	19585.25842	2.51E-05
GPR37	4.29E-02	0.643455332	0.465695909	0.889066784	7.52E-03
GPR31	2.86E-03	5.80595E+11	22085.84946	1.52627E+19	1.89E-03
GPR27	9.62E-05	0.451774918	0.289942801	0.703933935	4.46E-04
GPR25	4.64E-04	4.646141575	1.062571481	20.31546293	4.13E-02
GPR20	5.00E-03	72.73910704	10.1304059	522.2868409	2.02E-05
GPR19	2.31E-02	2.919315941	1.376078467	6.193255523	5.24E-03
GPR18	7.03E-03	4.548866514	1.606519655	12.88013284	4.34E-03
GPR176	2.47E-02	1.954377789	1.213294034	3.148117797	5.87E-03
GPR174	2.12E-02	2.885665557	1.291633625	6.446925467	9.77E-03
GPR17	5.06E-04	2.14E-05	2.73E-08	0.016871993	1.58E-03
GPR156	3.29E-06	0.001086305	1.89E-05	0.06231526	9.55E-04
GPR153	5.84E-06	0.410970794	0.257084829	0.656969897	2.03E-04
GPR143	6.69E-03	5.029739495	2.301254982	10.9932535	5.14E-05
GPR126	1.32E-02	16.99193337	2.22472762	129.7802918	6.32E-03
GPR108	1.79E-03	4.83682899	1.813286864	12.90193799	1.64E-03
GPNMB	1.47E-03	5.583938154	1.893340707	16.46843867	1.83E-03
GPM6B	2.26E-02	2.046172906	1.294433204	3.234484057	2.18E-03
GPD1L	1.29E-04	0.194076844	0.077171352	0.488080365	4.93E-04
GPC4	3.66E-04	3.059301764	1.660344902	5.636977758	3.36E-04
GPC1	2.36E-03	3.243310144	1.679845211	6.261922601	4.56E-04
GPBAR1	3.64E-03	4.117428119	1.251011433	13.55160622	1.99E-02
GPAA1P1	1.34E-07	6.81E+20	874.9645947	5.31E+38	2.25E-02
GPAA1	1.83E-03	2.622259971	1.360715877	5.053404219	3.97E-03
GOSR2	9.30E-03	6.832762373	1.468977792	31.78172053	1.43E-02
GORASP1	3.73E-04	0.19863675	0.078750353	0.501033415	6.17E-04
GOLM1	8.21E-04	2.715958399	1.504977657	4.901355173	9.10E-04
GOLGA6L6	5.28E-04	1.00E+176	5.69E+39	Inf	1.13E-02
GNPTG	1.92E-03	8.689219445	2.747381289	27.48163674	2.33E-04
GNPTAB	3.37E-02	1.732810817	1.064014455	2.821985466	2.72E-02
GNPDA1	1.74E-04	10.24247319	2.650113875	39.58632045	7.44E-04
GNPATP	5.28E-04	1.13E+87	4.61165E+19	2.77E+154	1.13E-02

GNLY	3.30E-04	1.852181408	1.129860837	3.036281865	1.45E-02
GNB5	4.77E-04	1.746777815	1.125976907	2.709853741	1.28E-02
GNB1L	3.40E-03	9.970852996	2.164962648	45.92130473	3.17E-03
GNAS	2.30E-03	4.830739195	1.842203873	12.66745853	1.36E-03
GNAI1	8.56E-03	0.549600119	0.342879222	0.880952451	1.29E-02
GNA15	8.33E-03	2.544523086	1.008545125	6.419740253	4.79E-02
GNA14-AS1	5.14E-03	3.03821E+11	730.3339294	1.26E+20	9.02E-03
GNA11	2.96E-03	0.134235535	0.045674879	0.394509615	2.61E-04
GMPS	2.77E-02	0.278848781	0.105415164	0.737622934	1.01E-02
GMPPA	1.33E-03	3.677561331	1.690643793	7.999590095	1.02E-03
GML	7.23E-05	2.33E+40	1.62862E+11	3.34E+69	6.65E-03
GMIP	9.40E-03	3.279920744	1.52796062	7.040678893	2.31E-03
GMFG	3.10E-05	2.208441762	1.462417019	3.335037101	1.65E-04
GMDS	7.06E-04	0.224273185	0.075966563	0.662113168	6.80E-03
GMCL1	1.94E-02	2.419880527	1.249074144	4.688129838	8.82E-03
GLYATL1P1	1.89E-02	5.66257E+17	3557348.992	9.01E+28	1.90E-03
GLUD1P7	4.31E-05	0.018099807	0.001922835	0.170374992	4.53E-04
GLUD1	2.01E-02	1.943802472	1.060221367	3.563753918	3.16E-02
GLTP	1.97E-02	3.44036443	1.284602094	9.213831634	1.40E-02
GLRX2	8.54E-05	4.592472898	2.111850003	9.986886989	1.20E-04
GLRX	4.46E-02	3.81314153	1.420192081	10.23808576	7.91E-03
GLIS3	5.70E-03	23.22886472	1.859482342	290.1776177	1.46E-02
GLIS2	1.98E-05	2.683305861	1.490442368	4.830868002	1.00E-03
GLIPR2	2.74E-04	2.653277926	1.486143414	4.737015073	9.68E-04
GLIDR	3.71E-02	0.166247448	0.038426133	0.719255662	1.64E-02
GLI4	2.58E-02	2.619714839	1.25793014	5.455713016	1.01E-02
GLI2	5.86E-06	15.94026864	3.439462736	73.87553926	4.02E-04
GLB1	2.64E-02	0.112180753	0.035838097	0.351149264	1.72E-04
GLA	8.35E-07	7.514212271	3.242221772	17.41502896	2.57E-06
GJC3	9.99E-05	0.197429502	0.077635903	0.502066785	6.57E-04
GJC1	9.59E-04	3.087482808	1.476236918	6.457330781	2.75E-03
GJB1	1.25E-02	0.503209737	0.286177834	0.884834566	1.71E-02
GJA5	7.47E-03	4.556425208	2.033328293	10.21035843	2.30E-04

GJA4	2.63E-04	2.764544869	1.524594739	5.012944186	8.12E-04
GJA3	7.22E-04	2.227384531	1.469357635	3.376469916	1.61E-04
GIPC3	3.00E-02	0.594911632	0.384980275	0.919319437	1.93E-02
GIMAP8	6.53E-03	2.634501235	1.348444234	5.147114414	4.59E-03
GIMAP7	2.92E-02	1.630507872	1.159511512	2.292824084	4.94E-03
GIMAP6	7.35E-04	2.025491696	1.242566501	3.301728002	4.64E-03
GIMAP5	3.97E-02	4.887329897	1.100362709	21.70738188	3.70E-02
GIMAP4	2.69E-05	1.733659791	1.234100736	2.435438358	1.51E-03
GIMAP1	6.36E-04	2.955589567	1.375473469	6.350911073	5.49E-03
GHRL	8.90E-03	0.008778079	0.000141563	0.544313356	2.45E-02
GGTLC1	1.41E-02	154655267.2	63.53074857	3.76483E+14	1.20E-02
GGT1	4.76E-04	2.16234964	1.495484536	3.126582626	4.15E-05
GGPS1	4.29E-03	4.311058228	1.573128684	11.81417848	4.50E-03
GGN	1.93E-03	16.87334725	2.178741766	130.6762701	6.82E-03
GGCX	7.23E-05	4.380042973	1.198927065	16.00162095	2.55E-02
GFRA3	2.12E-02	0.144582666	0.031936509	0.654553291	1.21E-02
GFPT2	1.93E-02	0.346079098	0.152283393	0.786499042	1.13E-02
GFPT1	8.48E-03	2.701446595	1.508672172	4.837242869	8.27E-04
GFER	3.71E-03	0.208726494	0.050168813	0.868403035	3.12E-02
GEM	5.65E-06	1.875863932	1.389308749	2.532817486	4.02E-05
GDF11	9.33E-06	2.329369859	1.50101438	3.614864729	1.62E-04
GCSHP1	5.42E-03	5909253.737	1475.694867	23662940420	2.30E-04
GCSAM	9.30E-04	7.707402256	1.142011568	52.01702959	3.61E-02
GCNT4	5.70E-04	17.81651967	1.420418864	223.4751883	2.56E-02
GCHFR	1.46E-02	12.54029237	3.25111755	48.37073112	2.41E-04
GCH1	7.67E-03	2.911301237	1.621667963	5.226516822	3.44E-04
GCAT	4.58E-03	3.781796732	1.727972076	8.276746317	8.73E-04
GBX2	7.36E-03	1.889946993	1.194089712	2.991315978	6.58E-03
GBP5	7.05E-04	1.68123724	1.161616283	2.433298068	5.88E-03
GBP4	1.14E-02	1.493337006	1.147459779	1.943471531	2.85E-03
GBP2	2.83E-02	1.364050834	1.105220243	1.683496742	3.83E-03
GBP1P1	7.91E-03	1.628463737	1.145053429	2.315956684	6.65E-03
GBP1	2.50E-03	1.374359678	1.123920255	1.680603687	1.95E-03

GBE1	4.14E-02	0.42481391	0.207454921	0.869908786	1.92E-02
GBA	7.96E-03	2.677614846	1.373650617	5.21939216	3.83E-03
GATA6-AS1	6.25E-03	21388425995	167.6888547	2.72806E+18	1.25E-02
GATA6	2.96E-05	13.05271861	2.395925771	71.10965843	2.98E-03
GATA4	2.74E-06	0.144736344	0.036726848	0.570389527	5.74E-03
GATA3	1.70E-02	3.762475408	1.36128983	10.3991236	1.06E-02
GAST	7.67E-05	3353774525	14351.7078	7.83726E+14	5.06E-04
GAS8	4.43E-03	2.361844262	1.222695049	4.562305478	1.05E-02
GAS6-AS2	1.04E-02	5.793290017	1.337398432	25.09514624	1.88E-02
GAS6-AS1	1.61E-02	2.14411177	1.392823727	3.300644003	5.30E-04
GAS1	5.80E-04	0.273962064	0.141500015	0.530425472	1.23E-04
GART	2.79E-04	2.984382585	1.324558787	6.724155619	8.34E-03
GARS	1.84E-03	4.090962583	1.338634168	12.50227677	1.34E-02
GARNL3	1.70E-02	0.230038479	0.060978536	0.867808664	3.01E-02
GAPDHP75	7.53E-06	4405451592	1433.149134	1.35422E+16	3.57E-03
GAPDHP59	4.11E-02	16.10410759	1.541777366	168.2099419	2.03E-02
GAPDHP32	1.57E-02	8.65363485	1.530696046	48.92244695	1.46E-02
GALNT7	2.70E-02	2.924505709	1.434017085	5.964178343	3.16E-03
GALNT18	1.56E-03	1.982978573	1.336580125	2.941988997	6.70E-04
GALNT10	4.27E-04	2.761257201	1.330933036	5.728718969	6.38E-03
GALNS	4.39E-02	3.223428637	1.158905647	8.965779226	2.49E-02
GALM	1.28E-02	0.433617065	0.291591046	0.644820072	3.67E-05
GALK1	3.63E-02	3.534499259	1.615953579	7.73084399	1.57E-03
GAGE2E	2.76E-06	8.89E+23	59014566.79	1.34E+40	3.71E-03
GAGE13	5.28E-04	4.09E+23	215532.9122	7.76E+41	1.13E-02
GAGE12H	7.23E-05	6.86E+22	2201526.346	2.14E+39	6.65E-03
GADD45G	7.62E-05	1.65132113	1.156384142	2.358093107	5.79E-03
GABRP	9.83E-03	0.079105974	0.007366575	0.849479565	3.62E-02
GABRE	1.83E-03	148.8070133	8.284504533	2672.884915	6.87E-04
GABRD	1.19E-02	1.797587603	1.039150161	3.109580609	3.60E-02
GABRA5	1.46E-02	1.692791698	1.173666137	2.441532259	4.85E-03
GABARAPL1	2.94E-05	0.142780463	0.059982562	0.339869787	1.09E-05
GAB3	3.08E-04	9.121790915	2.699948943	30.81801592	3.72E-04

G6PC3	5.32E-06	3.790657864	2.042915971	7.033616284	2.39E-05
G0S2	7.29E-04	4.147795698	2.008487009	8.565755753	1.21E-04
FZD7	1.84E-04	2.062579551	1.425908521	2.983525479	1.21E-04
FZD4	3.20E-02	3.943124909	1.400688683	11.10042099	9.38E-03
FYCO1	2.15E-02	0.354429707	0.165273693	0.760075092	7.70E-03
FXYD5	1.92E-03	2.578694053	1.52341642	4.364967407	4.19E-04
FXYD3	9.34E-03	0.305920937	0.124271382	0.753090679	9.97E-03
FUT3	9.10E-04	0.040284776	0.002785928	0.582521599	1.85E-02
FUT11	1.29E-03	5.483087781	1.633228292	18.40786849	5.89E-03
FUT1	1.23E-02	38.7303882	1.594923422	940.5109669	2.47E-02
FUCA2	2.45E-03	3.122998405	1.30282976	7.486103968	1.07E-02
FUCA1P1	8.40E-05	5.16E+34	1.40186E+15	1.90E+54	5.07E-04
FTSJ2	2.20E-03	7.862319229	1.655957637	37.3294958	9.47E-03
FTSJ1	5.75E-05	8.710057462	2.575446487	29.45706749	4.98E-04
FTLP6	1.96E-03	3.17522E+12	4173.578791	2.42E+21	5.80E-03
FTLP3	1.01E-03	1.955482883	1.280795618	2.985576508	1.89E-03
FTLP2	8.16E-03	2.471508968	1.395615432	4.376819314	1.91E-03
FTLP19	5.28E-04	4.57866E+17	9755.976275	2.15E+31	1.13E-02
FTHL17	5.16E-03	5.63E+36	22361.54147	1.42E+69	2.62E-02
FTH1P8	2.73E-03	1.74872177	1.137982953	2.687235184	1.08E-02
FTH1P7	1.23E-02	3.944261656	1.389958042	11.19256808	9.92E-03
FTH1P5	4.30E-02	4.156755681	1.386884233	12.45858694	1.10E-02
FTH1P3	8.63E-03	4.848656781	2.013923151	11.67347054	4.29E-04
FTH1P20	1.01E-03	3.574282555	1.635848472	7.809706095	1.40E-03
FTH1P2	4.67E-03	4.144595909	1.864380418	9.213610634	4.86E-04
FTH1P16	8.75E-04	3.595663552	1.726381895	7.488955032	6.29E-04
FTH1P12	2.72E-03	6.879215675	1.680848819	28.15458938	7.31E-03
FTH1P10	4.44E-04	11.73054975	2.96844787	46.35614415	4.45E-04
FTH1	4.64E-03	4.040699109	1.10619952	14.75976891	3.46E-02
FSTL4	5.53E-04	2.393435176	1.319914767	4.340077167	4.05E-03
FST	6.19E-04	2.324219441	1.087500099	4.967352203	2.95E-02
FSHR	2.69E-02	2.34E+53	3437458866	1.60E+97	1.70E-02
FRY	1.71E-03	2.608061027	1.228181725	5.538253977	1.26E-02

FRS3	8.94E-03	0.25743706	0.084185861	0.787232433	1.73E-02
FRMPD3	3.20E-03	35.40202204	5.904411587	212.2655486	9.50E-05
FRMD6-AS1	2.62E-02	0.099981121	0.012685458	0.788006585	2.88E-02
FRMD4B	1.64E-02	2.007801799	1.090201324	3.697728094	2.53E-02
FRMD1	1.23E-02	10243.75204	2.7124562	38686138.39	2.80E-02
FRAT1	2.40E-04	0.136471389	0.040880778	0.455579393	1.20E-03
FPR3	6.38E-03	1.940162688	1.219184951	3.087498129	5.17E-03
FP236383.9	5.28E-04	259.2268682	3.509220513	19149.14408	1.13E-02
FP236383.4	5.28E-04	351.7481864	3.759689204	32908.78046	1.13E-02
FOXS1	3.39E-04	3.124647898	1.54500189	6.319360868	1.52E-03
FOXRED1	1.96E-05	7.363821834	2.661116717	20.37711148	1.21E-04
FOXP4	3.36E-04	0.292700998	0.125589926	0.682171545	4.43E-03
FOXO3B	2.01E-02	0.054986921	0.007465818	0.404987281	4.41E-03
FOXO3	1.87E-04	0.2990071	0.14973129	0.597104626	6.23E-04
FOXN3P1	1.10E-03	1.07833E+15	795.8994587	1.46E+27	1.52E-02
FOXL2	2.14E-03	4.488435311	2.051188111	9.821649914	1.71E-04
FOXL1	7.83E-05	474.7336793	25.81956329	8728.732695	3.35E-05
FOXG1-AS1	1.99E-03	1.76E+45	64417302.08	4.80E+82	1.78E-02
FOXF1	1.97E-04	8.397667651	3.172591557	22.228144	1.83E-05
FOXD3-AS1	2.57E-03	0.467160092	0.274184142	0.795956141	5.12E-03
FOXD2-AS1	3.34E-02	2.051005252	1.101587697	3.818690564	2.35E-02
FOXD2	7.71E-05	3.009147259	1.452765066	6.232919166	3.03E-03
FOXD1	4.18E-04	4.972035926	2.672148364	9.251410432	4.14E-07
FOXC2	1.47E-03	6.509922111	1.120111642	37.83469816	3.70E-02
FOXC1	3.92E-04	2.930234349	1.480474855	5.799675227	2.03E-03
FOSL1P1	4.35E-03	1.12965E+12	113.49994	1.12E+22	1.81E-02
FOSL1	4.94E-03	2.485064951	1.489344473	4.146487213	4.92E-04
FOLH1	1.06E-02	10.29253496	1.730779956	61.20724679	1.04E-02
FNTAL1	2.25E-02	9108593647	141.3246471	5.87063E+17	1.24E-02
FNBP1	3.82E-04	2.356684054	1.251793343	4.436802416	7.91E-03
FN1	2.55E-03	2.158665673	1.470530907	3.168813021	8.53E-05
FMO4	2.61E-04	0.155793518	0.057772899	0.420121206	2.39E-04
FMNL1	2.85E-03	4.854695794	2.233411146	10.55250006	6.65E-05

FMN1	1.63E-03	2.135705308	1.342164005	3.398420121	1.37E-03
FLYWCH2	1.94E-03	0.244402945	0.092303265	0.647136364	4.57E-03
FLVCR1	1.43E-02	4.060141097	1.388207035	11.8748467	1.05E-02
FLT4	3.64E-03	6.346763066	1.494717341	26.94917649	1.23E-02
FLT3	1.36E-03	9306.699432	16.28629302	5318254.694	4.78E-03
FLOT2	1.68E-03	2.883476882	1.185484469	7.013536779	1.95E-02
FLNC	3.32E-02	1.668461559	1.120043285	2.485407493	1.18E-02
FLJ44511	1.30E-02	2.550068053	1.210522063	5.37193602	1.38E-02
FLI1	2.55E-03	5.108399207	2.026295577	12.87854682	5.47E-04
FLAD1	2.62E-02	14.30132795	3.174652079	64.42532162	5.32E-04
FKSG68	1.19E-03	2150.662454	3.246405257	1424760.197	2.06E-02
FKBP9	4.79E-02	0.369463398	0.138020515	0.989006615	4.75E-02
FKBP7	3.99E-05	5.063072227	2.063819122	12.42100148	3.96E-04
FKBP5	4.71E-02	3.36683952	1.845582991	6.142020387	7.56E-05
FKBP3	1.73E-03	2.719876148	1.201860988	6.155226215	1.63E-02
FKBP2	2.63E-06	11.96260605	3.734404833	38.32041512	2.94E-05
FKBP1C	2.27E-03	5.772297614	1.563612179	21.30926082	8.52E-03
FKBP1A	2.45E-04	5.504707749	2.483171903	12.20286335	2.68E-05
FKBP11	3.16E-08	3.116112875	1.918888623	5.06030383	4.33E-06
FKBP10	2.78E-06	3.054512317	1.834061225	5.087095985	1.78E-05
FJX1	3.36E-03	2.188911715	1.308164971	3.662637821	2.86E-03
FITM2	1.45E-02	3.111351137	1.271113401	7.615768889	1.29E-02
FILIP1L	3.61E-02	2.819652504	1.197711875	6.638024062	1.76E-02
FICD	1.22E-02	3.940126979	1.325563839	11.71169592	1.36E-02
FIBCD1	1.47E-02	1.768657323	1.22955778	2.544125031	2.11E-03
FHL3	3.10E-02	0.266259322	0.119646905	0.592527039	1.19E-03
FHIT	4.64E-02	0.499025408	0.254537742	0.978347479	4.30E-02
FHDC1	3.87E-03	2.048651086	1.280451068	3.277728746	2.78E-03
FGR	3.35E-02	3.293880003	1.418135762	7.650639499	5.56E-03
FGFR3P5	9.45E-03	1.15273E+12	15.97738395	8.32E+22	2.95E-02
FGF9	1.13E-02	1.47403202	1.066580111	2.037137552	1.88E-02
FGF7P1	4.28E-02	13.55395795	3.015742862	60.91692309	6.75E-04
FGF5	1.46E-03	4861085.477	5078.406881	4653063957	1.10E-05

FGF18	6.26E-03	9506539.527	2551.879167	35414801355	1.28E-04
FGF14-AS2	2.29E-03	0.373465489	0.186509436	0.747825284	5.43E-03
FGD5P1	1.62E-04	1.87E-09	2.62E-16	0.013359085	1.26E-02
FGD5	2.08E-02	1.65262176	1.03298354	2.643951792	3.61E-02
FGD2	4.52E-03	3.803684066	1.47512568	9.80798631	5.70E-03
FFAR3	3.47E-06	2.863659044	1.600886986	5.12249971	3.91E-04
FFAR2	1.03E-02	2.346649523	1.014448518	5.428332619	4.62E-02
FEZ1	2.78E-05	1.605579766	1.099395488	2.344821688	1.43E-02
FERMT3	1.24E-04	2.607040309	1.408706177	4.82475287	2.28E-03
FDXACB1	6.98E-03	7.645741709	1.453630765	40.21472832	1.63E-02
FDX1P1	7.61E-05	890.0521048	17.2711639	45867.94231	7.34E-04
FDPSP5	3.26E-04	1.57E+22	4264495070	5.76E+34	5.36E-04
FDPS	3.21E-03	18.58668448	3.605740802	95.80967102	4.78E-04
FDFT1	5.56E-05	0.43744748	0.279716796	0.684121584	2.90E-04
FCRL6	7.61E-03	5.257821482	1.871176192	14.77396242	1.64E-03
FCRL3	1.50E-02	3.82179706	1.461496848	9.993954338	6.26E-03
FCRL2	4.45E-02	376.8190162	10.19059568	13933.68705	1.28E-03
FCRL1	9.17E-03	461.6052916	5.56032722	38321.38593	6.51E-03
FCGRT	1.88E-02	0.28260784	0.125243021	0.63769774	2.34E-03
FCGR3A	2.73E-02	1.325876707	1.066195681	1.648805257	1.12E-02
FCGR1A	7.20E-03	2.086007782	1.210342656	3.595203758	8.11E-03
FCER2	2.14E-02	78.13132355	6.090315985	1002.329556	8.15E-04
FCER1G	2.86E-02	1.669064626	1.182785034	2.355268833	3.55E-03
FCAR	2.51E-02	19938703762	72.05834929	5.51708E+18	1.68E-02
FBXW9	1.45E-02	0.218284288	0.07773989	0.6129161	3.86E-03
FBXW5	4.32E-02	2.700234286	1.142267788	6.383148749	2.36E-02
FBXW4P1	6.65E-03	4442.47521	83.28439036	236966.2059	3.48E-05
FBXW12	2.29E-04	9.28E-07	5.63E-11	0.015297567	5.05E-03
FBXO7	1.34E-03	0.237214183	0.114994662	0.489332003	9.84E-05
FBXO4	2.00E-02	4.47796106	1.522234754	13.17282712	6.47E-03
FBXO36-IT1	0.00E+00	1.67E+57	125.8512734	2.20E+112	4.19E-02
FBXO32	2.57E-02	1.615981896	1.085215532	2.40633995	1.82E-02
FBXO18	1.78E-02	0.317049818	0.116185603	0.86517249	2.49E-02

FBXO17	5.01E-07	0.325162163	0.185463192	0.570088496	8.79E-05
FBXO15	3.29E-03	0.000129326	2.63E-07	0.063577162	4.63E-03
FBXO10	1.46E-03	14.12740415	2.64345715	75.50095825	1.96E-03
FBXL7	1.14E-04	2.310285893	1.381744781	3.862812424	1.41E-03
FBXL19	4.56E-02	2.486585623	1.070940398	5.773531438	3.41E-02
FBXL15	2.83E-02	0.273465525	0.094217568	0.793730883	1.71E-02
FBP2P1	5.45E-03	4.96E-07	1.03E-12	0.238315256	2.96E-02
FBP2	8.74E-05	0.172341033	0.046091575	0.644400449	8.97E-03
FBP1	5.15E-03	2.213382787	1.33210625	3.677682137	2.16E-03
FBLIM1	4.71E-03	3.607822841	1.541427032	8.444373545	3.10E-03
FBL	1.37E-02	0.392192543	0.19175366	0.802148918	1.04E-02
FAXC	3.85E-02	0.135882908	0.030401081	0.607352248	8.98E-03
FATE1	1.75E-02	17.52588761	1.959245898	156.7729385	1.04E-02
FASTKD2	3.16E-02	2.601519115	1.301806988	5.19885188	6.80E-03
FASTKD1	2.00E-02	2.442934461	1.265393712	4.716262397	7.78E-03
FASTK	3.59E-02	4.021385701	1.337958384	12.08673091	1.32E-02
FASLG	1.06E-03	2.043330021	1.222983323	3.413944815	6.36E-03
FARSBP1	4.45E-03	1.08E+86	13039505050	8.94E+161	2.63E-02
FARSB	1.02E-02	3.099457531	1.130773054	8.495636637	2.79E-02
FANCI	4.90E-02	3.916814314	1.368854064	11.20750179	1.09E-02
FANCE	1.38E-03	0.116425904	0.041228435	0.328777731	4.91E-05
FAM98A	1.38E-03	4.251989663	1.968877769	9.182599536	2.29E-04
FAM96AP2	2.16E-02	9.15E-05	2.06E-08	0.407638288	3.01E-02
FAM92B	2.16E-02	1.84926E+11	532.5436474	6.42154E+19	9.72E-03
FAM92A1	2.58E-02	2.102550903	1.036123541	4.266595752	3.96E-02
FAM8A2P	4.06E-03	1.42E+43	23703.18857	8.46E+81	2.92E-02
FAM86KP	2.57E-02	0.01276428	0.000177682	0.916955457	4.55E-02
FAM86JP	1.19E-03	0.243489716	0.089759814	0.660509854	5.53E-03
FAM86HP	6.07E-07	0.153746756	0.059321183	0.398475949	1.16E-04
FAM86FP	3.65E-03	0.280463254	0.117364164	0.670218523	4.23E-03
FAM86DP	4.79E-05	0.463542298	0.305100881	0.704263657	3.15E-04
FAM86B2	4.12E-02	0.014510786	0.000465127	0.452699788	1.59E-02
FAM86B1	5.90E-03	0.131210964	0.035469018	0.485390301	2.34E-03

FAM83C-AS1	1.28E-02	0.001055823	4.01E-06	0.27814899	1.60E-02
FAM83C	6.94E-03	3.56E+38	77143217.89	1.65E+69	1.37E-02
FAM83A-AS1	4.39E-02	17305024845	23.21588269	1.28991E+19	2.37E-02
FAM72D	3.43E-03	5448566.856	739.487687	40145199585	6.40E-04
FAM72C	1.77E-02	5706.45662	1.336858068	24358342.85	4.26E-02
FAM72A	1.55E-04	7625.525401	86.56750229	671714.3974	9.14E-05
FAM71E1	1.40E-03	0.307317699	0.140145457	0.67390103	3.23E-03
FAM69B	1.24E-02	0.369053658	0.206571994	0.659337213	7.61E-04
FAM57A	2.38E-02	2.533238521	1.018052593	6.303502832	4.57E-02
FAM53B-AS1	6.85E-03	7.513425129	2.239467123	25.2075847	1.09E-03
FAM53A	9.69E-03	10.18661448	3.309215322	31.35701504	5.21E-05
FAM50B	3.38E-04	0.277810832	0.126582192	0.609713399	1.40E-03
FAM50A	1.54E-05	13.302998	4.16659885	42.47343267	1.25E-05
FAM49B	1.95E-03	1.978594129	1.315977515	2.974849252	1.04E-03
FAM47E-STBD1	3.96E-02	10.7715716	1.202559352	96.48318371	3.36E-02
FAM35A	1.87E-02	1.886354554	1.076752863	3.304689149	2.65E-02
FAM26F	5.31E-03	1.504583964	1.134251553	1.995829672	4.60E-03
FAM25A	1.35E-03	9.33768E+12	18389.21195	4.74E+21	3.50E-03
FAM24B	1.82E-05	0.080113874	0.016257902	0.394776202	1.92E-03
FAM227B	1.27E-02	0.01838882	0.001205928	0.280405439	4.04E-03
FAM225A	1.55E-03	1660.029646	2.716047578	1014598.731	2.35E-02
FAM222A	7.05E-03	0.451149343	0.211937928	0.960355383	3.89E-02
FAM213B	2.79E-03	0.439582654	0.227606943	0.848976344	1.44E-02
FAM213A	3.08E-03	0.3033288	0.119571723	0.769482606	1.20E-02
FAM212A	4.85E-03	0.489920199	0.267363516	0.897735807	2.09E-02
FAM205CP	4.05E-02	1.05E-30	3.57E-55	3.06E-06	1.63E-02
FAM189A2	9.66E-06	0.098343169	0.041446785	0.233344485	1.43E-07
FAM188B	5.87E-03	357.4959582	3.792446111	33699.45318	1.13E-02
FAM186B	9.15E-03	0.004620398	5.09E-05	0.419323227	1.94E-02
FAM181B	1.93E-02	0.196105822	0.05694631	0.675328984	9.82E-03
FAM177A1	3.78E-02	4.217807478	1.312667279	13.55248219	1.57E-02
FAM173B	5.02E-05	3.791069325	1.95527885	7.350463912	7.99E-05
FAM172BP	3.66E-02	487574.9627	4.386325756	54197831515	2.71E-02

FAM163A	1.04E-03	2.833183935	1.480553228	5.421575568	1.66E-03
FAM157A	1.71E-02	3.49E-27	2.24E-50	0.000541632	2.54E-02
FAM151A	2.07E-02	0.004985699	2.73E-05	0.910722326	4.60E-02
FAM149A	4.60E-02	0.35760595	0.133361861	0.958909947	4.10E-02
FAM134B	2.81E-02	1.933719021	1.172468583	3.189227673	9.79E-03
FAM132B	2.52E-02	3.29438408	1.896037035	5.724026622	2.34E-05
FAM132A	3.61E-03	0.149632255	0.041074862	0.545097669	3.98E-03
FAM131A	3.03E-02	0.20938511	0.050084913	0.875355902	3.22E-02
FAM129A	6.60E-04	0.611234505	0.440766393	0.84763182	3.17E-03
FAM127C	2.44E-04	0.2059581	0.096778324	0.43830826	4.12E-05
FAM127B	2.82E-02	0.425199553	0.185426096	0.975022738	4.34E-02
FAM124B	1.60E-02	89.17655677	3.566017939	2230.066818	6.26E-03
FAM120C	6.92E-04	4.595391211	1.722745068	12.25812268	2.32E-03
FAM110B	1.11E-02	2.678024488	1.232526266	5.818792955	1.28E-02
FAM110A	1.14E-02	6.717503199	2.477863978	18.21118901	1.82E-04
FAM105A	2.82E-04	5.500321792	2.436063358	12.41902831	4.08E-05
FAIM3	2.61E-02	1.898670917	1.147415998	3.141799712	1.26E-02
FAHD2CP	1.11E-02	0.428789986	0.222479978	0.826415273	1.14E-02
FAHD2B	1.84E-03	0.313900886	0.127493396	0.772853884	1.17E-02
FAHD2A	2.53E-02	0.28422045	0.095463183	0.846203344	2.38E-02
FAF2	2.40E-03	5.139933002	1.786573314	14.78747671	2.40E-03
FADS3	1.29E-03	3.15620433	1.490691906	6.682551726	2.67E-03
FADS1	3.66E-04	4.601125566	2.228984088	9.497760255	3.67E-05
FABP5P7	3.01E-03	6.950594039	2.747875502	17.58113039	4.22E-05
FABP5P11	2.18E-04	830.5063761	13.3760557	51565.33858	1.42E-03
FABP5P1	9.90E-05	5369.322413	181.5702462	158779.4464	6.69E-07
FABP5	2.46E-06	2.67353739	1.724188773	4.145603015	1.11E-05
FABP3	3.28E-06	2.281173597	1.469222252	3.541841933	2.39E-04
FAAHP1	4.72E-02	72.50972889	4.456549382	1179.760468	2.61E-03
F7	4.98E-02	2.163164455	1.245645533	3.75651045	6.14E-03
F2RL3	3.16E-05	84.94010839	9.985311238	722.5435284	4.77E-05
F2R	4.70E-02	2.780359942	1.511178509	5.115478655	1.01E-03
F12	4.31E-03	4.817293591	2.061003366	11.25971841	2.84E-04

EZR	1.74E-02	1.631343106	1.001441795	2.657448832	4.93E-02
EYA4	1.83E-03	79.40659963	7.079020644	890.7175698	3.90E-04
EXTL3	1.21E-02	0.232479367	0.095137914	0.568087463	1.37E-03
EXTL1	7.56E-03	0.625379898	0.475657751	0.822229883	7.74E-04
EXT2	4.62E-02	0.222953152	0.078723785	0.631424259	4.72E-03
EXT1	1.64E-02	3.058131879	1.331999451	7.021151983	8.39E-03
EXPH5	8.86E-03	5.36E-11	3.56E-18	0.000807055	5.04E-03
EXOSC7	1.75E-02	0.138069695	0.024010341	0.793959584	2.65E-02
EXOSC4	1.44E-02	3.060667518	1.683605617	5.564061773	2.44E-04
EXOC3L4	6.04E-03	40.02588911	1.526947373	1049.199094	2.68E-02
EXOC3	1.43E-03	2.021327567	1.210782323	3.374483632	7.11E-03
EXOC2	1.81E-02	0.454410225	0.219121698	0.942346897	3.40E-02
EVI2B	6.54E-04	1.861254659	1.230513324	2.815303856	3.26E-03
EVA1B	4.27E-03	2.603604578	1.38821188	4.883085137	2.86E-03
ETV7	5.61E-03	1.534881781	1.140258123	2.066077878	4.72E-03
ETV5	2.06E-02	0.503035308	0.269089554	0.940372886	3.14E-02
ETS2	2.75E-02	2.436531845	1.313877493	4.518448229	4.71E-03
ETNPPL	6.69E-05	7.090865988	1.364074604	36.86043293	1.99E-02
ETFB	1.54E-02	2.533649743	1.016281272	6.316539719	4.61E-02
ESRRG	1.30E-02	0.01834922	0.000354215	0.950534151	4.71E-02
ESD	2.12E-04	0.322481521	0.145125717	0.716580998	5.47E-03
ERVMER34-1	2.18E-04	4.332170375	2.199432268	8.532974819	2.25E-05
ERVFRD-1	1.71E-04	0.002285206	2.96E-05	0.176611775	6.11E-03
ERP44	4.11E-04	2.773543306	1.209353271	6.360872921	1.60E-02
ERN2	2.72E-05	3.89384E+11	29258.00724	5.18216E+18	1.43E-03
ERICH6	6.45E-03	1.54E-06	5.68E-12	0.415970885	3.60E-02
ERICH4	5.07E-03	1449.116644	4.998340761	420127.2276	1.19E-02
ERH	1.28E-03	17.64168935	3.718916926	83.68813002	3.02E-04
EREG	3.40E-04	6.72E+98	1.53E+54	2.95E+143	1.43E-05
ERBB3	6.35E-07	0.604756713	0.446610068	0.818903801	1.15E-03
EPYC	3.93E-02	2430.232106	1.870863504	3156846.066	3.31E-02
EPX	3.18E-03	2.76E-07	1.67E-11	0.004548105	2.30E-03
EPSTI1	3.85E-03	1.854368619	1.159194587	2.966441541	9.99E-03

EPS8L2	3.77E-02	0.42951635	0.258197248	0.714509144	1.14E-03
EPPK1	9.78E-04	2.01E-05	6.27E-09	0.06464269	8.67E-03
EPM2A	7.93E-03	0.367976833	0.169051087	0.800982429	1.18E-02
EPHX2	4.98E-03	0.348229258	0.192562698	0.629735754	4.83E-04
EPHA8	2.90E-03	2.182726896	1.095533514	4.34883702	2.65E-02
EPHA5	3.06E-02	4.263754359	1.188024073	15.30238457	2.61E-02
EPDR1	1.87E-02	1.871001884	1.186428191	2.950577269	7.03E-03
EPB41L4A-AS1	5.06E-03	0.518201033	0.305939257	0.877730804	1.45E-02
EPB41L1	3.37E-03	1.875308684	1.092068611	3.220294607	2.27E-02
EOMES	7.13E-04	2.020592729	1.28124994	3.186571837	2.48E-03
ENY2	1.62E-03	2.971102319	1.603150695	5.506312672	5.42E-04
ENTPD6	8.99E-05	0.153599777	0.064767277	0.364271783	2.12E-05
ENTPD5	2.03E-02	4.635999542	1.535525516	13.9968314	6.51E-03
ENTPD3-AS1	1.12E-06	0.05831255	0.014051423	0.24199354	9.08E-05
ENTPD1	1.58E-02	0.437064865	0.242328748	0.788291515	5.95E-03
ENTHD1	2.88E-03	200.4852276	18.13386423	2216.533993	1.54E-05
ENSA	1.85E-03	7.471556537	1.84817915	30.20494906	4.78E-03
ENPP7P2	4.02E-03	0.001244372	2.76E-06	0.561591528	3.20E-02
ENPP2	1.31E-06	0.406330979	0.271636142	0.607816263	1.17E-05
ENPP1	8.43E-03	0.323673256	0.122517526	0.855097061	2.29E-02
ENOX1-AS1	4.01E-02	6.18E+39	2230125.321	1.71E+73	1.97E-02
ENOX1	3.28E-02	7.839211284	2.530787083	24.28226142	3.58E-04
ENOSF1	2.57E-02	2.134419987	1.087391789	4.189611074	2.76E-02
ENKD1	1.26E-02	0.315963315	0.149808565	0.666402596	2.48E-03
ENDOV	3.27E-02	0.13464113	0.0289808	0.62552565	1.05E-02
EMR4P	1.76E-03	63.12026228	4.529019932	879.6974995	2.04E-03
EMR1	1.48E-03	6.54652245	1.069225233	40.08225289	4.21E-02
EMP3	1.17E-05	2.884305758	1.702154492	4.887464531	8.26E-05
EMILIN3	7.31E-03	4.22129567	1.995186279	8.931164637	1.66E-04
EMILIN2	5.88E-03	4.766783509	1.55125738	14.64761768	6.40E-03
EMID1	1.19E-04	3.382839201	1.926166421	5.941127897	2.22E-05
EMG1	7.48E-03	9.519258995	1.848187677	49.02981064	7.05E-03
EMC9	1.96E-02	2.0798752	1.028786211	4.204839449	4.15E-02

EMC7	1.05E-05	4.845692499	2.309246605	10.16813697	3.00E-05
EMC4	1.75E-02	5.763989423	1.892266008	17.55756005	2.05E-03
EMC3-AS1	7.31E-03	0.01882121	0.001303013	0.271860633	3.55E-03
EMC2	4.83E-02	1.98264077	1.26899448	3.097621372	2.64E-03
EMB	3.87E-02	3.195935524	1.509111934	6.768221526	2.41E-03
ELMO1	3.44E-04	2.160415066	1.058864499	4.407923075	3.42E-02
ELL2	4.63E-02	0.268579708	0.104894937	0.687688667	6.13E-03
ELFN1-AS1	4.17E-05	1.669834817	1.252069227	2.226992132	4.83E-04
ELFN1	2.78E-04	2.566143439	1.438612533	4.577391063	1.41E-03
ELF4	7.91E-03	2.964452672	1.373188216	6.399690546	5.65E-03
EIF5AP4	1.03E-02	7.160695621	1.943071771	26.38891808	3.10E-03
EIF5AP2	3.58E-03	54.92271538	1.887128417	1598.462848	1.98E-02
EIF5A	2.06E-04	6.718054791	2.19317364	20.57851661	8.53E-04
EIF4G1	3.07E-03	0.235088448	0.099022926	0.558119021	1.03E-03
EIF4EBP3	4.45E-03	2.957431356	1.299380082	6.73121002	9.76E-03
EIF4E2	5.30E-05	15.06310634	3.986531576	56.91593513	6.36E-05
EIF4A2P3	2.63E-02	8.00E-06	1.06E-10	0.603842953	4.06E-02
EIF4A2	9.95E-03	0.440006398	0.268510352	0.721036001	1.12E-03
EIF4A1P7	4.68E-03	1066.78452	5.311445473	214259.794	9.96E-03
EIF4A1P5	3.84E-03	27149.00054	23.24031499	31715070.6	4.61E-03
EIF4A1P10	4.83E-02	8.298219964	2.762887024	24.92336964	1.63E-04
EIF3M	4.79E-02	0.46941359	0.231145719	0.953290936	3.64E-02
EIF3LP3	3.04E-03	0.002907195	1.88E-05	0.450250435	2.32E-02
EIF3L	3.73E-03	0.311513482	0.177207167	0.547611315	5.07E-05
EIF3KP1	2.11E-02	2591.424547	1.198604464	5602750.02	4.48E-02
EIF3J	2.91E-02	1.794815111	1.013310139	3.179047716	4.49E-02
EIF3C	2.03E-02	0.329081002	0.127003146	0.852689948	2.21E-02
EIF2D	7.18E-03	0.383046663	0.166062641	0.8835506	2.44E-02
EIF2B5	3.06E-05	0.109413643	0.034480597	0.347190783	1.73E-04
EIF2B2	3.07E-02	5.393510046	1.250906671	23.25509272	2.38E-02
EIF2AK1	4.20E-02	5.207578447	1.188216162	22.82318162	2.86E-02
EIF1B-AS1	2.02E-05	6.44E-07	1.39E-10	0.002974302	9.29E-04
EIF1B	3.85E-06	0.203182593	0.095526537	0.432164371	3.49E-05

EIF1	4.34E-02	0.205412655	0.043450185	0.971097334	4.58E-02
EHMT2	3.91E-02	0.300722488	0.126295099	0.71605324	6.64E-03
EHHADH	8.67E-04	0.217480503	0.081728687	0.578716864	2.25E-03
EHD3	4.43E-02	1.93725461	1.186597608	3.162786945	8.19E-03
EHD2	3.36E-05	2.900049101	1.5962896	5.268645981	4.74E-04
EGR3	8.32E-03	2.685369612	1.533984199	4.700967558	5.45E-04
EGR2	3.08E-02	2.355918176	1.210951703	4.583461449	1.16E-02
EGFLAM-AS1	7.23E-05	3.2784E+14	10755.6091	9.99E+24	6.65E-03
EFS	9.46E-08	0.320836008	0.191568023	0.537332602	1.56E-05
EFNA3	1.15E-03	8.514846273	2.832882714	25.59322583	1.36E-04
EFHD2	4.70E-04	3.289444415	1.697579987	6.374041071	4.19E-04
EFEMP2	8.76E-03	0.534487638	0.336907839	0.847938226	7.80E-03
EFCAB6-AS1	2.48E-03	5.92E-39	2.22E-64	1.58E-13	3.21E-03
EFCAB12	1.41E-05	6.54E-11	1.47E-17	0.000289903	2.67E-03
EFCAB10	3.84E-02	2813.708439	15.34578419	515904.2433	2.82E-03
EFCAB1	2.29E-03	0.2636426	0.096040619	0.723729412	9.67E-03
EEPD1	9.72E-05	7.481171619	1.936588693	28.90026623	3.52E-03
EEFSEC	3.05E-03	0.189497991	0.080531954	0.445903608	1.39E-04
EEF1DP3	1.20E-02	60190.89179	15.56198625	232807264.9	9.02E-03
EEF1A2	4.38E-04	1.454718717	1.191533321	1.776036396	2.32E-04
EEF1A1P8	3.38E-02	0.097963071	0.009806375	0.978624996	4.79E-02
EEF1A1P32	4.66E-02	1.16E-16	2.24E-31	0.060422603	3.38E-02
EEF1A1P10	1.05E-03	0.093036706	0.012890882	0.671469051	1.85E-02
EEF1A1	7.32E-03	0.55832566	0.363154088	0.858389187	7.91E-03
EDNRB	1.24E-04	0.431669118	0.296680759	0.628076549	1.13E-05
EDNRA	4.91E-02	4.125649242	1.859052942	9.155727245	4.93E-04
EDEM2	2.67E-02	2.791854327	1.002899251	7.77191784	4.94E-02
ECSCR	2.36E-04	6.482479213	2.080972319	20.1937029	1.26E-03
ECM1	5.78E-04	1.661471061	1.312467878	2.103278969	2.44E-05
ECHS1	4.17E-02	11.04506228	2.21861916	54.9861837	3.36E-03
ECHDC3	4.35E-05	0.114346248	0.039673006	0.329570797	5.94E-05
ECH1	2.14E-02	4.302301026	1.742275872	10.62391692	1.56E-03
ECE1	7.11E-04	2.273154623	1.344473793	3.843311761	2.18E-03

ECD	2.33E-02	4.75826594	1.267483225	17.86303307	2.08E-02
EBI3	2.51E-02	1.911414646	1.181535131	3.0921687	8.30E-03
E2F8	1.52E-02	171.9216067	9.284016639	3183.64777	5.48E-04
E2F5	4.07E-02	3.740544045	1.813126015	7.716876619	3.56E-04
DYSF	1.87E-03	2.049392874	1.391702143	3.017895154	2.79E-04
DYRK3	2.29E-02	3.991657722	1.065223528	14.95773512	4.00E-02
DYRK2	3.15E-02	2.698602782	1.189599081	6.121774208	1.75E-02
DYNLT3P2	2.65E-02	271.4586058	1.053288121	69961.64985	4.79E-02
DYNLRB2	2.28E-02	0.068894584	0.010483996	0.452734214	5.35E-03
DYNLL1P3	4.61E-02	270.644453	1.692527831	43277.52761	3.05E-02
DYNLL1-AS1	4.99E-02	0.342399502	0.128633225	0.911408535	3.19E-02
DYNLL1	5.97E-04	9.300772618	2.571548249	33.63902324	6.74E-04
DVL3	2.47E-03	0.295273829	0.156664991	0.556516383	1.62E-04
DVL2	3.19E-02	0.269513287	0.11308695	0.642314711	3.09E-03
DUXAP4	1.40E-03	5.57866E+13	7809.162637	3.99E+23	6.25E-03
DUTP1	2.18E-03	8.36E-11	1.40E-19	0.050102807	2.44E-02
DUSP9	3.63E-02	1.997808352	1.064519056	3.749334677	3.12E-02
DUSP8	2.73E-03	0.359252897	0.184595185	0.699165822	2.58E-03
DUSP7	1.87E-03	0.1260391	0.039908361	0.398058307	4.16E-04
DUSP5P1	7.22E-03	55.79856359	2.874713441	1083.057412	7.87E-03
DUSP5	4.17E-03	2.363734772	1.355838656	4.120875333	2.42E-03
DUSP22	3.59E-04	0.129875504	0.042413855	0.397691905	3.50E-04
DUSP2	3.63E-02	2.5366179	1.289469127	4.989984046	7.01E-03
DUSP15	3.12E-04	0.37393902	0.188353425	0.742383053	4.93E-03
DUSP14	1.06E-05	3.787249885	1.911416989	7.503994042	1.35E-04
DUSP13	2.75E-02	231.0117758	1.945437493	27431.5884	2.55E-02
DUOX1	1.65E-02	0.273790448	0.086604724	0.865555662	2.74E-02
DTYMK	6.08E-06	14.59041285	4.090212009	52.04623784	3.62E-05
DTWD1	3.15E-04	0.079140556	0.016534985	0.378786411	1.50E-03
DSTNP2	1.21E-02	2.221526828	1.0645764	4.635817071	3.34E-02
DSN1	1.19E-02	3.321742002	1.478062268	7.465159059	3.66E-03
DSCR10	1.99E-03	3.36E+44	48416042.41	2.34E+81	1.78E-02
DSCC1	2.95E-02	3.410532568	1.418581544	8.199551477	6.12E-03

DRG2	5.69E-03	6.600408218	1.893461463	23.00833129	3.06E-03
DRG1	4.84E-03	4.987646656	1.260179755	19.7405323	2.21E-02
DPYS	7.87E-04	7.113086182	1.397403055	36.20715931	1.81E-02
DPYD-AS2	4.59E-02	1.25E+22	2.58913605	6.04E+43	4.58E-02
DPYD	1.70E-02	2.326100736	1.211201697	4.46725318	1.12E-02
DPRXP6	2.59E-02	3246033.913	1.149290497	9.16804E+12	4.79E-02
DPRXP2	6.11E-04	9271841.296	242.1907862	3.54956E+11	2.89E-03
DPPA3P2	2.76E-06	2.34E+71	1.44E+23	3.79E+119	3.71E-03
DPP7	4.18E-02	2.198463544	1.104697303	4.375173127	2.49E-02
DPH1	1.43E-02	0.3514545	0.134270744	0.919934324	3.32E-02
DPF2	3.94E-02	0.144984225	0.026105135	0.805221877	2.73E-02
DPEP1	8.10E-04	28.38469043	5.069935866	158.9153536	1.41E-04
DPCD	4.66E-03	0.188845719	0.066997197	0.532301457	1.62E-03
DOLK	5.47E-03	3.289466673	1.175653605	9.203893861	2.33E-02
DOK7	2.74E-02	12.4620842	2.97904218	52.13203887	5.50E-04
DOK5	9.74E-03	1.492826904	1.059741822	2.102901026	2.19E-02
DOK4	4.38E-02	4.919561819	1.573952153	15.37663547	6.14E-03
DOK2	1.75E-02	1.921402464	1.256899053	2.937218721	2.56E-03
DOK1	4.80E-03	10.51788829	2.71271505	40.78053608	6.66E-04
DOCK4	1.07E-02	3.622331888	1.440049232	9.111694249	6.24E-03
DOCK11	1.24E-03	4.097708125	1.811055415	9.271506402	7.10E-04
DOCK10	4.64E-04	2.441058438	1.543861455	3.859650927	1.35E-04
DNTTIP1	1.81E-03	3.432667861	1.419203301	8.302692531	6.20E-03
DNM2	1.85E-02	6.199903957	1.249692947	30.75860287	2.56E-02
DNASE1L3	2.78E-02	1.96315425	1.238666446	3.111390174	4.09E-03
DNALI1	6.14E-05	0.221891295	0.090239357	0.545612781	1.04E-03
DNAL4	8.56E-04	0.047626395	0.009349424	0.24261104	2.47E-04
DNAJC8P1	6.08E-03	2095.370804	6.847935141	641153.6787	8.82E-03
DNAJC3	1.04E-02	2.276674007	1.355496196	3.82387243	1.87E-03
DNAJC19P1	1.99E-03	5.74721E+16	780.8318418	4.23E+30	1.78E-02
DNAJC17	1.90E-02	21.89109004	4.489781868	106.7356583	1.35E-04
DNAJB12	1.34E-02	12.02774671	1.736197329	83.32387602	1.18E-02
DNAJA1P2	2.19E-03	2.14E+20	224978.2905	2.04E+35	7.81E-03

DNAJA1	3.45E-02	1.88349154	1.020520528	3.476206783	4.29E-02
DNAI1	1.95E-05	4.19E-09	1.00E-15	0.017529467	1.31E-02
DNAH9	1.16E-02	7.47E-18	5.92E-35	0.942069784	4.97E-02
DNAH1	3.15E-02	0.067530244	0.01128502	0.404105067	3.15E-03
DNAAF5	1.02E-03	7.021458582	2.298425938	21.44984522	6.25E-04
DNAAF3	3.44E-02	0.014941462	0.000257525	0.866894012	4.25E-02
DNAAF1	2.89E-05	1.12E-05	1.08E-08	0.01156884	1.29E-03
DMRT2	2.79E-04	0.124582928	0.02983482	0.520227899	4.29E-03
DMP1	9.68E-04	4.46105E+19	2419.316298	8.23E+35	1.79E-02
DMD	3.60E-02	0.381244394	0.145928095	0.996019909	4.91E-02
DLX2	9.74E-04	1552.632289	3.125619946	771260.4435	2.04E-02
DLST	8.38E-04	6.47735404	1.868135554	22.45881744	3.23E-03
DLL4	5.01E-07	2.724019236	1.751594141	4.236301451	8.67E-06
DLL3	4.71E-03	0.481649544	0.293917446	0.78929062	3.74E-03
DLGAP5	4.47E-03	5.546736989	2.299570795	13.37914505	1.37E-04
DLGAP3	3.53E-04	0.275999162	0.088537491	0.860376059	2.65E-02
DLG1-AS1	3.00E-02	1.39E-14	5.00E-28	0.384477064	4.33E-02
DLEU7-AS1	2.34E-02	89623.61512	17.34412021	463119045	8.95E-03
DLEU2	4.86E-02	39.67486978	2.549403047	617.4368128	8.58E-03
DLEU1	2.45E-02	7.434158426	1.46178321	37.80773449	1.56E-02
DLC1	1.15E-04	0.287120228	0.153639152	0.536569127	9.18E-05
DKKL1	1.58E-02	0.084018839	0.014895265	0.473920101	5.02E-03
DKFZP434A062	2.52E-05	1.65E-05	4.79E-09	0.056652158	8.03E-03
DIXDC1	1.26E-03	0.28912339	0.100461593	0.832082509	2.14E-02
DISP1	4.92E-03	0.130438116	0.038071872	0.44689429	1.19E-03
DISC1	1.43E-02	3.999635568	1.538750554	10.39615202	4.45E-03
DIRAS1	8.20E-03	3.34264257	1.536765529	7.27063377	2.34E-03
DIP2A	2.14E-02	2.549673675	1.145535756	5.67493054	2.19E-02
DIAPH3-AS2	1.99E-03	1.67233E+19	2078.749625	1.35E+35	1.78E-02
DIAPH2	1.02E-02	4.94984346	1.914947839	12.79457841	9.64E-04
DHX34	1.44E-02	0.164198645	0.035683715	0.755560202	2.03E-02
DHX30	1.90E-05	0.078064275	0.022073127	0.276083719	7.59E-05
DHRS7B	1.60E-06	4.620295169	2.167694549	9.847848471	7.38E-05

DHRS3	1.63E-02	1.544026086	1.038843021	2.294876614	3.17E-02
DGKK	1.77E-02	2.64E+52	1.77947E+19	3.91E+85	1.95E-03
DGKH	3.59E-02	5.971661791	1.493929496	23.8704334	1.15E-02
DGAT2	8.29E-04	9.172967638	2.580249705	32.61053964	6.15E-04
DGAT1	4.66E-02	2.364123963	1.26778734	4.408532831	6.80E-03
DET1	7.33E-04	0.329237272	0.143376485	0.756031795	8.81E-03
DERL3	4.09E-02	2.568508005	1.545093841	4.26979462	2.75E-04
DERL2	7.95E-03	14.61329594	3.717959582	57.43699297	1.23E-04
DERL1	2.92E-06	2.610265114	1.603977775	4.247866819	1.13E-04
DEPDC5	2.20E-02	6.014721615	1.088319802	33.24103452	3.97E-02
DEPDC1	4.98E-02	9.042801699	2.136101633	38.28107301	2.78E-03
DENND3	2.51E-04	3.578080652	1.957627091	6.53988761	3.43E-05
DENND2C	1.21E-02	0.000766934	6.17E-06	0.09535302	3.56E-03
DENND1A	2.78E-02	0.127492334	0.034479519	0.471418854	2.02E-03
DECR1	1.54E-05	3.425630666	1.935233597	6.063839258	2.38E-05
DDX56	1.04E-02	5.452927804	1.981567341	15.00550651	1.02E-03
DDX39B-AS1	2.11E-03	0.053857061	0.00292155	0.992823239	4.94E-02
DDX39A	1.70E-06	5.53639016	2.60254628	11.77754887	8.85E-06
DDX27	2.09E-03	6.309795406	1.917139075	20.76715174	2.44E-03
DDX25	3.21E-02	0.036191145	0.002924983	0.447797161	9.71E-03
DDX11-AS1	2.00E-03	0.011733942	0.000588086	0.234124566	3.61E-03
DDX11	1.14E-02	0.29593223	0.146609718	0.597340244	6.79E-04
DDRGK1	1.88E-05	5.009522748	2.24862374	11.16030117	8.06E-05
DDR1-AS1	2.66E-02	7.31E+41	152551651.7	3.51E+75	1.48E-02
DDR1	1.89E-03	0.4307364	0.254637155	0.728620481	1.69E-03
DDAH2	6.63E-03	0.482678775	0.277435015	0.839759897	9.94E-03
DCUN1D3	1.30E-02	3.535781244	1.176382913	10.62727864	2.45E-02
DCTN3	5.67E-04	0.086381577	0.024544712	0.304007514	1.36E-04
DCSTAMP	6.73E-04	1.440083119	1.010937867	2.051401434	4.34E-02
DCPS	3.29E-03	5.01040352	1.751336949	14.33427385	2.66E-03
DCLRE1CP1	9.83E-03	17.69691424	1.934587797	161.8850145	1.10E-02
DCDC1	1.16E-02	1.45E-21	9.69E-40	0.002160656	2.46E-02
DCAF8L2	5.96E-03	3.55E+73	1.17898E+16	1.07E+131	1.21E-02

DCAF13P2	4.42E-04	0.022401094	0.00177611	0.282532558	3.31E-03
DCAF13	5.19E-04	2.329015312	1.404140096	3.863084845	1.06E-03
DCAF12L1	1.07E-02	0.152604193	0.031605819	0.736827607	1.93E-02
DCAF11	1.89E-03	0.238836308	0.085230579	0.669276012	6.45E-03
DBH-AS1	7.48E-03	0.353789582	0.125332494	0.998680106	4.97E-02
DBF4P1	4.52E-02	1613492826	41.18550763	6.32106E+16	1.75E-02
DAZL	2.10E-03	1.07E+117	2.07E+31	5.52E+202	7.45E-03
DAXX	3.07E-03	0.160683097	0.056496306	0.457004351	6.07E-04
DARS-AS1	8.98E-03	13.80975736	1.116376819	170.8288769	4.08E-02
DAP	1.67E-04	4.029657017	1.846904537	8.792081752	4.63E-04
DALRD3	4.80E-04	0.186089228	0.079275052	0.436823438	1.12E-04
DAK	3.95E-03	3.11674894	1.343939139	7.228098114	8.08E-03
DAGLB	7.18E-05	5.996672836	1.93110316	18.62152466	1.95E-03
DAD1	5.16E-03	5.734949275	2.030400609	16.19859797	9.78E-04
DAB2	7.31E-03	0.320677322	0.129505093	0.79405329	1.40E-02
D4S234E	5.16E-03	0.406787467	0.249791237	0.662457359	3.00E-04
CYYR1	6.33E-03	3.416161794	1.036739471	11.25659988	4.35E-02
CYTIP	1.34E-02	3.394316585	1.726138641	6.674657994	3.97E-04
CYTH4	1.10E-02	2.17469209	1.210848824	3.905760647	9.31E-03
CYSLTR2	9.29E-03	1.47747946	1.185194401	1.841845989	5.19E-04
CYP51A1P2	1.52E-02	2173465.826	147.5423567	32017610422	2.88E-03
CYP4V2	1.76E-02	1.897812967	1.175800785	3.063183918	8.72E-03
CYP4F27P	7.23E-05	2.73E+21	898752.4535	8.28E+36	6.65E-03
CYP39A1	3.04E-03	0.020841891	0.000860444	0.50483724	1.73E-02
CYP2T1P	2.36E-02	0.354710449	0.156016418	0.806450404	1.34E-02
CYP2S1	7.03E-03	34.22669925	5.388719682	217.3924439	1.80E-04
CYP2F2P	2.67E-02	62.5296469	2.692449948	1452.192916	9.96E-03
CYP2B7P	2.53E-04	0.000234291	8.69E-07	0.063159411	3.42E-03
CYP2AC1P	4.33E-04	1.98E+25	6974709096	5.60E+40	1.33E-03
CYP27C1	2.32E-02	0.013029371	0.000454544	0.373483311	1.12E-02
CYGB	2.93E-03	2.285139833	1.48454023	3.517495822	1.73E-04
CYCSP49	2.76E-06	1.16596E+19	1537400.104	8.84E+31	3.71E-03
CYCSP28	7.23E-05	1.23329E+14	8197.962673	1.86E+24	6.65E-03

CYCSP25	1.45E-02	239950737.2	3.891447224	1.47956E+16	3.50E-02
CYCSP10	1.86E-03	0.029470485	0.002059271	0.421755775	9.44E-03
CYCS	6.85E-03	3.352260528	1.489103252	7.546589282	3.48E-03
CYC1	1.83E-06	4.038001017	2.139483799	7.621208545	1.66E-05
CYBA	2.73E-02	2.667624232	1.509441099	4.714472825	7.33E-04
CYB5D2	9.30E-03	3.368551395	1.425453115	7.960373007	5.64E-03
CYB5B	2.70E-02	3.682639891	1.069377604	12.68199046	3.88E-02
CYB561A3	4.48E-03	0.145957718	0.055234459	0.385695011	1.04E-04
CXXC5	1.89E-03	1.566107575	1.026863144	2.388529525	3.72E-02
CXorf65	5.04E-04	4.660878652	1.87443508	11.58951305	9.27E-04
CXorf49	4.59E-02	4.63E+39	5.685664078	3.77E+78	4.57E-02
CXorf40A	9.00E-03	0.181475547	0.040366688	0.815855247	2.61E-02
CXCR6	1.34E-02	2.000158997	1.221970869	3.27392094	5.83E-03
CXCR3	7.40E-04	1.72904656	1.256384226	2.379528448	7.77E-04
CXCR2P1	2.99E-03	1.619774959	1.146149822	2.289116891	6.28E-03
CXCL9	1.23E-05	1.439152431	1.164438898	1.77867617	7.55E-04
CXCL8	9.90E-03	3.952736745	1.502939587	10.39571245	5.34E-03
CXCL3	4.92E-02	11046.58809	17.67675383	6903253.263	4.59E-03
CXCL16	1.72E-02	1.90204513	1.077931865	3.356219252	2.65E-02
CXCL11	4.11E-03	2.470384208	1.345991611	4.53405362	3.51E-03
CXCL10	1.77E-03	1.464325272	1.152964176	1.859770275	1.77E-03
CUZD1	4.04E-03	0.001871328	9.58E-06	0.365641489	1.96E-02
CUX1	1.77E-03	3.269267141	1.132644337	9.436419969	2.85E-02
CUTA	3.48E-03	0.359539432	0.182932557	0.706646237	3.01E-03
CUL9	3.01E-02	0.202933448	0.062849758	0.65524491	7.66E-03
CUL1	1.52E-02	2.321658195	1.133771569	4.754129419	2.13E-02
CTSW	6.01E-04	1.721806826	1.262883954	2.347498942	5.91E-04
CTSS	1.81E-03	1.516565807	1.13796671	2.021124016	4.48E-03
CTSLP8	1.50E-02	0.000735115	5.41E-07	0.999214087	5.00E-02
CTSL	8.07E-03	0.43736104	0.243222988	0.78645806	5.74E-03
CTSK	1.11E-02	2.036856581	1.304832474	3.179553554	1.74E-03
CTSH	2.97E-04	3.93446272	1.964150991	7.881266239	1.11E-04
CTSF	2.40E-02	0.162703657	0.07439459	0.355838776	5.42E-06

CTSC	6.81E-06	2.639553481	1.628205794	4.27909212	8.23E-05
CTSA	3.41E-02	2.836157168	1.45951035	5.511291839	2.10E-03
CTNNBIP1	5.63E-05	0.165208806	0.067071624	0.40693736	9.05E-05
CTNNB1	2.21E-03	0.408513406	0.260561719	0.640474753	9.54E-05
CTHRC1P1	4.59E-03	15206.89703	8.684933774	26626537.8	1.15E-02
CTH	1.47E-02	0.357376996	0.172942893	0.738499949	5.46E-03
CTF1	3.47E-05	0.293312316	0.17165402	0.501194874	7.22E-06
CTDSP2	3.08E-03	0.098926361	0.035078592	0.278985679	1.22E-05
CTD-3236F5.1	1.57E-02	189.7044047	10.11825453	3556.716334	4.52E-04
CTD-3224K15.2	1.98E-02	308766.3832	8.261725927	11539559680	1.86E-02
CTD-3224I3.3	1.13E-02	10960148.31	84.20578151	1.42656E+12	6.98E-03
CTD-3203P2.1	7.81E-03	0.048063563	0.003278193	0.704688937	2.67E-02
CTD-3193K9.3	1.87E-02	48.87891512	5.15558914	463.4093755	7.01E-04
CTD-3162L10.5	7.38E-05	3.70E-08	1.68E-13	0.008173033	6.42E-03
CTD-3148I10.9	1.14E-06	7.44E+25	2.43162E+11	2.28E+40	4.64E-04
CTD-3131K8.1	1.53E-02	7.49E-09	1.38E-15	0.040701873	1.80E-02
CTD-3116E22.6	2.90E-05	1.09E+26	423797189.5	2.82E+43	3.38E-03
CTD-3093B17.2	1.06E-02	1771942.893	2.479251364	1.26642E+12	3.64E-02
CTD-3088G3.6	4.82E-02	4.81E+23	1251741752	1.85E+38	1.46E-03
CTD-3076O17.2	1.66E-02	3.72E-09	1.25E-15	0.011048419	1.07E-02
CTD-3074O7.2	2.42E-04	9.33E-10	1.99E-16	0.004373764	7.98E-03
CTD-3066C23.1	5.28E-04	2.79377E+11	385.0405675	2.03E+20	1.13E-02
CTD-3065J16.9	1.38E-02	2.62971101	1.440507707	4.800654633	1.64E-03
CTD-3064M3.7	2.74E-02	2.294139781	1.346488734	3.908742198	2.26E-03
CTD-3064M3.3	2.07E-03	8.739321629	2.60698302	29.29660145	4.44E-04
CTD-3064H18.4	2.37E-04	0.054528538	0.008813472	0.337365512	1.76E-03
CTD-3051D23.1	4.61E-02	3.64E-05	1.33E-08	0.099425032	1.13E-02
CTD-3035K23.7	1.19E-04	45.80315007	5.936126859	353.417069	2.44E-04
CTD-3035K23.3	2.03E-02	18637.27795	19.96886066	17394489.12	4.83E-03
CTD-3022G6.1	1.06E-02	2.16547E+13	6.943299743	6.75E+25	3.64E-02
CTD-2666L21.1	2.99E-02	0.139690757	0.025783235	0.756829304	2.24E-02
CTD-2655K5.1	1.97E-04	13.05752335	2.91522043	58.48577153	7.84E-04
CTD-2651B20.7	1.29E-02	0.116872851	0.014615036	0.934603484	4.30E-02

CTD-2630F21.1	1.66E-03	0.069130969	0.012095423	0.395115633	2.66E-03
CTD-2623N2.5	2.93E-02	7.03E-08	2.70E-14	0.182932347	2.89E-02
CTD-2619J13.9	2.94E-06	0.139745538	0.049450813	0.39491394	2.05E-04
CTD-2619J13.3	7.32E-04	0.047771387	0.00742116	0.307513294	1.37E-03
CTD-2619J13.13	4.78E-02	0.008473354	0.000109373	0.656449558	3.16E-02
CTD-2616J11.9	1.60E-02	13.42845347	1.27924311	140.9609801	3.04E-02
CTD-2591A6.2	5.28E-04	4.46E+22	130637.9924	1.52E+40	1.13E-02
CTD-2588J6.1	1.26E-02	1.95E+22	662.5795844	5.75E+41	2.48E-02
CTD-2588E21.1	1.49E-02	0.06508168	0.007206049	0.587787441	1.50E-02
CTD-2587H24.5	2.71E-02	4.08128E+12	5373.630227	3.10E+21	5.38E-03
CTD-2582D11.1	1.88E-02	1.64918E+16	1.235932139	2.20E+32	4.87E-02
CTD-2571L23.6	2.96E-04	0.078564756	0.018549135	0.332760582	5.52E-04
CTD-2571E19.3	4.11E-02	1.39E-16	8.46E-32	0.228814161	4.11E-02
CTD-2562J17.6	1.58E-02	1.945271395	1.134532223	3.335366525	1.56E-02
CTD-2555I5.1	5.28E-04	1.04E+26	753556.5982	1.44E+46	1.13E-02
CTD-2554C21.3	6.06E-07	0.007086206	0.000420635	0.11937739	5.92E-04
CTD-2554C21.2	4.47E-05	0.118346046	0.031413312	0.445855141	1.61E-03
CTD-2554C21.1	2.59E-04	0.000990332	2.95E-05	0.03329294	1.15E-04
CTD-2553L13.9	2.41E-04	0.01140154	0.001556204	0.083533493	1.07E-05
CTD-2552K11.2	1.95E-04	73.97685536	10.76440997	508.3952714	1.21E-05
CTD-2547L24.3	4.56E-02	9.48942892	1.380292586	65.23925589	2.22E-02
CTD-2545G14.4	3.80E-02	5.236256603	1.600875577	17.12711694	6.18E-03
CTD-2541J13.1	2.24E-02	9571839941	9129.130421	1.0036E+16	1.16E-03
CTD-2540M10.1	2.79E-02	0.059675404	0.00605328	0.588301542	1.58E-02
CTD-2540B15.6	8.28E-03	23908.5325	7.397405442	77272758.75	1.45E-02
CTD-2538C1.3	7.89E-03	41.2761961	1.317238454	1293.406186	3.43E-02
CTD-2538C1.2	7.12E-04	3.768803695	1.671696388	8.496687195	1.38E-03
CTD-2534J5.1	2.76E-06	4.06E+32	38083372559	4.32E+54	3.71E-03
CTD-2531D15.4	1.67E-02	174127.5192	14.73742169	2057374321	1.17E-02
CTD-2530N21.5	2.99E-05	2.84E+61	1.73E+33	4.68E+89	1.97E-05
CTD-2527I21.9	4.06E-02	11.39455301	2.254312779	57.59442057	3.25E-03
CTD-2527I21.7	4.02E-02	13760.79795	8.692847433	21783375.57	1.12E-02
CTD-2527I21.5	7.22E-03	1.28E-05	1.66E-10	0.983241112	4.97E-02

CTD-2525I3.2	2.30E-02	0.000556464	2.90E-06	0.106687368	5.20E-03
CTD-2523D13.2	3.33E-07	63.34401918	12.17287503	329.6234255	8.23E-07
CTD-2522B17.4	1.99E-03	8.56E+38	5241476.467	1.40E+71	1.78E-02
CTD-2521M24.6	2.18E-02	0.017146688	0.001080081	0.272210029	3.95E-03
CTD-2520I13.1	1.51E-05	59424738531	13643.72958	2.58822E+17	1.47E-03
CTD-2516K3.3	1.77E-02	215412539.9	2.728199677	1.70085E+16	3.86E-02
CTD-2509G16.5	2.41E-02	5.71219E+18	1416192.468	2.30E+31	3.54E-03
CTD-2509G16.1	2.00E-02	51123046.11	18.10331161	1.44369E+14	1.92E-02
CTD-2396E7.7	8.12E-03	1.0448E+11	361.7272979	3.01774E+19	1.07E-02
CTD-2396E7.11	2.33E-03	0.070479338	0.010730548	0.462915506	5.75E-03
CTD-2380F24.1	4.38E-02	0.542382334	0.319307649	0.921301438	2.36E-02
CTD-2377O17.1	4.41E-03	0.000171529	5.35E-08	0.549702627	3.53E-02
CTD-2377D24.6	3.19E-03	1.61E+23	33906306628	7.64E+35	3.33E-04
CTD-2376I20.1	2.79E-02	58188606.48	6.124123942	5.52881E+14	2.92E-02
CTD-2369P2.10	2.14E-02	7.18E-05	6.24E-08	0.08260054	7.97E-03
CTD-2358C21.5	2.65E-02	4.93E-05	1.58E-08	0.153456388	1.57E-02
CTD-2341M24.1	1.14E-03	2.700201009	1.474806317	4.943757973	1.29E-03
CTD-2339F6.1	5.83E-04	402019.8631	1.607161344	1.00562E+11	4.19E-02
CTD-2337A12.1	9.22E-04	4.19E-07	1.10E-11	0.015892146	6.34E-03
CTD-2336H13.1	3.07E-02	2.67199E+19	376645887	1.90E+30	4.50E-04
CTD-2325P2.4	5.21E-03	2.979586994	1.758125521	5.049661445	4.99E-05
CTD-2325A15.3	4.63E-02	103.3492838	2.598773972	4110.043646	1.36E-02
CTD-2323K18.3	1.43E-04	2807849.841	138.7487596	56822279017	3.34E-03
CTD-2319I12.4	2.99E-02	0.097402677	0.011311562	0.838724281	3.40E-02
CTD-2319I12.2	2.30E-02	0.060389366	0.004496293	0.81108487	3.42E-02
CTD-2315A10.2	5.28E-04	1.94E+41	2120185825	1.78E+73	1.13E-02
CTD-2313F11.1	1.78E-03	14018.58203	1.536049127	127939034.4	4.01E-02
CTD-2309O5.3	3.02E-02	1892554674	1.538637714	2.32788E+18	4.55E-02
CTD-2301A4.1	8.32E-03	243.7914237	13.42183248	4428.177625	2.03E-04
CTD-2291D10.4	4.61E-02	0.05075263	0.00302317	0.85202933	3.83E-02
CTD-2288F12.1	1.26E-02	45.05450416	2.672582397	759.5306876	8.24E-03
CTD-2282P23.2	2.76E-06	3.48E+23	43546184.27	2.79E+39	3.71E-03
CTD-2278I10.4	8.92E-04	0.012787415	0.000923241	0.177113017	1.15E-03

CTD-2245F17.3	6.33E-03	0.192404313	0.058133357	0.636801689	6.95E-03
CTD-2233C11.2	2.60E-02	9.27E+26	3.812215621	2.25E+53	4.52E-02
CTD-2231H16.1	5.89E-05	3.13014447	1.977777117	4.953947702	1.11E-06
CTD-2231E14.4	5.28E-04	5.60091E+17	10210.33428	3.07E+31	1.13E-02
CTD-2228K2.1	2.07E-02	18.3507031	4.071178454	82.71519119	1.52E-04
CTD-2227I18.1	2.69E-07	0.068779674	0.016696828	0.283325884	2.11E-04
CTD-2218G20.2	1.88E-02	8.40E+61	112824954.7	6.26E+115	2.43E-02
CTD-2215E18.1	6.77E-04	1.99881E+17	48312495.31	8.27E+26	4.22E-04
CTD-2210P24.1	5.16E-03	6.78E+63	35854437.68	1.28E+120	2.62E-02
CTD-2207O23.10	3.53E-02	1.80E-13	9.85E-26	0.327779888	4.16E-02
CTD-2199O4.6	1.90E-02	25.324064	3.585387504	178.8671983	1.19E-03
CTD-2199O4.3	6.02E-05	2.60E+48	3.41039E+16	1.98E+80	2.92E-03
CTD-2196E14.8	2.24E-02	1.15566E+16	9.566399964	1.40E+31	3.69E-02
CTD-2195M15.1	2.64E-02	0.085051037	0.014511945	0.498463765	6.30E-03
CTD-2194D22.3	4.18E-02	254199390.5	1.242815782	5.19927E+16	4.75E-02
CTD-2169H9.1	1.28E-03	7244067256	319.2395606	1.6438E+17	8.61E-03
CTD-2162K18.4	3.94E-07	1.17E-09	1.36E-15	0.001006501	3.18E-03
CTD-2161E19.1	2.07E-02	0.096220389	0.018783874	0.49288892	4.97E-03
CTD-2154B17.2	2.76E-06	3.88864E+19	2272623.769	6.65E+32	3.71E-03
CTD-2141G3.1	5.28E-04	2.12E+68	2.71654E+15	1.66E+121	1.13E-02
CTD-2132N18.4	1.67E-02	0.261717004	0.090479024	0.757035025	1.34E-02
CTD-2132N18.2	9.26E-03	0.207725045	0.073722568	0.585298313	2.95E-03
CTD-2130F23.1	2.52E-02	0.00064934	6.38E-07	0.661158759	3.78E-02
CTD-2091N23.1	1.48E-03	2.757E+18	356134.7178	2.13E+31	5.04E-03
CTD-2081C10.5	5.16E-03	3402077.007	5.929894255	1.95183E+12	2.62E-02
CTD-2074D8.1	1.88E-02	6.29096E+18	1.27831604	3.10E+37	4.87E-02
CTD-2057J6.2	1.99E-03	7.02E+27	63983.0541	7.70E+50	1.78E-02
CTD-2054N24.2	4.21E-04	0.18442419	0.051858011	0.655873243	9.01E-03
CTD-2033A16.3	9.52E-03	18.67989867	4.365966186	79.92242714	7.91E-05
CTD-2028E8.1	2.99E-08	9.82E+33	1.48814E+17	6.48E+50	7.46E-05
CTD-2026K11.5	1.54E-03	14.93883419	2.623295564	85.07191114	2.31E-03
CTD-2024P10.2	1.61E-02	5.975439369	1.26328807	28.26423879	2.41E-02
CTD-2024P10.1	8.16E-03	7.280296998	1.068936145	49.58455619	4.26E-02

CTD-2024I7.18	2.94E-04	32.20938454	3.671060865	282.6007223	1.73E-03
CTD-2023N9.1	2.17E-02	0.02151633	0.000653698	0.70820587	3.13E-02
CTD-2021J15.1	5.52E-03	2.82E-05	4.91E-09	0.162100143	1.77E-02
CTD-2020K17.1	4.67E-02	4.489882676	1.461674848	13.79174477	8.72E-03
CTD-2017C7.2	2.53E-03	0.304801297	0.128904157	0.720720209	6.81E-03
CTD-2014B16.5	1.47E-03	24.64316469	2.020186324	300.6086909	1.20E-02
CTD-2014B16.2	4.33E-02	248.8952898	3.381369426	18320.64394	1.19E-02
CTD-2006C1.2	3.95E-02	0.046161681	0.004811567	0.442870423	7.68E-03
CTD-2006C1.10	3.53E-02	2589274.425	1.604162016	4.17934E+12	4.29E-02
CTD-2005H7.1	2.39E-02	1.75E-07	3.73E-14	0.825516734	4.72E-02
CTCFL	1.45E-04	7.87E+110	1.90E+45	3.25E+176	9.25E-04
CTC-575N7.1	5.05E-04	3.30868311	1.638402854	6.68174124	8.47E-04
CTC-571O20.1	1.99E-03	1.20E+31	231282.7693	6.24E+56	1.78E-02
CTC-559E9.9	1.80E-03	9.29E-19	1.39E-32	6.23E-05	1.06E-02
CTC-559E9.12	2.11E-03	9.01E-06	5.09E-09	0.015942345	2.33E-03
CTC-559E9.1	2.12E-02	0.116487857	0.022399647	0.605787251	1.06E-02
CTC-548K16.1	1.83E-05	9.006372561	3.092075093	26.23311021	5.59E-05
CTC-543D15.8	1.88E-02	0.24921458	0.096082269	0.646403416	4.27E-03
CTC-526N19.1	1.99E-04	2.871802441	1.277138304	6.457600745	1.07E-02
CTC-523E23.5	2.30E-07	2.10E-11	4.28E-17	1.03E-05	2.36E-04
CTC-523E23.4	1.87E-06	5.13E-07	1.08E-10	0.002433323	7.98E-04
CTC-523E23.11	7.79E-08	0.150806124	0.059404903	0.382838548	6.89E-05
CTC-523E23.1	1.26E-06	0.000462546	5.36E-06	0.039917321	7.35E-04
CTC-498J12.1	1.32E-05	0.028863457	0.002412254	0.345361338	5.12E-03
CTC-479C5.12	2.04E-02	23.43491776	2.99948077	183.0968133	2.64E-03
CTC-444N24.13	2.11E-02	0.019200268	0.000643429	0.572946223	2.25E-02
CTC-436P18.5	7.03E-04	10.9682126	2.216694302	54.27076142	3.33E-03
CTC-428G20.1	3.59E-02	27.52641539	2.057042419	368.346096	1.22E-02
CTC-422A18.1	4.27E-02	6852.538621	1.530873295	30673528.44	3.95E-02
CTC-360J11.4	1.49E-02	7.74E-05	7.40E-08	0.080948722	7.62E-03
CTC-360G5.9	4.13E-02	0.000357175	1.16E-06	0.110438765	6.67E-03
CTC-359M8.3	5.28E-04	6.67E+22	143088.5698	3.11E+40	1.13E-02
CTC-338M12.5	2.63E-04	0.370732069	0.165773101	0.82909873	1.57E-02

CTC-321K16.4	2.76E-06	5.48E+62	2.28E+20	1.32E+105	3.71E-03
CTC-311G1.1	1.88E-02	2.45E+29	1.467970078	4.10E+58	4.87E-02
CTC-308K20.1	3.32E-03	0.009586708	0.000447	0.205603851	2.97E-03
CTC-277H1.7	1.44E-04	0.003485182	0.000104206	0.116562509	1.58E-03
CTC-264K15.6	2.76E-06	5.04E+29	4343899798	5.85E+49	3.71E-03
CTC-260E6.6	5.30E-04	2.41E-07	1.36E-12	0.042956775	1.35E-02
CTC-260E6.4	4.74E-03	0.034678269	0.0025932	0.463744534	1.11E-02
CTC-258N23.3	7.23E-05	605830311.5	274.9435691	1.33493E+15	6.65E-03
CTC-255N20.1	9.94E-04	3.598740687	1.816758081	7.128596083	2.41E-04
CTC-251I16.1	1.36E-03	1.764798869	1.193951821	2.608576824	4.38E-03
CTC-250P20.1	2.43E-02	1.52E-13	5.53E-25	0.041689382	2.81E-02
CTC-236F12.4	2.59E-02	0.000385536	4.66E-07	0.318767203	2.18E-02
CTBP1	3.09E-02	7.473476533	1.624344888	34.38484765	9.80E-03
CTB-95D12.1	1.54E-02	2.45E+34	5.64314E+12	1.06E+56	1.84E-03
CTB-60B18.6	3.14E-04	2.08189E+12	5306.520183	8.17E+20	4.96E-03
CTB-60B18.10	4.71E-03	3.579720331	1.697824041	7.547541642	8.06E-04
CTB-57H20.1	2.76E-06	2.97E+48	5.35218E+15	1.65E+81	3.71E-03
CTB-54O9.9	3.04E-03	0.075988619	0.008799716	0.656188232	1.91E-02
CTB-50L17.2	4.61E-03	4.82E-05	6.25E-09	0.371951317	2.95E-02
CTB-49A3.2	9.70E-04	125.536512	11.87535594	1327.068925	5.90E-05
CTB-39G8.2	4.78E-03	0.006045537	4.63E-05	0.789384746	3.99E-02
CTB-37A13.1	3.21E-02	3819490.752	3.113803488	4.68511E+12	3.41E-02
CTB-33G10.1	4.51E-02	0.361499197	0.134469546	0.971830969	4.37E-02
CTB-32O4.4	3.90E-04	7.20E-11	2.87E-18	0.00180711	7.22E-03
CTB-32O4.3	6.12E-04	6.18E-05	1.77E-07	0.021535908	1.17E-03
CTB-32O4.2	5.63E-03	0.072355435	0.007439062	0.703759315	2.37E-02
CTB-30L5.1	3.67E-03	0.005231245	6.91E-05	0.396317826	1.74E-02
CTB-25B13.12	1.28E-02	0.277107459	0.109562639	0.700864319	6.71E-03
CTB-20D2.1	2.60E-02	1454543.811	2.973552082	7.11505E+11	3.38E-02
CTB-193M12.5	3.53E-02	3.215423409	1.084855387	9.53025428	3.51E-02
CTB-191K22.6	6.66E-05	0.000168107	6.99E-07	0.040414642	1.89E-03
CTB-179K24.3	2.83E-04	5.862514982	1.79452632	19.15217488	3.41E-03
CTB-152G17.6	2.61E-02	7.025731253	1.675450053	29.46127791	7.69E-03

CTB-147N14.4	2.01E-02	940934.6415	1.466639339	6.03664E+11	4.38E-02
CTB-129O4.1	1.17E-02	0.292835572	0.116417001	0.736599222	9.07E-03
CTB-113P19.1	3.41E-02	9294517783	7639.143353	1.13086E+16	1.32E-03
CTB-105L4.2	2.12E-07	42790758990	21391.34827	8.55976E+16	9.43E-04
CTA-992D9.9	5.16E-03	1.66E+59	10206929.65	2.71E+111	2.62E-02
CTA-797E19.1	1.33E-02	757246.5538	88.48139638	6480710824	3.39E-03
CTA-481E9.4	8.70E-04	479.7187263	1.618674689	142171.9002	3.35E-02
CTA-397C4.2	4.46E-02	2991.975085	4.3840336	2041935.743	1.62E-02
CTA-384D8.35	1.30E-02	9.94627161	2.725236666	36.30081753	5.06E-04
CTA-384D8.31	1.54E-03	6.022647482	1.668485398	21.73964648	6.11E-03
CTA-342B11.2	7.49E-03	2088572385	8.734969904	4.99387E+17	2.92E-02
CTA-292E10.6	1.50E-02	80.5542471	7.510552861	863.9825651	2.88E-04
CTA-256D12.11	1.72E-02	762.4026627	1.303312207	445985.0964	4.12E-02
CTA-250D10.19	1.72E-02	296.8067521	5.464379428	16121.54669	5.22E-03
CTA-228A9.3	2.61E-02	1.837434922	1.037623264	3.253750385	3.69E-02
CTA-126B4.7	1.91E-04	69.23274864	1.32820321	3608.765171	3.57E-02
CTA-113A6.2	2.76E-06	1.83E+22	16751704.16	2.01E+37	3.71E-03
CSTB	1.36E-04	3.700613896	1.927639717	7.104306415	8.42E-05
CST7	3.77E-05	1.616790549	1.267533459	2.062282193	1.09E-04
CSPG4	1.84E-02	1.720312595	1.095965818	2.700335517	1.84E-02
CSNK2B-LY6G5B-562	9.73E-03	5.88E-07	6.65E-12	0.0520058	1.36E-02
CSNK2A2	2.68E-03	0.306218859	0.134163659	0.698922419	4.94E-03
CSN1S2AP	8.59E-03	2.623592545	1.287837566	5.344802811	7.89E-03
CSF3R	1.16E-02	4.473436529	1.102714116	18.14761786	3.60E-02
CSF3	2.88E-03	29444729.7	237.1397764	3.65604E+12	4.06E-03
CSF2RB	5.19E-03	3.689746856	1.341154871	10.15112584	1.15E-02
CSF2RA	6.20E-03	2.527902846	1.450279141	4.406250229	1.07E-03
CSF2	2.28E-02	9306.462555	4.669378077	18548561.25	1.84E-02
CRY2	7.26E-06	0.073663841	0.024996608	0.217083917	2.25E-06
CRTAP	3.32E-05	0.226216288	0.097109738	0.526968873	5.72E-04
CRTAM	4.25E-04	2.995799727	1.261671328	7.11343422	1.29E-02
CRLS1	9.61E-03	4.033661489	1.465210755	11.10449466	6.95E-03
CRIP2	3.28E-04	2.145847817	1.377637104	3.342435277	7.33E-04

CRIP1	4.49E-03	8.797398566	2.16874287	35.68621371	2.34E-03
CRHR1-IT1	2.22E-03	0.110725755	0.033873733	0.361938044	2.71E-04
CRHR1	8.26E-05	2.405589044	1.007827673	5.741912835	4.80E-02
CREM	2.51E-02	2.485995981	1.27056884	4.864101667	7.83E-03
CRELD2	2.27E-05	9.77135499	3.019763994	31.61815907	1.42E-04
CRELD1	6.84E-04	0.473744751	0.284037748	0.790155851	4.21E-03
CREB3L4	3.58E-03	0.142780048	0.045434736	0.448690674	8.63E-04
CRB3	1.48E-03	0.018608744	0.000829031	0.417698923	1.21E-02
CRACR2A	8.94E-04	4.597965657	1.443604085	14.64479659	9.85E-03
CRABP2	2.22E-03	2.317316769	1.617840093	3.319213704	4.56E-06
CPVL	9.19E-06	2.555387602	1.510665972	4.322600705	4.68E-04
CPSF2	1.27E-02	2.880895426	1.2428803	6.677681235	1.36E-02
CPS1	8.33E-04	0.356566275	0.165963526	0.766068978	8.22E-03
CPNE7	9.14E-03	3.461505016	1.846706841	6.48831569	1.07E-04
CPNE5	1.28E-03	1.738943484	1.130404918	2.675080753	1.18E-02
CPLX1	2.32E-03	1.641084753	1.180598711	2.281180846	3.20E-03
CPEB4	1.66E-02	1.937713514	1.21771223	3.083432661	5.25E-03
CPB2-AS1	1.91E-03	0.093662943	0.010731551	0.817472451	3.22E-02
CPA2	3.74E-02	0.001495922	4.86E-06	0.460310545	2.61E-02
COX7A2L	1.00E-02	0.204880774	0.060425455	0.694676301	1.09E-02
COX7A1	2.68E-04	1.745948449	1.231567988	2.475166631	1.75E-03
COX6C	4.11E-07	4.158652144	2.336451555	7.401988547	1.27E-06
COX6B1	4.27E-02	5.362012783	1.731813806	16.60177381	3.59E-03
COX6A2	1.67E-05	0.624664425	0.474103919	0.823038216	8.26E-04
COX6A1P2	4.92E-03	4.474419757	1.719660824	11.64208191	2.13E-03
COX6A1	4.77E-05	7.386825956	2.581202564	21.13944813	1.93E-04
COX5BP1	2.07E-02	1.01487E+11	3.69958035	2.78E+21	3.88E-02
COX5B	4.62E-02	6.29389169	2.00901823	19.71762725	1.59E-03
COX5A	2.68E-02	10.1829056	3.292793626	31.49045406	5.60E-05
COX4I2	7.58E-03	2.06162981	1.330195797	3.195257033	1.21E-03
COX16	8.06E-04	17.68108752	3.027678643	103.2543056	1.42E-03
COX14	8.52E-06	29.02666226	4.955305111	170.0293126	1.88E-04
COX11P1	1.82E-02	3.14E-14	6.25E-27	0.157231303	3.72E-02

COTL1	1.06E-03	3.52428287	1.894236441	6.557032419	6.99E-05
CORO7	2.68E-02	4.161579612	1.704507214	10.16055827	1.74E-03
CORO6	3.91E-04	3.327399397	1.897804293	5.833892772	2.71E-05
CORO1C	1.92E-03	3.169617434	1.546430813	6.496556196	1.63E-03
CORO1A	1.61E-03	1.685388561	1.22664429	2.315695451	1.28E-03
COQ6	6.33E-06	22.12068488	4.626444328	105.7669054	1.05E-04
COQ4	4.68E-02	3.844949077	1.135444401	13.02012973	3.05E-02
COQ2	5.22E-05	5.106598388	2.342084381	11.13424747	4.13E-05
COPZ1	1.12E-03	5.04631143	1.250178973	20.3692908	2.30E-02
COPS8	3.81E-03	6.084179639	2.159159071	17.14428658	6.35E-04
COPS7A	3.46E-03	5.609442951	1.18753712	26.49672982	2.95E-02
COPS5	1.64E-02	2.251763396	1.383541804	3.664824854	1.09E-03
COPG1	3.57E-02	0.360948871	0.139612709	0.933182147	3.55E-02
COPA	8.35E-03	7.446373005	2.342921298	23.66638222	6.66E-04
COMTD1	8.89E-04	3.219012215	1.567552401	6.610330625	1.45E-03
COMT	9.28E-04	2.594566297	1.447015785	4.652177493	1.37E-03
COMMD7	2.58E-05	6.121322711	2.546975591	14.71179853	5.13E-05
COMMD6	2.22E-03	0.33029176	0.157785019	0.691400537	3.29E-03
COMMD5	2.17E-03	4.286849834	2.017286096	9.109804274	1.54E-04
COMMD1	4.41E-02	8.889684734	2.160194659	36.58304327	2.47E-03
COLGALT2	1.32E-04	2.229767989	1.359961537	3.655886691	1.48E-03
COLEC11	1.20E-03	0.477451505	0.280177504	0.813626848	6.56E-03
COL9A3	3.00E-05	1.641572014	1.293853932	2.082737944	4.48E-05
COL6A6	2.20E-03	9.15E-17	1.45E-28	5.78E-05	7.73E-03
COL5A3	7.39E-05	2.204105068	1.331842556	3.647637726	2.11E-03
COL5A1-AS1	4.05E-03	213.2872469	3.736176603	12175.93667	9.36E-03
COL5A1	2.32E-02	2.42554475	1.41549947	4.156318997	1.26E-03
COL4A2-AS1	4.78E-02	11232.01516	1.213891995	103928656.8	4.53E-02
COL26A1	4.31E-02	6.164049709	2.407444089	15.78250934	1.50E-04
COL22A1	6.33E-03	1.478613162	1.134740365	1.926693499	3.78E-03
COL18A1-AS2	2.01E-02	530263985	61.00802116	4.6089E+15	1.37E-02
COL18A1-AS1	7.18E-03	3.17E+32	230201.9532	4.36E+59	1.89E-02
COL18A1	1.46E-04	3.164011361	1.876131388	5.335963119	1.56E-05

COL12A1	1.43E-02	2.88572771	1.553418627	5.360708485	7.97E-04
COL11A2	1.39E-03	0.615971426	0.428181069	0.88612231	9.01E-03
COL11A1	7.53E-06	0.151871177	0.058662952	0.393175823	1.03E-04
COBLL1	2.26E-04	0.137281965	0.036903271	0.510695588	3.05E-03
COASY	3.90E-02	3.60001355	1.015745441	12.75919835	4.72E-02
COA6	1.06E-02	2.030343751	1.063402954	3.876513348	3.19E-02
COA4	7.11E-04	7.154942154	2.08644614	24.53607417	1.75E-03
CNTNAP5	1.13E-02	1.963575605	1.109431595	3.475319413	2.05E-02
CNTNAP1	4.37E-05	2.032288595	1.321642682	3.125048086	1.24E-03
CNTN3	3.73E-05	0.286179463	0.089376662	0.916331881	3.51E-02
CNTN1	1.49E-02	0.258752301	0.079476827	0.842418551	2.48E-02
CNTF	1.66E-03	2.395959352	1.355451897	4.235208367	2.64E-03
CNTD2	2.46E-03	0.001072087	1.82E-06	0.631313314	3.56E-02
CNR2	4.98E-03	3930.404229	3.46143487	4462911.475	2.11E-02
CNPY2	3.25E-02	13.43274812	2.623915575	68.76696943	1.82E-03
CNOT4P1	5.16E-03	1.07E+54	2479578.569	4.60E+101	2.62E-02
CNOT10-AS1	4.20E-03	1076354967	56.99182813	2.03282E+16	1.50E-02
CNNM3	4.36E-02	0.455069083	0.216418623	0.95688563	3.79E-02
CNNM2	1.87E-03	4.729142241	1.996037779	11.2045907	4.15E-04
CNN2P1	9.32E-04	7.02E-06	1.59E-10	0.310520098	2.97E-02
CNIH4	3.17E-04	5.4816391	2.344824389	12.81476232	8.61E-05
CNIH3	4.08E-04	10.17359186	2.843132836	36.40419825	3.62E-04
CNBD2	1.67E-02	4065.593096	3.320783809	4977453.568	2.20E-02
CMSS1	3.40E-03	0.195701704	0.069448702	0.551474047	2.03E-03
CMPK2	1.36E-04	1.721546943	1.05960826	2.796999597	2.83E-02
CMB9-94B1.2	1.92E-03	0.194286317	0.065126243	0.579600044	3.30E-03
CMB9-22P13.2	4.18E-03	10.60392799	2.176414295	51.66446897	3.47E-03
CMB9-22P13.1	6.55E-05	1.858840258	1.370860103	2.520524958	6.60E-05
CMA1	1.07E-02	1700.22601	1.003917741	2879487.4	4.99E-02
CLYBL	3.23E-06	13.14774665	3.666260044	47.14974932	7.69E-05
CLUHP3	1.13E-02	0.129058297	0.034740841	0.479436985	2.23E-03
CLSTN2-AS1	1.17E-02	4.77E-31	1.04E-60	0.217839211	4.51E-02
CLPSL2	4.16E-04	2260.907209	77.62112542	65854.51293	7.13E-06

CLPB	2.02E-04	3.37972998	1.645700637	6.940858185	9.11E-04
CLNS1AP1	3.49E-03	2.31792E+11	316.7548231	1.70E+20	1.20E-02
CLN3	2.38E-03	4.278539237	1.546606529	11.83617013	5.11E-03
CLK3P2	2.40E-03	1.75E-40	1.65E-78	0.018521831	4.04E-02
CLIP3	8.51E-04	1.631248649	1.206365292	2.205776453	1.48E-03
CLIP1-AS1	8.33E-03	3.63798E+13	4035.871338	3.28E+23	7.59E-03
CLIC4P3	3.60E-03	3.19953E+16	107773.1563	9.50E+27	4.81E-03
CLIC4P1	4.16E-04	10110738.23	210.1294594	4.86495E+11	3.37E-03
CLECL1	4.83E-02	22.57332649	1.843052784	276.4733995	1.48E-02
CLEC4G	9.67E-05	24.44587133	1.804766667	331.123483	1.62E-02
CLEC4A	2.15E-02	3.058350228	1.208144821	7.742040486	1.83E-02
CLEC1A	3.83E-02	75.72348207	1.925825142	2977.448789	2.09E-02
CLEC14A	1.29E-02	3.005204779	1.397322268	6.463259027	4.86E-03
CLEC12A	1.54E-03	5.839410292	1.811076159	18.82787335	3.13E-03
CLEC11A	6.58E-06	0.434457089	0.284893848	0.662537866	1.08E-04
CLEC10A	2.17E-02	2.457031233	1.143016806	5.281639294	2.13E-02
CLDN7	4.05E-04	0.11176556	0.031115832	0.401452883	7.83E-04
CLDN5	1.67E-02	2.125286106	1.188292381	3.801119239	1.10E-02
CLDN23	2.78E-03	4.174345093	1.720366927	10.12874445	1.58E-03
CLDN18	1.53E-02	11663.36916	2.391834821	56874404.11	3.07E-02
CLDN17	5.28E-04	4.17E+52	7.69247E+11	2.26E+93	1.13E-02
CLDN10-AS1	2.72E-02	0.000986883	1.09E-06	0.891432488	4.63E-02
CLCNKB	1.58E-05	4.387895727	1.609939021	11.95922868	3.84E-03
CLCNKA	1.03E-02	2.270422894	1.381727213	3.730707529	1.21E-03
CLCN3P1	2.77E-02	1.89E-13	1.30E-23	0.002755059	1.41E-02
CKS2	4.97E-04	2.20840063	1.409000417	3.461342725	5.49E-04
CKLF-CMTM1	2.65E-02	467.5880573	10.40844687	21005.88051	1.54E-03
CKLF	4.85E-02	3.69814606	1.425891951	9.591388932	7.15E-03
CIZ1	1.00E-02	0.107382143	0.033378438	0.345460279	1.82E-04
CITF22-1A6.3	8.93E-04	317.0744139	13.84599714	7261.028793	3.12E-04
CITED4	1.17E-04	0.405026613	0.229111825	0.716010871	1.88E-03
CITED1	5.77E-05	3.664006657	1.923861912	6.978122857	7.79E-05
CISD2	1.94E-02	3.483535626	1.453446262	8.349135965	5.14E-03

CIRBP-AS1	3.03E-02	4.301990746	1.240974315	14.9133823	2.14E-02
CIRBP	7.30E-03	0.191589574	0.078500806	0.467594752	2.84E-04
CIR1P1	3.30E-03	8.22E+24	76259852.33	8.86E+41	4.14E-03
CIITA	4.56E-02	2.581174443	1.360631599	4.89659472	3.70E-03
CIDECP	9.31E-06	0.114197159	0.034364966	0.379485063	3.98E-04
CIDEC	1.11E-06	7.59E-09	6.85E-14	0.000841992	1.61E-03
CIB1	4.58E-06	7.826823227	2.988720347	20.49678615	2.80E-05
CHSY3	8.16E-04	1.959446004	1.225126579	3.133903639	4.99E-03
CHST9	2.77E-02	5.807696467	2.602737213	12.95917931	1.74E-05
CHST6	8.58E-03	7.269824428	1.920469404	27.5194945	3.49E-03
CHST3	8.03E-04	4.438106261	1.815109455	10.85156993	1.09E-03
CHST15	1.03E-02	4.935171452	1.744564596	13.96102919	2.62E-03
CHST13	3.65E-03	14.03897458	2.870662643	68.65759988	1.11E-03
CHST12	7.83E-04	11.86979995	1.854013206	75.99306762	9.01E-03
CHST11	1.64E-03	3.945204524	1.837131965	8.472248609	4.32E-04
CHRND	8.26E-03	5.91E-06	3.73E-10	0.093581613	1.47E-02
CHRNB2	5.39E-04	0.13730201	0.036753413	0.512927653	3.15E-03
CHRNA9	7.87E-06	3.37814E+14	6593255.005	1.73E+22	2.21E-04
CHRNA5	5.87E-03	46.38018918	6.887588939	312.3185729	8.04E-05
CHRD	5.02E-04	48.04328999	5.387091308	428.4608486	5.24E-04
CHRAC1	3.83E-04	4.932048184	2.243750804	10.84126599	7.16E-05
chr22-38_28785274-29006793.1	1.18E-02	2.58E+29	2.65489E+14	2.51E+44	1.20E-04
CHPT1	4.34E-05	3.603525956	1.614599243	8.042490649	1.75E-03
CHPF2	6.87E-05	4.266140074	1.921793637	9.470294197	3.63E-04
CHMP6	1.16E-02	0.213345021	0.058960814	0.771972013	1.86E-02
CHMP4B	2.44E-02	3.284730481	1.292715467	8.346348914	1.24E-02
CHMP4A	4.42E-04	9.782500106	2.305871072	41.50158675	1.98E-03
CHMP2A	2.05E-04	10.24099423	3.328324064	31.51074262	4.97E-05
CHL1-AS2	1.21E-05	0.222012773	0.089309691	0.551896114	1.20E-03
CHL1	2.73E-05	0.327822524	0.154273156	0.696606011	3.73E-03
CHKB-CPT1B	4.19E-02	0.042110061	0.002012247	0.881232516	4.12E-02
CHKB	6.23E-03	5.582726193	1.457468472	21.38422364	1.21E-02
CHGB	5.25E-04	0.338039469	0.16171109	0.706634793	3.94E-03

CHEK2P3	9.46E-03	48452499.92	22.76161515	1.03141E+14	1.73E-02
CHEK2	2.51E-02	3.742296375	1.199269611	11.67775955	2.30E-02
CHD7	4.92E-03	3.604778397	1.766533974	7.355888695	4.26E-04
CHCHD7	1.89E-02	3.043685275	1.476280838	6.275242359	2.57E-03
CHCHD4	5.30E-03	0.183329518	0.062293861	0.539534902	2.07E-03
CHCHD2P4	1.63E-02	37.68541069	4.32878725	328.080383	1.01E-03
CHCHD2	1.55E-03	13.50535334	3.070178279	59.40846176	5.73E-04
CHCHD10	2.44E-02	1.714873209	1.091632722	2.693937312	1.93E-02
CHCHD1	1.60E-02	7.594848559	1.76018442	32.77027337	6.57E-03
CHAF1B	3.25E-02	3.219728229	1.0117881	10.24587052	4.77E-02
CHADL	1.73E-02	0.515287794	0.333634197	0.795846208	2.79E-03
CHAD	1.38E-03	0.029486457	0.001545003	0.562750305	1.92E-02
CHAC1	5.74E-07	1.673640813	1.32577627	2.112779987	1.48E-05
CH507-9B2.9	6.59E-03	34.70414119	3.735537294	322.4107594	1.82E-03
CH507-9B2.2	1.71E-03	2.02632E+15	16218.23856	2.53E+26	6.86E-03
CH507-396I9.6	7.79E-05	21171068	1668.273934	2.68669E+11	4.67E-04
CH507-210P18.1	5.28E-04	8.96E+70	1.06411E+16	7.55E+125	1.13E-02
CH17-472G23.2	2.94E-05	4.850709632	2.373153374	9.914818061	1.50E-05
CH17-189H20.1	6.09E-03	4.281978499	2.173483901	8.435921637	2.62E-05
CH17-118O6.2	2.50E-03	3.73705237	1.704040194	8.195558105	1.00E-03
CGREF1	1.22E-03	6.278659889	2.78178846	14.17130403	9.72E-06
CGN	1.96E-04	0.021522529	0.002123359	0.21815401	1.16E-03
CFL1P4	3.50E-02	24286.0403	30.14740711	19564261.41	3.10E-03
CFL1P2	2.42E-02	37.07480821	2.722974179	504.7941382	6.69E-03
CFD	4.90E-02	1.947523261	1.231071227	3.080932094	4.40E-03
CFAP70	1.56E-02	0.042446361	0.002795607	0.644473014	2.28E-02
CFAP57	2.69E-03	7.66E-13	2.06E-22	0.002843817	1.31E-02
CFAP53	1.00E-05	0.015194826	0.001254454	0.184050329	1.00E-03
CFAP46	2.59E-03	5.38E-05	1.02E-07	0.028384716	2.12E-03
CFAP45	8.37E-04	473.6190642	25.37378628	8840.423557	3.70E-05
CFAP43	3.25E-04	4.27E-06	1.57E-09	0.011643258	2.19E-03
CETN4P	2.15E-02	0.043558853	0.003202517	0.592463177	1.86E-02
CETN2	2.72E-03	0.216747952	0.077453892	0.606550215	3.59E-03

CES1P2	1.92E-02	451619660.3	1.342312009	1.51947E+17	4.67E-02
CERS6	4.96E-02	2.737642856	1.278685338	5.861245281	9.52E-03
CERS5	1.53E-03	13.67599495	2.829061506	66.11126604	1.14E-03
CERS2	6.47E-04	8.419243502	2.362886347	29.9987603	1.01E-03
CERS1	2.25E-03	7.92061564	2.72707097	23.00495763	1.42E-04
CEP89	1.82E-02	0.163094577	0.039355135	0.675892512	1.24E-02
CEP19	3.86E-03	0.216302725	0.056678138	0.825483509	2.51E-02
CEP164	2.82E-03	0.105879137	0.027396399	0.409192157	1.13E-03
CENPVP3	1.64E-04	0.002204426	4.02E-05	0.120774623	2.75E-03
CENPV	2.80E-04	0.272082501	0.127790605	0.579298355	7.36E-04
CENPO	4.53E-03	7.15102773	1.891805723	27.03089274	3.74E-03
CENPM	8.76E-04	4.651444733	2.021908879	10.70074835	2.99E-04
CENPL	4.47E-02	6.246169544	1.628552621	23.95663086	7.56E-03
CEMIP	2.23E-02	4.12195589	1.470940682	11.55078553	7.06E-03
CELSR3	9.73E-04	0.000272107	1.04E-06	0.071156091	3.85E-03
CELSR1	1.45E-02	2.231730688	1.434230939	3.472677747	3.73E-04
CELF5	1.63E-06	0.046389413	0.007986653	0.26944674	6.24E-04
CELF2	7.25E-06	2.104048862	1.455640101	3.04128858	7.58E-05
CELA1	4.38E-03	44487.26628	5.16328849	383305497.1	2.06E-02
CECR5-AS1	1.18E-03	0.057200248	0.010703424	0.305684279	8.20E-04
CECR2	1.78E-02	0.120256891	0.020359757	0.710309064	1.94E-02
CEBPD	6.50E-03	2.26328452	1.425983183	3.592228071	5.29E-04
CEBPB	1.95E-05	4.023688341	2.240378304	7.226488418	3.16E-06
CEBPA-AS1	3.05E-06	0.035261938	0.006357048	0.195594583	1.30E-04
CEACAM3	2.66E-03	1.37491E+12	49902.39432	3.78813E+19	1.39E-03
CDYL2	1.40E-02	5.582082138	1.751066386	17.79466572	3.65E-03
CDV3	1.60E-03	0.153714854	0.056347304	0.419332504	2.55E-04
CDRT8	5.16E-03	1.83E+21	328.4489678	1.02E+40	2.62E-02
CDR2	1.23E-02	2.331001455	1.323294158	4.106092173	3.39E-03
CDO1	1.51E-03	0.447425088	0.247457154	0.808985335	7.78E-03
CDKN3	3.93E-03	2.710139067	1.374627275	5.343160213	3.99E-03
CDKN2C	2.99E-02	3.684869375	1.694864413	8.011415076	9.97E-04
CDKN2B-AS1	6.28E-03	913.1354661	4.636119123	179852.2336	1.14E-02

CDKN2B-AS_3	5.28E-04	3624.520425	6.367606578	2063121.857	1.13E-02
CDKN2AIPNLP2	1.99E-03	2.84037E+18	1530.788359	5.27E+33	1.78E-02
CDKN2AIPNLP1	4.30E-03	3174.319818	2.762977878	3646900.827	2.49E-02
CDKN2AIPNL	1.81E-05	9.152321879	3.045365656	27.5057268	8.03E-05
CDKN1A	8.21E-03	1.444342098	1.046960639	1.992552555	2.51E-02
CDK5R1	2.80E-02	1.697209516	1.017995966	2.829598778	4.25E-02
CDK5	2.79E-02	4.198126025	1.640670112	10.74211201	2.76E-03
CDK2AP2P3	7.23E-05	1.80478E+16	32743.18013	9.95E+27	6.65E-03
CDK2	7.85E-04	0.275513659	0.142902074	0.531187365	1.19E-04
CDK15	2.59E-03	0.179252074	0.046447391	0.691778494	1.26E-02
CDK11B	1.42E-02	0.27294236	0.080344015	0.927231879	3.74E-02
CDIP1	5.86E-04	0.397228373	0.196822449	0.801688942	9.97E-03
CDHR4	1.39E-02	1.38479E+11	41.5468159	4.62E+20	2.18E-02
CDHR3	5.74E-03	23.41659194	3.870503651	141.6706526	5.96E-04
CDH6	2.45E-02	11.13699537	1.864761872	66.5139435	8.21E-03
CDH24	1.27E-06	4.389650958	2.177994092	8.847147753	3.52E-05
CDH17	1.06E-02	465.8726023	7.275498896	29831.25757	3.79E-03
CDH1	8.10E-03	1.87321722	1.23993937	2.829930914	2.87E-03
CDCA7L	2.92E-07	0.226652283	0.118199483	0.434614908	7.87E-06
CDCA7	2.51E-02	2.288592332	1.249952803	4.190282104	7.30E-03
CDC6	9.34E-03	5.158617052	1.81098754	14.69437492	2.13E-03
CDC45	4.22E-02	4.735644798	1.658274318	13.52389735	3.68E-03
CDC42SE1	7.03E-03	4.81984891	1.94896642	11.91962225	6.63E-04
CDC42BPG	4.95E-05	0.191432984	0.082621615	0.44354722	1.15E-04
CDC37P1	2.52E-02	0.001053761	4.86E-06	0.228437679	1.25E-02
CDC27	1.96E-02	2.840447646	1.413467193	5.708051004	3.37E-03
CDC25B	1.94E-03	2.499568945	1.641031267	3.80726744	1.98E-05
CDC25A	4.12E-02	0.064360819	0.009086008	0.455900448	6.03E-03
CDC20P1	2.03E-03	0.000850283	9.32E-06	0.077542042	2.14E-03
CDC123	2.58E-02	3.077344608	1.339749921	7.068520542	8.07E-03
CD99	1.51E-02	0.386707303	0.203254448	0.735740546	3.79E-03
CD97	6.62E-04	3.892554962	1.761786649	8.600351321	7.79E-04
CD96	5.25E-05	3.318392784	1.508652865	7.299048659	2.86E-03

CD93	1.94E-02	2.540776019	1.345545312	4.797714888	4.04E-03
CD9	3.14E-02	1.875571168	1.198158049	2.935979282	5.95E-03
CD8B	6.84E-05	1.736741001	1.243473539	2.425680328	1.20E-03
CD8A	1.70E-04	1.511381645	1.182206364	1.932212976	9.82E-04
CD86	1.88E-04	2.317677044	1.312583104	4.092408979	3.76E-03
CD84	5.34E-03	3.576370456	1.466551833	8.721427606	5.08E-03
CD83	7.73E-05	0.326633238	0.167032361	0.638734141	1.08E-03
CD82	1.09E-03	0.494923306	0.331412907	0.739105429	5.87E-04
CD79B	3.17E-04	2.182167156	1.398511044	3.404945224	5.87E-04
CD74	2.43E-04	1.473938015	1.17449108	1.849731605	8.14E-04
CD70	4.93E-03	1.59564432	1.164031784	2.187294909	3.69E-03
CD7	4.93E-05	1.888370785	1.323732932	2.693854731	4.53E-04
CD69	2.69E-02	5.82413549	1.718173198	19.74222055	4.67E-03
CD63	4.98E-02	3.94779384	1.179151097	13.21720027	2.59E-02
CD6	4.70E-05	3.034578648	1.759862917	5.232605042	6.52E-05
CD53	3.96E-04	1.65343568	1.193352607	2.290898375	2.51E-03
CD52	7.48E-03	1.650937655	1.245127776	2.189008383	4.96E-04
CD5	2.25E-03	2.15613839	1.283742604	3.621390099	3.68E-03
CD44	6.66E-03	0.304760194	0.142947413	0.649740862	2.10E-03
CD40LG	4.62E-02	5.682868127	1.484954837	21.74812954	1.12E-02
CD3G	1.60E-03	2.604709377	1.346811016	5.037463207	4.45E-03
CD3E	2.10E-04	1.601087245	1.233990766	2.077390234	3.97E-04
CD3D	1.05E-04	1.50441632	1.196945973	1.890869358	4.63E-04
CD38	1.53E-03	2.426493277	1.302328167	4.52103377	5.24E-03
CD300LF	3.60E-05	3.027172418	1.561337261	5.869182196	1.04E-03
CD300LB	2.76E-02	816.5207477	11.31000321	58948.35919	2.13E-03
CD300C	5.55E-03	2.992482759	1.101339602	8.130964368	3.16E-02
CD300A	5.02E-03	2.252141889	1.415934434	3.582187825	6.06E-04
CD276	3.60E-03	2.017806752	1.086117929	3.748712712	2.63E-02
CD27	3.81E-02	1.653121119	1.248051012	2.189661648	4.57E-04
CD247	3.97E-05	2.291202244	1.349264955	3.890716725	2.15E-03
CD209	2.13E-05	1.866649896	1.292455009	2.695940523	8.75E-04
CD2	4.72E-05	1.522258	1.200107099	1.930885518	5.33E-04

CD180	1.05E-02	12.37535048	2.145600704	71.37828543	4.90E-03
CD163L1	1.15E-03	3.121365382	1.726308384	5.643789915	1.65E-04
CD109	3.47E-02	2.064839068	1.395633777	3.054927766	2.86E-04
CCT7	9.00E-03	4.270961672	1.393301392	13.09200845	1.11E-02
CCT5	3.24E-03	3.93075724	1.4429725	10.70765554	7.42E-03
CCRN4L	2.30E-03	3.192500842	1.641552763	6.208793195	6.25E-04
CCRL2	4.65E-03	8.860153727	2.366365259	33.174221	1.20E-03
CCR5	1.78E-03	1.840680926	1.232473457	2.749029808	2.87E-03
CCR4	2.76E-02	8.219252039	1.312364499	51.47663176	2.44E-02
CCR2	1.71E-02	6.905862071	2.448834101	19.47495378	2.59E-04
CCR1	9.17E-03	2.24499895	1.224893915	4.1146586	8.89E-03
CCNO	1.07E-03	2.274625823	1.483478424	3.487696587	1.64E-04
CCNI	2.62E-03	0.399362503	0.182174018	0.875483844	2.19E-02
CCND3	4.35E-04	0.124457258	0.039867038	0.388531728	3.34E-04
CCNB1IP1	3.26E-04	0.383743974	0.196496744	0.749424312	5.04E-03
CCL5	1.70E-04	1.501563677	1.223803793	1.842365163	9.81E-05
CCL4L1	1.58E-02	2.086019578	1.376693156	3.160818853	5.25E-04
CCL4	2.19E-02	1.854063693	1.326334285	2.591769071	3.03E-04
CCL3	9.33E-05	2.299921331	1.470101441	3.598144986	2.65E-04
CCL28	3.37E-02	0.533860965	0.310821976	0.916947806	2.30E-02
CCL24	2.42E-03	3.313966735	1.968738721	5.578381428	6.50E-06
CCL22	5.63E-05	7.36722251	1.489996501	36.42690938	1.43E-02
CCL20	2.83E-02	166258.1971	331.7836064	83312700.11	1.51E-04
CCL18	2.82E-03	1.457054821	1.140344316	1.861726079	2.61E-03
CCL1	1.87E-02	376232.8999	14.07799998	10054780164	1.36E-02
CCKBR	4.75E-03	9763.135047	14.68729063	6489883.554	5.60E-03
CCHCR1	1.69E-03	0.210025593	0.087868632	0.502007928	4.48E-04
CCDC97	1.25E-02	0.097194679	0.021441366	0.44058786	2.50E-03
CCDC87	4.90E-02	0.082843934	0.007340306	0.934990599	4.40E-02
CCDC8	2.15E-02	0.426642171	0.277075016	0.656946789	1.10E-04
CCDC78	4.61E-02	2.389125234	1.017600172	5.60919656	4.55E-02
CCDC74A	1.56E-04	4.369677058	2.437043031	7.834936578	7.42E-07
CCDC71	1.63E-02	0.298956001	0.126324889	0.707498667	6.01E-03

CCDC57	1.23E-02	0.138521557	0.03538522	0.542266577	4.53E-03
CCDC51	3.79E-05	0.2092561	0.08705345	0.503002643	4.73E-04
CCDC42	2.75E-03	3265565.329	730.3048237	14602008054	4.70E-04
CCDC34	2.74E-02	0.17157952	0.053678369	0.548443111	2.95E-03
CCDC30	4.23E-02	0.001952084	1.07E-05	0.357738882	1.89E-02
CCDC27	7.47E-05	5.30E-09	1.78E-14	0.001579181	3.05E-03
CCDC26	6.20E-04	8.879104967	2.008837851	39.24582812	3.98E-03
CCDC185	4.33E-06	2.70E+22	29119360517	2.50E+34	2.39E-04
CCDC173	8.19E-04	2.03E-09	8.96E-15	0.000457656	1.46E-03
CCDC166	1.26E-02	3.33E-18	8.05E-33	0.001375136	1.91E-02
CCDC162P	2.26E-02	516388.5894	180.4565937	1477680421	1.20E-03
CCDC147-AS1	2.77E-06	0.129353694	0.036638213	0.456691983	1.48E-03
CCDC134	2.35E-03	2.77685924	1.108319634	6.957331625	2.93E-02
CCDC13-AS1	2.66E-02	0.017759532	0.000960357	0.328420631	6.77E-03
CCDC127	1.05E-04	8.300573091	2.398180065	28.72991675	8.36E-04
CCDC120	9.65E-03	0.131704989	0.024691215	0.702525346	1.76E-02
CCDC12	1.76E-03	0.160309084	0.043672393	0.588449605	5.79E-03
CCDC109B	1.28E-03	2.033132116	1.300829835	3.177684037	1.84E-03
CCDC108	6.49E-03	4.44E-17	2.04E-27	9.66E-07	1.93E-03
CCDC107	1.24E-02	5.414359554	1.860529419	15.75642345	1.94E-03
CBX6	4.05E-02	3.916111147	1.103142116	13.90204063	3.47E-02
CBX3P1	4.47E-03	1059937639	138.527894	8.11005E+15	1.02E-02
CBS	3.94E-02	19.34974535	2.814573021	133.0264457	2.60E-03
CBR3	2.92E-02	2.509862382	1.502638425	4.192232189	4.38E-04
CBR1	3.00E-04	3.327731001	1.754659828	6.311077191	2.32E-04
CAST	1.22E-02	2.460569325	1.303564709	4.644496251	5.47E-03
CASP8	3.91E-02	2.604965341	1.209052632	5.612530215	1.45E-02
CASP5	2.44E-03	19.93331361	1.639781833	242.3108875	1.89E-02
CASP1P2	2.74E-02	1523.109413	2.479636251	935565.5619	2.53E-02
CASP12	3.57E-02	8.402468792	1.3293845	53.1083985	2.37E-02
CASP10	6.08E-04	15.41845012	2.98316062	79.69017914	1.10E-03
CASP1	1.57E-02	1.911433839	1.27609575	2.863091834	1.67E-03
CASKIN1	3.62E-02	0.23988834	0.091925728	0.626009899	3.53E-03

CASC11	3.94E-02	1214418.76	5.539667634	2.66228E+11	2.56E-02
CASC1	1.24E-03	0.007911269	0.00025274	0.247639004	5.88E-03
CARD14	8.00E-03	229.8497846	11.22990635	4704.484779	4.15E-04
CARD11	5.30E-08	2.368189639	1.611519188	3.480146067	1.14E-05
CAPN9	8.65E-05	0.01051757	0.000603046	0.183434366	1.79E-03
CAPN5	5.27E-03	2.111275183	1.204529317	3.700601415	9.06E-03
CAPN3	1.87E-02	1.947043248	1.169997703	3.240157993	1.03E-02
CAPN14	7.64E-03	16.29221493	1.806242425	146.9549512	1.29E-02
CAPN11	3.25E-02	96767.49219	212.378807	44090781.36	2.37E-04
CANX	2.89E-02	1.865874951	1.008648003	3.451639541	4.69E-02
CAND2	1.17E-03	0.280669973	0.147184534	0.535216792	1.14E-04
CAMSAP3	2.07E-06	0.259407101	0.136053984	0.494598118	4.17E-05
CAMKMT	6.40E-03	5.129797902	1.544863374	17.03375649	7.58E-03
CAMK1	2.48E-05	18.44934946	4.308532164	79.0010339	8.56E-05
CALU	1.54E-02	2.803319294	1.514259674	5.189730136	1.04E-03
CALM2P3	3.34E-02	401.9175289	1.251349465	129090.7972	4.17E-02
CALM2	6.47E-03	2.84971222	1.425414731	5.697190832	3.05E-03
CALM1	2.19E-04	2.564151023	1.358308093	4.840485381	3.68E-03
CALHM2	3.53E-07	4.117429987	2.152813199	7.874919064	1.89E-05
CALHM1	8.27E-03	3.03341E+17	346524756.1	2.66E+26	1.27E-04
CALCR	2.65E-03	1437.049769	18.58461135	111119.4633	1.05E-03
CALCOCO1	5.01E-05	0.067936281	0.025045013	0.184281728	1.28E-07
CADPS2	2.49E-02	0.576945283	0.350506624	0.949670668	3.05E-02
CADM1	2.02E-05	1.553793964	1.176789346	2.051578467	1.88E-03
CACYBPP2	3.29E-03	795.1800701	3.535655472	178838.5064	1.56E-02
CACTIN	2.79E-02	0.078293925	0.010799183	0.567629842	1.17E-02
CACNB4	1.70E-02	437.3556952	4.031788894	47442.96121	1.10E-02
CACNB1	9.83E-04	3.41943438	1.368299251	8.545302841	8.51E-03
CACNA2D1	1.01E-02	5.961854454	2.180370463	16.30168319	5.04E-04
CABYR	1.89E-03	3.823758463	1.533829749	9.532432653	4.00E-03
CABP7	8.62E-03	1.509855001	1.098701525	2.074869353	1.11E-02
CABLES2	2.77E-02	0.192611745	0.042189837	0.879341725	3.35E-02
CAB39L	4.13E-03	1.840420856	1.000214811	3.386421487	4.99E-02

CA8	3.79E-04	2.036818904	1.402032017	2.959013201	1.89E-04
CA5BP1	4.51E-02	0.276428993	0.088588892	0.862557219	2.68E-02
CA4	5.36E-03	0.210403167	0.08933406	0.495549987	3.62E-04
CA12	1.55E-06	2.012763053	1.591150797	2.546091242	5.45E-09
C9orf9	2.86E-03	0.146734194	0.054445789	0.395456177	1.48E-04
C9orf69	3.00E-02	0.183865914	0.066682401	0.506980462	1.07E-03
C9orf50	1.35E-03	46.01692245	5.217754264	405.8368878	5.66E-04
C9orf40	4.51E-02	2.195913096	1.150172809	4.192443336	1.71E-02
C9orf173	3.18E-02	0.069070735	0.005346225	0.892361628	4.06E-02
C9orf170	3.82E-02	0.000119456	9.13E-08	0.156247924	1.36E-02
C8orf76	5.73E-03	2.705364994	1.481875937	4.939009783	1.19E-03
C8orf59	2.21E-02	2.397083085	1.335632936	4.302085673	3.39E-03
C8orf49	7.05E-04	4.32E-22	1.66E-40	0.001121026	2.30E-02
C8orf37-AS1	5.05E-03	779.4053417	9.08632288	66855.72313	3.37E-03
C8orf33	2.72E-03	2.510245122	1.402708689	4.492258885	1.94E-03
C8G	7.64E-03	2.967278003	1.267164927	6.9483763	1.22E-02
C7orf72	3.13E-04	5.76E+37	16602233589	2.00E+65	7.20E-03
C6orf48	1.51E-04	0.378063574	0.220315536	0.648760719	4.15E-04
C6orf226	2.65E-04	0.382217937	0.215651517	0.677438087	9.89E-04
C5orf58	5.52E-03	45.7625815	7.375940494	283.9249947	4.03E-05
C5orf51	4.76E-02	3.057633213	1.144404498	8.169419885	2.58E-02
C5orf34	1.13E-02	8.021515235	1.784181604	36.06398951	6.63E-03
C5AR2	1.11E-02	1528.828694	6.784658156	344500.3598	7.99E-03
C4orf51	2.82E-02	2.63E-10	9.59E-20	0.718188375	4.66E-02
C4orf48	3.74E-05	4.601816735	2.334078969	9.07283667	1.05E-05
C4orf19	5.78E-03	0.068394049	0.009738202	0.480350047	6.99E-03
C4orf17	0.00E+00	7.79E+213	2.88E+114	Inf	2.49E-05
C4B	1.50E-02	1.794438931	1.197284246	2.689429086	4.62E-03
C4A	7.23E-03	1.571193009	1.06041605	2.327998969	2.43E-02
C3orf80	8.77E-03	2331.149475	5.16191012	1052761.042	1.29E-02
C3orf67	8.54E-03	0.000431744	4.36E-07	0.427370232	2.77E-02
C3orf36	1.81E-02	32488.80398	2.644878113	399081673.7	3.06E-02
C3orf17	4.48E-02	0.479355135	0.258057264	0.890427739	2.00E-02

C3orf14	5.87E-04	0.226311945	0.07977287	0.642036523	5.22E-03
C3AR1	1.80E-02	1.805249529	1.063005533	3.065765662	2.88E-02
C2orf66	4.20E-02	12.62829109	1.640055539	97.23678983	1.49E-02
C2orf42	4.49E-02	4.591415831	1.168060572	18.04795046	2.91E-02
C2orf27B	2.93E-02	7.93E+32	219.783982	2.86E+63	3.48E-02
C2CD4B	3.82E-02	5668.039518	72.6128133	442438.0563	1.01E-04
C2CD2	2.66E-04	2.998704757	1.550510391	5.799529157	1.10E-03
C22orf34	1.21E-03	1255125.296	1105.113754	1425499866	9.14E-05
C22orf23	4.86E-03	0.087965926	0.019509901	0.396619344	1.56E-03
C21orf62-AS1	2.29E-03	0.00112281	2.51E-05	0.050190436	4.60E-04
C20orf24	1.56E-04	4.976882694	2.263408725	10.94338865	6.55E-05
C20orf144	7.57E-03	337.6649989	8.624748569	13219.82323	1.86E-03
C2-AS1	3.64E-02	5.969135815	1.26026188	28.27236382	2.44E-02
C1S	2.67E-02	1.534150915	1.164387346	2.021336833	2.35E-03
C1QTNF6	2.21E-02	2.737968731	1.55898503	4.808559818	4.56E-04
C1QTNF1-AS1	2.51E-02	573219523.2	47123.68323	6.97273E+12	2.64E-05
C1QC	2.79E-04	1.548970101	1.189684379	2.016760423	1.15E-03
C1QBPP	3.46E-02	2.95088E+16	2.725577565	3.19E+32	4.41E-02
C1QBP	1.79E-02	3.679766637	1.271971853	10.64542621	1.62E-02
C1QB	4.08E-05	1.495199746	1.169046257	1.912347153	1.36E-03
C1QA	2.01E-03	1.499083715	1.147839695	1.957809956	2.96E-03
C1orf95	9.49E-04	2.517530822	1.564624882	4.050786555	1.42E-04
C1orf56	6.21E-06	7.208289671	3.093712601	16.79517353	4.72E-06
C1orf229	2.82E-02	0.122440346	0.025574254	0.586200424	8.58E-03
C1orf210	1.27E-02	1.62E-09	2.05E-16	0.012722432	1.25E-02
C1orf21	6.57E-05	3.546285091	1.871040054	6.721469121	1.04E-04
C1orf185	3.78E-08	3.82E+22	2.83633E+11	5.14E+33	6.98E-05
C1orf147	3.06E-03	15018.43164	28.19818475	7998858.466	2.68E-03
C1orf140	1.83E-02	10060596.48	186.0688838	5.43968E+11	3.73E-03
C1orf116	2.95E-04	1.523515376	1.210785604	1.917019078	3.29E-04
C1GALT1C1	2.97E-02	3.398747044	1.423082452	8.117225712	5.88E-03
C19orf81	3.92E-03	0.18036848	0.061425472	0.529630259	1.83E-03
C19orf66	6.65E-04	3.305370306	1.566561525	6.97417413	1.70E-03

C19orf57	3.91E-02	0.097599722	0.013199696	0.721660961	2.26E-02
C19orf53	1.50E-02	3.970516022	1.481165532	10.64364323	6.13E-03
C19orf35	4.01E-02	22.5524006	1.211244945	419.9074469	3.68E-02
C18orf65	1.09E-03	42.1279859	4.652376816	381.4753762	8.76E-04
C17orf96	1.13E-03	5.246470646	2.169307203	12.6885921	2.35E-04
C17orf89	4.60E-02	6.467215339	1.996236499	20.9518633	1.85E-03
C17orf59	7.98E-03	0.171203413	0.051613548	0.567885947	3.92E-03
C17orf100	1.45E-06	0.041741623	0.008867181	0.19649572	5.86E-05
C16orf86	3.18E-06	0.118063944	0.043942542	0.317211846	2.27E-05
C16orf54	3.93E-04	2.806780575	1.405596036	5.604752002	3.45E-03
C16orf47	1.19E-02	20325.31529	14.7341305	28038196.18	7.16E-03
C16orf45	3.35E-04	1.575169202	1.127973502	2.199659842	7.66E-03
C15orf59-AS1	1.36E-02	3.05249281	1.227016218	7.593797228	1.64E-02
C14orf93	4.51E-05	0.112083991	0.034039818	0.369062516	3.19E-04
C14orf80	3.00E-03	6.006120494	1.869901209	19.29165199	2.60E-03
C14orf37	1.05E-02	0.251816788	0.092028677	0.689042777	7.25E-03
C14orf2	4.16E-03	13.47553637	3.054654716	59.44700709	5.93E-04
C14orf169	3.12E-03	20.56004378	3.993022828	105.863507	2.99E-04
C14orf159	4.45E-03	2.79585795	1.224541694	6.38346715	1.47E-02
C14orf119	8.26E-05	9.842718765	2.55642581	37.89631301	8.86E-04
C12orf75	9.55E-06	2.906186255	1.381369116	6.114164891	4.93E-03
C12orf60	7.55E-03	5.882751082	1.239960741	27.90956128	2.57E-02
C12orf57	6.90E-03	3.779268044	1.726241265	8.273969133	8.83E-04
C12orf43	9.00E-04	0.11171461	0.019265658	0.647792764	1.45E-02
C11orf98P1	1.06E-02	4008845014	4.036613702	3.98127E+18	3.64E-02
C11orf96	3.81E-03	0.488364508	0.238703648	0.999146407	4.97E-02
C11orf95	4.09E-04	0.139043502	0.055765618	0.34668486	2.31E-05
C11orf80	1.15E-03	3.611035349	1.647103094	7.916672815	1.35E-03
C11orf73	3.77E-05	7.514365426	1.933962638	29.19688656	3.59E-03
C11orf45	4.03E-04	4.574666928	2.082138438	10.05100195	1.53E-04
C11orf31	1.66E-02	10.79683018	2.184669528	53.35889041	3.52E-03
C10orf90	2.45E-02	2.967636074	1.250690603	7.041600736	1.36E-02
C10orf82	7.38E-03	0.012805316	0.000314078	0.522087314	2.13E-02

C10orf67	3.50E-05	9.11E-09	2.09E-14	0.00398205	5.21E-03
C10orf54	3.43E-05	2.122658407	1.208028075	3.729779801	8.87E-03
C10orf2	4.10E-06	0.243807356	0.1285599	0.462368334	1.54E-05
C10orf128	1.50E-02	4.032371419	1.238314137	13.13077092	2.06E-02
C10orf105	3.59E-06	2.34256E+15	11550996.9	4.75E+23	2.87E-04
BZRAP1	1.74E-02	0.177693853	0.055079256	0.573266735	3.84E-03
BX470102.3	4.58E-02	37.89660931	7.524898827	190.8534626	1.05E-05
BTRC	1.76E-02	0.35055673	0.127833776	0.961326689	4.17E-02
BTN3A3	3.28E-02	1.699429733	1.205944327	2.39485468	2.45E-03
BTLA	4.58E-02	28.98608534	2.359968969	356.018725	8.51E-03
BTG1	4.05E-03	0.278945692	0.148078051	0.525470848	7.77E-05
BTF3	1.51E-02	0.394038325	0.178230642	0.871153244	2.14E-02
BTBD6	6.88E-09	38.80436108	9.585741631	157.0852311	2.92E-07
BTBD16	3.25E-04	196639.3308	46.96353112	823341548.6	4.18E-03
BST1	3.10E-05	6.785928053	2.853217232	16.13926168	1.48E-05
BSN	5.73E-03	0.002718435	3.16E-05	0.233756161	9.34E-03
BSCL2	1.42E-02	0.290524422	0.096547161	0.874230158	2.79E-02
BRPF1	5.47E-09	0.032539035	0.006422409	0.164858529	3.51E-05
BRK1	5.85E-05	0.092861402	0.027741467	0.310842973	1.15E-04
BRI3BP	7.71E-03	2.302694174	1.035160158	5.122299597	4.09E-02
BRD9P2	1.80E-04	19.2288944	5.399622416	68.47708065	5.06E-06
BRD9	4.82E-02	3.880226536	1.178912099	12.77123034	2.57E-02
BRD7P6	1.99E-03	7.08E+95	3.49108E+16	1.43E+175	1.78E-02
BRD3	4.91E-03	0.223801981	0.094012329	0.532774027	7.17E-04
BRAT1	3.20E-02	3.366639353	1.260034721	8.995196991	1.55E-02
BPIFB6	9.46E-03	1.67E+37	72640796797	3.86E+63	5.65E-03
BPGM	1.67E-02	3.38692844	1.327701017	8.639960433	1.07E-02
bP-21201H5.1	1.81E-02	10212156.46	505.9680107	2.06116E+11	1.42E-03
BOLA3-AS1	2.52E-04	0.241728792	0.092870307	0.629187205	3.62E-03
BOLA3	3.71E-04	7.758715882	2.81155224	21.41083181	7.62E-05
BOLA2B	3.87E-04	10.72503631	2.852083391	40.3306594	4.47E-04
BOC	4.84E-02	0.608828889	0.380517956	0.974126476	3.85E-02
BNIPL	3.31E-02	249.731104	1.475573618	42265.34249	3.50E-02

BNIP3P30	1.63E-02	0.01908866	0.000509033	0.715821439	3.23E-02
BNIP3P16	9.84E-03	7.34E-06	2.18E-10	0.246905311	2.62E-02
BNIP3P15	1.18E-02	2.14E-08	3.59E-15	0.128032198	2.65E-02
BNIP1	3.61E-04	15.91017421	4.080592108	62.03355706	6.73E-05
BMS1P12	3.39E-03	5.41E+20	16524.10707	1.77E+37	1.39E-02
BMPR1B	2.21E-02	1.953092854	1.100423244	3.46645867	2.22E-02
BMP1	5.80E-03	3.298394248	1.482198376	7.340046239	3.45E-03
BLOC1S2	4.79E-04	2.238398395	1.29204763	3.877896803	4.06E-03
BLOC1S1	4.26E-03	3.69657585	1.371266316	9.965003042	9.77E-03
BIRC7	2.80E-04	1.731747251	1.11892764	2.680198819	1.37E-02
BIN2	4.49E-05	2.497207992	1.41975378	4.392344533	1.49E-03
BIN1	4.14E-02	2.032258774	1.010494635	4.087182242	4.67E-02
BID	9.99E-03	3.649636403	1.298718431	10.25614603	1.41E-02
BICC1	4.10E-03	6.494248451	1.480695114	28.48342142	1.31E-02
BHLHA15	3.27E-06	22.87750649	6.830810364	76.62052894	3.86E-07
BFSP1	2.70E-02	0.186598011	0.059727211	0.582964062	3.87E-03
BEX5	5.06E-04	0.386131959	0.216466161	0.688781512	1.27E-03
BEX4	1.18E-03	0.457325156	0.272694215	0.766962723	3.02E-03
BEX1	3.19E-03	0.662423076	0.484561648	0.90556967	9.83E-03
BET1P1	1.06E-03	19241.69394	1.820780065	203342947.8	3.69E-02
BEND5	3.40E-02	0.319309938	0.142669569	0.714650203	5.48E-03
BEND3	2.68E-02	0.151485061	0.034131961	0.672323627	1.31E-02
BDKRB1	1.91E-02	13801.19562	44.44443975	4285642.966	1.13E-03
BCS1L	2.04E-03	6.730973897	1.717315931	26.3818723	6.22E-03
BCR	4.81E-02	4.263130701	1.237687953	14.68405936	2.16E-02
BCL9P1	7.72E-04	3.04E+37	28722549272	3.21E+64	6.56E-03
BCL7B	1.03E-02	10.54306187	1.987071997	55.9396709	5.67E-03
BCL3	1.73E-04	2.974100724	1.774055344	4.985907091	3.56E-05
BCL2L13	1.70E-03	4.130561392	1.418450346	12.02829373	9.30E-03
BCL2L1	5.32E-03	2.092668445	1.192016464	3.673826119	1.01E-02
BCL11B	1.31E-04	5.068520144	1.890118738	13.59168391	1.26E-03
BCL11A	1.30E-05	0.05326169	0.006810348	0.416543686	5.20E-03
BCHE	6.11E-04	0.363227956	0.187094993	0.705174123	2.77E-03

BCAT2	3.23E-03	3.791734891	1.055319642	13.62360077	4.11E-02
BCAT1	1.16E-05	2.780385382	1.623580261	4.761417136	1.95E-04
BCAS4	1.76E-04	0.284621941	0.144323748	0.561305055	2.87E-04
BCAS2P2	2.85E-02	111545857.5	4.299278153	2.89409E+15	3.34E-02
BCAP29	2.84E-02	4.014701518	1.881980354	8.564291462	3.23E-04
BBS1	1.32E-02	0.029165039	0.002001013	0.425084423	9.72E-03
BAZ1B	3.70E-02	3.05200805	1.040711235	8.950372425	4.21E-02
BAX	4.27E-06	3.441078916	1.587249654	7.460089268	1.75E-03
BATF3	8.17E-06	10.5635896	3.694222431	30.20647166	1.09E-05
BATF	5.97E-03	1.921077314	1.320836638	2.794091216	6.36E-04
BASP1	9.23E-05	1.483394857	1.070561115	2.055427074	1.78E-02
BARX1-AS1	1.50E-04	5.98E+32	1.56046E+18	2.29E+47	1.06E-05
BARX1	2.55E-03	23.01840012	5.479190623	96.70164454	1.85E-05
BAP1	1.40E-06	0.561778701	0.412394493	0.765275275	2.56E-04
BANCR	1.43E-02	1.268243053	1.050077308	1.531735262	1.36E-02
BAMBI	9.34E-04	0.323161808	0.166676883	0.626562919	8.26E-04
BAIAP2L2	6.98E-04	2.59880243	1.544350228	4.373214019	3.22E-04
BAIAP2-AS1	1.91E-04	0.10912255	0.028604982	0.416281713	1.18E-03
BAI2	1.52E-04	1.755366991	1.113723018	2.766678268	1.54E-02
BAI1	2.27E-04	2.896021989	1.870273517	4.484340545	1.88E-06
BAHCC1	6.70E-03	4.726800716	2.020190221	11.05967388	3.42E-04
BAG2	5.40E-04	2.574244373	1.661281572	3.98892891	2.32E-05
BAALC-AS1	2.14E-05	0.037171343	0.006180148	0.22357213	3.23E-04
B9D2	4.37E-05	9.081194832	2.626881271	31.39391966	4.90E-04
B9D1	4.71E-02	3.308896234	1.109452521	9.868646094	3.19E-02
B4GAT1	1.44E-03	0.300528101	0.140730795	0.6417724	1.90E-03
B4GALT3	3.53E-02	0.227865566	0.054319922	0.955868752	4.32E-02
B4GALT1	2.72E-03	2.139942363	1.079463658	4.242248715	2.93E-02
B4GALNT3	2.12E-05	3.978533862	1.89559158	8.350285925	2.62E-04
B4GALNT1	3.22E-05	4.321693916	1.54451038	12.09253013	5.30E-03
B3GNT7	1.81E-03	2.28023286	1.312901659	3.960282829	3.43E-03
B3GNT5	1.02E-02	49.93234078	1.165994052	2138.294489	4.13E-02
B3GALTL	9.24E-03	1.978841709	1.114422391	3.513761515	1.98E-02

B2M	4.67E-04	1.633047946	1.266187101	2.106201834	1.58E-04
AZGP1	1.46E-05	0.521780639	0.380903837	0.714760546	5.09E-05
AVPR2	5.53E-04	0.022174057	0.001147107	0.428633946	1.17E-02
AUTS2	3.71E-02	2.017129317	1.126068527	3.613288698	1.83E-02
AURKA	2.54E-02	3.390492663	1.565174215	7.344511806	1.96E-03
AUP1	9.01E-04	6.699623823	2.680753729	16.74341022	4.70E-05
ATXN7L3B	8.73E-06	0.100734495	0.035396398	0.286679975	1.70E-05
ATXN2L	3.61E-02	0.112787955	0.026570698	0.478765085	3.09E-03
ATXN10	5.98E-04	3.18663541	1.132462659	8.966869814	2.81E-02
ATRIP	2.14E-03	8.39E-12	4.52E-19	0.000155922	2.82E-03
ATRAID	1.18E-04	12.66240715	3.529259252	45.43065369	9.83E-05
ATPIF1	2.55E-02	2.517918595	1.239964517	5.112980223	1.06E-02
ATP9A	1.01E-03	2.646934427	1.343235025	5.215961266	4.91E-03
ATP8B3	5.99E-05	12.3844694	4.430221545	34.62018337	1.60E-06
ATP8B2	2.40E-03	2.63443972	1.53503265	4.521254084	4.40E-04
ATP8B1	7.63E-03	2.654614442	1.575717962	4.472232979	2.44E-04
ATP6V1H	4.68E-03	2.275978646	1.330797938	3.892460791	2.67E-03
ATP6V1B1-AS1	1.14E-02	0.018396726	0.000570714	0.593010363	2.41E-02
ATP6V0E1	1.14E-03	5.68787371	1.780330255	18.1718573	3.35E-03
ATP6V0D2	9.19E-04	2.036494133	1.08523298	3.821583414	2.68E-02
ATP6V0B	6.12E-05	2.763165584	1.451432286	5.260379087	1.97E-03
ATP5O	3.19E-03	4.846053274	1.801357456	13.03696402	1.77E-03
ATP5LP6	1.45E-02	15267.89419	1.989493258	117169833.1	3.48E-02
ATP5J2-PTCD1	3.74E-03	91989.00884	3.37671191	2505981551	2.83E-02
ATP5J2	2.85E-05	39.99182445	9.37500532	170.5968123	6.23E-07
ATP5J	2.20E-02	6.085400268	1.660699617	22.29909374	6.42E-03
ATP5I	8.22E-04	8.818996622	2.99447386	25.97274347	7.81E-05
ATP5HP1	2.61E-02	13493975.9	45.6211092	3.9913E+12	1.06E-02
ATP5H	2.47E-02	8.503017174	2.530365788	28.5734582	5.38E-04
ATP5G3	3.73E-05	8.58487665	2.672316748	27.57910609	3.05E-04
ATP5G2P3	2.04E-05	11097.49864	69.47128271	1772739.342	3.20E-04
ATP5G1	7.69E-03	4.675458135	1.748053054	12.50528908	2.12E-03
ATP5E	6.19E-03	4.867597413	2.002249315	11.83344372	4.80E-04

ATP5A1P3	2.85E-02	2871.462486	2.99327962	2754602.929	2.30E-02
ATP4B	1.37E-02	0.098814404	0.01056275	0.924407618	4.25E-02
ATP2A3	4.30E-03	3.054766825	1.696099214	5.501801004	1.99E-04
ATP2A2	1.36E-03	2.910836975	1.404463742	6.032887601	4.06E-03
ATP1B3	3.03E-03	3.795462482	1.570964458	9.169867195	3.04E-03
ATP1B1	6.42E-03	1.405072623	1.034491905	1.908404567	2.95E-02
ATP13A4-AS1	5.16E-03	1.51E+47	383572.9735	5.97E+88	2.62E-02
ATP12A	2.59E-05	5087840.491	143.6065079	1.80257E+11	3.86E-03
ATP11C	3.29E-02	3.190109224	1.362267321	7.470484467	7.54E-03
ATOX1	3.71E-04	6.730998278	2.178466525	20.79735322	9.24E-04
ATIC	7.27E-03	3.305322808	1.370080576	7.974099523	7.80E-03
ATHL1	3.47E-04	3.871641194	2.096634855	7.149363897	1.52E-05
ATG2A	4.22E-04	0.439036134	0.223171394	0.86369818	1.71E-02
ATG10-IT1	2.90E-05	1.31296E+13	22830.63411	7.55E+21	3.33E-03
ATF6B	2.77E-02	0.322302969	0.148132403	0.701259158	4.31E-03
ATF3	1.96E-02	2.159605949	1.192248476	3.911850547	1.11E-02
ATAT1	1.30E-03	0.218795532	0.084401091	0.567190354	1.77E-03
ATAD3C	2.12E-02	0.475855393	0.278265916	0.813748079	6.67E-03
ATAD2	2.77E-02	2.666901693	1.495833683	4.754783047	8.85E-04
ATAD1	3.89E-02	1.959430256	1.031378671	3.722558007	3.99E-02
ASTN2	4.52E-04	2.084598465	1.358019352	3.199918142	7.81E-04
ASTE1	1.90E-02	0.154971142	0.043486905	0.552259462	4.03E-03
ASS1P9	2.50E-02	4.89501E+14	1.527401313	1.57E+29	4.72E-02
ASS1P6	1.99E-03	5.27E+63	99515399965	2.79E+116	1.78E-02
ASS1P5	3.34E-03	4.17109E+16	93.94135161	1.85E+31	2.62E-02
ASS1P4	1.88E-02	1.43E+47	1.851361381	1.10E+94	4.87E-02
ASS1	1.07E-06	3.091934271	1.758556565	5.436309373	8.83E-05
ASRGL1	5.40E-04	0.188201628	0.07434587	0.476419911	4.24E-04
ASPHD1	1.60E-02	0.31686561	0.159503728	0.629476291	1.03E-03
ASPG	2.49E-02	0.305098839	0.101814002	0.91426817	3.40E-02
ASMTL	4.78E-03	0.370986526	0.167032235	0.823978688	1.49E-02
ASMT	1.49E-03	0.003373185	4.96E-05	0.229447003	8.20E-03
ASL	1.77E-02	3.865804308	1.559581583	9.582341261	3.51E-03

ASIP	1.66E-03	2.107594472	1.411289295	3.147444309	2.69E-04
ASIC3	4.09E-03	0.151259848	0.039209388	0.583522023	6.11E-03
ASIC1	3.23E-03	4.520670606	2.031499674	10.05979129	2.18E-04
ASCL2	1.39E-02	3.29325517	1.393395305	7.783526736	6.61E-03
ASB9	1.54E-06	2.361715902	1.517342596	3.675967456	1.41E-04
ASAP1	6.38E-03	2.100975231	1.328972219	3.321436564	1.49E-03
ARX	9.35E-04	1.46481167	1.166220485	1.839852117	1.03E-03
ART5	1.67E-02	0.579342719	0.349904054	0.959228629	3.39E-02
ART3	1.03E-02	3.05E-06	8.21E-10	0.011298352	2.45E-03
ARSH	8.00E-05	1.35E-09	1.23E-15	0.001483574	4.01E-03
ARSG	5.25E-03	2.931728038	1.450431221	5.925844096	2.74E-03
ARSEP1	2.58E-02	0.209907562	0.045238535	0.9739746	4.62E-02
ARSE	3.90E-02	0.614463342	0.401326551	0.940792972	2.50E-02
ARRB2	1.30E-02	2.840370886	1.342037164	6.011537524	6.35E-03
ARPP21-AS1	7.93E-03	3.52453E+11	368.2188559	3.37E+20	1.17E-02
ARPC4-TTLL3	1.45E-03	2.44E-05	9.47E-09	0.062711572	8.02E-03
ARPC4	8.62E-03	0.141637467	0.041649002	0.481672334	1.75E-03
ARPC3	6.10E-03	4.380894986	1.533786508	12.51298064	5.80E-03
ARPC2	4.47E-04	6.189472636	2.155824239	17.77026662	7.05E-04
ARPC1B	5.33E-06	11.98135775	4.081923505	35.16796271	6.18E-06
ARNTL2	2.97E-04	169788.6709	845.6739358	34089016.53	8.53E-06
ARMCX6	3.77E-05	0.200621727	0.073187037	0.549948175	1.80E-03
ARMC9	1.88E-06	2.405804312	1.62965117	3.55161552	9.99E-06
ARMC8	4.98E-02	0.338615073	0.130274309	0.880144127	2.63E-02
ARMC3	3.87E-02	4.69E-10	2.50E-16	0.00088183	3.57E-03
ARL9	2.42E-02	3.412339368	1.20139603	9.692107906	2.12E-02
ARL6IP4	3.57E-03	23.83738773	3.388968033	167.6678706	1.44E-03
ARL2	6.20E-03	0.341295393	0.179816885	0.647784245	1.01E-03
ARL15	2.99E-02	2.824658981	1.232667841	6.472707481	1.41E-02
ARL11	1.74E-03	15.45021611	2.345748198	101.7624901	4.42E-03
ARIH2OS	1.76E-02	0.163909092	0.048991149	0.54838865	3.34E-03
ARIH1	3.17E-02	2.836801971	1.131032336	7.115132929	2.63E-02
ARID5A	4.09E-03	3.357226142	1.658423608	6.796193275	7.63E-04

ARHGEF5	1.06E-03	0.132301214	0.039148959	0.447102856	1.13E-03
ARHGEF37	3.58E-04	0.290886757	0.163693037	0.516913285	2.56E-05
ARHGEF15	2.39E-02	3.796089283	1.193638211	12.07258089	2.38E-02
ARHGDIG	1.33E-02	2.467406998	1.620639208	3.756602496	2.54E-05
ARHGAP9	2.39E-02	2.14610658	1.284392083	3.585955966	3.55E-03
ARHGAP36	1.81E-02	0.001825942	9.38E-06	0.355485274	1.91E-02
ARHGAP31-AS1	1.06E-02	0.028033829	0.001915977	0.410180068	9.03E-03
ARHGAP31	1.03E-04	0.104080915	0.02710607	0.39964616	9.80E-04
ARHGAP27	2.17E-02	4.706116922	1.638094735	13.52030258	4.02E-03
ARHGAP19-SLIT1	5.73E-04	1304.601023	1.141442824	1491081.106	4.58E-02
ARHGAP19	6.06E-03	6.560791251	1.942895328	22.15455522	2.45E-03
ARG2	7.04E-05	4.030779036	1.898624444	8.557342496	2.84E-04
ARF1P3	1.99E-03	4.12E+26	39215.84776	4.32E+48	1.78E-02
ARF1	2.76E-02	7.472362781	2.037944972	27.39828911	2.41E-03
AREG	2.27E-02	15.81986009	2.598963344	96.29530703	2.73E-03
ARC	2.91E-04	2.731319744	1.859029845	4.012903592	3.08E-07
ARAP3	7.06E-03	0.255921604	0.098018301	0.668200392	5.38E-03
ARAP1-AS1	1.16E-03	3785395873	19844.03065	7.22092E+14	3.78E-04
ARAP1	4.25E-02	2.73905393	1.028930461	7.291470824	4.37E-02
AQP7P2	1.50E-02	3896893698	1.100694957	1.37965E+19	4.90E-02
AQP3	5.06E-04	7.03095147	2.388887019	20.69343514	3.99E-04
AQP1	1.84E-03	2.024448917	1.294818668	3.16522577	1.98E-03
APP	4.15E-03	2.368243296	1.259951409	4.451422706	7.41E-03
APOO	1.86E-02	4.224471272	1.188833184	15.01149005	2.59E-02
APOM	5.68E-04	0.124895522	0.046875732	0.332771151	3.17E-05
APOL6	7.44E-03	1.549053656	1.168710926	2.053174293	2.33E-03
APOL4	1.59E-03	2.0721718	1.281288378	3.351233056	2.97E-03
APOL3	2.09E-05	1.550179813	1.192895024	2.014475208	1.04E-03
APOL2	7.88E-03	1.919318472	1.252157365	2.94194923	2.77E-03
APOL1	2.99E-02	1.351328351	1.128605532	1.618004041	1.05E-03
APOBR	8.14E-05	2.789622546	1.621430512	4.799461888	2.11E-04
APOBEC3D	7.82E-04	4.110012475	1.821050891	9.276073847	6.66E-04
APOBEC3B	4.05E-02	3.054501375	1.374904523	6.785910214	6.11E-03

APOBEC2	5.11E-04	0.001142045	7.06E-06	0.184717906	9.03E-03
APOA1BP	6.58E-03	4.141523231	1.817209838	9.43876393	7.22E-04
APLP2	2.34E-03	1.746594498	1.005633603	3.03350279	4.77E-02
APLNR	2.88E-02	2.664742269	1.50607128	4.714817587	7.61E-04
APITD1-CORT	2.15E-03	93.59102908	3.767096009	2325.207721	5.62E-03
APITD1	6.50E-03	7.687140485	1.967496288	30.03417551	3.35E-03
APEH	1.10E-03	0.129691782	0.045383937	0.37061479	1.37E-04
APBB1IP	2.93E-03	2.172693673	1.205472878	3.915971799	9.83E-03
APBB1	1.19E-02	0.21840582	0.08594051	0.55504793	1.39E-03
APBA3	1.42E-03	4.81523338	1.044103758	22.2070578	4.39E-02
AP4S1	2.04E-03	0.153253886	0.042952069	0.54681309	3.85E-03
AP3S2	2.91E-02	2.960465739	1.147244347	7.639486229	2.48E-02
AP2S1	8.51E-03	3.742907946	1.456485239	9.618607533	6.13E-03
AP2M1	2.61E-03	0.310333604	0.144329452	0.667271611	2.74E-03
AP1M1	4.65E-03	7.177286156	1.723684566	29.88565169	6.77E-03
AP006564.1	2.33E-02	2.730957139	1.425044597	5.233609465	2.47E-03
AP006547.3	1.00E-02	58553.23917	369.2312958	9285458.345	2.17E-05
AP006477.1	2.24E-02	19.12114869	1.241018847	294.6114221	3.45E-02
AP006285.7	1.06E-02	2.99075E+17	12.67193836	7.06E+33	3.64E-02
AP005118.1	5.16E-03	802400.502	4.998023386	1.2882E+11	2.62E-02
AP004782.1	1.25E-04	1.84E-08	1.73E-12	0.000196987	1.68E-04
AP004550.1	5.28E-04	8.16E+22	149752.0027	4.45E+40	1.13E-02
AP002967.1	7.23E-05	99637.98797	24.45015151	406039555.3	6.65E-03
AP002884.1	5.28E-04	20228.57215	9.3895937	43579641.9	1.13E-02
AP001631.10	1.04E-02	3.29E+22	4235.238016	2.55E+41	1.95E-02
AP001619.3	3.27E-02	1932506.353	166.3944624	22444141175	2.44E-03
AP001476.3	5.16E-03	1.77307E+16	83.76723417	3.75E+30	2.62E-02
AP001442.2	3.16E-02	3.39E+24	176328164.9	6.53E+40	3.15E-03
AP001434.2	2.76E-06	729154910.2	751.3138005	7.0765E+14	3.71E-03
AP001350.4	9.07E-04	2.701694608	1.488944356	4.902234074	1.08E-03
AP001058.3	6.27E-07	13825696956	68668.6183	2.78366E+15	1.79E-04
AP001048.4	7.23E-05	2.23928E+12	2692.563744	1.86E+21	6.65E-03
AP000770.1	4.74E-04	7.37756E+18	359836345.5	1.51E+29	3.36E-04

AP000695.6	1.26E-02	344.3975998	1.989499932	59617.8491	2.63E-02
AP000568.2	1.60E-03	1919239933	455.6240594	8.08448E+15	6.02E-03
AP000567.27	5.16E-03	21785265.23	7.387346316	6.42447E+13	2.62E-02
AP000487.6	2.03E-03	14.22858092	2.203120648	91.89352172	5.27E-03
AP000439.2	7.12E-03	100.2774344	9.960628368	1009.531073	9.20E-05
AP000439.1	8.24E-05	6.780780528	1.343090652	34.23371647	2.05E-02
AP000351.13	3.52E-02	0.144436172	0.024528054	0.850528456	3.24E-02
AP000320.7	5.94E-03	14.24214299	2.075419747	97.73378957	6.87E-03
AP000295.10	1.15E-03	266.1757468	17.45992924	4057.83593	5.88E-05
AP000282.2	1.75E-05	3309.103272	31.34620606	349329.8182	6.52E-04
AP000254.8	1.66E-02	3.754798281	1.618630448	8.710147613	2.06E-03
AP000237.1	5.28E-04	18283.92409	9.177649249	36425654.45	1.13E-02
AP000235.2	2.76E-06	3.41E+158	2.76E+51	4.21E+265	3.71E-03
AP000233.3	2.15E-02	463165.3093	3.383470969	63402968659	3.06E-02
AP000221.2	5.28E-04	124641.8747	14.15881059	1097238841	1.13E-02
AP000146.2	2.76E-06	1.63E+36	5.62154E+11	4.70E+60	3.71E-03
AOAH	2.59E-02	1.878897189	1.086372315	3.249580828	2.40E-02
ANXA4	2.99E-03	2.918323695	1.617612627	5.26492749	3.74E-04
ANXA2P2	5.38E-06	4.184994955	2.140279391	8.183129197	2.86E-05
ANXA2	4.39E-05	4.184050466	2.032482397	8.613249653	1.02E-04
ANXA13	8.34E-03	2009.331873	3.559789287	1134172.348	1.86E-02
ANTXR1	8.69E-03	1.769908383	1.185500329	2.642408111	5.24E-03
ANPEP	3.47E-05	4.620533624	2.478406281	8.614136887	1.47E-06
ANO9	1.95E-02	1.661750583	1.153588827	2.393760183	6.39E-03
ANO8	2.79E-04	0.168127986	0.060483692	0.467349442	6.30E-04
ANO7P1	6.60E-03	0.126166818	0.017006352	0.936007059	4.29E-02
ANO6	2.56E-02	2.263254914	1.292723147	3.962428319	4.26E-03
ANO4	2.03E-05	4.088721636	1.692160145	9.879469544	1.76E-03
ANO10	4.52E-04	0.177467205	0.062314934	0.505410293	1.20E-03
ANO1	1.67E-02	5.152439608	1.839745881	14.43005482	1.81E-03
ANKRD65	2.58E-02	0.436056802	0.248263073	0.765903411	3.88E-03
ANKRD53	3.94E-02	0.145981199	0.046326379	0.460008121	1.02E-03
ANKRD44	2.82E-02	2.436764773	1.391156329	4.26826406	1.84E-03

ANKRD36BP2	2.67E-03	1144.764057	40.73320823	32172.3921	3.50E-05
ANKRD34A	2.30E-06	2.794119227	1.524178282	5.122171301	8.91E-04
ANKRD30BP3	3.56E-03	2.05E+21	37202.36096	1.12E+38	1.26E-02
ANKRD30BP1	4.87E-03	7.909208887	2.04901437	30.5295981	2.69E-03
ANKRD30B	2.63E-03	2.054791685	1.342295782	3.145483228	9.16E-04
ANKRD26P3	2.46E-04	34.19125984	1.684699483	693.9173789	2.15E-02
ANKRD26P1	1.21E-03	7.31E+52	647436.7203	8.25E+99	2.77E-02
ANKRD2	3.13E-04	5.79493732	1.794773445	18.71060587	3.30E-03
ANKRD19P	3.81E-02	9.541166605	1.083058695	84.05256389	4.22E-02
ANKRD13D	1.26E-03	7.410483532	1.943716437	28.25271481	3.35E-03
ANKRD13A	4.97E-02	0.262118508	0.09586902	0.716666472	9.08E-03
ANKHD1-EIF4EBP3	2.48E-03	78.16360563	4.297301022	1421.717774	3.23E-03
ANKEF1	2.22E-03	0.095420463	0.019765931	0.46064437	3.45E-03
ANKDD1A	1.40E-02	2.097473501	1.170876242	3.757352764	1.28E-02
ANGPT2	4.17E-02	3.890743132	1.366475592	11.07804795	1.09E-02
ANG	5.73E-05	0.222281609	0.102161458	0.483637515	1.50E-04
ANAPC7	5.43E-03	7.380111884	1.875674172	29.03811986	4.24E-03
ANAPC2	1.40E-02	0.182713322	0.039193006	0.851788652	3.04E-02
ANAPC13	1.79E-05	0.158223017	0.059852753	0.418268531	2.01E-04
ANAPC11	3.20E-03	15.11805513	3.802454968	60.10737611	1.15E-04
AMZ2	3.72E-03	3.645934959	1.693881462	7.847563139	9.42E-04
AMZ1	9.38E-07	5.355088631	2.073086367	13.83298579	5.29E-04
AMYP1	5.16E-03	1.41E+46	289736.5494	6.90E+86	2.62E-02
AMOTL2	6.72E-04	0.340746147	0.190696693	0.608861824	2.78E-04
AMOTL1	3.35E-02	2.277021127	1.055646527	4.911516386	3.59E-02
AMN	9.21E-05	2.886128292	1.827699997	4.557496596	5.44E-06
AMMECR1	2.05E-02	5.007634161	2.057368796	12.18857792	3.86E-04
AMICA1	4.04E-05	4.006729379	1.827724734	8.783532888	5.28E-04
AMER1	5.49E-07	0.001268599	6.57E-05	0.024506319	1.01E-05
AMDHD2	1.73E-02	3.363553777	1.529142535	7.398586954	2.56E-03
AMBRA1	4.44E-02	0.298099609	0.11552425	0.769218383	1.23E-02
ALS2CR12	3.12E-02	3.394809476	1.500339015	7.681418174	3.35E-03
ALPK3	4.27E-03	8.966193893	1.170683711	68.67152261	3.47E-02

ALOX12	1.48E-03	0.022909689	0.002539428	0.20668188	7.66E-04
ALK	2.83E-02	1.735017122	1.16071413	2.593476151	7.22E-03
ALG9	1.75E-05	0.098090152	0.021670886	0.443990976	2.58E-03
ALG8	8.16E-06	5.492723013	2.245925322	13.43321873	1.89E-04
ALG5	9.28E-05	4.708959433	2.344579264	9.457687902	1.33E-05
ALG1L8P	6.01E-05	0.000562974	7.44E-06	0.042579774	6.99E-04
ALG1L7P	2.46E-04	6.69E-05	2.12E-07	0.021124279	1.06E-03
ALG1L6P	1.67E-04	0.287640404	0.142736834	0.579647171	4.92E-04
ALG1L2	1.76E-06	0.031985268	0.004568992	0.223913158	5.26E-04
ALG1L12P	8.01E-04	0.115285994	0.029592549	0.449128613	1.85E-03
ALG1L	2.28E-03	0.384527617	0.201760761	0.732855525	3.68E-03
ALG14	2.62E-03	6.018708369	1.873458902	19.3358127	2.58E-03
ALG12	3.13E-03	8.054056332	2.109033331	30.75713525	2.28E-03
ALG1	4.82E-04	5.456994503	2.225904343	13.37828784	2.08E-04
ALDH6A1	7.46E-03	0.266914468	0.119412622	0.596614765	1.29E-03
ALDH5A1	1.04E-04	0.384103946	0.205788964	0.716927857	2.65E-03
ALDH1L2	1.68E-02	1.55009179	1.053514843	2.280731567	2.61E-02
ALDH1L1-AS2	1.87E-04	0.007622097	0.000302345	0.192152717	3.06E-03
ALDH1L1	1.09E-05	0.30849184	0.172812875	0.550695169	6.96E-05
ALDH1A2	1.46E-03	2.729883641	1.439787497	5.175947639	2.09E-03
ALDH16A1	2.56E-03	8.027984083	2.639388813	24.41797439	2.43E-04
ALCAM	4.80E-02	1.991036598	1.070594601	3.702827132	2.96E-02
AL928874.1	5.28E-04	50223.39261	11.53074697	218753318.5	1.13E-02
AL928761.1	3.22E-02	3.333169513	1.511136758	7.352093677	2.86E-03
AL691477.1	2.79E-04	21116735.39	249.3624814	1.78823E+12	3.58E-03
AL596220.1	1.88E-02	271051.2274	1.07354366	68435752175	4.87E-02
AL590708.2	7.23E-05	664.3030722	6.079876239	72583.4794	6.65E-03
AL590084.1	5.28E-04	18283.92409	9.177649249	36425654.45	1.13E-02
AL512505.1	2.76E-06	732347.4093	79.98202905	6705665438	3.71E-03
AL391650.1	3.42E-02	342.1137972	1.048118311	111668.5483	4.82E-02
AL365331.2	3.02E-03	3344280019	341.1032899	3.27883E+16	7.58E-03
AL359771.1	1.76E-02	488.3309985	1.733207198	137587.2223	3.15E-02
AL359091.1	5.28E-04	27392.74241	10.05515927	74624609.73	1.13E-02

AL358852.1	4.77E-02	0.060904161	0.005677962	0.653283101	2.08E-02
AL358340.1	1.33E-02	0.299808459	0.10306551	0.872116307	2.70E-02
AL354981.1	1.64E-06	352764.5702	30.38598023	4095403243	7.48E-03
AL161668.5	2.63E-03	3.37E-10	8.53E-17	0.001329352	4.88E-03
AL158801.1	1.16E-04	19.95717609	5.466631577	72.85818917	5.87E-06
AL139295.1	1.88E-02	2011.265011	1.044093452	3874353.332	4.87E-02
AL139188.1	1.88E-02	1687.785027	1.043055431	2731032.515	4.87E-02
AL138726.1	2.76E-06	2449231.058	118.3374419	50691756358	3.71E-03
AL138479.3	1.06E-02	2.77676E+14	8.156193639	9.45E+27	3.64E-02
AL137792.1	5.28E-04	112670.3582	13.83951296	917272858.5	1.13E-02
AL137024.1	1.41E-03	72.75897495	6.627519041	798.7707622	4.53E-04
AL136985.1	5.28E-04	16526.1077	8.970474814	30445683.37	1.13E-02
AL136446.1	5.16E-03	36258.23983	3.464306243	379487222.9	2.62E-02
AL136380.1	2.76E-06	4187837.146	140.8359099	1.24528E+11	3.71E-03
AL133335.1	5.28E-04	6.74985E+16	6330.836733	7.20E+29	1.13E-02
AL133244.1	1.54E-02	13514.81025	2.361006838	77361104.21	3.12E-02
AL132768.1	1.06E-02	322.1192551	1.439704182	72070.92667	3.64E-02
AL122127.25	1.75E-04	10.46726494	2.718516612	40.3027279	6.40E-04
AL122127.2	1.50E-03	2.55451192	1.543626589	4.22740266	2.63E-04
AL121761.1	6.95E-05	85.22955894	3.304923082	2197.956666	7.34E-03
AL080272.1	5.28E-04	14937.18091	8.767962925	25447116.46	1.13E-02
AL050327.1	1.68E-03	0.000192155	8.19E-08	0.451060222	3.07E-02
AL050321.1	2.93E-02	0.018039792	0.00038895	0.836699125	4.03E-02
AL050310.1	1.84E-02	0.246180482	0.091628524	0.661418812	5.44E-03
AL049868.1	5.16E-03	209748.8503	4.264178655	10317246006	2.62E-02
AL035610.2	7.08E-05	2.85E-55	3.34E-99	2.43E-11	1.50E-02
AL033522.1	1.88E-02	88.92500038	1.025784421	7708.886517	4.87E-02
AL031587.1	1.12E-04	0.201559728	0.086167592	0.471480321	2.21E-04
AL031229.1	1.56E-03	458.2256035	1.842891601	113935.4608	2.95E-02
AL022345.7	5.97E-03	0.078178848	0.009136702	0.668942952	2.00E-02
AL022345.10	3.85E-02	0.002528916	1.17E-05	0.545301681	2.92E-02
AL022341.3	1.19E-05	0.010948446	0.001146615	0.104541134	8.80E-05
AL021918.1	1.35E-03	20731.54601	25.67502421	16739886.84	3.61E-03

AKT2	3.53E-02	0.138797443	0.032114293	0.599880256	8.19E-03
AKR7A3	5.94E-03	8.64780187	1.333753389	56.07069326	2.37E-02
AKR1D1	7.38E-03	2.00E+45	9.72E+23	4.13E+66	3.10E-05
AKR1C5P	5.16E-03	4.16E+27	1856.809411	9.31E+51	2.62E-02
AKR1B15	9.38E-05	180915682.8	6.092537303	5.37223E+15	3.03E-02
AKIP1	3.79E-05	5.071886778	1.519594101	16.92822806	8.28E-03
AKAP3	2.87E-05	0.003079877	0.000181866	0.052157423	6.18E-05
AKAP17A	2.07E-04	0.142000985	0.053143506	0.37943074	9.92E-05
AKAP13	3.30E-02	4.381952367	1.87200484	10.25718852	6.62E-04
AK8	1.25E-05	0.030185976	0.005417922	0.1681813	6.49E-05
AK4P1	1.36E-03	42.53100494	3.225000094	560.8949854	4.38E-03
AK4	6.98E-05	2.992077384	1.739471501	5.146693732	7.48E-05
AK3P4	5.16E-03	2.5461E+13	38.60257441	1.68E+25	2.62E-02
AK1	1.36E-02	0.337883383	0.117473461	0.971838059	4.41E-02
AJ006995.3	3.05E-03	1.67E-07	7.34E-14	0.381833982	3.67E-02
AIM2	2.12E-04	2.927042605	1.365770133	6.27307495	5.75E-03
AIM1L	4.12E-03	1.2331E+13	37121.3583	4.10E+21	2.60E-03
AIFM2	6.03E-06	6.104896507	2.780109603	13.40586045	6.55E-06
AIFM1	1.18E-03	3.51650736	1.554661392	7.954030425	2.53E-03
AIF1	3.60E-05	1.650613226	1.173074349	2.322550165	4.03E-03
AHRR	5.22E-03	4.123262698	1.985439693	8.562987505	1.45E-04
AHNAK2	5.49E-05	1.936649442	1.343632404	2.791396701	3.95E-04
AHCYL2	2.69E-04	0.522143826	0.378804632	0.719722391	7.23E-05
AGTRAP	3.03E-02	3.166367385	1.391419753	7.205505306	6.01E-03
AGPAT4	3.41E-04	7.316224065	2.759169408	19.39972747	6.34E-05
AGPAT3	3.36E-03	3.091626417	1.438622006	6.643964751	3.83E-03
AGGF1P3	2.91E-02	3.75E-06	1.89E-11	0.744498691	4.47E-02
AGGF1P2	1.44E-02	0.066950316	0.006664038	0.672616979	2.16E-02
AGGF1P1	1.67E-02	2.56E-06	7.92E-12	0.830372865	4.67E-02
AGFG1	1.91E-02	3.086289961	1.262932434	7.542118223	1.34E-02
AGBL4	4.12E-03	9.83E-06	1.65E-09	0.058695541	9.35E-03
AGBL1	3.58E-04	6.06E+23	258059.4547	1.42E+42	1.12E-02
AFF3	4.66E-03	0.469204265	0.231216864	0.952147858	3.61E-02

AFAP1L2	1.84E-03	2.61798047	1.253892076	5.466038008	1.04E-02
AF186996.1	2.76E-06	428163.421	67.19752999	2728134727	3.71E-03
AF186192.6	7.10E-03	0.004749069	0.000104796	0.21521462	5.97E-03
AF146191.4	3.87E-02	0.000346261	5.16E-07	0.232183622	1.64E-02
AF131216.5	3.05E-03	1.529562251	1.001930571	2.335052693	4.90E-02
AF131216.1	1.44E-02	0.002568768	4.27E-05	0.154421094	4.32E-03
AF127936.5	9.06E-03	9.446916757	2.287876499	39.00745352	1.91E-03
AF127577.12	1.06E-02	0.014827626	0.000231538	0.949557989	4.72E-02
AF111168.2	3.84E-04	272.417884	4.790600588	15491.06467	6.53E-03
AE000658.31	5.39E-04	2.81885E+12	533227.9418	1.49015E+19	2.84E-04
AE000658.25	1.59E-02	1.18E+22	11.52852364	1.21E+43	3.95E-02
ADRB2	2.24E-02	0.318870283	0.149953659	0.678064529	2.99E-03
ADRA2C	3.01E-02	0.522666097	0.346077897	0.789359422	2.04E-03
ADRA2B	4.70E-03	5.978724724	1.135531849	31.47877301	3.49E-02
ADRA2A	7.99E-04	1.517540023	1.035847077	2.223231377	3.23E-02
ADPRH	6.18E-03	0.251677437	0.109795784	0.576903138	1.12E-03
ADORA3	4.73E-02	2.494018001	1.199215244	5.186830157	1.44E-02
ADORA2B	7.15E-03	3.123957026	1.322816544	7.377521505	9.38E-03
ADORA2A-AS1	3.24E-02	0.000409548	1.17E-06	0.143136516	9.04E-03
ADM2	2.46E-05	4.601464502	2.496752193	8.480407313	9.92E-07
ADK	2.35E-02	3.152814584	1.314032842	7.564681405	1.01E-02
ADI1	8.87E-04	3.527913547	1.468073805	8.477893925	4.83E-03
ADH5P2	2.42E-05	5.58E+21	31080836.12	1.00E+36	2.79E-03
ADCY6	6.83E-05	0.301019439	0.164636296	0.550381082	9.64E-05
ADCY4	1.98E-03	42.90913419	3.237449263	568.7174214	4.36E-03
ADCY3	6.55E-03	3.11289941	1.580931154	6.129389451	1.02E-03
ADCY2	5.77E-03	2.789968348	1.19883959	6.492881489	1.73E-02
ADCY1	7.55E-05	1.483765541	1.126537424	1.954271678	4.99E-03
ADCK5	1.39E-05	4.704348747	2.35114654	9.412810625	1.21E-05
ADAT3	6.51E-03	3.220620266	1.00556535	10.31498838	4.89E-02
ADAMTSL4	8.06E-04	0.283310086	0.130293434	0.616029545	1.46E-03
ADAMTS9	6.49E-03	692.5706114	31.93842142	15018.08889	3.09E-05
ADAMTS5	5.11E-03	34.60900796	3.032060769	395.0393885	4.33E-03

ADAMTS4	3.43E-02	15.0890128	3.49285716	65.18397312	2.78E-04
ADAMTS2	3.20E-04	2.193153363	1.501469106	3.203476951	4.86E-05
ADAMTS19-AS1	8.92E-03	1135.41409	14.7943999	87138.72577	1.49E-03
ADAMTS14	1.83E-04	2.789188275	1.648404418	4.719455462	1.32E-04
ADAMTS12	4.56E-02	295.9911214	9.785227676	8953.36796	1.07E-03
ADAMDEC1	3.22E-07	1.931431063	1.287961262	2.896380552	1.45E-03
ADAM33	6.73E-03	1.937908534	1.198257469	3.134125664	6.99E-03
ADAM23	6.09E-03	1.464253264	1.159448193	1.849187945	1.36E-03
ADAM19	2.25E-04	1.803842118	1.014926227	3.205993007	4.44E-02
ADAM15	1.75E-03	3.761628789	1.794180167	7.886527455	4.52E-04
ADAM12	9.28E-03	3.907577119	2.040907742	7.481552754	3.91E-05
ADAM11	3.92E-06	2.828831346	1.772500142	4.51468894	1.30E-05
ADAM10	1.90E-02	2.230964819	1.309379404	3.801193154	3.16E-03
ACY3	7.24E-03	2.922078242	1.10075493	7.756986611	3.13E-02
ACVR2B-AS1	1.55E-06	0.012879965	0.000942457	0.176022298	1.11E-03
ACVR2B	2.73E-02	0.099723326	0.021334952	0.466124407	3.39E-03
ACTRT3	1.26E-02	0.137101537	0.023739852	0.791783853	2.64E-02
ACTN3	9.11E-03	1.02E-08	1.49E-16	0.697152262	4.56E-02
ACTN1	4.78E-02	2.605626055	1.222265258	5.554675707	1.32E-02
ACTL10	1.48E-03	4.476067161	2.057192924	9.739085233	1.58E-04
ACTG1P14	8.10E-03	19.46882634	1.303080481	290.876277	3.14E-02
ACTG1P1	1.39E-04	0.001323804	2.22E-05	0.07887039	1.48E-03
ACTBP2	3.51E-03	9.315176759	1.336575176	64.92153946	2.43E-02
ACTB	1.96E-03	8.196084082	2.617069985	25.6683217	3.04E-04
ACTA2-AS1	1.71E-02	32.41707754	2.434437235	431.6672868	8.45E-03
ACTA1	1.95E-02	7.642777945	2.106062234	27.73519879	1.98E-03
ACSM5	4.89E-02	17.9688153	1.125745038	286.8130106	4.10E-02
ACSL6	4.56E-04	2.283441779	1.099533069	4.742109633	2.68E-02
ACSL1	6.64E-03	1.873686292	1.236619189	2.838950221	3.06E-03
ACSF2	1.30E-06	0.374046448	0.221309634	0.632194553	2.40E-04
ACSBG1	1.95E-03	1.780388677	1.017806983	3.114327071	4.32E-02
ACRC	8.88E-03	17.49338723	5.036861937	60.75580401	6.63E-06
ACOX3	1.32E-04	5.286881153	1.842119287	15.17334547	1.96E-03

ACOX2	3.61E-02	0.275668065	0.091286013	0.832470163	2.23E-02
ACOT8	2.76E-03	3.571446529	1.592567462	8.009224482	2.01E-03
ACOT4	1.05E-02	3.425377059	1.505077337	7.795750894	3.34E-03
ACOT2	2.57E-02	5.548988649	1.739314299	17.70311154	3.79E-03
ACOT12	2.04E-04	1.42E-06	1.53E-10	0.013271505	3.89E-03
ACN9	3.89E-02	0.50304457	0.264201963	0.957804537	3.65E-02
ACLY	2.82E-02	3.897327415	1.383116083	10.98184106	1.01E-02
ACKR2	8.25E-07	4.78E-07	1.17E-10	0.001942821	5.98E-04
ACE	5.96E-03	16.68999722	3.727037231	74.73926073	2.33E-04
ACCSL	7.58E-03	0.366608111	0.196649656	0.683456607	1.59E-03
ACAT2	1.10E-02	3.229749345	1.501111864	6.9490363	2.71E-03
ACAN	1.06E-02	6.036369399	2.688672329	13.55232288	1.32E-05
ACADVL	5.79E-06	4.102837157	2.038140433	8.259132914	7.66E-05
ACAD10	2.44E-05	5.368134161	2.157704089	13.35533659	3.02E-04
ACACB	7.58E-05	3.93701292	1.785740451	8.67991243	6.80E-04
ACACA	1.87E-02	4.947148558	1.705658192	14.34887657	3.25E-03
ACAA1	1.31E-03	0.308918445	0.116209493	0.82119458	1.85E-02
AC246787.4	1.01E-02	2.605279883	1.393737747	4.869985967	2.70E-03
AC246787.1	1.10E-02	4.190054263	1.684303723	10.42362758	2.06E-03
AC245028.1	2.86E-02	7.285783108	2.082721019	25.48715599	1.88E-03
AC244250.3	9.71E-05	271.84363	4.609370973	16032.33058	7.05E-03
AC244157.1	9.71E-05	23.90000013	2.439624255	234.1385175	6.41E-03
AC242988.1	3.77E-02	4.854773591	1.646241604	14.31674826	4.19E-03
AC241585.2	2.05E-02	15.47385651	2.6588077	90.05549189	2.30E-03
AC226150.3	1.88E-02	1605564.495	1.084431825	2.37713E+12	4.87E-02
AC226119.5	3.48E-05	3.78592E+14	1830012.493	7.83E+22	5.90E-04
AC217773.1	1.06E-02	53779.52863	1.988542041	1454451372	3.64E-02
AC159540.14	1.50E-04	3.87E+46	8.97319E+15	1.67E+77	2.88E-03
AC147651.4	2.97E-04	3.358034805	1.781378548	6.330152434	1.80E-04
AC144652.1	2.29E-02	0.240191055	0.05971292	0.966151766	4.46E-02
AC141928.1	2.35E-02	0.095089955	0.020021126	0.451627922	3.08E-03
AC140725.4	3.21E-04	1.22E-13	7.38E-22	2.01E-05	2.07E-03
AC139451.1	5.16E-03	67375.27979	3.727899146	1217690755	2.62E-02

AC138744.2	1.97E-02	158.1136146	1.189649046	21014.52963	4.24E-02
AC138304.1	1.88E-02	13822.01458	1.055572204	180990069.9	4.87E-02
AC138123.1	5.16E-03	36258.23983	3.464306243	379487222.9	2.62E-02
AC133644.2	4.48E-03	9.173512919	2.655593038	31.6890947	4.58E-04
AC130469.1	3.64E-02	17.12565582	1.124300746	260.8626635	4.09E-02
AC128709.4	5.74E-03	4.08E-06	9.77E-10	0.017082166	3.54E-03
AC127391.3	4.45E-03	121206.6325	3.981490493	3689836202	2.63E-02
AC124861.1	1.35E-02	1420.391635	14.06377673	143454.5241	2.05E-03
AC118463.1	6.01E-03	12.01378119	1.115300252	129.4099398	4.04E-02
AC118282.1	1.87E-04	618.2572862	16.18626024	23615.21848	5.44E-04
AC117507.1	1.88E-02	27847.32894	1.059774901	731734378.5	4.87E-02
AC117479.1	5.16E-03	32700.28751	3.422217176	312460825.3	2.62E-02
AC115522.3	4.32E-03	0.138663632	0.02717981	0.707422263	1.75E-02
AC114776.3	2.37E-02	7.35E+87	5.99654E+12	9.00E+162	2.18E-02
AC114737.6	9.61E-06	2.24E+22	12275320794	4.09E+34	3.53E-04
AC113189.5	4.33E-02	0.327353504	0.136422537	0.785503033	1.24E-02
AC112721.1	3.46E-02	2.27313E+11	106.3950646	4.86E+20	1.70E-02
AC110781.3	2.51E-06	2.432702864	1.711007723	3.458805676	7.38E-07
AC109826.1	6.13E-04	2.246528359	1.326443083	3.80482942	2.61E-03
AC109335.1	2.07E-02	184.8278083	4.425901971	7718.498725	6.12E-03
AC108676.1	7.45E-03	2.88E-13	1.95E-25	0.42701522	4.34E-02
AC108463.2	4.47E-02	6.53034695	2.706964551	15.7539674	2.96E-05
AC108463.1	1.35E-02	71.56728599	8.835685858	579.6806843	6.30E-05
AC108051.1	2.76E-06	8.41E+25	258307129.9	2.74E+43	3.71E-03
AC106870.1	6.48E-03	3.53864E+17	8.536054538	1.47E+34	3.85E-02
AC106801.1	2.30E-02	3.12E+22	77.31214361	1.26E+43	3.24E-02
AC106786.1	2.05E-02	1.56776E+11	159383.7438	1.5421E+17	2.51E-04
AC105402.3	5.16E-03	5.68E+24	850.5627227	3.79E+46	2.62E-02
AC104978.1	5.54E-03	8.28E-10	1.32E-17	0.052117416	2.25E-02
AC104946.1	5.94E-03	8.989433003	1.316708107	61.37268032	2.50E-02
AC104820.2	5.96E-05	22885.59673	40.94350146	12792030.95	1.87E-03
AC104809.3	3.47E-02	28.03705256	1.296020999	606.5305402	3.36E-02
AC104654.2	6.41E-03	5.679010027	1.149792343	28.04954745	3.31E-02

AC104389.28	2.82E-03	212635369.6	2489.088129	1.81648E+13	9.34E-04
AC103996.1	1.88E-02	51393.88166	1.063465183	2483702443	4.87E-02
AC103740.1	4.19E-03	50.25688852	2.706734793	933.1371696	8.59E-03
AC103563.8	2.09E-02	0.293495028	0.143690467	0.599478401	7.68E-04
AC103563.3	2.76E-06	8.22E+21	12909378.78	5.23E+36	3.71E-03
AC100848.1	2.76E-06	2.30E+38	2.80252E+12	1.88E+64	3.71E-03
AC100821.2	2.50E-02	23.99691002	2.328538385	247.3017814	7.58E-03
AC099344.3	1.37E-05	1.91E+30	4.06914E+12	8.96E+47	7.84E-04
AC099339.1	5.28E-04	252695.3251	16.60955309	3844469927	1.13E-02
AC098795.1	4.14E-03	1.51E-09	4.71E-18	0.481198992	4.20E-02
AC097713.4	3.58E-04	1.80668E+14	335510.2202	9.73E+22	1.37E-03
AC097713.3	9.45E-03	1.70422E+15	32.85965371	8.84E+28	2.95E-02
AC097533.1	5.44E-03	74450501.75	4042.765954	1.37106E+12	2.98E-04
AC097495.2	2.53E-03	2521238589	15.377689	4.13368E+17	2.49E-02
AC097461.4	1.23E-03	149.0552091	5.495218958	4043.051882	2.96E-03
AC096664.2	1.06E-02	1.04E+23	28.341014	3.78E+44	3.64E-02
AC096558.1	4.91E-03	132638.4077	15.50625167	1134571241	1.07E-02
AC093850.1	4.86E-02	12834.6981	3.809237562	43244736.7	2.24E-02
AC093818.1	3.89E-02	302.6794286	21.75356446	4211.486199	2.11E-05
AC093787.1	1.07E-02	2.81716159	1.125624667	7.050662318	2.69E-02
AC093732.1	6.78E-04	8090035.65	341.111643	1.91869E+11	1.97E-03
AC093724.2	2.29E-02	26.20351556	4.177037248	164.380681	4.91E-04
AC093702.1	1.21E-02	133.5194638	3.975571724	4484.247413	6.34E-03
AC093673.5	2.60E-03	3.421962383	1.588857963	7.369964355	1.67E-03
AC093620.5	7.66E-05	17.71079049	4.66538966	67.23384811	2.41E-05
AC093616.4	1.56E-02	153.0909762	6.015765982	3895.90404	2.31E-03
AC093484.4	2.05E-03	644.1337227	18.44397774	22495.59496	3.60E-04
AC093381.2	5.28E-04	3.99E+49	1.60009E+11	9.97E+87	1.13E-02
AC093106.5	3.53E-04	132.0595054	4.760418827	3663.482897	3.97E-03
AC093063.2	1.47E-04	2.483282179	1.312899985	4.696999354	5.16E-03
AC092835.2	4.83E-04	1.09E-06	4.25E-10	0.002804187	6.10E-04
AC092638.2	1.65E-08	3.92E+21	15312557993	1.00E+33	2.07E-04
AC092580.3	4.63E-02	1.90E-05	3.68E-10	0.981331435	4.96E-02

AC092578.1	7.23E-05	2.03069E+12	2620.42424	1.57E+21	6.65E-03
AC092431.3	1.61E-02	0.002057302	1.47E-05	0.287871148	1.41E-02
AC092415.1	9.08E-04	8.17E+33	72511149219	9.20E+56	3.93E-03
AC091878.1	1.99E-03	0.035724803	0.003545195	0.359997559	4.70E-03
AC091814.3	1.31E-04	2.78234E+12	10150.38776	7.63E+20	3.85E-03
AC091654.7	7.23E-04	73.74795167	8.827264746	616.1320105	7.16E-05
AC091199.1	3.52E-04	16518853442	24956.14569	1.09341E+16	5.80E-04
AC091167.1	2.79E-04	8271.584575	19.14700051	3573359.249	3.57E-03
AC090505.6	9.90E-03	2.55E-39	1.45E-73	4.49E-05	2.72E-02
AC090505.5	1.86E-02	3.23E-13	2.12E-23	0.004933146	1.62E-02
AC090044.2	2.92E-02	0.46126172	0.231570917	0.918778476	2.77E-02
AC087499.7	7.74E-03	1.47209E+12	290.3685003	7.46E+21	1.40E-02
AC087499.4	4.74E-03	4.683531962	1.53817112	14.2607486	6.57E-03
AC084125.4	3.07E-04	5.479699513	2.290266692	13.11074682	1.33E-04
AC083949.1	3.18E-05	101.1327952	3.073314451	3327.95177	9.60E-03
AC079951.1	1.27E-02	32.92731818	5.251046236	206.4747165	1.91E-04
AC079896.1	1.88E-02	6.8656E+18	1.278950047	3.69E+37	4.87E-02
AC079796.1	4.87E-05	1.689142379	1.287922627	2.215352007	1.51E-04
AC079779.6	7.41E-03	5.79E+20	6133.087318	5.46E+37	1.65E-02
AC079776.1	8.13E-06	0.003494003	0.000253346	0.04818727	2.39E-05
AC079767.4	9.20E-03	2.540809477	1.20891182	5.340102309	1.39E-02
AC079610.2	1.37E-05	4.15E-15	9.51E-24	1.81E-06	1.10E-03
AC079150.2	3.39E-02	141.4249875	2.085118498	9592.273586	2.14E-02
AC079145.4	2.93E-02	0.113518816	0.017081838	0.754398997	2.43E-02
AC078942.1	4.49E-02	50861975.55	9.678580278	2.67285E+14	2.46E-02
AC078819.1	3.12E-03	1748.144107	4.939383147	618702.3213	1.27E-02
AC074389.6	2.44E-02	248.7483405	1.07918321	57335.71122	4.69E-02
AC074366.3	1.84E-04	9.26639E+11	318092.105	2.69941E+18	2.85E-04
AC073508.1	2.38E-03	1614.268365	14.31089964	182089.3458	2.19E-03
AC073465.3	4.35E-03	245.927617	5.720919076	10571.7966	4.12E-03
AC073464.7	5.16E-03	5.14648E+15	72.35914809	3.66E+29	2.62E-02
AC073254.1	1.92E-02	52.5781481	1.636324047	1689.434109	2.52E-02
AC072062.1	4.91E-03	5.88E-09	6.20E-16	0.055852517	2.08E-02

AC072031.1	2.76E-06	286247.1266	58.9673668	1389538349	3.71E-03
AC069368.3	3.31E-02	35.92500827	1.175686547	1097.746864	4.01E-02
AC069363.1	1.95E-04	55.40920275	3.936699527	779.886737	2.92E-03
AC069155.1	2.15E-03	9.14854E+13	478422.248	1.75E+22	9.53E-04
AC068499.10	1.57E-03	0.01355399	0.00069719	0.263501713	4.50E-03
AC068014.2	2.76E-06	1637886.871	103.8533173	25831369402	3.71E-03
AC064871.3	3.03E-03	7.58299E+16	5093.546839	1.13E+30	1.20E-02
AC064847.4	5.28E-04	1.12782E+11	313.7036591	4.05473E+19	1.13E-02
AC063979.1	1.88E-02	4.19561E+14	1.210458101	1.45E+29	4.87E-02
AC061992.2	1.47E-02	18.96700795	1.170963261	307.2234649	3.84E-02
AC058791.1	4.56E-02	5.58631831	1.334292639	23.38838674	1.85E-02
AC055733.1	2.09E-04	69408258.6	1329.745697	3.62288E+12	1.12E-03
AC053503.4	5.21E-04	6.33E-07	5.07E-11	0.007908996	3.02E-03
AC037445.1	5.16E-03	5.28E+29	3294.295753	8.47E+55	2.62E-02
AC034236.2	5.16E-03	36258.23983	3.464306243	379487222.9	2.62E-02
AC034220.3	3.49E-02	0.128316407	0.024528203	0.671272189	1.50E-02
AC026877.1	5.28E-04	13500.92413	8.570008526	21268934.78	1.13E-02
AC024995.1	3.56E-03	6.040631216	2.315103442	15.76138017	2.37E-04
AC024937.6	4.59E-02	0.031723992	0.002347287	0.428755214	9.39E-03
AC024475.1	5.25E-05	236967.6395	18.39977838	3051866225	1.04E-02
AC024082.3	2.79E-04	1.74607E+18	597193.4935	5.11E+30	4.13E-03
AC023672.2	7.46E-03	8.11E-08	2.45E-14	0.268242918	3.30E-02
AC023469.1	3.38E-06	8671900.307	7200.683286	10443710957	1.01E-05
AC023385.1	5.28E-04	186665.4804	15.51124863	2246369871	1.13E-02
AC022007.5	3.06E-05	0.053342317	0.010943139	0.260017054	2.87E-04
AC021192.1	5.28E-04	1554343.87	25.03622044	96499584381	1.13E-02
AC021087.1	3.61E-03	2.047438871	1.167210223	3.591474651	1.24E-02
AC020595.1	2.76E-06	3.19E+31	16689844495	6.10E+52	3.71E-03
AC020550.7	1.75E-02	146.1598516	1.4966189	14273.97597	3.30E-02
AC019181.3	2.94E-02	0.010402439	0.000217778	0.496885943	2.06E-02
AC019181.2	5.71E-04	5.75E-07	7.91E-12	0.041853189	1.19E-02
AC019178.3	1.99E-03	2.03E+26	34698.48834	1.18E+48	1.78E-02
AC019100.3	1.94E-02	0.041125726	0.003235955	0.522666489	1.39E-02

AC019055.1	1.99E-03	1.33E+23	9796.636255	1.81E+42	1.78E-02
AC019050.1	2.69E-03	1145.638907	25.31582357	51844.59043	2.93E-04
AC018866.1	5.25E-05	2.98E+33	3077707879	2.88E+57	6.23E-03
AC017104.6	2.57E-03	2.375203023	1.509178164	3.738186475	1.85E-04
AC017076.1	1.70E-02	49.11110491	1.321334672	1825.351803	3.48E-02
AC017060.1	5.16E-03	17752963687	16.33145171	1.92982E+19	2.62E-02
AC017028.8	9.45E-03	575.4093702	1.92375239	172109.4383	2.89E-02
AC017028.2	5.16E-03	280.1873337	1.948323916	40293.57816	2.62E-02
AC017028.10	2.73E-03	6742.490054	26.48328389	1716598.753	1.81E-03
AC017002.1	6.67E-03	39270.39008	55.65311248	27710283.72	1.57E-03
AC016907.3	4.85E-04	4.71E-10	9.27E-17	0.002397856	6.42E-03
AC016831.7	4.13E-04	6.813934486	2.527743999	18.36804012	1.49E-04
AC016757.3	3.10E-04	0.299626524	0.142826588	0.62856682	1.43E-03
AC016753.2	2.29E-04	268.2048503	6.315419299	11390.19253	3.46E-03
AC016747.3	2.93E-05	9.54463357	3.554713605	25.6279521	7.58E-06
AC016734.2	1.82E-03	18.60684549	3.406604889	101.630424	7.38E-04
AC016582.2	3.94E-07	5.07E-06	1.46E-09	0.017579941	3.37E-03
AC015933.2	6.02E-03	1.62E-06	1.05E-11	0.248105612	2.86E-02
AC013448.2	8.04E-03	4.08E+21	59771234.79	2.78E+35	2.20E-03
AC013448.1	1.61E-03	5140387.012	258.6867302	1.02145E+11	2.21E-03
AC013402.5	1.99E-03	1.52E+29	108738.636	2.11E+53	1.78E-02
AC012512.1	1.16E-04	2.756787125	1.583093745	4.800647639	3.39E-04
AC012485.2	4.02E-02	1.50E-09	2.52E-18	0.896410349	4.88E-02
AC012462.2	4.21E-02	10497779078	147.0237013	7.49562E+17	1.24E-02
AC012456.4	1.79E-02	3.77E-05	2.88E-08	0.049168405	5.39E-03
AC012442.5	6.09E-08	0.016181445	0.002313218	0.113192602	3.25E-05
AC012368.1	5.80E-03	1169536.148	4.899498602	2.79174E+11	2.70E-02
AC012358.8	6.16E-03	108.4255654	5.745103268	2046.282319	1.77E-03
AC012354.8	8.73E-03	1151.706964	3.206252198	413700.7472	1.89E-02
AC012354.6	1.35E-02	7.846780713	1.466649334	41.9813831	1.61E-02
AC012314.20	1.85E-02	124933.4499	14.78976536	1055349191	1.10E-02
AC011998.2	4.45E-03	2.2004E+19	2.669551736	1.81E+38	4.51E-02
AC011897.2	5.16E-03	7.01157E+15	75.05656707	6.55E+29	2.62E-02

AC011648.1	5.28E-04	2.08E+25	523574.0122	8.27E+44	1.13E-02
AC011516.2	3.74E-03	3.40E-06	2.65E-11	0.436206979	3.59E-02
AC011298.2	1.09E-02	8.34953E+13	1687631.589	4.13E+21	3.91E-04
AC011196.3	5.16E-03	9.82E+26	1565.301964	6.17E+50	2.62E-02
AC010971.1	1.64E-06	1.03E+35	2000448520	5.35E+60	7.61E-03
AC010890.1	1.77E-04	0.135988319	0.041493172	0.445683512	9.87E-04
AC010884.1	7.30E-03	4.16E-06	5.71E-11	0.30331453	3.01E-02
AC010745.2	5.28E-04	1.35E+37	244050654.7	7.52E+65	1.13E-02
AC010609.1	5.16E-03	316962.2448	4.477719659	22436658003	2.62E-02
AC010226.4	2.37E-02	21.24070599	3.112938551	144.9330219	1.81E-03
AC010149.4	5.16E-03	9000.139668	14.89514806	5438181.194	5.33E-03
AC010146.1	1.88E-02	2.08E+20	1.303922982	3.30E+40	4.87E-02
AC010136.2	4.91E-03	0.28568391	0.1319963	0.618315035	1.47E-03
AC010095.6	9.60E-07	142934648.8	4448.701666	4.59242E+12	3.90E-04
AC010086.1	6.20E-03	1.24E+20	114.9952532	1.35E+38	2.90E-02
AC009965.2	1.88E-02	8.90E+21	1.33202248	5.95E+43	4.87E-02
AC009960.4	6.35E-05	8.42E+23	2289440775	3.10E+38	1.28E-03
AC009831.1	1.20E-03	32.81596979	2.024336265	531.9708449	1.40E-02
AC009411.1	3.37E-05	4.51E+23	3872663517	5.25E+37	9.81E-04
AC009312.1	4.22E-02	0.002018249	7.96E-06	0.511766543	2.80E-02
AC009305.1	1.99E-03	1.88214E+19	2121.58905	1.67E+35	1.78E-02
AC009299.5	1.61E-04	3.089143041	1.488311202	6.411834241	2.47E-03
AC009299.3	2.01E-02	1470066.477	61.83154986	34951338767	5.74E-03
AC009276.4	1.10E-05	1.97861E+12	93045.65986	4.21E+19	1.01E-03
AC009274.6	4.14E-02	0.000183113	6.05E-08	0.553935485	3.53E-02
AC009263.2	2.76E-06	3.39E+72	3.43E+23	3.36E+121	3.71E-03
AC009196.1	1.99E-03	78819.26938	7.000390734	887447209.9	1.78E-02
AC009133.12	5.33E-06	12.10013378	3.320460528	44.09425627	1.57E-04
AC008940.1	4.69E-03	6.12E-24	4.10E-46	0.091383911	4.02E-02
AC008171.1	5.28E-04	1478461538	117.8445304	1.85486E+16	1.13E-02
AC008088.4	2.25E-03	4.473810734	2.176162723	9.197374018	4.61E-05
AC008079.10	2.93E-02	154.699547	10.57900615	2262.211544	2.30E-04
AC008060.7	3.48E-04	543.3247106	21.07372131	14008.04997	1.46E-04

AC007879.7	3.03E-02	44916931670	102061.2042	1.97679E+16	2.16E-04
AC007879.3	8.59E-03	1517613.003	67.87643015	33931502031	5.35E-03
AC007879.2	1.76E-02	30791.35735	48.45809083	19565518.81	1.70E-03
AC007679.1	2.12E-03	70113985245	15107.38893	3.25402E+17	1.43E-03
AC007486.1	1.99E-03	55249.4061	6.584009119	463622819.9	1.78E-02
AC007463.2	1.99E-03	1.75E+41	13124120.39	2.32E+75	1.78E-02
AC007392.4	5.16E-03	4.00E+34	12451.26541	1.28E+65	2.62E-02
AC007364.1	1.05E-02	3.74E+25	113.2966665	1.24E+49	3.31E-02
AC007312.3	5.16E-03	8.05369E+11	25.65085863	2.53E+22	2.62E-02
AC007292.7	2.57E-02	0.104260907	0.018104316	0.60042792	1.14E-02
AC007246.3	6.53E-06	3.782113806	1.992772196	7.178133493	4.72E-05
AC007096.1	7.23E-05	527048.6338	38.83368215	7153075553	6.65E-03
AC006989.2	2.33E-02	4134.422957	1.564112389	10928532.57	3.83E-02
AC006960.7	3.39E-03	4.62158E+15	1543.001991	1.38E+28	1.39E-02
AC006946.17	2.40E-03	5.50E-13	2.38E-24	0.127494496	3.45E-02
AC006547.14	6.73E-05	5.51037085	2.431513251	12.48777356	4.34E-05
AC006538.1	2.42E-02	0.301374257	0.100570283	0.903114122	3.22E-02
AC006534.1	2.19E-02	43.21087276	2.718517162	686.8374974	7.62E-03
AC006499.7	4.19E-03	1.94E-11	3.72E-21	0.101314914	3.07E-02
AC006355.3	1.14E-02	5.88236E+17	93.22017489	3.71E+33	2.75E-02
AC006227.1	3.75E-03	7.66E+42	1698028120	3.46E+76	1.25E-02
AC006129.3	3.21E-02	39870.00042	1.862236273	853606471.3	3.73E-02
AC006129.2	1.63E-02	8.276647406	1.845628141	37.11630243	5.77E-03
AC006033.22	1.81E-04	12.66454041	1.785539727	89.8275079	1.11E-02
AC006026.13	2.79E-02	0.352650201	0.144414783	0.861145664	2.21E-02
AC005915.1	4.87E-04	3.82E+33	221293928.9	6.60E+58	9.11E-03
AC005757.6	4.47E-02	218.4062307	1.569607751	30390.57468	3.24E-02
AC005740.6	5.50E-03	0.11365287	0.015190669	0.850322959	3.42E-02
AC005609.17	5.70E-04	7.43E-07	1.06E-11	0.052110133	1.32E-02
AC005538.1	2.09E-02	339.8521307	1.156821123	99842.11772	4.44E-02
AC005523.3	4.31E-02	0.041148251	0.002565001	0.660108276	2.42E-02
AC005498.4	2.82E-03	0.008055462	0.000119635	0.542402819	2.48E-02
AC005488.11	6.48E-03	48378.04845	7.590755972	308327073.1	1.58E-02

AC005357.1	2.85E-02	2478255.269	1.529959026	4.01432E+12	4.36E-02
AC005330.2	1.43E-03	0.385630749	0.172258583	0.863301392	2.05E-02
AC005329.7	7.42E-03	3.14E-10	3.88E-17	0.002536772	7.01E-03
AC005324.7	5.28E-04	3149002762	139.7916528	7.09357E+16	1.13E-02
AC005324.6	1.22E-02	2.67E-50	2.00E-98	0.035695616	4.35E-02
AC005083.1	5.03E-04	9.036753268	2.514090579	32.48208728	7.45E-04
AC005077.14	1.31E-02	3.24E-09	2.14E-16	0.049205814	2.05E-02
AC005034.2	5.28E-04	6.57E+21	84776.42897	5.10E+38	1.13E-02
AC005019.3	1.26E-04	0.17765185	0.044586828	0.707836403	1.43E-02
AC004988.1	1.52E-02	220.5988313	8.939328934	5443.791668	9.70E-04
AC004906.3	6.89E-03	124753689.1	173.8899353	8.95019E+13	6.73E-03
AC004895.1	1.49E-02	94.07505022	1.566997381	5647.817399	2.96E-02
AC004854.4	3.33E-03	0.008883774	0.000126327	0.624738252	2.95E-02
AC004744.3	5.53E-04	8397609.482	25.6347655	2.75095E+12	1.39E-02
AC004702.2	1.05E-03	2656897.029	48.14659897	1.46617E+11	7.92E-03
AC004549.6	1.40E-02	2.50E-07	7.14E-12	0.008785626	4.42E-03
AC004510.3	2.64E-03	3.16E-08	4.86E-14	0.020552127	1.14E-02
AC004453.8	1.23E-03	0.471326482	0.263117442	0.844294665	1.14E-02
AC004159.1	2.55E-03	6352592125	246.414546	1.6377E+17	9.53E-03
AC004129.7	2.79E-03	3.19E-06	1.15E-10	0.08868365	1.54E-02
AC004067.5	1.13E-02	2.091150283	1.044097959	4.188217655	3.74E-02
AC003991.3	4.63E-02	9.60E-26	8.41E-49	0.010966504	3.35E-02
AC003988.1	4.77E-03	7.1066E+17	402.7792202	1.25E+33	2.17E-02
AC003986.6	3.39E-02	91688.01355	2.041796874	4117300765	3.66E-02
AC002454.1	2.37E-03	0.01673593	0.000569269	0.492019501	1.77E-02
AC002383.2	2.76E-06	3.34E+39	6.67847E+12	1.67E+66	3.71E-03
AC002310.12	7.14E-03	0.102619225	0.021728779	0.48464321	4.05E-03
AC000123.3	1.19E-02	6.390301322	2.081093707	19.62235091	1.19E-03
AC000067.1	6.68E-03	3.382229651	1.442493423	7.930349791	5.07E-03
ABTB2	2.56E-02	2.500450613	1.188214321	5.261890179	1.58E-02
ABTB1	6.25E-04	0.292969364	0.115391655	0.743823707	9.81E-03
ABT1	1.75E-02	0.299624265	0.111220535	0.807177382	1.71E-02
ABR	3.89E-04	4.188704183	1.46866813	11.94636308	7.39E-03

ABLIM1	1.02E-02	0.581888078	0.380150222	0.89068404	1.27E-02
ABL2	7.61E-03	3.625770312	1.754682049	7.492075481	5.04E-04
ABI3	5.09E-04	2.071618729	1.388261142	3.091352218	3.62E-04
ABI2	1.06E-03	2.636235179	1.053135016	6.599093008	3.84E-02
ABHD8	5.18E-05	0.250904783	0.116651561	0.539668818	4.03E-04
ABHD6	2.69E-02	0.445359339	0.226840353	0.874381207	1.88E-02
ABHD3	5.05E-03	2.3447737	1.429189031	3.846911489	7.42E-04
ABHD16A	4.27E-02	3.641797463	1.226226238	10.81585792	2.00E-02
ABHD15	2.24E-06	5.801531833	2.201836413	15.28622717	3.76E-04
ABHD14B	2.59E-03	0.464815962	0.275192942	0.785099637	4.17E-03
ABHD12	4.68E-02	2.594979161	1.084426236	6.209658735	3.22E-02
ABHD11	2.80E-05	5.043464017	1.970792526	12.90675145	7.38E-04
ABCF3	2.26E-04	0.30577646	0.142082101	0.658064898	2.45E-03
ABCC9	1.50E-02	12.06974549	1.836717393	79.31473651	9.52E-03
ABCC6P2	2.47E-02	0.001309025	3.15E-06	0.544050947	3.09E-02
ABCC6P1	1.06E-02	2.89E-23	2.13E-43	0.00392972	2.82E-02
ABCC4	1.03E-02	6.389719105	2.819350244	14.48153181	8.87E-06
ABCC12	1.46E-02	5.723307793	2.613671424	12.53265877	1.29E-05
ABCC1	6.27E-04	4.420733019	1.761024697	11.09744824	1.55E-03
ABCB9	3.43E-04	4.221432456	1.260967583	14.13239501	1.95E-02
ABCB8	5.55E-04	8.091592874	2.937960013	22.2854889	5.23E-05
ABCB6	8.69E-04	5.310151426	2.160802549	13.04964592	2.73E-04
ABCB4	5.04E-03	8.877374654	3.393019255	23.22644666	8.60E-06
ABCA3	4.18E-04	0.324535927	0.171206037	0.615186062	5.63E-04
ABCA17P	1.19E-04	0.001419882	2.16E-05	0.093321864	2.14E-03
ABCA12	3.65E-03	7.00E-27	1.67E-46	2.93E-07	8.99E-03
ABC7-42391500H16.2	3.42E-02	95719491656	1.263984788	7.25E+21	4.79E-02
AATBC	2.34E-03	2.087546169	1.371326595	3.177834533	5.97E-04
AAK1	7.59E-03	2.307427027	1.086753253	4.899198112	2.95E-02
AACS	8.41E-04	7.233110929	1.923402666	27.2006973	3.41E-03
A4GALT	1.05E-03	3.795534844	1.613963022	8.925907568	2.23E-03
A1BG-AS1	2.87E-02	0.170472912	0.059819342	0.485812995	9.29E-04
A1BG	1.16E-02	0.003677031	9.01E-05	0.149993013	3.05E-03

Table S5. The correlations of 490 autophagy-related genes with the seven-gene signature risk score examined by the Pearson correlation coefficient.

Gene Symbol	r	P-value
ABL1	0.264694623	1.77E-02
ABL2	0.471741242	1.00E-05
ACER2	-0.084066218	4.58E-01
ADRA1A	0.205245519	6.78E-02
ADRB2	-0.503108362	1.97E-06
AKT1	0.014815738	8.96E-01
AMBRA1	-0.228949693	4.11E-02
APOL1	0.618835385	9.48E-10
ARNT	0.204578072	6.87E-02
ARSA	0.177086005	1.16E-01
ARSB	0.31773936	4.08E-03
ATF4	-0.051953422	6.47E-01
ATF6	0.291402761	8.73E-03
ATG10	0.144063586	2.02E-01
ATG101	0.268030737	1.62E-02
ATG12	0.313316147	4.66E-03
ATG13	-0.141292274	2.11E-01
ATG14	0.194569242	8.37E-02
ATG16L1	0.299224244	7.01E-03
ATG16L2	0.400943471	2.28E-04
ATG2A	-0.295997177	7.68E-03
ATG2B	0.323180105	3.46E-03
ATG3	-0.234022096	3.67E-02
ATG4A	-0.07092666	5.32E-01
ATG4B	0.202460434	7.17E-02
ATG4C	0.01385368	9.03E-01
ATG4D	0.403004924	2.10E-04
ATG5	0.038324505	7.36E-01
ATG7	-0.214212063	5.64E-02
ATG9A	0.076198785	5.02E-01
ATG9B	0.070489082	5.34E-01
ATIC	0.509587084	1.38E-06
ATM	0.33871945	2.12E-03
ATP13A2	-0.095165318	4.01E-01
ATP6V0A1	-0.105992328	3.49E-01
ATP6V0A2	0.284170128	1.06E-02
ATP6V0B	0.639836581	1.67E-10
ATP6V0C	0.237731182	3.37E-02
ATP6V0D1	0.203637944	7.00E-02
ATP6V0D2	0.436249038	5.23E-05
ATP6V0E1	0.495357327	2.99E-06
ATP6V0E2	0.382285034	4.66E-04
ATP6V1A	-0.057295754	6.14E-01
ATP6V1B1	0.244471855	2.89E-02
ATP6V1B2	-0.174485689	1.22E-01
ATP6V1C1	0.417257365	1.18E-04

ATP6V1C2	0.533063624	3.57E-07
ATP6V1D	0.110882515	3.27E-01
ATP6V1E1	0.412678363	1.42E-04
ATP6V1E2	0.046998227	6.79E-01
ATP6V1G1	0.246573606	2.75E-02
ATP6V1G2	-0.364888738	8.75E-04
ATP6V1H	0.511215216	1.26E-06
BAD	0.488207604	4.35E-06
BAG1	0.323402129	3.43E-03
BAG3	0.16427565	1.45E-01
BAK1	0.105666881	3.51E-01
BAX	0.739227776	4.89E-15
BCL2	-0.004454534	9.69E-01
BCL2L1	0.341990504	1.90E-03
BCL2L11	0.324115161	3.36E-03
BECN1	0.059074558	6.03E-01
BID	0.435817535	5.33E-05
BIRC5	0.400102989	2.36E-04
BIRC6	0.257879702	2.09E-02
BMF	0.172722646	1.25E-01
BNIP1	0.649341831	7.25E-11
BNIP3	-0.195956449	8.15E-02
BNIP3L	-0.044896459	6.93E-01
BOK	0.007579847	9.47E-01
C9orf72	0.265419355	1.73E-02
CALCOCO2	0.177416572	1.15E-01
CAMKK2	0.342167489	1.89E-03
CANX	0.31905608	3.92E-03
CAPN1	0.15380185	1.73E-01
CAPN10	0.106478229	3.47E-01
CAPN2	0.233609431	3.70E-02
CAPNS1	0.273785792	1.40E-02
CASP1	0.519931892	7.71E-07
CASP3	0.338008613	2.17E-03
CASP4	0.343527098	1.81E-03
CASP8	0.418431762	1.12E-04
CCL2	0.223463812	4.63E-02
CCR2	0.333547648	2.50E-03
CD46	0.077168196	4.96E-01
CDC37	0.156793401	1.65E-01
CDK5	0.525924915	5.45E-07
CDK5R1	0.411459087	1.50E-04
CDKN1A	0.520351955	7.52E-07
CDKN1B	0.055973715	6.22E-01
CDKN2A	0.173407882	1.24E-01
CFLAR	0.33693375	2.24E-03
CHMP2B	-0.102734724	3.64E-01
CHMP4A	0.486832376	4.67E-06
CHMP4B	0.386174179	4.03E-04

CISD2	0.477920081	7.35E-06
CLEC16A	-0.181756383	1.07E-01
CLN3	0.611049638	1.75E-09
CLU	0.083175245	4.63E-01
CPTP	-0.10513355	3.53E-01
CSNK2A2	-0.561327818	6.08E-08
CTSA	0.532599054	3.67E-07
CTSB	0.171824996	1.28E-01
CTSD	0.109938171	3.32E-01
CTTN	0.239086488	3.27E-02
CX3CL1	0.212407473	5.85E-02
CXCR4	0.456682129	2.06E-05
DAP	0.653518249	4.99E-11
DAPK1	0.239154751	3.26E-02
DAPK2	0.351038539	1.41E-03
DAPK3	0.060903717	5.91E-01
DAPL1	-0.272697464	1.44E-02
DCN	0.125150071	2.69E-01
DDIT3	0.33603117	2.31E-03
DHRSX	-0.015693181	8.90E-01
DIRAS3	0.323707697	3.40E-03
DLC1	-0.618045215	1.01E-09
DNAJB1	0.264053888	1.79E-02
DNAJB9	0.122051241	2.81E-01
DNM1L	0.153590844	1.74E-01
DRAM1	0.2733431	1.42E-02
DRAM2	-0.073704213	5.16E-01
EDEM1	-0.099770883	3.79E-01
EEF1A1	-0.51683474	9.20E-07
EEF1A2	0.609303769	2.00E-09
EEF2	-0.492274492	3.52E-06
EEF2K	0.042684575	7.07E-01
EGFR	0.237079504	3.42E-02
EIF2AK2	0.157534459	1.63E-01
EIF2AK3	0.231638267	3.87E-02
EIF2AK4	0.1337045	2.37E-01
EIF2S1	0.428801889	7.23E-05
EIF4EBP1	0.118035426	2.97E-01
EIF4G1	-0.501264295	2.18E-06
EIF4G2	0.13087093	2.47E-01
EP300	0.139607335	2.17E-01
EPM2A	-0.443123831	3.85E-05
ERBB2	0.006858597	9.52E-01
ERCC4	0.040403982	7.22E-01
ERN1	-0.07314959	5.19E-01
ERO1L	0.163243489	1.48E-01
EXOC1	0.163454252	1.47E-01
EXOC4	0.151386422	1.80E-01
EXOC7	-0.118983354	2.93E-01

EXOC8	0.138054793	2.22E-01
FADD	0.358217032	1.10E-03
FAS	0.362315226	9.58E-04
FBXL2	-0.374278166	6.26E-04
FBXO7	-0.466529491	1.29E-05
FBXW7	0.118525528	2.95E-01
FEZ1	0.604001865	3.00E-09
FEZ2	-0.060622044	5.93E-01
FKBP1A	0.657678157	3.41E-11
FKBP1B	-0.080079753	4.80E-01
FLCN	0.255046773	2.24E-02
FOS	0.329544412	2.84E-03
FOXK1	-0.032548146	7.74E-01
FOXK2	0.218282308	5.18E-02
FOXO1	-0.324350992	3.33E-03
FOXO3	-0.496520211	2.81E-06
FTH1	0.419290686	1.08E-04
FTL	0.467625238	1.22E-05
FYCO1	-0.431664457	6.39E-05
FZD5	0.41605901	1.24E-04
GAA	0.517820841	8.70E-07
GABARAP	-0.146013927	1.96E-01
GABARAPL1	-0.555671188	8.78E-08
GABARAPL2	-0.195646451	8.20E-02
GAPDH	0.32695991	3.07E-03
GATA4	-0.621813189	7.47E-10
GBA	0.607752332	2.26E-09
GFAP	0.110050857	3.31E-01
GNAI3	0.042577592	7.08E-01
GNB2L1	-0.422838801	9.33E-05
GOLGA2	0.266999355	1.67E-02
GOPC	0.070440485	5.35E-01
GPSM1	-0.170099967	1.31E-01
GRID1	0.601925412	3.51E-09
GRID2	0.091080734	4.22E-01
GSK3A	0.239682989	3.22E-02
GSK3B	-0.144646367	2.00E-01
HAX1	0.636993913	2.12E-10
HDAC1	0.3447205	1.74E-03
HDAC6	-0.248931342	2.60E-02
HERC1	-0.091607854	4.19E-01
HGF	0.246464797	2.75E-02
HGS	-0.200438833	7.46E-02
HIF1A	0.219631578	5.03E-02
HK2	0.250086272	2.53E-02
HMGB1	0.230723319	3.95E-02
HMGB4	0.133008315	2.40E-01
HMOX1	0.706194472	2.53E-13
HSP90AA1	0.255072431	2.24E-02

HSP90AB1	-0.464879048	1.40E-05
HSPA5	0.52823189	4.76E-07
HSPA8	0.105345561	3.52E-01
HSPB1	-0.272141433	1.46E-02
HSPB8	0.60409264	2.98E-09
HTR2B	0.814348177	4.08E-20
HTRA2	0.203127707	7.07E-02
HTT	0.193087011	8.62E-02
IFI16	0.326878076	3.08E-03
IFNG	0.429778536	6.94E-05
IFT20	-0.074164752	5.13E-01
IFT88	0.135373152	2.31E-01
IKBKB	-0.042374268	7.09E-01
IKBKE	0.529144526	4.51E-07
IKBKG	-0.147488607	1.92E-01
IL10	0.455346313	2.20E-05
IL10RA	0.560362749	6.48E-08
IL24	0.291847428	8.62E-03
IL4	-0.317496173	4.11E-03
IRGM	0.075118597	5.08E-01
ITGA3	-0.05032669	6.58E-01
ITGA6	0.650968562	6.27E-11
ITGB1	-0.004579507	9.68E-01
ITGB4	-0.22168726	4.81E-02
ITPR1	-0.056658065	6.18E-01
KAT5	-0.079331261	4.84E-01
KAT8	-0.454525444	2.28E-05
KDM4A	0.052459293	6.44E-01
KDR	0.543982024	1.84E-07
KEAP1	0.138802606	2.19E-01
KIAA0226	-0.276433417	1.31E-02
KIAA1324	0.401858789	2.20E-04
KIF25	-0.358079003	1.11E-03
KIF5B	0.284756904	1.05E-02
KLHL22	0.437050163	5.05E-05
KLHL24	-0.465556541	1.35E-05
KLHL3	-0.177263296	1.16E-01
LACRT	NA	NA
LAMP1	-0.171098774	1.29E-01
LAMP2	0.212873137	5.80E-02
LAMP3	0.358017085	1.11E-03
LAMTOR1	0.04028219	7.23E-01
LAMTOR2	0.797163415	9.07E-19
LAMTOR3	0.118866283	2.94E-01
LAMTOR4	0.261440146	1.92E-02
LAMTOR5	0.167903551	1.37E-01
LARP1	0.301691108	6.54E-03
LEP	0.127864127	2.58E-01
LEPR	0.351175263	1.40E-03

LGALS8	0.24076122	3.15E-02
LRRK2	-0.482856419	5.73E-06
LRSAM1	0.446872386	3.25E-05
LZTS1	0.104053401	3.58E-01
MAP1LC3A	0.289682718	9.15E-03
MAP1LC3B	0.139274025	2.18E-01
MAP1LC3C	-0.553794974	9.90E-08
MAP2K7	0.140627873	2.13E-01
MAP3K7	0.122770101	2.78E-01
MAPK1	0.430394091	6.75E-05
MAPK15	0.171405332	1.28E-01
MAPK3	0.123917472	2.73E-01
MAPK8	0.04919311	6.65E-01
MAPK8IP1	-0.195766561	8.18E-02
MAPK9	0.128350899	2.57E-01
MAPT	0.600258499	3.98E-09
MBTPS2	0.19673625	8.03E-02
MCL1	0.471729594	1.00E-05
MEFV	0.452356899	2.52E-05
MET	0.331261401	2.69E-03
MFN2	0.026251049	8.17E-01
MFSD8	0.17088719	1.30E-01
MID2	-0.209073504	6.27E-02
MIR199A1	0.415349407	1.28E-04
MIR199A2	0.024196171	8.31E-01
MLST8	0.482825795	5.74E-06
MT3	0.201978602	7.24E-02
MTCL1	0.474618067	8.67E-06
MTDH	0.44102963	4.23E-05
MTM1	-0.005809795	9.59E-01
MTMR14	-0.7066742	2.40E-13
MTMR3	-0.260254032	1.97E-02
MTMR8	0.200432657	7.46E-02
MTMR9	0.022797581	8.41E-01
MTOR	0.24859926	2.62E-02
MUL1	0.258216902	2.07E-02
MYC	-0.157210586	1.64E-01
NAF1	-0.10608267	3.49E-01
NAMPT	0.166955708	1.39E-01
NBR1	0.124907392	2.70E-01
NCKAP1	0.176970752	1.16E-01
NCOA4	-0.081505198	4.72E-01
NEDD4	0.164844203	1.44E-01
NFE2L2	0.199064076	7.67E-02
NFKB1	0.204640162	6.86E-02
NKX2-3	-0.024027938	8.32E-01
NLRC4	0.552740333	1.06E-07
NLRP6	0.636252772	2.26E-10
NOD1	-0.218221093	5.18E-02

NPC1	0.062056414	5.84E-01
NPRL2	-0.552888432	1.05E-07
NPRL3	0.073600451	5.16E-01
NRBP2	-0.114044869	3.14E-01
NRG1	0.10770078	3.42E-01
NRG2	-0.086974403	4.43E-01
NRG3	0.287909926	9.61E-03
NUPR1	0.009369965	9.34E-01
OPTN	0.096058679	3.97E-01
OSBPL7	-0.240114105	3.19E-02
P4HB	0.528065664	4.80E-07
PAFAH1B2	0.31642267	4.24E-03
PARK2	-0.119777361	2.90E-01
PARK7	0.514913887	1.03E-06
PARP1	0.239750838	3.22E-02
PEA15	0.531154377	4.00E-07
PELP1	-0.102543427	3.65E-01
PEX14	0.216460763	5.38E-02
PEX3	0.239234248	3.26E-02
PHF23	0.07670834	4.99E-01
PIK3C3	0.116645277	3.03E-01
PIK3CA	-0.134332218	2.35E-01
PIK3CB	-0.014868893	8.96E-01
PIK3R2	0.163190812	1.48E-01
PIK3R4	-0.645363766	1.03E-10
PIKFYVE	0.066505226	5.58E-01
PIM2	0.533469854	3.49E-07
PINK1	-0.029438196	7.95E-01
PIP4K2A	0.31459619	4.48E-03
PIP4K2B	-0.033775947	7.66E-01
PIP4K2C	-0.065320625	5.65E-01
PLEKHF1	0.331258567	2.69E-03
PLK2	0.04717729	6.78E-01
PLK3	0.323260348	3.45E-03
POLDIP2	0.134432331	2.34E-01
PPP1R15A	-0.011778356	9.17E-01
PRKAA1	0.258039088	2.08E-02
PRKAA2	-0.050791701	6.55E-01
PRKAB1	0.018860205	8.68E-01
PRKAB2	-0.073907002	5.15E-01
PRKACA	0.437909225	4.86E-05
PRKAG1	0.090271327	4.26E-01
PRKAG2	-0.026701121	8.14E-01
PRKAG3	-0.121747391	2.82E-01
PRKAR1A	-0.158614295	1.60E-01
PRKCD	-0.705361725	2.78E-13
PRKCQ	-0.092131946	4.16E-01
PRKD1	0.221671429	4.81E-02
PSAP	0.56582778	4.52E-08

PTEN	-0.078993989	4.86E-01
PTK2	0.425587328	8.30E-05
PTK6	-0.15358273	1.74E-01
PTPN22	0.455277589	2.20E-05
PYCARD	-0.190168026	9.11E-02
QSOX1	-0.025256657	8.24E-01
RAB11A	0.382499428	4.63E-04
RAB12	0.053189915	6.39E-01
RAB1A	0.384165756	4.35E-04
RAB1B	0.070659103	5.33E-01
RAB24	-0.038954479	7.32E-01
RAB33B	0.127140514	2.61E-01
RAB39B	-0.163720142	1.47E-01
RAB3GAP1	0.138599906	2.20E-01
RAB3GAP2	0.250556641	2.50E-02
RAB5A	-0.430976937	6.58E-05
RAB7A	-0.583392896	1.35E-08
RAB8A	0.505461838	1.73E-06
RAC1	0.18675374	9.72E-02
RAF1	-0.686325386	2.12E-12
RALB	0.437081526	5.04E-05
RASIP1	-0.240483882	3.17E-02
RB1	0.053435426	6.38E-01
RB1CC1	0.250575677	2.50E-02
RELA	-0.200873532	7.40E-02
RGS19	0.614253045	1.36E-09
RHEB	0.372341978	6.71E-04
RIPK2	0.290657159	8.91E-03
RNF152	0.493417318	3.31E-06
RNF41	0.203375541	7.04E-02
RNF5	-0.381278146	4.84E-04
ROCK1	0.111294713	3.26E-01
RPS6KB1	0.095394089	4.00E-01
RPTOR	0.094775022	4.03E-01
RRAGA	0.082657777	4.66E-01
RRAGB	-0.135544002	2.31E-01
RRAGC	-0.025204954	8.24E-01
RRAGD	-0.192210661	8.76E-02
RUFY4	0.436396136	5.20E-05
SAR1A	0.389071012	3.61E-04
SCFD1	0.353748784	1.29E-03
SCOC	-0.085620983	4.50E-01
SEC22B	0.255825567	2.20E-02
SERPINA1	0.35668498	1.16E-03
SESN1	-0.209138162	6.26E-02
SESN2	0.480454135	6.47E-06
SESN3	-0.071651436	5.28E-01
SH3BP4	-0.18483332	1.01E-01
SH3GLB1	0.273479324	1.41E-02

SIRT1	0.008351642	9.41E-01
SIRT2	-0.249239185	2.58E-02
SLC38A9	0.54494674	1.73E-07
SMCR8	0.062813892	5.80E-01
SMURF1	-0.321626768	3.62E-03
SNCA	-0.565601219	4.58E-08
SNRNP70	0.206380811	6.63E-02
SNX32	0.396245474	2.74E-04
SNX5	0.051202307	6.52E-01
SNX6	0.206037082	6.67E-02
SOGA1	0.308710715	5.33E-03
SOGA3	-0.395958178	2.77E-04
SPHK1	0.654844271	4.42E-11
SPNS1	0.048367902	6.70E-01
SPTLC1	0.218752541	5.12E-02
SPTLC2	0.210315729	6.11E-02
SQSTM1	0.321361358	3.65E-03
SREBF1	0.214016456	5.66E-02
SREBF2	0.050865695	6.54E-01
ST13	-0.655595056	4.13E-11
STAT3	0.489051749	4.16E-06
STK11	0.056993307	6.16E-01
STUB1	-0.222249279	4.75E-02
STX12	0.131823673	2.44E-01
SUPT5H	-0.282881596	1.10E-02
SVIP	-0.071288266	5.30E-01
SYNPO2	0.241085738	3.12E-02
TAB2	-0.125510713	2.67E-01
TAB3	-0.187805231	9.53E-02
TBC1D12	-0.056139573	6.21E-01
TBC1D14	0.027228735	8.11E-01
TBC1D25	-0.113504046	3.16E-01
TBK1	0.362613901	9.48E-04
TEX264	0.019713918	8.62E-01
TFEB	0.194252542	8.42E-02
TICAM1	0.608429965	2.14E-09
TLK2	0.23214879	3.83E-02
TM9SF1	0.530904511	4.06E-07
TMEM150A	-0.333564995	2.50E-03
TMEM150B	0.517584768	8.81E-07
TMEM150C	-0.011264765	9.21E-01
TMEM59	0.075449446	5.06E-01
TMEM74	0.102124157	3.67E-01
TNFSF10	0.480663165	6.40E-06
TOMM7	-0.574883398	2.45E-08
TP53	0.182362687	1.05E-01
TP53INP1	0.414460846	1.32E-04
TP53INP2	0.476635222	7.84E-06
TP63	0.223120935	4.67E-02

TP73	0.329202913	2.87E-03
TPCN1	0.269915814	1.55E-02
TPCN2	-0.253311023	2.34E-02
TRAPPC8	0.106180064	3.49E-01
TREM2	0.26672456	1.68E-02
TRIB3	0.275115784	1.35E-02
TRIM13	0.420419801	1.03E-04
TRIM21	0.313458174	4.64E-03
TRIM22	0.268971838	1.58E-02
TRIM65	0.504607623	1.82E-06
TRIM8	-0.156797288	1.65E-01
TSC1	-0.01044583	9.27E-01
TSC2	-0.277855538	1.26E-02
TSPO	-0.184868066	1.01E-01
TUSC1	-0.560011431	6.63E-08
UBQLN1	-0.087345223	4.41E-01
UBQLN2	-0.118317238	2.96E-01
UBQLN4	-0.479890682	6.66E-06
UCHL1	0.605164404	2.75E-09
ULK1	-0.023842415	8.34E-01
ULK2	0.334974016	2.39E-03
ULK3	0.633993092	2.74E-10
USP10	-0.054117689	6.34E-01
USP13	-0.292638596	8.43E-03
USP30	-0.038026976	7.38E-01
USP33	0.108688921	3.37E-01
UVRAG	-0.18890037	9.33E-02
VAMP3	-0.251168428	2.46E-02
VAMP7	0.220553846	4.93E-02
VDAC1	0.608040872	2.21E-09
VEGFA	0.242671598	3.01E-02
VMP1	0.459863889	1.78E-05
VPS13C	0.25720273	2.13E-02
VPS13D	0.165061447	1.43E-01
VPS26A	0.171426639	1.28E-01
VPS26B	0.264362705	1.78E-02
VPS35	0.020077597	8.60E-01
WAC	0.130673212	2.48E-01
WDFY3	0.128463978	2.56E-01
WDR24	-0.090368309	4.25E-01
WDR41	0.26090132	1.94E-02
WDR45	0.426713822	7.91E-05
WDR45B	0.100623215	3.74E-01
WDR6	-0.544246021	1.81E-07
WDR81	0.003859122	9.73E-01
WIPI1	0.615928803	1.19E-09
WIPI2	-0.118866906	2.94E-01
XPA	0.241084312	3.12E-02
ZC3H12A	0.398966694	2.47E-04

ZFYVE1	-0.140657625	2.13E-01
ZKSCAN3	-0.451426683	2.64E-05
ZMPSTE24	0.40513486	1.93E-04

Table S6. Enriched gene sets in HALLMARK collection (| NES |> 1, NOM p-val <0.05, and FDR q-val <0.25).

NAME	SIZE	ES	NES	NOM p-val	FDR q-val
HALLMARK_IL6_JAK_STAT3_SIGNALING	87	0.7491942	1.7455785	0	0.049437765
HALLMARK_NOTCH_SIGNALING	32	0.6615237	1.6149615	0.004081633	0.063202575
HALLMARK_GLYCOLYSIS	198	0.56929886	1.6073792	0.008016032	0.05706074
HALLMARK_ALLOGRAFT_REJECTION	195	0.72458875	1.6431289	0.009746589	0.07224934
HALLMARK_REACTIVE_OXYGEN_SPECIES_PATHWAY	47	0.62119776	1.6834254	0.011673152	0.062074985
HALLMARK_IL2_STAT5_SIGNALING	195	0.5801725	1.556839	0.011881189	0.06232403
HALLMARK_ESTROGEN_RESPONSE_LATE	197	0.53945935	1.5825462	0.017475728	0.063670136
HALLMARK_COMPLEMENT	200	0.59321105	1.5822693	0.019267824	0.05631583
HALLMARK_APOPTOSIS	159	0.55616003	1.5371473	0.019646365	0.060964078
HALLMARK_EPITHELIAL_MESENCHYMAL_TRANSITION	197	0.6381576	1.6280315	0.023809524	0.06660893
HALLMARK_INFLAMMATORY_RESPONSE	198	0.6486952	1.5765752	0.0251938	0.053769764
HALLMARK_UV_RESPONSE_UP	154	0.49414575	1.4919928	0.025793651	0.07211612
HALLMARK_TNFA_SIGNALING_VIA_NFKB	198	0.6146418	1.554967	0.030947777	0.05800641
HALLMARK_COAGULATION	138	0.5220003	1.4977033	0.033864543	0.07286198
HALLMARK_HYPOXIA	191	0.5179352	1.478064	0.03468208	0.07719682
HALLMARK_ANGIOGENESIS	36	0.62482554	1.5032912	0.035643563	0.074198015
HALLMARK_KRAS_SIGNALING_UP	194	0.5733329	1.4762918	0.037181996	0.07423924
HALLMARK_INTERFERON_GAMMA_RESPONSE	198	0.6784527	1.5239024	0.041666668	0.0649747
HALLMARK_OXIDATIVE_PHOSPHORYLATION	184	0.56729764	1.5456758	0.04263566	0.059190802

Table S7. Correlations of risk score with 22 kinds of TICs tested by Spearman coefficient.

TIC	r	p-value
T cells CD4 memory activated	0.561737141	5.92E-08
T cells CD8	0.534360169	3.30E-07
Monocytes	-0.522176517	6.77E-07
Macrophages M1	0.510247178	1.33E-06
T cells CD4 memory resting	-0.477094675	7.66E-06
Dendritic cells resting	0.465056901	1.38E-05
Macrophages M2	-0.453563057	2.93E-05
T cells follicular helper	0.39203756	0.000322644
Mast cells resting	-0.340084388	0.002134905
B cells naive	-0.327594174	0.003014135
T cells regulatory (Tregs)	0.317410077	0.004118949
Mast cells activated	0.19245009	0.087217461
Dendritic cells activated	-0.164156263	0.145657968
T cells CD4 naive	-0.157977833	0.161643933
Macrophages M0	0.146891266	0.193524466
B cells memory	0.107851334	0.34096981
NK cells activated	0.090851536	0.422858961
Plasma cells	-0.084119186	0.458172319
Eosinophils	-0.04498895	0.691912444
NK cells resting	-0.042893067	0.705591683
T cells gamma delta	0.010964918	0.923097612
Neutrophils	-0.001241336	0.991280808

Table S8. Prognostic capacity of 22 TICs examined by Kaplan–Meier analysis.

TIC	P-value
Mast cells activated	0.001537564
T cells CD4 memory activated	0.038521927
Mast cells resting	0.039551499
T cells CD8	0.049309347
Monocytes	0.05406387
T cells CD4 memory resting	0.100353616
Macrophages M2	0.117285657
Macrophages M1	0.136789317
Dendritic cells resting	0.162790485
B cells memory	0.286033745
NK cells activated	0.296002627
T cells gamma delta	0.456956442
T cells follicular helper	0.51269323
B cells naive	0.54351247
Eosinophils	0.656681667
Neutrophils	0.66305121
Plasma cells	0.839336817
NK cells resting	0.849289694
Dendritic cells activated	0.882318948
T cells CD4 naive	0.913661644
T cells regulatory (Tregs)	0.93753452
Macrophages M0	0.989703529
